# Genome-wide identification of enhancers and transcription factors regulating the myogenic differentiation of bovine satellite cells

**DOI:** 10.1186/s12864-021-08224-7

**Published:** 2021-12-16

**Authors:** Pengcheng Lyu, Robert E. Settlage, Honglin Jiang

**Affiliations:** 1 Department of Animal and Poultry Sciences, Virginia Tech, Blacksburg, VA 24061 USA; 2 Advanced Research Computing, Virginia Tech, Blacksburg, VA 24061 USA

**Keywords:** Cattle, Enhancer, Skeletal muscle, Transcription factor

## Abstract

**Background:**

Satellite cells are the myogenic precursor cells in adult skeletal muscle. The objective of this study was to identify enhancers and transcription factors that regulate gene expression during the differentiation of bovine satellite cells into myotubes.

**Results:**

Chromatin immunoprecipitation followed by deep sequencing (ChIP-seq) was performed to identify genomic regions where lysine 27 of H3 histone is acetylated (H3K27ac), i.e., active enhancers, from bovine satellite cells before and during differentiation into myotubes. A total of 19,027 and 47,669 H3K27ac-marked enhancers were consistently identified from two biological replicates of before- and during-differentiation bovine satellite cells, respectively. Of these enhancers, 5882 were specific to before-differentiation, 35,723 to during-differentiation, and 13,199 common to before- and during-differentiation bovine satellite cells. Whereas most of the before- or during-differentiation-specific H3K27ac-marked enhancers were located distally to the transcription start site, the enhancers common to before- and during-differentiation were located both distally and proximally to the transcription start site. The three sets of H3K27ac-marked enhancers were associated with functionally different genes and enriched with different transcription factor binding sites. Specifically, many of the H3K27ac-marked enhancers specific to during-differentiation bovine satellite cells were associated with genes involved in muscle structure and development, and were enriched with binding sites for the MyoD, AP-1, KLF, TEAD, and MEF2 families of transcription factors. A positive role was validated for Fos and FosB, two AP-1 family transcription factors, in the differentiation of bovine satellite cells into myotubes by siRNA-mediated knockdown.

**Conclusions:**

Tens of thousands of H3K27ac-marked active enhancers have been identified from bovine satellite cells before or during differentiation. These enhancers contain binding sites not only for transcription factors whose role in satellite cell differentiation is well known but also for transcription factors whose role in satellite cell differentiation is unknown. These enhancers and transcription factors are valuable resources for understanding the complex mechanism that mediates gene expression during satellite cell differentiation. Because satellite cell differentiation is a key step in skeletal muscle growth, the enhancers, the transcription factors, and their target genes identified in this study are also valuable resources for identifying and interpreting skeletal muscle trait-associated DNA variants in cattle.

**Supplementary Information:**

The online version contains supplementary material available at 10.1186/s12864-021-08224-7.

## Background

Skeletal muscle is the largest tissue in the body and plays an important role in physiology [1, 2]. Skeletal muscle from meat-producing animals is a major source of food for humans and animals. Adult skeletal muscle is composed of mostly muscle fibers [3]. A muscle fiber, also known as a myofiber, is a multinucleated muscle cell differentiated and fused from multiple mononuclear muscle cells called myoblasts. For most mammals, the total number of myofibers is determined prenatally [4]; thus, postnatal skeletal muscle growth results primarily from myofiber hypertrophy [4, 5]. Postnatal myofiber hypertrophy, however, requires additional nuclei [6, 7]. In postnatal animals, nuclei added to the existing myofibers are widely believed to come from satellite cells, which are mononuclear cells located near myofibers and are considered stem cells in adult skeletal muscle [8–10]. Satellite cells are normally quiescent but can be activated by muscle injury and nutritional and environmental changes [6, 7]. Once activated, satellite cells become and proliferate as myoblasts and then differentiate and fuse with each other to generate new myotubes, the developing myofibers, or with existing myofibers to increase muscle fiber size.

A set of four transcription factors called myogenic regulatory factors (MRFs) are known to play important roles in myogenesis, the formation of muscle fibers from myoblasts or satellite cells [11, 12]. These MRFs include myogenic differentiation 1 (MYOD1, also known as MyoD and MYF3), myogenic factor 5 (MYF5), myogenin (MYOG, also known as MYF4), and myogenic factor 6 (MYF6, also known as MRF4 and herculin). All four MRFs are specifically or preferentially expressed in skeletal muscle [12]. All four MRFs are basic helix-loop-helix (bHLH) domain-containing transcription factors and regulate gene transcription by binding to the E-box sequence, CANNTG, where N is A, G, C, or T [13]. MYF5 and MYOD1 determine the myogenic lineage of stem cells in a redundant manner [11, 12]. MYOG is essential for myoblast differentiation and fusion into myotubes [12, 14]. MYF6 was thought to play a similar role to MYOG in myoblast differentiation, but a more recent study indicated an unexpected negative role of MYF6 in postnatal skeletal muscle growth [15].

Clearly, differentiation and fusion of myoblasts or satellite cells into myotubes is a key step in the development and growth of skeletal muscle. The objective of this study was to further understand the regulation of gene expression during the differentiation of bovine satellite cells into myotubes. Cattle are agriculturally important animals, and a better understanding of gene regulation during satellite cell differentiation could lead to the development of novel strategies to improve growth efficiency and meat quality in cattle. Enhancers are DNA sequences that enhance the transcription of associated genes when bound by sequence-specific transcription factors. Active enhancers are genomic regions that are bound by active transcription factors and that actively regulate gene transcription. Genomic regions containing active enhancers are found to be uniquely marked with H3K27ac, where lysine 27 of histone 3 protein is acetylated [16, 17]. We began this study by identifying genomic regions with H3K27ac modification in bovine satellite cells before and during induced differentiation and fusion into myotubes through chromatin immunoprecipitation coupled with deep sequencing (ChIP-seq). To our knowledge, such an approach had not been taken to study the transcriptional mechanisms that control gene expression during the myogenic differentiation of bovine satellite cells or satellite cells from any species.

## Results

### H3K27ac-marked enhancers in bovine satellite cells before and during differentiation

Four ChIP-seq libraries and two Input libraries constructed from bovine satellite cells immediately before and 2 days after induction of differentiation passed the quality control, and deep sequencing generated 23 to 40 million sequencing reads from these libraries (Table 1). Between 75 and 92% of these reads were uniquely mapped to the bovine genome, generating approximately 20 to 36 million uniquely mapped reads per library (Table 1).

**Table 1:** Mapping summary of ChIP-seq libraries

Experiment	Library^a^	Quality filtered reads	Mapped reads	Mapping rate (%)	Uniquely mapped reads	Uniquely mapping Rate (%)
1	Input	33,653,601	29,089,550	86.4	25,223,784	75.0
1	H3K27ac_BD	29,711,647	27,459,149	92.4	24,819,411	83.5
1	H3K27ac_DD	39,617,976	38,013,420	96.0	36,139,994	91.2
2	Input	24,480,296	21,751,323	88.9	19,052,773	77.8
2	H3K27ac_BD	23,063,317	21,580,412	93.6	19,676,362	85.3
2	H3K27ac_DD	31,550,183	30,421,776	96.4	29,009,219	92.0

^a^*BD* before differentiation; *DD* during differentiation

Analyzing the uniquely mapped reads from each ChIP-seq library against those from the corresponding Input library using the MACS peak calling program identified more than 30,000 and 50,000 H3K27ac-marked genomic regions, i.e., active enhancers, from before-differentiation (BD) and during-differentiation (DD) bovine satellite cells, respectively. A phantompeakqualtools analysis indicated that the four ChIP-seq libraries had normalized strand cross-correlation (NSC) values between 1.16 and 1.18 and relative strand cross-correlation (RSC) values between 0.98 and 1.06 (Additional File 1), indicating strong enrichment of reads in peaks [18].

**Fig. 1:**
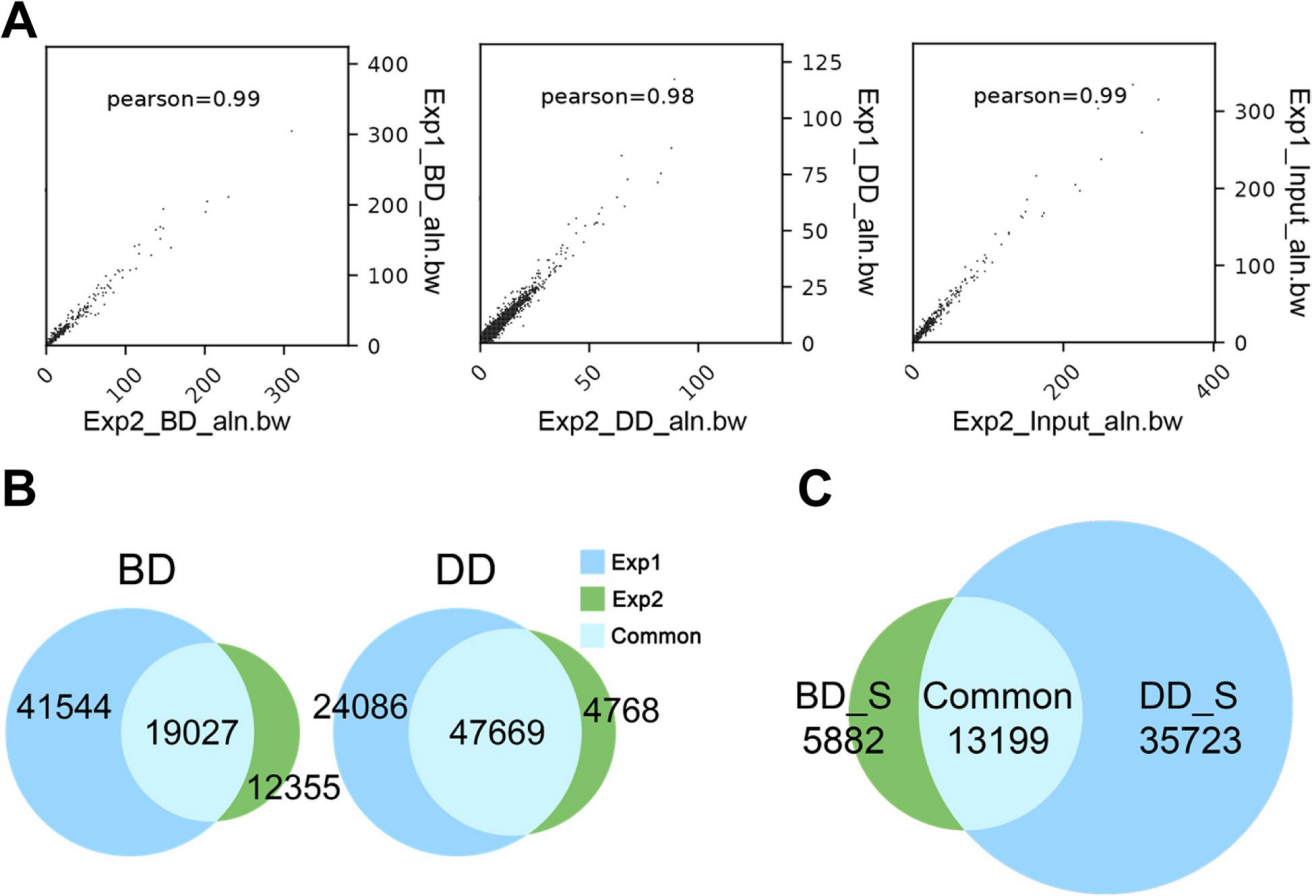
Identification of H3K27ac-marked enhancers in before-differentiation (BD) and during-differentiation (DD) bovine satellite cells. **A** Pearson correlation analyses of ChIP-seq and Input libraries from two biological replicates. **B** Numbers of H3K27ac-marked enhancers consistently identified from two experiments. **C** Numbers of H3K27ac-marked enhancers specific to BD or DD bovine satellite cells or common to both BD and DD bovine satellite cells

A Pearson correlation analysis revealed that the H3K27ac-marked enhancer regions identified from two biological replicates, which corresponded to BD or DD satellite cells originally isolated from two different cattle, were highly correlated (Fig. 1A). A total of 19,027 H3K27ac-marked enhancers were consistently identified from two biological replicates of BD bovine satellite cells, while 47,669 H3K27ac-marked enhancers were consistently identified from two biological replicates of DD bovine satellite cells (Fig. 1B). A total of 5882 H3K27ac-marked enhancers were found to be specific to BD bovine satellite cells, 35,723 H3K27ac-marked enhancers specific to DD bovine satellite cells, and 13,199 H3K27ac-marked enhancers common to both BD and DD bovine satellite cells (Fig. 1C, Additional Files 2, 3, 4).

**Fig. 2:**
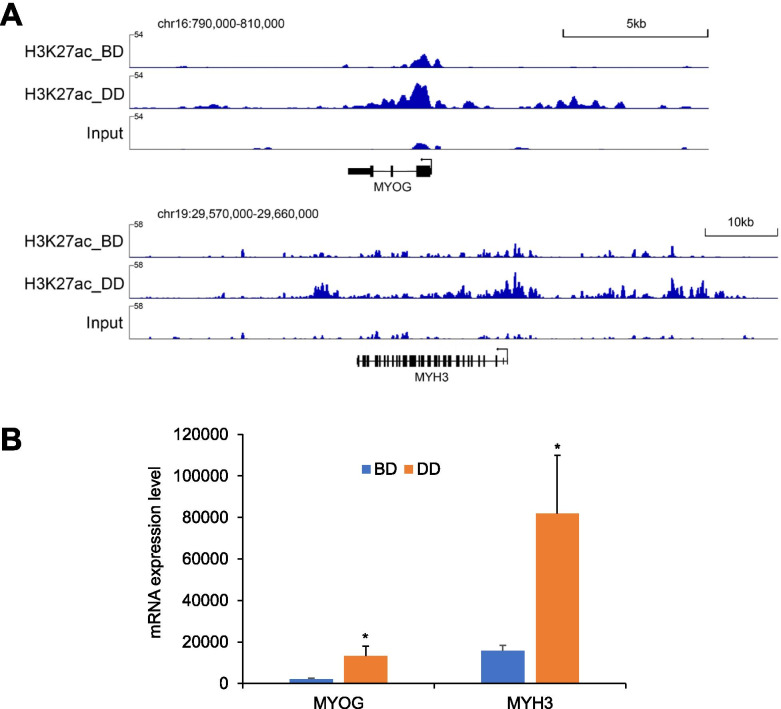
Examples of H3K27ac-marked enhancers in bovine satellite cells. **A** IGV tracks showing H3K27ac-marked enhancers associated with the myogenin (MYOG) and myosin heavy chain 3 (MYH3) genes in before-differentiation (BD) and during-differentiation (DD) bovine satellite cells. **B** Relative expression levels of MYOG and MHY3 mRNAs in BD and DD bovine satellite cells. *P < 0.05 (*n* = 4). Gene expression data was retrieved from a previous study [19]

Examples of H3K27ac-marked enhancers identified from BD and DD bovine satellite cells are shown in Table 2 and Fig. 2A. These enhancers were associated with the MYOG gene, which, as mentioned above, is a transcription factor essential for myoblast differentiation, and myosin heavy chain 3 (MYH3) gene, which, as indicated by its name, encodes a skeletal muscle-specific myosin heavy chain protein. As shown in Table 2 and Fig. 2A, MYOG-associated enhancers were marked with H3K27ac only in DD bovine satellite cells; MYH3-associated enhancers were marked with H3K27ac in both BD and DD bovine satellite cells but more MYH3-associated enhancers were marked with H3K27ac in DD than in BD bovine satellite cells. As shown by Fig. 2B, the increased H3K27ac modification to MYOG- and MYH3-associated genomic regions in DD satellite cells was accompanied by increased expression of both genes in these cells.

**Table 2:** Examples of H3K27ac-marked enhancers and associated genes in bovine satellite cells

Chr	PeakStart	PeakEnd	GeneStart	GeneEnd	GeneSymbol	CellStage^a^
chr16	782,062	782,253	797,555	800,395	MYOG	DD
chr16	798,279	800,338	797,555	800,395	MYOG	DD
chr16	800,596	800,870	797,555	800,395	MYOG	DD
chr16	801,536	801,833	797,555	800,395	MYOG	DD
chr16	804,051	804,227	797,555	800,395	MYOG	DD
chr16	804,761	805,609	797,555	800,395	MYOG	DD
chr16	806,018	806,188	797,555	800,395	MYOG	DD
chr16	806,808	807,049	797,555	800,395	MYOG	DD
chr16	830,439	830,694	797,555	800,395	MYOG	DD
chr16	834,418	835,077	797,555	800,395	MYOG	DD
chr16	836,171	836,762	797,555	800,395	MYOG	DD
chr16	837,735	838,159	797,555	800,395	MYOG	DD
chr16	838,508	838,769	797,555	800,395	MYOG	DD
chr16	839,360	839,542	797,555	800,395	MYOG	DD
chr19	29,582,805	29,583,077	29,601,526	29,622,481	MYH3	DD
chr19	29,594,980	29,595,243	29,601,526	29,622,481	MYH3	DD
chr19	29,595,616	29,597,652	29,601,526	29,622,481	MYH3	DD
chr19	29,598,274	29,598,915	29,601,526	29,622,481	MYH3	DD
chr19	29,603,029	29,603,187	29,601,526	29,622,481	MYH3	DD
chr19	29,612,384	29,612,573	29,601,526	29,622,481	MYH3	DD
chr19	29,613,638	29,613,770	29,601,526	29,622,481	MYH3	DD
chr19	29,614,001	29,614,998	29,601,526	29,622,481	MYH3	DD
chr19	29,618,302	29,618,478	29,601,526	29,622,481	MYH3	DD
chr19	29,624,364	29,624,841	29,601,526	29,622,481	MYH3	DD
chr19	29,625,585	29,626,580	29,601,526	29,622,481	MYH3	DD
chr19	29,631,500	29,631,690	29,601,526	29,622,481	MYH3	DD
chr19	29,632,712	29,632,903	29,601,526	29,622,481	MYH3	DD
chr19	29,634,635	29,634,790	29,601,526	29,622,481	MYH3	DD
chr19	29,635,013	29,635,603	29,601,526	29,622,481	MYH3	DD
chr19	29,635,962	29,636,178	29,601,526	29,622,481	MYH3	DD
chr19	29,636,376	29,636,759	29,601,526	29,622,481	MYH3	DD
chr19	29,638,316	29,638,761	29,601,526	29,622,481	MYH3	DD
chr19	29,615,976	29,616,342	29,601,526	29,622,481	MYH3	BD&DD
chr19	29,621,065	29,621,208	29,601,526	29,622,481	MYH3	BD&DD
chr19	29,622,186	29,622,458	29,601,526	29,622,481	MYH3	BD&DD
chr19	29,623,347	29,623,592	29,601,526	29,622,481	MYH3	BD&DD
chr19	29,641,704	29,641,866	29,601,526	29,622,481	MYH3	BD&DD

^a^Differentiation stage of cells from which H3K27ac-marked peaks were identified. *BD* before differentiation; *DD* during differentiation

### Genomic distribution of H3K27ac-marked enhancers in bovine satellite cells

**Fig. 3:**
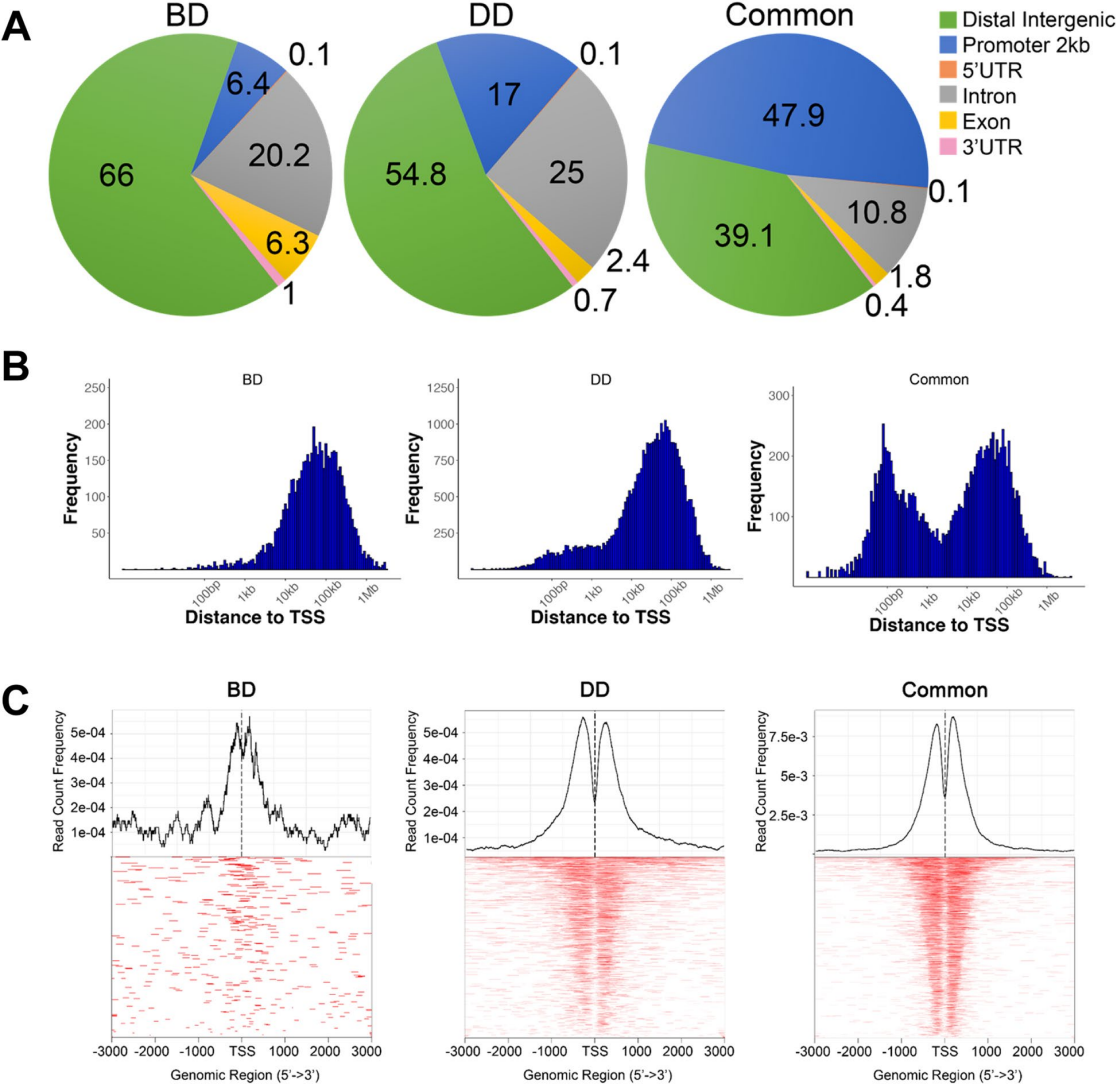
Genomic distribution and location of H3K27ac-marked enhancers in bovine satellite cells. **A** Percentages of H3K27ac-marked enhancers located in various genomic regions. BD, before differentiation; DD, during differentiation; UTR, untranslated region. **B** Distances of H3K27ac-marked enhancers from the transcription start sites (TSS). (C) Frequency of proximal H3K27ac-marked enhancers by distance from the TSS

H3K27ac-marked enhancers specific to BD or DD bovine satellite cells had different genomic distribution from the H3K27ac-marked enhancers common to both BD and DD bovine satellite cells. Whereas nearly 90% of H3K27ac-marked enhancers specific to BD or DD bovine satellite cells were located in the distal intergenic regions and introns, this percentage was only 60% for H3K27ac-marked enhancers common to BD and DD bovine satellite cells (Fig. 3A). Whereas approximately 6 and 17% of H3K27ac-marked enhancers specific to BD and DD bovine satellite cells, respectively, were located in the promoter regions, this percentage was almost 50% for H3K27ac-marked enhancers common to BD and DD bovine satellite cells (Fig. 3A). Whereas H3K27ac-marked enhancers specific to BD or DD bovine satellite cells were concentrated at 100,000 bp from the transcription start site (TSS), those common to BD and DD bovine satellite cells were concentrated at both 100 bp and 100,000 bp from the TSS (Fig. 3B and C).

### Expression levels of genes associated with and without H3K27ac modification in bovine satellite cells

**Fig. 4:**
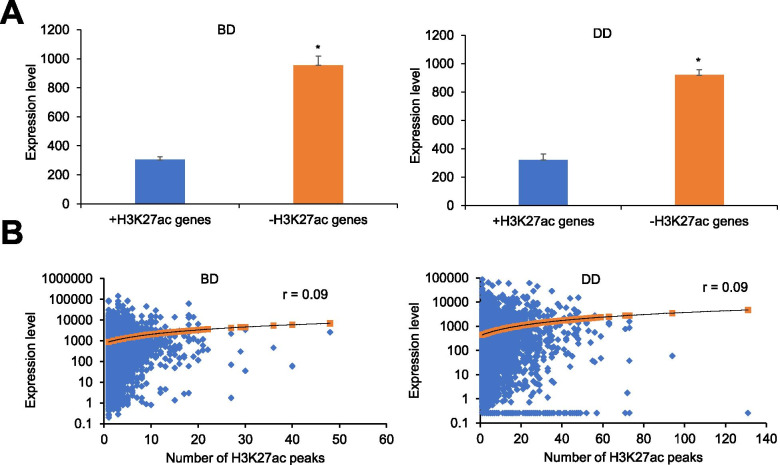
Association of H3K27ac modification with gene expression level in bovine satellite cells. **A** Genes with H3K27ac (+H3K27ac) modification were expressed at greater levels than genes without H3K27ac (−H3K27ac) modification in both before-differentiation (BD) and during-differentiation (DD) bovine satellite cells. *P < 0.05. **B** Pearson correlation analyses revealed that the numbers of H3K27ac-marked enhancers were not correlated with the expression levels of associated genes in either BD or DD bovine satellite cells

We compared the expression levels of genes associated with H3K27ac modification with those without H3K27ac modification in bovine satellite cells. The transcriptome data of BD and DD bovine satellite cells were generated in a previous study [19]. As shown in Fig. 4A, in both BD and DD bovine satellite cells, genes associated with H3K27ac modification were expressed at levels nearly three-fold those of genes without H3K27ac modification. Overall, 5882 H3K27ac-marked enhancer regions specific to BD bovine satellite cells were associated with 3064 protein-coding genes; 35,723 H3K27ac-marked enhancers specific to AD bovine satellite cells were associated with 7649 protein-coding genes; 13,199 H3K27ac-marked enhancers common to BD and DD bovine satellite cells were associated with 6337 protein-coding genes. Obviously, many genes were associated with more than one H3K27ac-marked enhancers in bovine satellite cells. However, the numbers of H3K27ac-marked enhancers were not correlated with the expression levels of associated genes, regardless of the differentiation stage of the cells (Fig. 4B).

### Functional terms enriched in genes associated with H3K27ac modification in bovine satellite cells

We performed gene ontology (GO) enrichment analyses on genes associated with H3K27ac modification in bovine satellite cells. Top biological processes and cellular components enriched in genes associated with H3K27ac modification specifically in DD bovine satellite cells were related to skeletal muscle structure, development, and adaptation, and cell cycle arrest (Tables 3 and 4). It is interesting to note that many genes involved in melanosome and phagocytic vesicle were also associated with H3K27ac modification in DD bovine satellite cells (Table 4). Most of the top molecular functions enriched in genes associated with H3K27ac modification specifically in DD bovine satellite cells were related to growth factor binding and serine and threonine kinase signaling (Table 5). Top biological processes, cellular components, and molecular functions enriched in genes associated with H3K27ac modification in BD bovine satellite cells included pentose metabolic process (Additional File 5), postsynaptic membrane (Additional File 6), and transmembrane receptor protein tyrosine kinase activity (Additional File 7), respectively, which were different from those enriched in genes associated with H3K27ac modification in DD satellite cells (Tables 3, 4 and 5). Top biological processes, cellular components, and molecular functions enriched in genes associated with H3K27ac modification in both BD and DD bovine satellite cells included those related to proteasome and autophagosome (Additional Files 8, 9 and 10).

**Table 3:** Top 10 GO biological processes enriched in genes associated with H3K27ac-marked enhancers in during-differentiation bovine satellite cells

GO biological process	FE^a^	*P*-value	FDR^b^
sarcomere organization (GO:0045214)	2.48	7.92E-04	2.10E-02
striated muscle cell development (GO:0055002)	2.38	5.82E-05	2.14E-03
myofibril assembly (GO:0030239)	2.37	7.88E-05	2.82E-03
regulation of muscle adaptation (GO:0043502)	2.12	9.64E-04	2.51E-02
negative regulation of G1/S transition of mitotic cell cycle (GO:2000134)	2.06	2.06E-03	4.82E-02
muscle cell development (GO:0055001)	2.03	1.78E-06	8.88E-05
skeletal muscle tissue development (GO:0007519)	1.94	4.96E-05	1.85E-03
skeletal muscle organ development (GO:0060538)	1.93	4.87E-05	1.82E-03
positive regulation of cellular amide metabolic process (GO:0034250)	1.92	1.24E-05	5.19E-04
negative regulation of mitotic cell cycle phase transition (GO:1901991)	1.92	1.08E-05	4.60E-04

^a^Fold enrichment

^b^False discovery rate

**Table 4:** Top 10 GO cellular components enriched in genes associated with H3K27ac-marked enhancers in during-differentiation bovine satellite cells

GO cellular component	FE^a^	*P*-value	FDR^b^
Z disc (GO:0030018)	2.24	1.06E-06	2.96E-05
I band (GO:0031674)	2.10	2.90E-06	7.53E-05
myofibril (GO:0030016)	2.10	1.99E-09	8.02E-08
sarcomere (GO:0030017)	2.08	2.68E-08	8.83E-07
contractile fiber (GO:0043292)	2.08	1.73E-09	7.30E-08
nuclear matrix (GO:0016363)	2.00	3.76E-04	6.82E-03
nuclear periphery (GO:0034399)	1.93	2.53E-04	4.77E-03
actin filament bundle (GO:0032432)	1.93	1.54E-03	2.37E-02
phagocytic vesicle (GO:0045335)	1.92	3.86E-04	6.94E-03
melanosome (GO:0042470)	1.87	6.24E-04	1.08E-02

^a^Fold enrichment

^b^False discovery rate

**Table 5:** Top 10 GO molecular functions enriched in genes associated with H3K27ac-marked enhancers in during-differentiation bovine satellite cells

GO molecular function	FE^a^	*P*-value	FDR^b^
growth factor binding (GO:0019838)	1.74	4.24E-04	2.61E-02
protein serine kinase activity (GO:0106310)	1.63	6.99E-05	6.20E-03
protein threonine kinase activity (GO:0106311)	1.62	9.06E-05	7.44E-03
isomerase activity (GO:0016853)	1.62	1.54E-04	1.16E-02
ubiquitin protein ligase binding (GO:0031625)	1.60	2.38E-05	2.24E-03
protein domain specific binding (GO:0019904)	1.59	2.48E-09	3.14E-07
ligase activity (GO:0016874)	1.59	3.47E-04	2.20E-02
identical protein binding (GO:0042802)	1.57	1.29E-26	4.39E-24
kinase binding (GO:0019900)	1.56	4.46E-10	6.38E-08
protein-macromolecule adaptor activity (GO:0030674)	1.56	1.68E-04	1.22E-02

^a^Fold enrichment

^b^False discovery rate

### Transcription factor binding sites enriched in H3K27ac-marked enhancers in bovine satellite cells

Motif enrichment analyses of 35,373 enhancer regions marked with H3K27ac specifically in DD bovine satellite cells indicated enrichment of binding sites for many transcription factors (Additional File 11). The top 30 motifs enriched in these enhancers included binding sites for the bHLH transcription factors MYF5, MYOG, TFAP4 (also known as AP4), MYOD1, TCF12, TCF21, ATOH1, and ASCL2, the basic leucine zipper (bZIP) transcription factors JUN, FOS, FOSB, FOSL2 (also known as FRA2), FOSL1 (also known as FRA1), BATF, JUNB, BACH2, and ATF3, the Krüppel-like family (KLF) transcription factors KLF1, KLF5, and KLF14 (Table 6). Motif enrichment analyses of 5882 enhancer regions marked with H3K27ac specifically in BD bovine satellite cells and 13,199 enhancers marked with H3K27ac in both BD and DD bovine satellite cells revealed enrichment of different sets of transcription factor binding sites (Additional Files 12 and 13). Top motifs enriched in enhancers marked with H3K27ac specifically in BD bovine satellite cells included binding sites for transcription factors ZIC3, TCP16, ASCL2, TFAP2C (also known as AP-2 gamma), ZIC2, MAX, and MYC (Additional File 12). Top motifs enriched in enhancers marked with H3K27ac in both BD and DD bovine satellite cells included many members of the ETS family transcription factors such as ELK1, ELF1, ELK4, GABPA, ETV4, FLI1, ETV1, and ELF4 (Additional File 13).

**Table 6:**
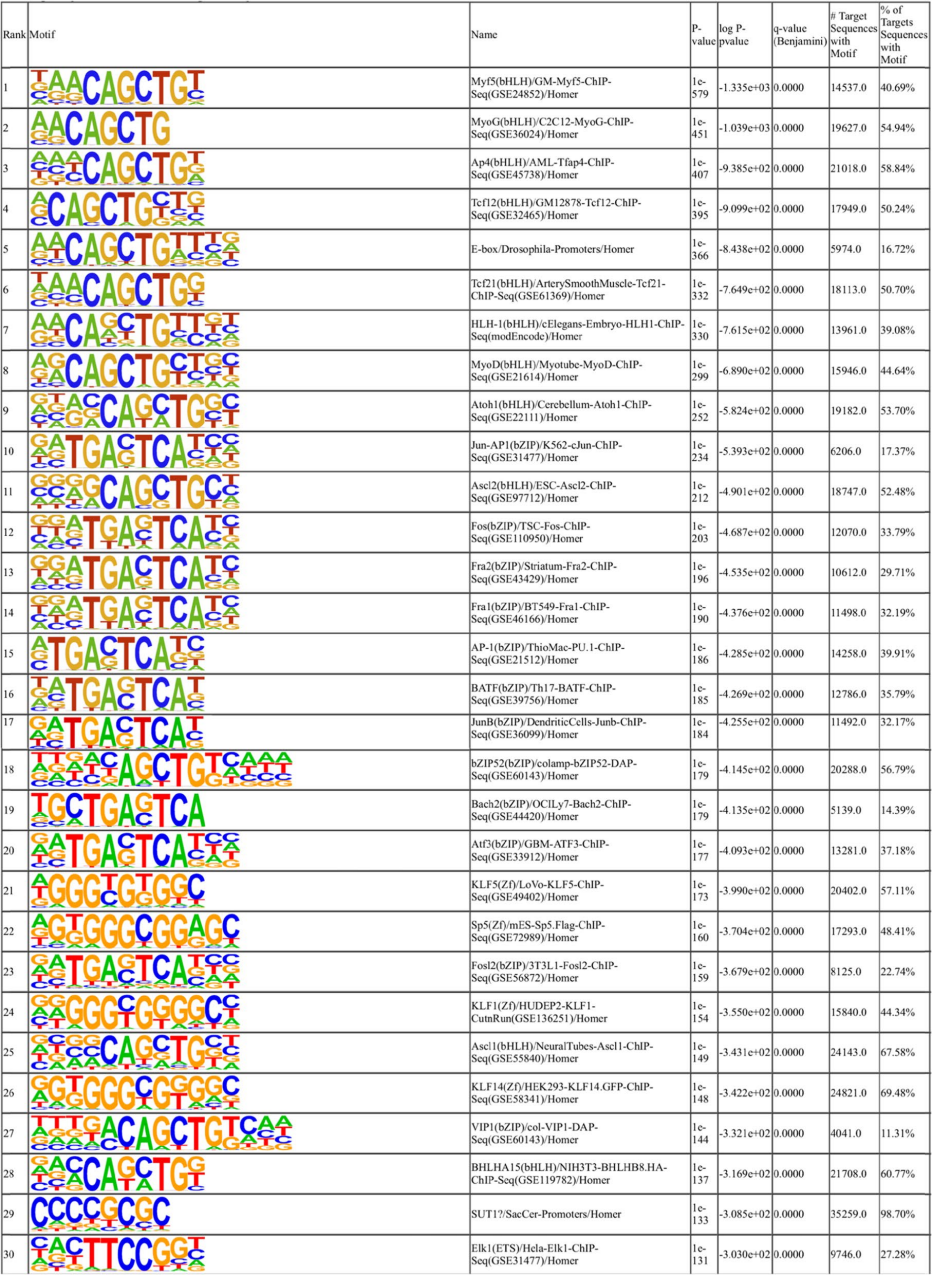
Top 30 motifs enriched in H3K27ac-marked enhancers in during-differentiation bovine satellite cells

### Validation of the role of FOS and FOSB in bovine satellite cell differentiation

**Fig. 5:**
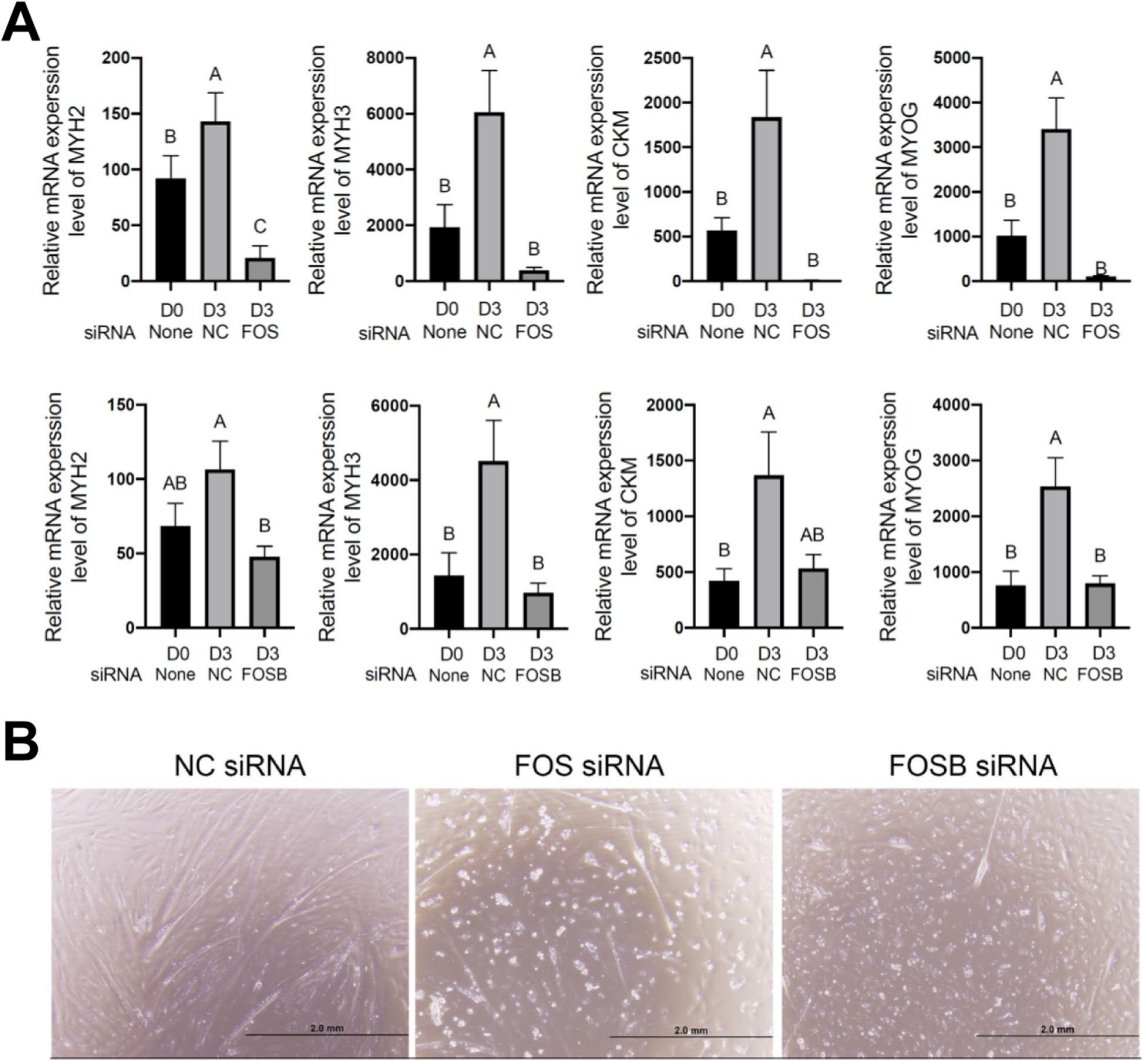
Effect of siRNA-mediated knockdown of FOS or FOSB mRNA on bovine satellite cell differentiation. Bovine satellite cells were transfected with siRNA targeting FOS or FOSB mRNA or negative control (NC) siRNA and then induced to differentiate and fuse into myotubes for 3 days. **A** Expression levels of 4 marker genes of differentiated myoblasts. D0 and D3 mean the day before and day 3 after induction of differentiation, respectively. Bars not sharing the same letter label are different (*P* < 0.05, *n* = 6). **B** Representative images of bovine satellite cells transfected with FOS, FOSB, or negative control siRNA on day 3 of differentiation

The motif enrichment analyses of H3K27ac-marked enhancers in BD and DD bovine satellite cells indicated that gene transcription during bovine satellite cell differentiation is controlled by transcription factors besides the widely known four MRFs. These additional transcription factors include the AP-1 family of transcription factors. We tested the role of the AP-1 transcription factor family members FOS and FOSB in bovine satellite cell differentiation. In this experiment, we transfected bovine satellite cells with siRNA targeting bovine FOS or FOSB mRNA, induced the satellite cells to differentiate and fuse into myotubes, and measured the myogenic differentiation extent of the cells by quantifying the expression of markers of differentiated myoblasts, including MYH2, MYH3, MYOG, and CKM (creatine kinase, M-type). As shown in Fig. 5A and B, in bovine satellite cells transfected with the negative control siRNA, the mRNA expression levels of these markers were significantly increased on day 3 of differentiation compared to their expression levels on the day before induction of differentiation. However, the mRNA expression levels of these markers in bovine satellite cells transfected with siRNA targeting FOS or FOSB mRNA were markedly lower than in cells transfected with the negative control siRNA. Morphologically, satellite cells transfected with siRNA targeting FOS or FOSB mRNA formed fewer and smaller myotubes than those transfected with the negative control siRNA (Fig. 5C). These data support a positive role for FOS and FOSB in bovine satellite cell differentiation and fusion into myotubes.

## Discussion

Histone modification affects gene transcription by altering the accessibility of chromatin and the recruitment of transcription factors and cofactors to chromatin [20]. Large-scale mapping of histone modification has revealed that different types of enhancers are associated with different histone modifications. Active enhancers are associated with H3K27ac and histone 3 lysine 4 monomethylation (H3K4me1) modifications; primed or poised enhancers are marked with H3K4me1 but not H3K27ac; and silenced or repressed enhancers are often associated with H3K27me3 modification [21–24]. Based on these associations, ChIP-seq has been widely used to identify enhancers and other types of regulatory DNA regions in whole genomes [25–31]. In this study, we have identified 19,027 and 47,669 H3K27ac-marked enhancers in bovine satellite cells before and during differentiation, respectively. Identification of these enhances provides a valuable resource for understanding the mechanism that regulates gene expression during satellite cell differentiation, a key step in skeletal muscle development and growth.

Compared to the consistent identification of 47,669 H3K27ac-marked active enhancers from two biological replicates of during-differentiation bovine satellite cells, only 19,027 H3K27ac-marked active enhancers were repeatedly identified from two samples of before-differentiation bovine satellite cells. This difference suggests much more active transcription factor binding to the genome, much more active recruitment of histone acetylases, and hence much more active H3K27ac modification in bovine satellite cells during differentiation than before differentiation. There is a possibility that this difference was caused by biological variation, as indicated by the large difference in the numbers of H3K27-marked enhancers identified from two samples of before-differentiation satellite cells. In our research on satellite cells, we have noticed satellite cells from different animals differ in differentiation potential in culture, and this difference suggests animal-to-animal variation in gene expression and histone modification in satellite cells.

Enhancers can be located upstream or downstream of TSS, in introns or exons, and near or distantly from the promoters [32–34]. Some enhancers can be located in intergenic regions several hundred kilobases away from TSS and control gene transcription by forming DNA loops with the promoters [35–37]. Genomic distribution analyses showed that H3K27ac-marked enhancers in bovine satellite cells have similar genomic distribution to enhancers in other types of cells or species [32–34]. However, the genomic location of H3K27ac-marked enhancers in bovine satellite cells varies with the differentiation stage of these cells. Whereas most of the differentiation stage-dependent H3K27ac-marked enhancers in bovine satellite cells were located in the distal intergenic regions, most of the differentiation stage-independent H3K27ac-marked enhancers in bovine satellite cells were located in the promoter regions. This study also showed that H3K27ac-marked enhancers specific to during-differentiation bovine satellite cells were associated with genes involved in muscle organization, adaptation, and development while H3K27ac-marked enhancers common to both before- and during-differentiation satellite cells were associated with genes involved in basic cellular functions and processes. These results suggest that distal enhancers are preferentially activated to increase the expression of genes determining the differentiation stage of satellite cells, whereas proximal enhancers or promoters are preferentially activated to increase the expression of genes maintaining the basic cellular function of satellite cells. This differentiation stage-dependent activation of distal and proximal enhancers in bovine satellite cells is apparently consistent with earlier findings that distal enhancers mediate the expression of cell type- or developmental stage-specific genes while core promoters and proximal enhancers are responsible for the expression of housekeeping genes [38, 39].

This study showed that genes associated with H3K27ac modification were expressed at greater levels than those without H3K27ac modification in bovine satellite cells, regardless of the differentiation stage of the cells. This result supports H3K27ac as a histone marker for transcriptional activation [16]. This study also showed that many genes were associated with multiple H3K27ac-marked enhancers, but that the numbers of H3K27ac-marked enhancers were not correlated with the expression levels of associated genes in bovine satellite cells. These results suggest that multiple H3K27ac-marked enhancers do not function in an additive manner to increase gene expression or that multiple H3K27ac-marked enhancers are functionally redundant in bovine satellite cells. Indeed, recent studies using the CRISPR-Cas9 approach demonstrate that not every enhancer is functionally important and that most enhancers provide only a supportive or backup role in regulating gene expression [40–42].

H3K27ac modification at enhancers results from transcription factor binding and subsequent recruitment of histone acetyltransferases such as p300 and CBP [43, 44]. In this study we have identified many transcription factors that may bind to H3K27ac-marked enhancers in bovine satellite cells before or during differentiation. Among the transcription factors that are predicted to bind to H3K27ac-marked enhancers in during-differentiation bovine satellite cells are MYOG and MYOD1, the MEF2 family transcription factors, the KLF family transcription factors, and the TEAD family transcription factors. Both MYOG and MYOD1 are known as the central transcriptional regulators of myoblast differentiation, and they regulate the expression of muscle-specific genes by binding to the motif called E-box [45]. The MEF2 family transcription factors MEF2A, MEF2C and MEF2D [45, 46], the KLF family transcription factors KLF3 and KLF5 [47, 48], and the TEAD family transcription factors TEAD2 and TEAD4 [49–51] have also been shown to play a positive role in myoblast differentiation. Identification of the binding sites for MYOG, MYOD1, MEF2, KLF, and TEAD transcription factors among the top motifs enriched in H3K27ac-marked enhancers in during-differentiation bovine satellite cells validates the quality of active enhancers identified in this study.

This study shows that many other transcription factors regulate gene expression during satellite cell differentiation, and these other transcription factors include the AP-1 family of transcription factors (e.g., FOS). Enrichment of binding sites for the AP-1 family of transcription factors in active enhancers in during-differentiation bovine satellite cells is intriguing because the member of the AP-1 family of transcription factors JUN is known to antagonize the stimulatory effect of MYOD1 on myoblast differentiation [52]. However, overexpression of JUNB, a member of the AP-1 family of transcription factors closely related to JUN, increased hypertrophy and expression of the muscle-specific gene MYH4 in C2C12 myoblasts [53]. We have also validated a positive role of two members of the AP-1 family of transcription factors, namely FOS and FOSB, in driving bovine satellite cell differentiation in this study. Therefore, different members of the AP-1 family of transcription factors might have different effects on gene expression during myoblast or satellite cell differentiation.

## Conclusions

In summary, we have identified tens of thousands of genomic regions associated with H3K27ac modification, i.e., active enhancers, in before- or during-differentiation bovine satellite cells. These enhancer regions contain binding sites for many transcription factors, including MYOG and MYOD1, whose role in myoblast or satellite cell differentiation is widely known, and the AP-1 transcription factors, AP-4, and many others, whose roles in myoblast or satellite cell differentiation are less known or unknown. These enhancers and transcription factors should be valuable for elucidating the mechanisms that mediate gene transcription during myoblast or satellite cell differentiation. Because myoblast or satellite cell differentiation is a key step of skeletal muscle development and growth, the enhancers, the transcription factors, and the genes targeted by these enhancers and transcription factors should be also valuable for identifying and interpreting skeletal muscle trait-associated DNA sequences and variants in cattle, which are agriculturally important animals.

## Methods

### Isolation and culture of bovine satellite cells

Skeletal muscle was collected from Angus-crossbred steers slaughtered at the Virginia Tech Meat Center. Satellite cells was isolated through pronase digestion and differential centrifugation as described before [54, 55]. Satellite cells were cultured in growth medium for about a week before being induced to differentiate and fuse into myotubes. Differentiation of bovine satellite cells into myotubes was induced by replacing growth medium with differentiation medium. Growth medium consisted of Dulbecco’s Modified Eagle Medium (DMEM), 10% fetal bovine serum (FBS) (R&D Systems, Minneapolis, MN, USA), 2 mM L-glutamine, and 1% Antibiotic-Antimycotic (100×) (ABAM). Differentiation medium consisted of DMEM, 2% horse serum (R&D Systems), 2 mM L-glutamine, and 1% ABAM. All cell culture was performed at 37 °C in a humidified, 5% CO_2_ atmosphere. All cell culture reagents were purchased from ThermoFisher Scientific (Waltham, MA, USA) unless otherwise indicated.

### ChIP assay

Satellite cells from two steers immediately before and 2 days after induction of differentiation were cross-linked in 1% formaldehyde for 10 min and then lysed in lysis buffer from the ChIP-IT kit (Active Motif, Carlsbad, CA, USA). Cell nuclei were suspended in ChIP buffer from the ChIP-IT kit and then sheared on ice by 10 pulses of 20-s sonication using a sonic dismembrator Model 100 at setting 3 (ThermoFisher Scientific) to generate chromatin fragments of 200 to 1000 bp. To identify the genomic regions associated with H3K27ac modification, chromatin fragments were incubated with an anti-histone H3K27ac antibody (ab4729, abcam, Cambridge, MA, USA) at 4 °C overnight with gentle rocking. The H3K27ac antibody-chromatin complexes were separated from unbound chromatin fragments using protein G-Dynal beads (ThermoFisher Scientific). Chromatin fragments immunoprecipitated by the H3K27ac antibody and those before immunoprecipitation (i.e., input chromatin) were reverse cross-linked by incubating them at 65 °C for 4 h. DNA was extracted and purified using spin columns from the ChIP-IT kit.

### ChIP-seq library construction and sequencing

ChIP-seq libraries were prepared using the NEBNext ChIP-Seq Library Prep Reagent Set for Illumina (New England BioLabs, Ipswich, MA, USA), according to the supplier’s instructions. Briefly, ChIP DNA was end repaired using T4 DNA polymerase, Klenow DNA polymerase, and T4 polynucleotide kinase. End-repaired DNA was then added with 3′ dA overhangs using exonuclease minus Klenow DNA polymerase and dATP. The dA-tailed DNA fragments were ligated to the sequencing adaptor. DNA fragments of approximately 300 bp were selected from the adaptor-ligated DNA using AMPure XP Beads. The size-selected DNA fragments were amplified in 12 cycles of PCR using an index primer and a universal PCR primer. Two ChIP-seq libraries were prepared from DNA immunoprecipitated from before- or during-differentiation bovine satellite cells originally isolated from two different cattle. Two Input-seq libraries were prepared from input DNA pooled equally from before- and during-differentiation bovine satellite cells. Two biological replicates were used for ChIP-seq according to the ChIP-seq guidelines and practices proposed by the ENCODE and modENCODE consortia [18]. Each library was assessed for quality on a Bioanalyzer before being single-end sequenced on an Illumina Hiseq 2500 at the Genomics Sequencing Center at Virginia Tech.

### ChIP-seq data analyses

Sequences from ChIP-seq libraries were first trimmed to remove the adapters using Trimmomatic [56]. The trimmed reads were then mapped to the bovine genome assembly (ARS-UCD 1.2 BosTau 9) using Hisat2 (2.2.0) [57]. The aligned reads were sorted and merged using SAMtools (1.9) [58]. Peak calling of the aligned reads was made using MACS3 (3.0.0a5) [59], where a H3K27ac-ChIP-seq library was compared to an Input-seq library (i.e., control) made from the same satellite cells, and where the q-value threshold was set as 0.05. Quality of peak enrichment in ChIP-seq reads was assessed by Phantompeakqualtools [60]. ChIP-seq peaks were visualized in the IGV browser (2.8.2) [61]. ChIP-seq peaks were annotated using ChIPseeker (1.22.1) [62]. Motif enrichment analyses were performed using HOMER (4.11.1) [63]. Gene ontology (GO) enrichment analysis was performed using the PANTHER Classification System [64–66].

### Small interfering RNA (siRNA)-mediated gene knockdown

Bovine satellite cells in 12-well plates were transfected with 30 nM of siRNA targeting bovine FOS or FOSB mRNA using 6 μL of Lipofectamine RNAiMAX Reagent according to the supplier’s instructions (ThermoFisher Scientific). A universal negative control siRNA (MISSION siRNA Universal Negative Control #1, Millipore Sigma, Burlington, MA, USA) was transfected as a negative control. The sense and antisense siRNA sequences targeting bovine FOS and FOSB mRNAs were CAGAAGAGAUGUCUGUGGCUUCUCU and AGAGAAGCCACAGACAUCUCUUCUG, and GACAUGCCAGGAACCAGUUACUCCA and UGGAGUAACUGGUUCCUGGCAUGUC, respectively. These siRNAs were confirmed to have at least 70% knockdown efficiency in pilot experiments. Following transfection, bovine satellite cells were cultured in differentiation medium for 48 h as descried above. The differentiation degree of satellite cells was assessed by quantifying mRNA expression of markers of differentiated myoblasts, including CKM, MYH2, MYH3, and MYOG, as previously described [19].

### RNA extraction and reverse transcription-quantitative PCR (RT-qPCR)

Total RNA was extracted from bovine satellite cells using the Direct-zol RNA Miniprep Kit (Zymo Research, Irvine, CA, USA). Reverse transcription of total RNA into cDNA was performed using ImProm-II reverse transcriptase and random primers according to the manufacture’s instruction (Promega, Madison, WI, USA). Quantitative PCR was performed using the SYBR Green chemistry as described previously [19]. The relative abundance of target mRNAs was calculated using the 2^-ΔΔCt^ method [67]. The Ct values for target mRNAs were normalized to the Ct values for HMBS, which was chosen as a reference gene because it was stably expressed in different conditions [68].

### Statistical analysis

Gene expression data were analyzed by ANOVA. Two means were compared by t-test, and multiple means were compared by the Tukey test. All data are expressed as mean ± standard error.

## Supplementary Information

**Additional file 1.** Assessment of ChIP-seq enrichment by Phantompeakqualtools

**Additional file 2.** H3K27ac peaks specific to before-differentiation bovine satellite cells

**Additional file 3.** H3K27ac peaks specific to during-differentiation bovine satellite cells

**Additional file 4.** H3K27ac peaks common to both before- and during-differentiation bovine satellite cells

**Additional file 5.** Top 10 GO biological processes enriched in genes associated with H3K27ac modification in before-differentiation bovine satellite cells

**Additional file 6.** Top 10 GO cellular components enriched in genes associated with H3K27ac modification in before-differentiation bovine satellite cells

**Additional file 7.** Top 10 GO molecular functions enriched in genes associated with H3K27ac modification in before-differentiation bovine satellite cells

**Additional file 8.** Top 10 GO biological processes enriched in genes associated with H3K27ac modification in both before- and during-differentiation bovine satellite cells

**Additional file 9.** Top 10 GO cellular components enriched in genes associated with H3K27ac modification in both before- and during-differentiation bovine satellite cells

**Additional file 10.** Top 10 GO molecular functions enriched in genes associated with H3K27ac modification in both before- and during-differentiation bovine satellite cells

**Additional file 11.** Motifs enriched in enhancers marked with H3K27ac in during-differentiation bovine satellite cells

**Additional file 12.** Motifs enriched in enhancers marked with H3K27ac in before-differentiation bovine satellite cells

**Additional file 13.** Motifs enriched in enhancers marked with H3K27ac in both before- and during-differentiation bovine satellite cells

## Data Availability

The datasets generated and/or analyzed during this study are available as additional files 1–14. The sequencing data from this study has been deposited in the NCBI GEO database (https://www.ncbi.nlm.nih.gov/geo/) under accession number GSE179821.

## Abbreviations

ABAM: Antibiotic-antimycotic
BD: Before differentiation
ChIP: Chromatin immunoprecipitation assay
ChIP-seq: Chromatin immunoprecipitation coupled with sequencing
DD: During differentiation
DMEM: Dulbecco’s Modified Eagle Medium
FBS: Fetal bovine serum
GO: Gene ontology
H3K27ac: Histone 3 lysine 27 acetylation
H3K4me1: Histone 3 lysine 4 monomethylation
H3K4me3: Histone 3 lysine 4 trimethylation
MRF: Myogenic regulatory factor
RT-qPCR: Reverse transcription-quantitative PCR
