# Supplementary figures and images for: Genome-wide identification of enhancers and transcription factors regulating the myogenic differentiation of bovine satellite cells
Source: BMC Genomics. 2021 Dec 16;22:901. doi: 10.1186/s12864-021-08224-7 (PMC8675486; doi:10.1186/s12864-021-08224-7)

## Assessment of ChIP-seq enrichment by Phantompeakqualtools

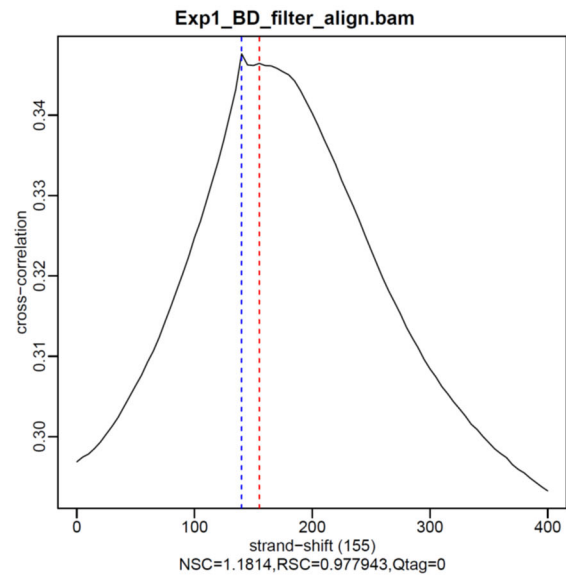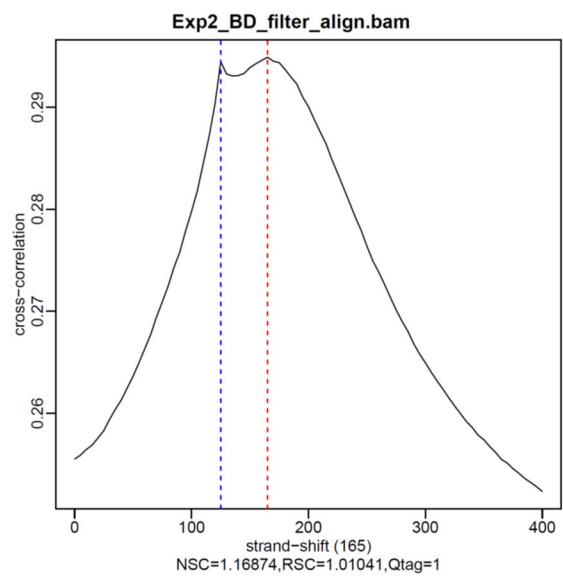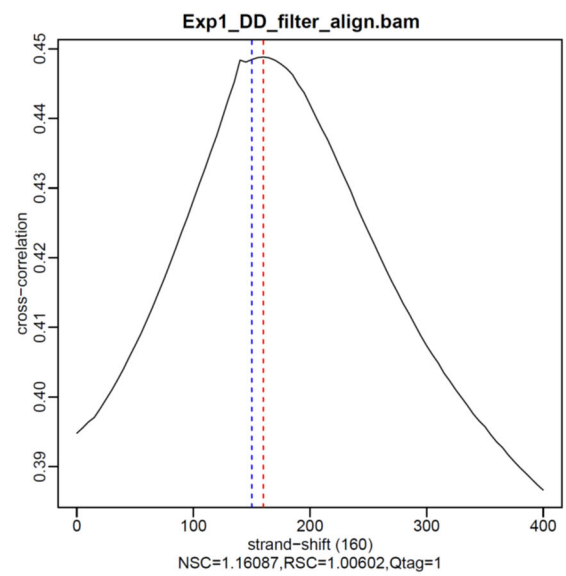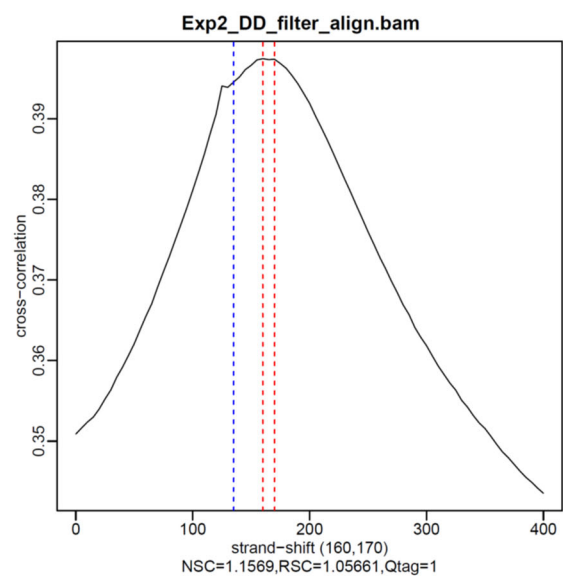

Supplement: Supplementary file 1 — Additional file 1. Assessment of ChIP-seq enrichment by Phantompeakqualtools [file 12864_2021_8224_MOESM1_ESM.pdf]
