# Supplementary material for: Genome-wide identification of enhancers and transcription factors regulating the myogenic differentiation of bovine satellite cells
Source: BMC Genomics. 2021 Dec 16;22:901. doi: 10.1186/s12864-021-08224-7 (PMC8675486; doi:10.1186/s12864-021-08224-7)
Supplement: Supplementary file 5 — Additional file 5. Top 10 GO biological processes enriched in genes associated with H3K27ac modification in before-differentiation bovine satellite cells [file 12864_2021_8224_MOESM5_ESM.docx]

**Top 10 GO biological processes enriched in genes associated with H3K27ac modification in before-differentiation bovine satellite cells**

| GO biological process | FE^1^ | P-value | FDR^2^ |
| --- | --- | --- | --- |
| pentose metabolic process (GO:0019321) | 4.75 | 4.40E-04 | 1.88E-02 |
| negative regulation of fibroblast growth factor receptor signaling pathway (GO:0040037) | 4.19 | 9.01E-04 | 3.30E-02 |
| response to lipoprotein particle (GO:0055094) | 3.92 | 7.65E-04 | 2.95E-02 |
| cellular response to lipoprotein particle stimulus (GO:0071402) | 3.74 | 1.04E-03 | 3.73E-02 |
| regulation of fibroblast growth factor receptor signaling pathway (GO:0040036) | 3.71 | 3.91E-04 | 1.71E-02 |
| positive regulation of neural precursor cell proliferation (GO:2000179) | 3.48 | 1.49E-05 | 1.04E-03 |
| negative regulation of Notch signaling pathway (GO:0045746) | 3.43 | 6.86E-04 | 2.72E-02 |
| prostaglandin biosynthetic process (GO:0001516) | 3.42 | 1.12E-03 | 3.93E-02 |
| prostanoid biosynthetic process (GO:0046457) | 3.42 | 1.12E-03 | 3.93E-02 |
| negative regulation of blood coagulation (GO:0030195) | 3.08 | 4.43E-04 | 1.89E-02 |

^1^Fold enrichment; ^2^False discovery rate
