# Supplementary material for: Genome-wide identification of enhancers and transcription factors regulating the myogenic differentiation of bovine satellite cells
Source: BMC Genomics. 2021 Dec 16;22:901. doi: 10.1186/s12864-021-08224-7 (PMC8675486; doi:10.1186/s12864-021-08224-7)
Supplement: Supplementary file 6 — Additional file 6. Top 10 GO cellular components enriched in genes associated with H3K27ac modification in before-differentiation bovine satellite cells [file 12864_2021_8224_MOESM6_ESM.docx]

**Top 10 GO cellular components enriched in genes associated with H3K27ac modification in before-differentiation bovine satellite cells**

| GO cellular component | FE^1^ | P-value | FDR^2^ |
| --- | --- | --- | --- |
| Schaffer collateral - CA1 synapse (GO:0098685) | 2.50 | 1.28E-03 | 3.82E-02 |
| cytoplasmic side of plasma membrane (GO:0009898) | 2.04 | 1.88E-04 | 7.76E-03 |
| cytoplasmic side of membrane (GO:0098562) | 1.93 | 4.21E-04 | 1.56E-02 |
| receptor complex (GO:0043235) | 1.89 | 8.39E-07 | 7.25E-05 |
| postsynaptic membrane (GO:0045211) | 1.85 | 2.82E-04 | 1.14E-02 |
| glutamatergic synapse (GO:0098978) | 1.84 | 1.14E-04 | 4.93E-03 |
| cell-substrate junction (GO:0030055) | 1.84 | 9.65E-04 | 3.31E-02 |
| synaptic membrane (GO:0097060) | 1.83 | 3.25E-05 | 1.59E-03 |
| focal adhesion (GO:0005925) | 1.83 | 1.55E-03 | 4.46E-02 |
| postsynapse (GO:0098794) | 1.73 | 2.63E-06 | 2.07E-04 |

^1^Fold enrichment; ^2^False discovery rate
