# Supplementary material for: Genome-wide identification of enhancers and transcription factors regulating the myogenic differentiation of bovine satellite cells
Source: BMC Genomics. 2021 Dec 16;22:901. doi: 10.1186/s12864-021-08224-7 (PMC8675486; doi:10.1186/s12864-021-08224-7)
Supplement: Supplementary file 7 — Additional file 7. Top 10 GO molecular functions enriched in genes associated with H3K27ac modification in before-differentiation bovine satellite cells [file 12864_2021_8224_MOESM7_ESM.docx]

**Top 10 GO molecular functions enriched in genes associated with H3K27ac modification in before-differentiation bovine satellite cells**

| GO molecular function | FE^1^ | P-value | FDR^2^ |
| --- | --- | --- | --- |
| transmembrane receptor protein tyrosine kinase activity (GO:0004714) | 2.50 | 2.15E-05 | 2.80E-03 |
| protein tyrosine kinase activity (GO:0004713) | 2.40 | 1.51E-05 | 2.16E-03 |
| growth factor binding (GO:0019838) | 2.25 | 1.10E-04 | 1.28E-02 |
| transmembrane receptor protein kinase activity (GO:0019199) | 2.23 | 8.61E-05 | 1.03E-02 |
| sulfur compound binding (GO:1901681) | 2.02 | 1.22E-05 | 1.80E-03 |
| oxidoreductase activity, acting on CH-OH group of donors (GO:0016614) | 1.94 | 3.65E-04 | 3.44E-02 |
| phosphatase activity (GO:0016791) | 1.65 | 4.97E-04 | 4.41E-02 |
| phosphoric ester hydrolase activity (GO:0042578) | 1.64 | 8.29E-05 | 1.02E-02 |
| tubulin binding (GO:0015631) | 1.60 | 2.64E-04 | 2.61E-02 |
| phosphotransferase activity, alcohol group as acceptor (GO:0016773) | 1.50 | 2.11E-05 | 2.83E-03 |

^1^Fold enrichment; ^2^False discovery rate
