# Supplementary material for: Genome-wide identification of enhancers and transcription factors regulating the myogenic differentiation of bovine satellite cells
Source: BMC Genomics. 2021 Dec 16;22:901. doi: 10.1186/s12864-021-08224-7 (PMC8675486; doi:10.1186/s12864-021-08224-7)
Supplement: Supplementary file 8 — Additional file 8. Top 10 GO biological processes enriched in genes associated with H3K27ac modification in both before- and during-differentiation bovine satellite cells [file 12864_2021_8224_MOESM8_ESM.docx]

**Top 10 GO biological processes enriched in genes associated with H3K27ac modification in both before- and during-differentiation bovine satellite cells**

| GO biological process | FE^1^ | P-value | FDR^2^ |
| --- | --- | --- | --- |
| viral gene expression (GO:0019080) | 3.03 | 2.33E-03 | 4.72E-02 |
| stress granule assembly (GO:0034063) | 2.92 | 2.31E-03 | 4.68E-02 |
| regulation of fibroblast migration (GO:0010762) | 2.75 | 1.06E-03 | 2.39E-02 |
| protein K11-linked ubiquitination (GO:0070979) | 2.72 | 6.39E-04 | 1.54E-02 |
| regulation of intrinsic apoptotic signaling pathway in response to DNA damage (GO:1902229) | 2.54 | 2.40E-03 | 4.82E-02 |
| positive regulation of response to endoplasmic reticulum stress (GO:1905898) | 2.54 | 2.40E-03 | 4.81E-02 |
| regulation of autophagosome assembly (GO:2000785) | 2.53 | 8.07E-04 | 1.89E-02 |
| positive regulation of RNA splicing (GO:0033120) | 2.52 | 1.38E-03 | 2.97E-02 |
| retrograde vesicle-mediated transport, Golgi to endoplasmic reticulum (GO:0006890) | 2.40 | 3.73E-04 | 9.58E-03 |
| protein targeting to ER (GO:0045047) | 2.39 | 1.97E-03 | 4.08E-02 |

^1^Fold enrichment; ^2^False discovery rate
