# Supplementary material for: Genome-wide identification of enhancers and transcription factors regulating the myogenic differentiation of bovine satellite cells
Source: BMC Genomics. 2021 Dec 16;22:901. doi: 10.1186/s12864-021-08224-7 (PMC8675486; doi:10.1186/s12864-021-08224-7)
Supplement: Supplementary file 9 — Additional file 9. Top 10 GO cellular components enriched in genes associated with H3K27ac modification in both before- and during-differentiation bovine satellite cells [file 12864_2021_8224_MOESM9_ESM.docx]

**Top 10 GO cellular components enriched in genes associated with H3K27ac modification in both before- and during-differentiation bovine satellite cells**

| GO cellular component | FE^1^ | P-value | FDR^2^ |
| --- | --- | --- | --- |
| proteasome regulatory particle (GO:0005838) | 2.84 | 1.22E-03 | 1.30E-02 |
| translation preinitiation complex (GO:0070993) | 2.83 | 4.75E-03 | 3.97E-02 |
| proteasome accessory complex (GO:0022624) | 2.77 | 7.18E-04 | 8.00E-03 |
| chaperone complex (GO:0101031) | 2.72 | 1.55E-03 | 1.59E-02 |
| ER to Golgi transport vesicle membrane (GO:0012507) | 2.45 | 2.90E-03 | 2.70E-02 |
| proteasome complex (GO:0000502) | 2.40 | 2.09E-05 | 3.61E-04 |
| vesicle coat (GO:0030120) | 2.39 | 6.03E-05 | 9.87E-04 |
| endopeptidase complex (GO:1905369) | 2.39 | 6.96E-06 | 1.30E-04 |
| clathrin vesicle coat (GO:0030125) | 2.38 | 6.10E-03 | 4.94E-02 |
| endoribonuclease complex (GO:1902555) | 2.33 | 4.10E-03 | 3.56E-02 |

^1^Fold enrichment; ^2^False discovery rate
