# Supplementary material for: Genome-wide identification of enhancers and transcription factors regulating the myogenic differentiation of bovine satellite cells
Source: BMC Genomics. 2021 Dec 16;22:901. doi: 10.1186/s12864-021-08224-7 (PMC8675486; doi:10.1186/s12864-021-08224-7)
Supplement: Supplementary file 10 — Additional file 10. Top 10 GO molecular functions enriched in genes associated with H3K27ac modification in both before- and during-differentiation bovine satellite cells [file 12864_2021_8224_MOESM10_ESM.docx]

**Top 10 GO molecular functions enriched in genes associated with H3K27ac modification in both before- and during-differentiation bovine satellite cells**

| GO molecular function | FE^1^ | P-value | FDR^2^ |
| --- | --- | --- | --- |
| ubiquitin conjugating enzyme activity (GO:0061631) | 2.73 | 7.08E-05 | 3.34E-03 |
| ubiquitin-like protein conjugating enzyme activity (GO:0061650) | 2.64 | 4.88E-05 | 2.38E-03 |
| protein folding chaperone (GO:0044183) | 2.63 | 7.62E-04 | 2.41E-02 |
| core promoter sequence-specific DNA binding (GO:0001046) | 2.45 | 9.44E-04 | 2.85E-02 |
| promoter-specific chromatin binding (GO:1990841) | 2.37 | 4.61E-04 | 1.63E-02 |
| SNAP receptor activity (GO:0005484) | 2.29 | 1.56E-03 | 4.24E-02 |
| polyubiquitin modification-dependent protein binding (GO:0031593) | 2.27 | 2.47E-04 | 9.79E-03 |
| snRNA binding (GO:0017069) | 2.26 | 1.92E-03 | 4.89E-02 |
| transcription coregulator binding (GO:0001221) | 2.24 | 7.14E-04 | 2.36E-02 |
| SMAD binding (GO:0046332) | 2.24 | 3.58E-05 | 1.83E-03 |

^1^Fold enrichment; ^2^False discovery rate
