# Supplementary material for: Genome-wide identification of enhancers and transcription factors regulating the myogenic differentiation of bovine satellite cells
Source: BMC Genomics. 2021 Dec 16;22:901. doi: 10.1186/s12864-021-08224-7 (PMC8675486; doi:10.1186/s12864-021-08224-7)
Supplement: Supplementary file 11 — Additional file 11. Motifs enriched in enhancers marked with H3K27ac in during-differentiation bovine satellite cells [file 12864_2021_8224_MOESM11_ESM.pdf]

Motifs enriched in enhancers marked with H3K27ac in during-differentiation bovine satellite cells

Rank	Motif	Name	P-value	log P-pvalue	q-value (Benjamini)	# Target Sequences with Motif	% of Targets Sequences with Motif	# Background Sequences with Motif	% of Background Sequences with Motif	Motif File	SVG
1	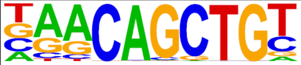	Myf5(bHLH)/GM-Myf5-ChIP-Seq(GSE24852)/Homer	1e-579	-1.335e+03	0.0000	14537.0	40.69%	9801.7	27.98%	motif file (matrix)	svg
2	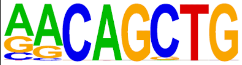	MyoG(bHLH)/C2C12-MyoG-ChIP-Seq(GSE36024)/Homer	1e-451	-1.039e+03	0.0000	19627.0	54.94%	15046.5	42.95%	motif file (matrix)	svg
3	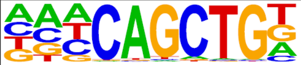	Ap4(bHLH)/AML-Tfap4-ChIP-Seq(GSE45738)/Homer	1e-407	-9.385e+02	0.0000	21018.0	58.84%	16612.1	47.42%	motif file (matrix)	svg
4	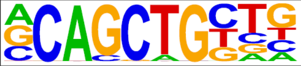	Tcf12(bHLH)/GM12878-Tcf12-ChIP-Seq(GSE32465)/Homer	1e-395	-9.099e+02	0.0000	17949.0	50.24%	13704.2	39.12%	motif file (matrix)	svg
5	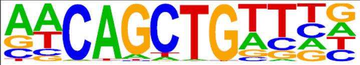	E-box/Drosophila-Promoters/Homer	1e-366	-8.438e+02	0.0000	5974.0	16.72%	3405.8	9.72%	motif file (matrix)	svg
6	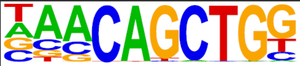	Tcf21(bHLH)/ArterySmoothMuscle-Tcf21-ChIP-Seq(GSE61369)/Homer	1e-332	-7.649e+02	0.0000	18113.0	50.70%	14179.3	40.48%	motif file (matrix)	svg
7	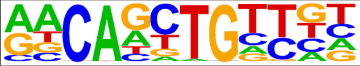	HLH-1(bHLH)/cElegans-Embryo-HLH1-ChIP-Seq(modEncode)/Homer	1e-330	-7.615e+02	0.0000	13961.0	39.08%	10315.0	29.45%	motif file (matrix)	svg
8	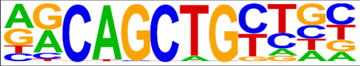	MyoD(bHLH)/Myotube-MyoD-ChIP-Seq(GSE21614)/Homer	1e-299	-6.890e+02	0.0000	15946.0	44.64%	12307.6	35.13%	motif file (matrix)	svg
9	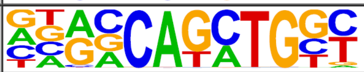	Atoh1(bHLH)/Cerebellum-Atoh1-ChIP-Seq(GSE22111)/Homer	1e-252	-5.824e+02	0.0000	19182.0	53.70%	15666.0	44.72%	motif file (matrix)	svg
10	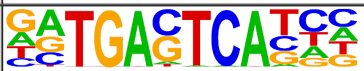	Jun-AP1(bZIP)/K562-cJun-ChIP-Seq(GSE31477)/Homer	1e-234	-5.393e+02	0.0000	6206.0	17.37%	4026.4	11.49%	motif file (matrix)	svg
11	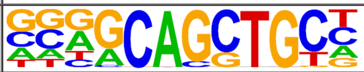	Ascl2(bHLH)/ESC-Ascl2-ChIP-Seq(GSE97712)/Homer	1e-212	-4.901e+02	0.0000	18747.0	52.48%	15502.9	44.26%	motif file (matrix)	svg
12	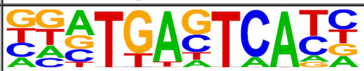	Fos(bZIP)/TSC-Fos-ChIP-Seq(GSE110950)/Homer	1e-203	-4.687e+02	0.0000	12070.0	33.79%	9278.1	26.49%	motif file (matrix)	svg
13	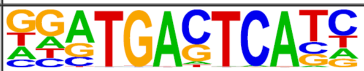	Fra2(bZIP)/Striatum-Fra2-ChIP-Seq(GSE43429)/Homer	1e-196	-4.535e+02	0.0000	10612.0	29.71%	8000.5	22.84%	motif file (matrix)	svg
14	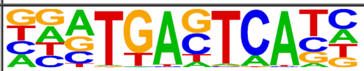	Fra1(bZIP)/BT549-Fra1-ChIP-Seq(GSE46166)/Homer	1e-190	-4.376e+02	0.0000	11498.0	32.19%	8840.3	25.24%	motif file (matrix)	svg
15	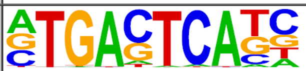	AP-1(bZIP)/ThioMac-PU.1-ChIP-Seq(GSE21512)/Homer	1e-186	-4.285e+02	0.0000	14258.0	39.91%	11408.4	32.57%	motif file (matrix)	svg
16	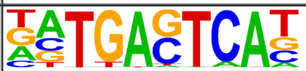	BATF(bZIP)/Th17-BATF-ChIP-Seq(GSE39756)/Homer	1e-185	-4.269e+02	0.0000	12786.0	35.79%	10047.9	28.68%	motif file (matrix)	svg
17		JunB(bZIP)/DendriticCells-Junb-ChIP-Seq(GSE36099)/Homer	1e-184	-4.255e+02	0.0000	11492.0	32.17%	8867.7	25.31%	motif file	svg

	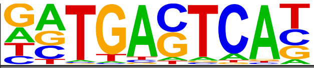									(matrix)	
18	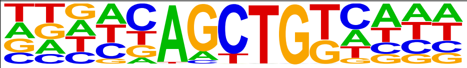	bZIP52(bZIP)/colamp-bZIP52-DAP-Seq(GSE60143)/Homer	1e-179	-4.145e+02	0.0000	20288.0	56.79%	17243.6	49.22%	motif file (matrix)	svg
19	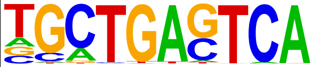	Bach2(bZIP)/OCILy7-Bach2-ChIP-Seq(GSE44420)/Homer	1e-179	-4.135e+02	0.0000	5139.0	14.39%	3373.2	9.63%	motif file (matrix)	svg
20	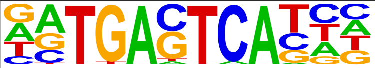	Atf3(bZIP)/GBM-ATF3-ChIP-Seq(GSE33912)/Homer	1e-177	-4.093e+02	0.0000	13281.0	37.18%	10555.6	30.13%	motif file (matrix)	svg
21	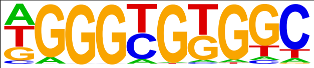	KLF5(Zf)/LoVo-KLF5-ChIP-Seq(GSE49402)/Homer	1e-173	-3.990e+02	0.0000	20402.0	57.11%	17406.4	49.69%	motif file (matrix)	svg
22	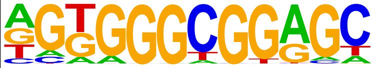	Sp5(Zf)/mES-Sp5.Flag-ChIP-Seq(GSE72989)/Homer	1e-160	-3.704e+02	0.0000	17293.0	48.41%	14471.4	41.31%	motif file (matrix)	svg
23	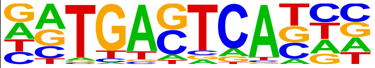	Fosl2(bZIP)/3T3L1-Fosl2-ChIP-Seq(GSE56872)/Homer	1e-159	-3.679e+02	0.0000	8125.0	22.74%	6009.4	17.15%	motif file (matrix)	svg
24	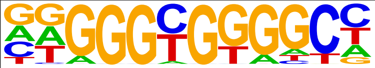	KLF1(Zf)/HUDEP2-KLF1-CutnRun(GSE136251)/Homer	1e-154	-3.550e+02	0.0000	15840.0	44.34%	13131.8	37.49%	motif file (matrix)	svg
25	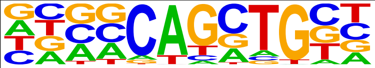	Ascl1(bHLH)/NeuralTubes-Ascl1-ChIP-Seq(GSE55840)/Homer	1e-149	-3.431e+02	0.0000	24143.0	67.58%	21348.2	60.94%	motif file (matrix)	svg
26	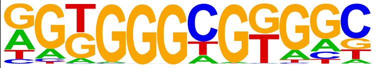	KLF14(Zf)/HEK293-KLF14.GFP-ChIP-Seq(GSE58341)/Homer	1e-148	-3.422e+02	0.0000	24821.0	69.48%	22045.0	62.93%	motif file (matrix)	svg
27	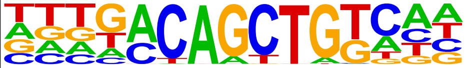	VIP1(bZIP)/col-VIP1-DAP-Seq(GSE60143)/Homer	1e-144	-3.321e+02	0.0000	4041.0	11.31%	2626.5	7.50%	motif file (matrix)	svg
28	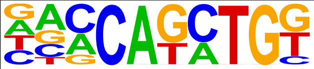	BHLHA15(bHLH)/NIH3T3-BHLHB8.HA-ChIP-Seq(GSE119782)/Homer	1e-137	-3.169e+02	0.0000	21708.0	60.77%	18989.6	54.21%	motif file (matrix)	svg
29	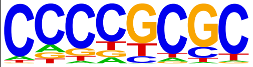	SUT1?/SacCer-Promoters/Homer	1e-133	-3.085e+02	0.0000	35259.0	98.70%	33850.9	96.63%	motif file (matrix)	svg
30	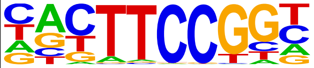	Elk1(ETS)/Hela-Elk1-ChIP-Seq(GSE31477)/Homer	1e-131	-3.030e+02	0.0000	9746.0	27.28%	7634.6	21.79%	motif file (matrix)	svg
31	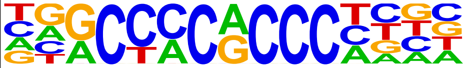	KLF3(Zf)/MEF-Klf3-ChIP-Seq(GSE44748)/Homer	1e-128	-2.958e+02	0.0000	9587.0	26.84%	7512.9	21.45%	motif file (matrix)	svg
32	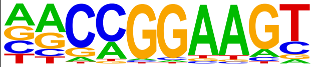	GABPA(ETS)/Jurkat-GABPa-ChIP-Seq(GSE17954)/Homer	1e-125	-2.884e+02	0.0000	14617.0	40.92%	12202.2	34.83%	motif file (matrix)	svg
33	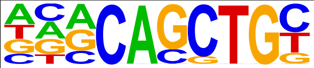	E2A(bHLH)/proBcell-E2A-ChIP-Seq(GSE21978)/Homer	1e-120	-2.775e+02	0.0000	22577.0	63.20%	20009.9	57.12%	motif file (matrix)	svg
34	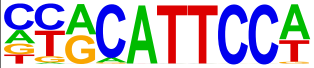	TEAD1(TEAD)/HepG2-TEAD1-ChIP-Seq(Encode)/Homer	1e-118	-2.725e+02	0.0000	15828.0	44.31%	13415.0	38.30%	motif file (matrix)	svg
35	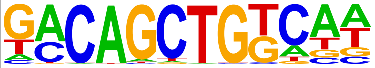	bZIP69(bZIP)/col-bZIP69-DAP-Seq(GSE60143)/Homer	1e-114	-2.628e+02	0.0000	2709.0	7.58%	1682.6	4.80%	motif file (matrix)	svg
36	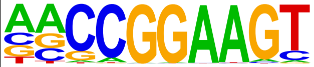	ETS(ETS)/Promoter/Homer	1e-110	-2.556e+02	0.0000	5800.0	16.24%	4268.2	12.18%	motif file (matrix)	svg
37	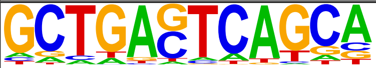	MafK(bZIP)/C2C12-MafK-ChIP-Seq(GSE36030)/Homer	1e-108	-2.503e+02	0.0000	4735.0	13.25%	3368.7	9.62%	motif file (matrix)	svg

38	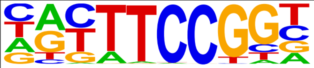	Elk4(ETS)/Hela-Elk4-ChIP-Seq(GSE31477)/Homer	1e-108	-2.500e+02	0.0000	9814.0	27.47%	7866.1	22.46%	motif file (matrix)	svg
39	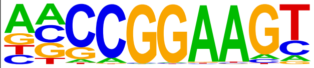	ELF1(ETS)/Jurkat-ELF1-ChIP-Seq(SRA014231)/Homer	1e-108	-2.487e+02	0.0000	9013.0	25.23%	7141.5	20.39%	motif file (matrix)	svg
40	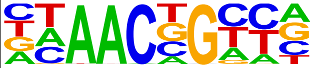	BMYB(HTH)/Hela-BMYB-ChIP-Seq(GSE27030)/Homer	1e-106	-2.457e+02	0.0000	19852.0	55.57%	17431.2	49.76%	motif file (matrix)	svg
41	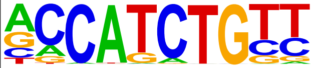	NeuroG2(bHLH)/Fibroblast-NeuroG2-ChIP-Seq(GSE75910)/Homer	1e-101	-2.332e+02	0.0000	22782.0	63.77%	20397.6	58.23%	motif file (matrix)	svg
42	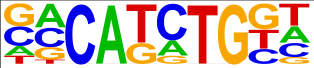	TCF4(bHLH)/SHSY5Y-TCF4-ChIP-Seq(GSE96915)/Homer	1e-100	-2.319e+02	0.0000	22555.0	63.14%	20175.8	57.60%	motif file (matrix)	svg
43	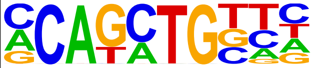	Twist2(bHLH)/Myoblast-Twist2.Ty1-ChIP-Seq(GSE127998)/Homer	1e-99	-2.285e+02	0.0000	24496.0	68.57%	22148.7	63.23%	motif file (matrix)	svg
44	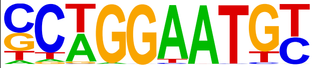	TEAD2(TEA)/Py2T-Tead2-ChIP-Seq(GSE55709)/Homer	1e-98	-2.269e+02	0.0000	8852.0	24.78%	7068.1	20.18%	motif file (matrix)	svg
45	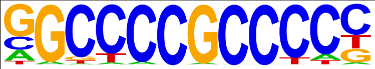	Sp1(Zf)/Promoter/Homer	1e-97	-2.234e+02	0.0000	5341.0	14.95%	3955.8	11.29%	motif file (matrix)	svg
46	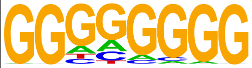	Maz(Zf)/HepG2-Maz-ChIP-Seq(GSE31477)/Homer	1e-96	-2.221e+02	0.0000	21550.0	60.33%	19214.3	54.85%	motif file (matrix)	svg
47	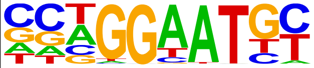	TEAD4(TEA)/Tropoblast-Tead4-ChIP-Seq(GSE37350)/Homer	1e-96	-2.216e+02	0.0000	14435.0	40.41%	12290.6	35.09%	motif file (matrix)	svg
48	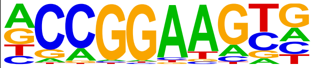	ETV4(ETS)/HepG2-ETV4-ChIP-Seq(ENCODE)/Homer	1e-95	-2.195e+02	0.0000	17713.0	49.58%	15452.7	44.11%	motif file (matrix)	svg
49	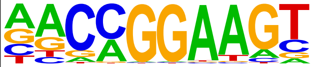	ETV1(ETS)/GIST48-ETV1-ChIP-Seq(GSE22441)/Homer	1e-92	-2.131e+02	0.0000	20277.0	56.76%	17992.3	51.36%	motif file (matrix)	svg
50	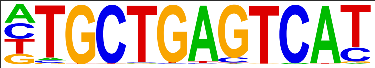	Nrf2(bZIP)/Lymphoblast-Nrf2-ChIP-Seq(GSE37589)/Homer	1e-91	-2.118e+02	0.0000	1380.0	3.86%	746.2	2.13%	motif file (matrix)	svg
51	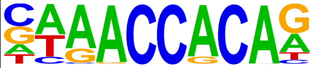	RUNX(Runt)/HPC7-Runx1-ChIP-Seq(GSE22178)/Homer	1e-88	-2.037e+02	0.0000	11813.0	33.07%	9889.2	28.23%	motif file (matrix)	svg
52	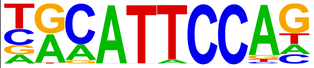	TEAD3(TEA)/HepG2-TEAD3-ChIP-Seq(Encode)/Homer	1e-87	-2.025e+02	0.0000	17311.0	48.46%	15139.0	43.22%	motif file (matrix)	svg
53	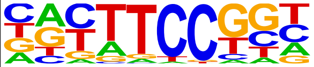	Fli1(ETS)/CD8-FLI-ChIP-Seq(GSE20898)/Homer	1e-86	-1.993e+02	0.0000	18051.0	50.53%	15872.9	45.31%	motif file (matrix)	svg
54	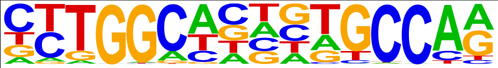	NF1(CTF)/LNCAP-NF1-ChIP-Seq(Unpublished)/Homer	1e-85	-1.975e+02	0.0000	7916.0	22.16%	6321.2	18.05%	motif file (matrix)	svg
55	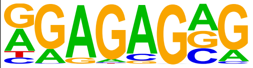	Trl(Zf)/S2-GAGAfactor-ChIP-Seq(GSE40646)/Homer	1e-84	-1.948e+02	0.0000	30151.0	84.40%	28168.2	80.41%	motif file (matrix)	svg
56	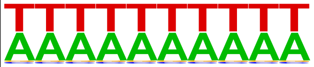	SeqBias: A/T bias	1e-84	-1.948e+02	0.0000	35333.0	98.91%	34141.2	97.46%	motif file (matrix)	svg
57	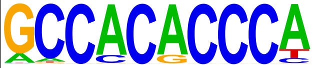	Klf4(Zf)/mES-Klf4-ChIP-Seq(GSE11431)/Homer	1e-84	-1.939e+02	0.0000	7272.0	20.36%	5752.1	16.42%	motif file (matrix)	svg
58		AMYB(HTH)/Testes-AMYB-ChIP-Seq(GSE44588)/Homer	1e-84	-1.935e+02	0.0000	19989.0	55.96%	17799.3	50.81%	motif file	svg

	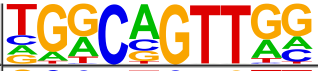									(matrix)	
59	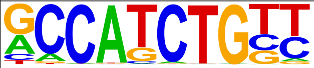	NeuroD1(bHLH)/Islet-NeuroD1-ChIP-Seq(GSE30298)/Homer	1e-84	-1.935e+02	0.0000	15234.0	42.64%	13176.1	37.61%	motif file (matrix)	svg
60	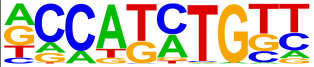	Olig2(bHLH)/Neuron-Olig2-ChIP-Seq(GSE30882)/Homer	1e-82	-1.897e+02	0.0000	24855.0	69.58%	22685.5	64.76%	motif file (matrix)	svg
61	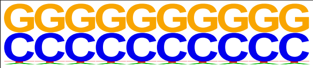	SeqBias: CG bias	1e-81	-1.886e+02	0.0000	35699.0	99.93%	34768.9	99.25%	motif file (matrix)	svg
62	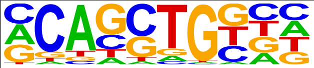	HEB(bHLH)/mES-Heb-ChIP-Seq(GSE53233)/Homer	1e-80	-1.843e+02	0.0000	26436.0	74.00%	24325.7	69.44%	motif file (matrix)	svg
63	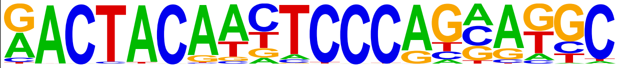	Ronin(THAP)/ES-Thap11-ChIP-Seq(GSE51522)/Homer	1e-78	-1.815e+02	0.0000	568.0	1.59%	225.2	0.64%	motif file (matrix)	svg
64	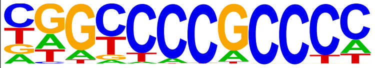	Sp2(Zf)/HEK293-Sp2.eGFP-ChIP-Seq(Encode)/Homer	1e-76	-1.766e+02	0.0000	23043.0	64.50%	20918.3	59.71%	motif file (matrix)	svg
65	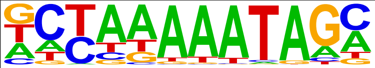	Mef2c(MADS)/GM12878-Mef2c-ChIP-Seq(GSE32465)/Homer	1e-75	-1.746e+02	0.0000	6784.0	18.99%	5380.1	15.36%	motif file (matrix)	svg
66	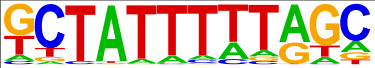	Mef2d(MADS)/Retina-Mef2d-ChIP-Seq(GSE61391)/Homer	1e-74	-1.714e+02	0.0000	3431.0	9.60%	2453.9	7.01%	motif file (matrix)	svg
67	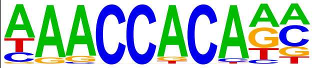	RUNX1(Runt)/Jurkat-RUNX1-ChIP-Seq(GSE29180)/Homer	1e-73	-1.684e+02	0.0000	15729.0	44.03%	13771.6	39.31%	motif file (matrix)	svg
68	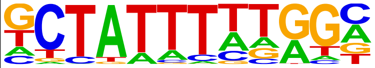	Mef2b(MADS)/HEK293-Mef2b.V5-ChIP-Seq(GSE67450)/Homer	1e-71	-1.647e+02	0.0000	11462.0	32.09%	9728.9	27.77%	motif file (matrix)	svg
69	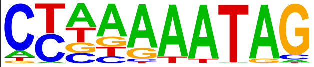	Mef2a(MADS)/HL1-Mef2a.biotin-ChIP-Seq(GSE21529)/Homer	1e-67	-1.545e+02	0.0000	6445.0	18.04%	5147.6	14.69%	motif file (matrix)	svg
70	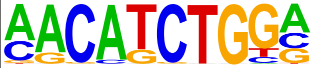	ZBTB18(Zf)/HEK293-ZBTB18.GFP-ChIP-Seq(GSE58341)/Homer	1e-66	-1.542e+02	0.0000	9113.0	25.51%	7585.3	21.65%	motif file (matrix)	svg
71	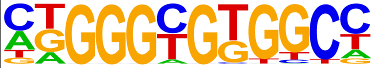	KLF6(Zf)/PDAC-KLF6-ChIP-Seq(GSE64557)/Homer	1e-66	-1.530e+02	0.0000	17682.0	49.50%	15742.8	44.94%	motif file (matrix)	svg
72	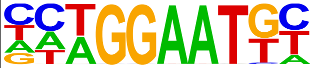	TEAD(TEA)/Fibroblast-PU.1-ChIP-Seq(Unpublished)/Homer	1e-65	-1.503e+02	0.0000	11417.0	31.96%	9753.1	27.84%	motif file (matrix)	svg
73	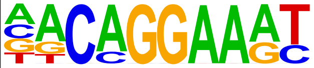	EWS:FLI1-fusion(ETS)/SK_N_MC-EWS:FLI1-ChIP-Seq(SRA014231)/Homer	1e-63	-1.460e+02	0.0000	10022.0	28.05%	8466.5	24.17%	motif file (matrix)	svg
74	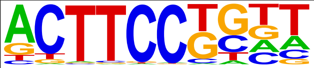	Elf4(ETS)/BMDM-Elf4-ChIP-Seq(GSE88699)/Homer	1e-59	-1.366e+02	0.0000	15956.0	44.67%	14157.0	40.41%	motif file (matrix)	svg
75	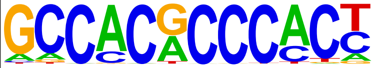	Klf9(Zf)/GBM-Klf9-ChIP-Seq(GSE62211)/Homer	1e-57	-1.333e+02	0.0000	7604.0	21.29%	6285.1	17.94%	motif file (matrix)	svg
76	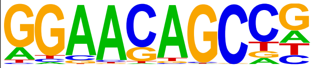	ZNF341(Zf)/EBV-ZNF341-ChIP-Seq(GSE113194)/Homer	1e-55	-1.273e+02	0.0000	11829.0	33.11%	10257.3	29.28%	motif file (matrix)	svg
77	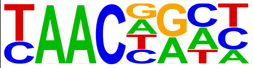	MYB101(MYB)/colamp-MYB101-DAP-Seq(GSE60143)/Homer	1e-54	-1.257e+02	0.0000	22803.0	63.83%	20949.6	59.80%	motif file (matrix)	svg
78	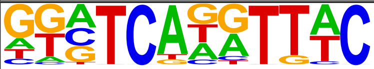	Six1(Homeobox)/Myoblast-Six1-ChIP-Chip(GSE20150)/Homer	1e-52	-1.202e+02	0.0000	4135.0	11.58%	3208.1	9.16%	motif file (matrix)	svg

79	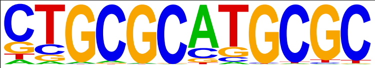	NRF1(NRF)/MCF7-NRF1-ChIP-Seq(Unpublished)/Homer	1e-49	-1.144e+02	0.0000	1803.0	5.05%	1228.8	3.51%	motif file (matrix)	svg
80	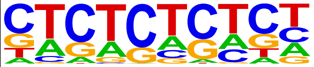	GAGA-repeat/Arabidopsis-Promoters/Homer	1e-47	-1.087e+02	0.0000	18836.0	52.73%	17127.5	48.89%	motif file (matrix)	svg
81	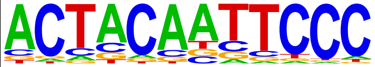	GFY(?)/Promoter/Homer	1e-46	-1.069e+02	0.0000	1077.0	3.01%	662.7	1.89%	motif file (matrix)	svg
82	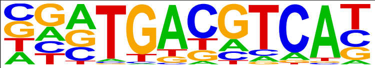	Atf2(bZIP)/3T3L1-Atf2-ChIP-Seq(GSE56872)/Homer	1e-46	-1.064e+02	0.0000	5354.0	14.99%	4349.6	12.42%	motif file (matrix)	svg
83	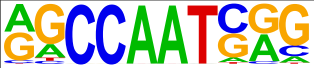	NFY(CCAAT)/Promoter/Homer	1e-45	-1.055e+02	0.0000	10053.0	28.14%	8698.5	24.83%	motif file (matrix)	svg
84	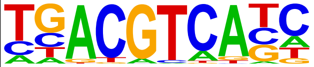	TGA6(bZIP)/colamp-TGA6-DAP-Seq(GSE60143)/Homer	1e-45	-1.045e+02	0.0000	7526.0	21.07%	6345.2	18.11%	motif file (matrix)	svg
85	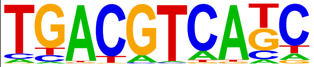	TGA1(bZIP)/colamp-TGA1-DAP-Seq(GSE60143)/Homer	1e-43	-1.005e+02	0.0000	5330.0	14.92%	4352.0	12.42%	motif file (matrix)	svg
86	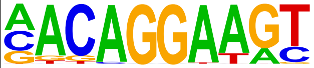	Ets1-distal(ETS)/CD4+-PolII-ChIP-Seq(Barski_et_al.)/Homer	1e-42	-9.803e+01	0.0000	5228.0	14.63%	4270.4	12.19%	motif file (matrix)	svg
87	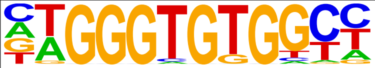	EKLF(Zf)/Erythrocyte-Klf1-ChIP-Seq(GSE20478)/Homer	1e-42	-9.718e+01	0.0000	3946.0	11.05%	3122.7	8.91%	motif file (matrix)	svg
88	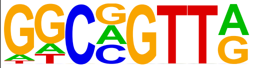	MYB(HTH)/ERMYB-Myb-ChIPSeq(GSE22095)/Homer	1e-41	-9.639e+01	0.0000	22122.0	61.93%	20453.6	58.39%	motif file (matrix)	svg
89	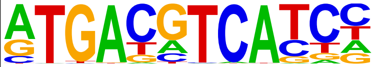	c-Jun-CRE(bZIP)/K562-cJun-ChIP-Seq(GSE31477)/Homer	1e-41	-9.514e+01	0.0000	4833.0	13.53%	3925.5	11.21%	motif file (matrix)	svg
90	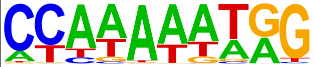	TAGL1(MADS)/Tomato-TAGL1-ChIP-Seq(GSE116581)/Homer	1e-41	-9.457e+01	0.0000	14469.0	40.50%	12973.3	37.03%	motif file (matrix)	svg
91	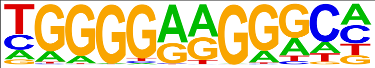	ZNF467(Zf)/HEK293-ZNF467.GFP-ChIP-Seq(GSE58341)/Homer	1e-41	-9.443e+01	0.0000	16382.0	45.86%	14825.9	42.32%	motif file (matrix)	svg
92	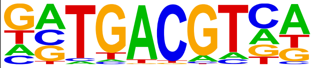	bZIP50(bZIP)/colamp-bZIP50-DAP-Seq(GSE60143)/Homer	1e-40	-9.266e+01	0.0000	11839.0	33.14%	10465.0	29.87%	motif file (matrix)	svg
93	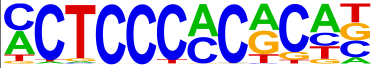	WT1(Zf)/Kidney-WT1-ChIP-Seq(GSE90016)/Homer	1e-40	-9.240e+01	0.0000	11459.0	32.08%	10105.2	28.85%	motif file (matrix)	svg
94	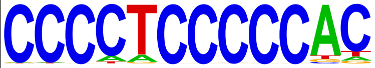	Zfp281(Zf)/ES-Zfp281-ChIP-Seq(GSE81042)/Homer	1e-40	-9.231e+01	0.0000	5270.0	14.75%	4333.4	12.37%	motif file (matrix)	svg
95	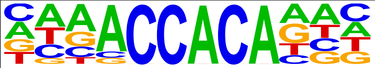	RUNX2(Runt)/PCa-RUNX2-ChIP-Seq(GSE33889)/Homer	1e-39	-9.129e+01	0.0000	13262.0	37.12%	11834.4	33.78%	motif file (matrix)	svg
96	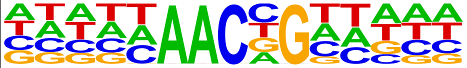	MYB73(MYB)/col-MYB73-DAP-Seq(GSE60143)/Homer	1e-39	-9.062e+01	0.0000	21580.0	60.41%	19954.1	56.96%	motif file (matrix)	svg
97	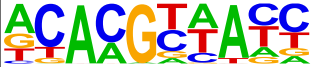	ANAC046(NAC)/colamp-ANAC046-DAP-Seq(GSE60143)/Homer	1e-39	-9.022e+01	0.0000	20573.0	57.59%	18960.8	54.13%	motif file (matrix)	svg
98	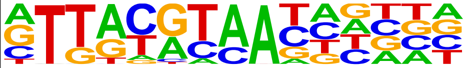	NFIL3(bZIP)/HepG2-NFIL3-ChIP-Seq(Encode)/Homer	1e-38	-8.949e+01	0.0000	7510.0	21.02%	6407.6	18.29%	motif file (matrix)	svg
99		SPDEF(ETS)/VCaP-SPDEF-ChIP-Seq(SRA014231)/Homer	1e-38	-8.930e+01	0.0000	15902.0	44.51%	14394.0	41.09%	motif file	svg

	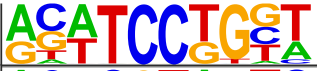									(matrix)	
100	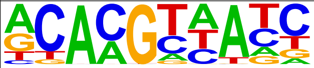	ANAC038(NAC)/col-ANAC038-DAP-Seq(GSE60143)/Homer	1e-38	-8.893e+01	0.0000	22302.0	62.43%	20683.6	59.05%	motif file (matrix)	svg
101	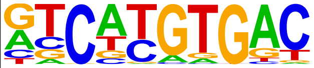	MITF(bHLH)/MastCells-MITF-ChIP-Seq(GSE48085)/Homer	1e-38	-8.788e+01	0.0000	13653.0	38.22%	12232.8	34.92%	motif file (matrix)	svg
102	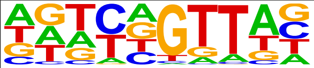	MYB33(MYB)/col-MYB33-DAP-Seq(GSE60143)/Homer	1e-36	-8.313e+01	0.0000	19879.0	55.65%	18326.2	52.32%	motif file (matrix)	svg
103	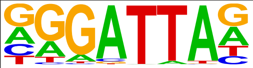	GSC(Homeobox)/FrogEmbryos-GSC-ChIP-Seq(DRA000576)/Homer	1e-35	-8.202e+01	0.0000	14140.0	39.58%	12741.5	36.37%	motif file (matrix)	svg
104	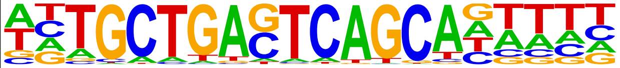	MafB(bZIP)/BMM-Mafb-ChIP-Seq(GSE75722)/Homer	1e-34	-8.003e+01	0.0000	6923.0	19.38%	5913.6	16.88%	motif file (matrix)	svg
105	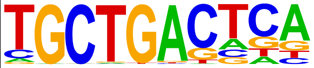	MafA(bZIP)/Islet-MafA-ChIP-Seq(GSE30298)/Homer	1e-34	-7.849e+01	0.0000	13312.0	37.26%	11970.0	34.17%	motif file (matrix)	svg
106	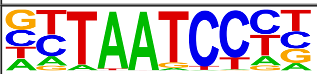	Otx2(Homeobox)/EpiLC-Otx2-ChIP-Seq(GSE56098)/Homer	1e-33	-7.770e+01	0.0000	10575.0	29.60%	9359.3	26.72%	motif file (matrix)	svg
107	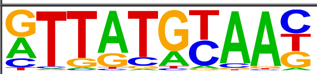	HLF(bZIP)/HSC-HLF.Flag-ChIP-Seq(GSE69817)/Homer	1e-32	-7.527e+01	0.0000	9399.0	26.31%	8261.7	23.58%	motif file (matrix)	svg
108	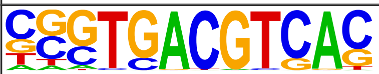	CRE(bZIP)/Promoter/Homer	1e-32	-7.481e+01	0.0000	3595.0	10.06%	2896.3	8.27%	motif file (matrix)	svg
109	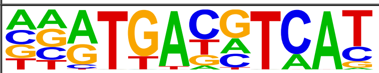	CREB5(bZIP)/LNCaP-CREB5.V5-ChIP-Seq(GSE137775)/Homer	1e-32	-7.475e+01	0.0000	5745.0	16.08%	4853.0	13.85%	motif file (matrix)	svg
110	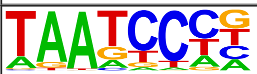	Pitx1(Homeobox)/Chicken-Pitx1-ChIP-Seq(GSE38910)/Homer	1e-32	-7.452e+01	0.0000	32945.0	92.22%	31674.2	90.42%	motif file (matrix)	svg
111	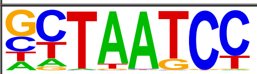	CRX(Homeobox)/Retina-Crx-ChIP-Seq(GSE20012)/Homer	1e-32	-7.400e+01	0.0000	24616.0	68.91%	23102.9	65.95%	motif file (matrix)	svg
112	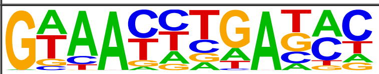	Six2(Homeobox)/NephronProgenitor-Six2-ChIP-Seq(GSE39837)/Homer	1e-32	-7.397e+01	0.0000	14116.0	39.52%	12776.0	36.47%	motif file (matrix)	svg
113	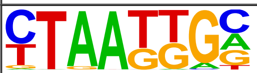	Isl1(Homeobox)/Neuron-Isl1-ChIP-Seq(GSE31456)/Homer	1e-31	-7.356e+01	0.0000	21329.0	59.71%	19830.9	56.61%	motif file (matrix)	svg
114	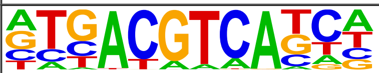	TGA4(bZIP)/colamp-TGA4-DAP-Seq(GSE60143)/Homer	1e-31	-7.349e+01	0.0000	3756.0	10.51%	3046.7	8.70%	motif file (matrix)	svg
115	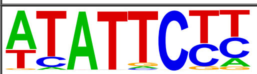	KAN2(G2like)/colamp-KAN2-DAP-Seq(GSE60143)/Homer	1e-31	-7.303e+01	0.0000	16557.0	46.35%	15149.1	43.25%	motif file (matrix)	svg
116	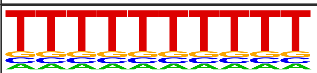	VRN1(ABI3VP1)/col-VRN1-DAP-Seq(GSE60143)/Homer	1e-30	-7.135e+01	0.0000	5219.0	14.61%	4386.5	12.52%	motif file (matrix)	svg
117	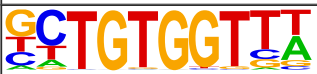	RUNX-AML(Runt)/CD4+-PolII-ChIP-Seq(Barski_et_al.)/Homer	1e-30	-7.118e+01	0.0000	12091.0	33.85%	10850.6	30.97%	motif file (matrix)	svg
118	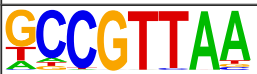	AT3G10030(Trihelix)/colamp-AT3G10030-DAP-Seq(GSE60143)/Homer	1e-30	-7.089e+01	0.0000	7381.0	20.66%	6392.1	18.25%	motif file (matrix)	svg
119	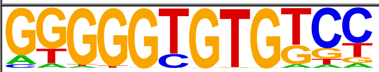	KLF10(Zf)/HEK293-KLF10.GFP-ChIP-Seq(GSE58341)/Homer	1e-30	-6.909e+01	0.0000	10799.0	30.23%	9631.7	27.50%	motif file (matrix)	svg

120	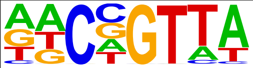	MYB65(MYB)/colamp-MYB65-DAP-Seq(GSE60143)/Homer	1e-29	-6.850e+01	0.0000	14512.0	40.62%	13200.3	37.68%	motif file (matrix)	svg
121	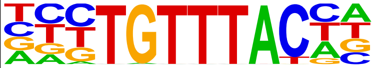	FOXP1(Forkhead)/H9-FOXP1-ChIP-Seq(GSE31006)/Homer	1e-29	-6.849e+01	0.0000	6262.0	17.53%	5364.7	15.31%	motif file (matrix)	svg
122	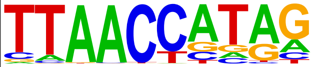	AT3G25990(Trihelix)/colamp-AT3G25990-DAP-Seq(GSE60143)/Homer	1e-29	-6.784e+01	0.0000	9016.0	25.24%	7948.7	22.69%	motif file (matrix)	svg
123	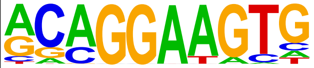	ETS1(ETS)/Jurkat-ETS1-ChIP-Seq(GSE17954)/Homer	1e-28	-6.673e+01	0.0000	17981.0	50.33%	16589.5	47.36%	motif file (matrix)	svg
124	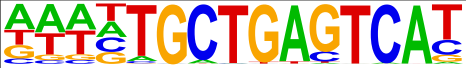	Bach1(bZIP)/K562-Bach1-ChIP-Seq(GSE31477)/Homer	1e-28	-6.586e+01	0.0000	1756.0	4.92%	1310.0	3.74%	motif file (matrix)	svg
125	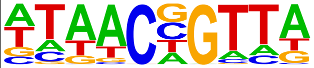	MYB70(MYB)/col-MYB70-DAP-Seq(GSE60143)/Homer	1e-28	-6.539e+01	0.0000	17615.0	49.31%	16242.9	46.37%	motif file (matrix)	svg
126	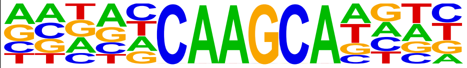	AT5G25475(ABI3VP1)/col-AT5G25475-DAP-Seq(GSE60143)/Homer	1e-28	-6.510e+01	0.0000	15677.0	43.88%	14356.7	40.98%	motif file (matrix)	svg
127	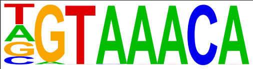	Foxo3(Forkhead)/U2OS-Foxo3-ChIP-Seq(E-MTAB-2701)/Homer	1e-28	-6.500e+01	0.0000	9879.0	27.65%	8785.6	25.08%	motif file (matrix)	svg
128	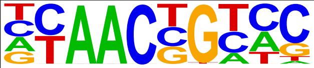	MYB77(MYB)/col-MYB77-DAP-Seq(GSE60143)/Homer	1e-28	-6.480e+01	0.0000	17093.0	47.85%	15737.7	44.93%	motif file (matrix)	svg
129	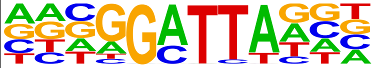	bcd(Homeobox)/Embryo-Bcd-ChIP-Seq(GSE86966)/Homer	1e-27	-6.441e+01	0.0000	15223.0	42.61%	13921.3	39.74%	motif file (matrix)	svg
130	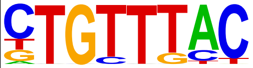	Foxo1(Forkhead)/RAW-Foxo1-ChIP-Seq(Fan_et_al.)/Homer	1e-27	-6.417e+01	0.0000	23140.0	64.78%	21703.3	61.96%	motif file (matrix)	svg
131	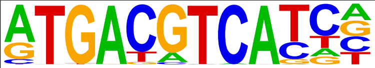	JunD(bZIP)/K562-JunD-ChIP-Seq/Homer	1e-27	-6.415e+01	0.0000	1387.0	3.88%	1001.3	2.86%	motif file (matrix)	svg
132	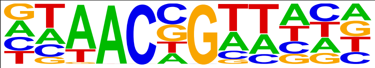	MYB81(MYB)/col-MYB81-DAP-Seq(GSE60143)/Homer	1e-27	-6.274e+01	0.0000	17891.0	50.08%	16534.5	47.20%	motif file (matrix)	svg
133	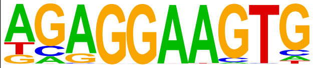	PU.1(ETS)/ThioMac-PU.1-ChIP-Seq(GSE21512)/Homer	1e-27	-6.263e+01	0.0000	8394.0	23.50%	7396.2	21.11%	motif file (matrix)	svg
134	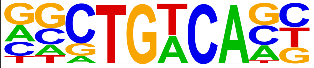	Meis1(Homeobox)/MastCells-Meis1-ChIP-Seq(GSE48085)/Homer	1e-26	-6.210e+01	0.0000	21558.0	60.35%	20149.3	57.52%	motif file (matrix)	svg
135	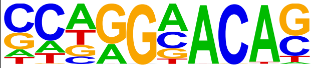	AR-halfsite(NR)/LNCaP-AR-ChIP-Seq(GSE27824)/Homer	1e-26	-6.185e+01	0.0000	32992.0	92.36%	31786.6	90.74%	motif file (matrix)	svg
136	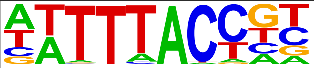	GTL1(Trihelix)/colamp-GTL1-DAP-Seq(GSE60143)/Homer	1e-26	-6.129e+01	0.0000	13386.0	37.47%	12170.7	34.74%	motif file (matrix)	svg
137	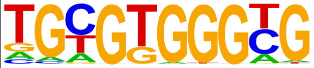	Egr1(Zf)/K562-Egr1-ChIP-Seq(GSE32465)/Homer	1e-26	-6.090e+01	0.0000	12156.0	34.03%	10990.8	31.38%	motif file (matrix)	svg
138	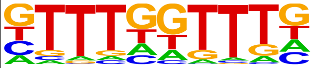	HuR(?)/HEK293-HuR-CLIP-Seq(GSE87887)/Homer	1e-26	-6.050e+01	0.0000	29958.0	83.86%	28622.6	81.71%	motif file (matrix)	svg
139	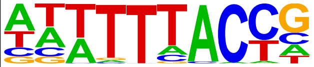	AT5G47660(Trihelix)/colamp-AT5G47660-DAP-Seq(GSE60143)/Homer	1e-26	-6.028e+01	0.0000	16721.0	46.81%	15412.4	44.00%	motif file (matrix)	svg
140		FOXK2(Forkhead)/U2OS-FOXK2-ChIP-Seq(E-MTAB-2204)/Homer	1e-25	-5.935e+01	0.0000	8976.0	25.13%	7969.9	22.75%	motif file	svg

	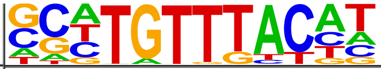									(matrix)	
141	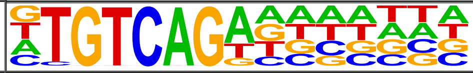	AtGRF6(GRF)/col-AtGRF6-DAP-Seq(GSE60143)/Homer	1e-25	-5.912e+01	0.0000	17344.0	48.55%	16030.6	45.76%	motif file (matrix)	svg
142	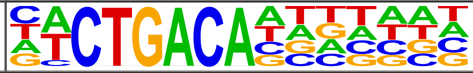	GRF9(GRF)/colamp-GRF9-DAP-Seq(GSE60143)/Homer	1e-25	-5.909e+01	0.0000	14573.0	40.79%	13335.2	38.07%	motif file (matrix)	svg
143	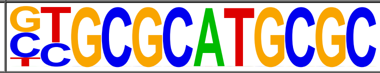	NRF(NRF)/Promoter/Homer	1e-25	-5.856e+01	0.0000	2250.0	6.30%	1764.9	5.04%	motif file (matrix)	svg
144	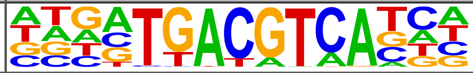	TGA5(bZIP)/col-TGA5-DAP-Seq(GSE60143)/Homer	1e-25	-5.830e+01	0.0000	1238.0	3.47%	891.3	2.54%	motif file (matrix)	svg
145	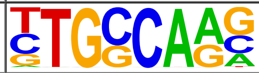	NF1-halfsite(CTF)/LNCaP-NF1-ChIP-Seq(Unpublished)/Homer	1e-25	-5.799e+01	0.0000	24896.0	69.69%	23508.4	67.11%	motif file (matrix)	svg
146	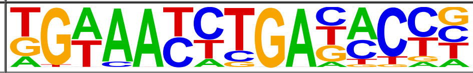	Six4(Homeobox)/MCF7-SIX4-ChIP-Seq(Encode)/Homer	1e-25	-5.771e+01	0.0000	912.0	2.55%	622.7	1.78%	motif file (matrix)	svg
147	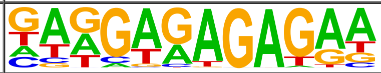	BPC1(BBRBPC)/colamp-BPC1-DAP-Seq(GSE60143)/Homer	1e-24	-5.650e+01	0.0000	10938.0	30.62%	9858.7	28.14%	motif file (matrix)	svg
148	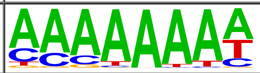	REM19(REM)/colamp-REM19-DAP-Seq(GSE60143)/Homer	1e-24	-5.580e+01	0.0000	11090.0	31.04%	10009.1	28.57%	motif file (matrix)	svg
149	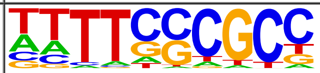	DEL2(E2FDP)/col-DEL2-DAP-Seq(GSE60143)/Homer	1e-23	-5.421e+01	0.0000	5196.0	14.55%	4459.1	12.73%	motif file (matrix)	svg
150	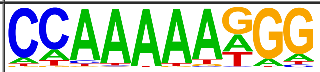	SEP3(MADS)/Arabidoposis-Flower-Sep3-ChIP-Seq/Homer	1e-23	-5.415e+01	0.0000	16314.0	45.67%	15069.0	43.02%	motif file (matrix)	svg
151	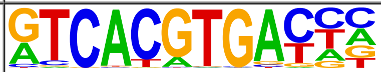	TFE3(bHLH)/MEF-TFE3-ChIP-Seq(GSE75757)/Homer	1e-23	-5.339e+01	0.0000	1394.0	3.90%	1037.4	2.96%	motif file (matrix)	svg
152	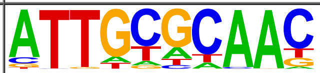	CEBP(bZIP)/ThioMac-CEBPb-ChIP-Seq(GSE21512)/Homer	1e-22	-5.228e+01	0.0000	7932.0	22.20%	7033.7	20.08%	motif file (matrix)	svg
153	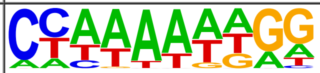	RIN(MADS)/Tomato-RIN-ChIP-Seq(GSE116581)/Homer	1e-22	-5.207e+01	0.0000	15855.0	44.38%	14641.0	41.80%	motif file (matrix)	svg
154	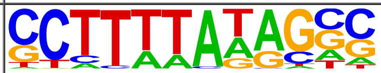	TATA-Box(TBP)/Promoter/Homer	1e-22	-5.201e+01	0.0000	16101.0	45.07%	14881.9	42.48%	motif file (matrix)	svg
155	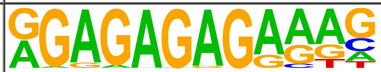	FRS9(ND)/col-FRS9-DAP-Seq(GSE60143)/Homer	1e-22	-5.173e+01	0.0000	5662.0	15.85%	4908.0	14.01%	motif file (matrix)	svg
156	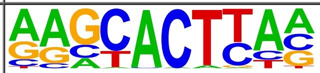	Nkx3.1(Homeobox)/LNCaP-Nkx3.1-ChIP-Seq(GSE28264)/Homer	1e-22	-5.167e+01	0.0000	25453.0	71.25%	24120.2	68.86%	motif file (matrix)	svg
157	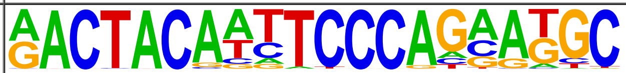	GFY-Staf(?,Zf)/Promoter/Homer	1e-22	-5.152e+01	0.0000	878.0	2.46%	608.4	1.74%	motif file (matrix)	svg
158	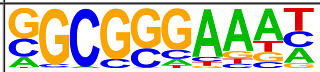	E2F4(E2F)/K562-E2F4-ChIP-Seq(GSE31477)/Homer	1e-22	-5.142e+01	0.0000	6293.0	17.62%	5498.7	15.70%	motif file (matrix)	svg
159	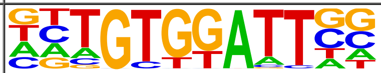	Foxh1(Forkhead)/hESC-FOXH1-ChIP-Seq(GSE29422)/Homer	1e-22	-5.106e+01	0.0000	9057.0	25.35%	8108.5	23.15%	motif file (matrix)	svg
160	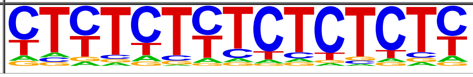	GAGA-repeat/SacCer-Promoters/Homer	1e-21	-5.064e+01	0.0000	33651.0	94.20%	32548.9	92.92%	motif file (matrix)	svg

161	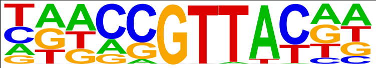	MYB119(MYB)/colamp-MYB119-DAP-Seq(GSE60143)/Homer	1e-21	-4.961e+01	0.0000	3722.0	10.42%	3128.7	8.93%	motif file (matrix)	svg
162	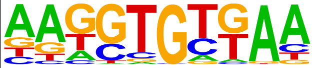	Tbr1(T-box)/Cortex-Tbr1-ChIP-Seq(GSE71384)/Homer	1e-21	-4.953e+01	0.0000	17931.0	50.19%	16691.6	47.65%	motif file (matrix)	svg
163	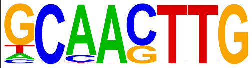	bHLH130(bHLH)/col-bHLH130-DAP-Seq(GSE60143)/Homer	1e-21	-4.884e+01	0.0000	9526.0	26.67%	8572.4	24.47%	motif file (matrix)	svg
164	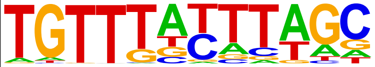	FoxD3(forkhead)/ZebrafishEmbryo-Foxd3.biotin-ChIP-seq(GSE106676)/Homer	1e-20	-4.766e+01	0.0000	11068.0	30.98%	10056.0	28.71%	motif file (matrix)	svg
165	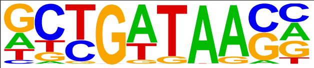	Unknown5/Drosophila-Promoters/Homer	1e-20	-4.764e+01	0.0000	8211.0	22.99%	7332.1	20.93%	motif file (matrix)	svg
166	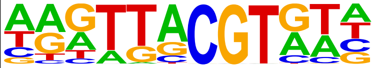	NAP(NAC)/col-NAP-DAP-Seq(GSE60143)/Homer	1e-20	-4.712e+01	0.0000	10260.0	28.72%	9287.1	26.51%	motif file (matrix)	svg
167	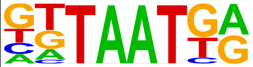	Nkx6.1(Homeobox)/Islet-Nkx6.1-ChIP-Seq(GSE40975)/Homer	1e-20	-4.703e+01	0.0000	24124.0	67.53%	22833.9	65.18%	motif file (matrix)	svg
168	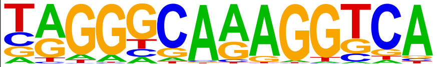	RXR(NR),DR1/3T3L1-RXR-ChIP-Seq(GSE13511)/Homer	1e-20	-4.698e+01	0.0000	17867.0	50.02%	16653.6	47.54%	motif file (matrix)	svg
169	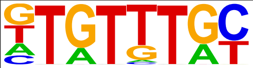	PHA-4(Forkhead)/cElegans-Embryos-PHA4-ChIP-Seq(modEncode)/Homer	1e-20	-4.647e+01	0.0000	28686.0	80.30%	27426.4	78.29%	motif file (matrix)	svg
170	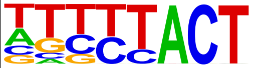	At3g60580(C2H2)/col-At3g60580-DAP-Seq(GSE60143)/Homer	1e-20	-4.618e+01	0.0000	34722.0	97.20%	33737.5	96.31%	motif file (matrix)	svg
171	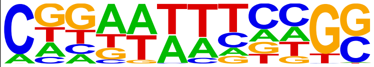	LBD18(LOBAS2)/colamp-LBD18-DAP-Seq(GSE60143)/Homer	1e-20	-4.617e+01	0.0000	27651.0	77.40%	26381.0	75.31%	motif file (matrix)	svg
172	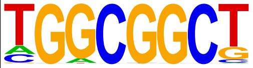	ERF105(AP2EREBP)/colamp-ERF105-DAP-Seq(GSE60143)/Homer	1e-20	-4.607e+01	0.0000	13574.0	38.00%	12484.6	35.64%	motif file (matrix)	svg
173	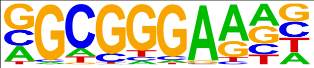	E2F6(E2F)/Hela-E2F6-ChIP-Seq(GSE31477)/Homer	1e-19	-4.574e+01	0.0000	8039.0	22.50%	7184.4	20.51%	motif file (matrix)	svg
174	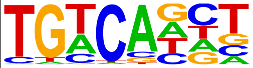	Tgif2(Homeobox)/mES-Tgif2-ChIP-Seq(GSE55404)/Homer	1e-19	-4.567e+01	0.0000	30214.0	84.58%	28991.1	82.76%	motif file (matrix)	svg
175	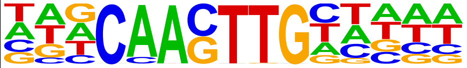	bHLH122(bHLH)/col100-bHLH122-DAP-Seq(GSE60143)/Homer	1e-19	-4.553e+01	0.0000	11414.0	31.95%	10407.7	29.71%	motif file (matrix)	svg
176	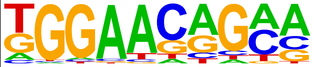	ZNF189(Zf)/HEK293-ZNF189.GFP-ChIP-Seq(GSE58341)/Homer	1e-19	-4.483e+01	0.0000	13001.0	36.39%	11942.6	34.09%	motif file (matrix)	svg
177	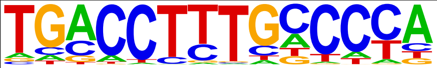	PPARE(NR),DR1/3T3L1-Pparg-ChIP-Seq(GSE13511)/Homer	1e-18	-4.370e+01	0.0000	15405.0	43.12%	14283.0	40.77%	motif file (matrix)	svg
178	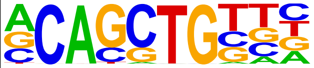	Ptf1a(bHLH)/Panc1-Ptf1a-ChIP-Seq(GSE47459)/Homer	1e-18	-4.284e+01	0.0000	30303.0	84.83%	29104.8	83.08%	motif file (matrix)	svg
179	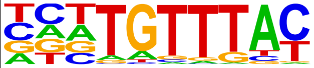	FOXK1(Forkhead)/HEK293-FOXK1-ChIP-Seq(GSE51673)/Homer	1e-18	-4.282e+01	0.0000	13336.0	37.33%	12285.8	35.07%	motif file (matrix)	svg
180	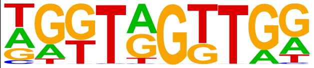	MYB96(MYB)/colamp-MYB96-DAP-Seq(GSE60143)/Homer	1e-18	-4.262e+01	0.0000	12235.0	34.25%	11224.3	32.04%	motif file (matrix)	svg
181		SeqBias: CA-repeat	1e-18	-4.260e+01	0.0000	35014.0	98.02%	34079.5	97.29%	motif file	svg

	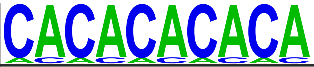									(matrix)	
182	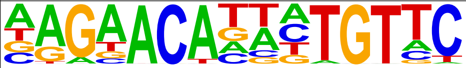	PGR(NR)/EndoStromal-PGR-ChIP-Seq(GSE69539)/Homer	1e-18	-4.183e+01	0.0000	3069.0	8.59%	2573.5	7.35%	motif file (matrix)	svg
183	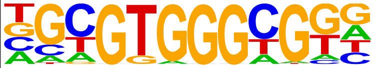	Egr2(Zf)/Thymocytes-Egr2-ChIP-Seq(GSE34254)/Homer	1e-18	-4.162e+01	0.0000	3220.0	9.01%	2712.5	7.74%	motif file (matrix)	svg
184	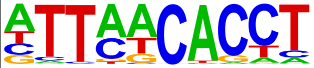	Eomes(T-box)/H9-Eomes-ChIP-Seq(GSE26097)/Homer	1e-18	-4.162e+01	0.0000	24216.0	67.79%	22977.4	65.59%	motif file (matrix)	svg
185	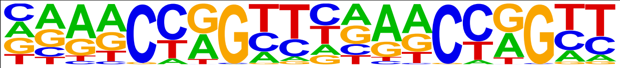	Tcfcp2l1(CP2)/mES-Tcfcp2l1-ChIP-Seq(GSE11431)/Homer	1e-17	-4.118e+01	0.0000	2427.0	6.79%	1994.5	5.69%	motif file (matrix)	svg
186	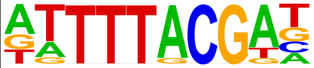	ARF16(ARF)/col-ARF16-DAP-Seq(GSE60143)/Homer	1e-17	-4.085e+01	0.0000	1657.0	4.64%	1309.0	3.74%	motif file (matrix)	svg
187	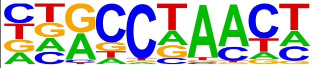	BOS1(MYB)/col-BOS1-DAP-Seq(GSE60143)/Homer	1e-17	-4.060e+01	0.0000	11331.0	31.72%	10373.9	29.61%	motif file (matrix)	svg
188	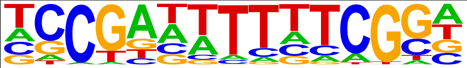	LBD2(LOBAS2)/colamp-LBD2-DAP-Seq(GSE60143)/Homer	1e-17	-4.052e+01	0.0000	3621.0	10.14%	3086.6	8.81%	motif file (matrix)	svg
189	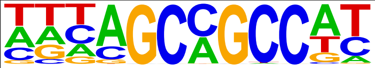	ERF15(AP2EREBP)/colamp-ERF15-DAP-Seq(GSE60143)/Homer	1e-17	-4.047e+01	0.0000	23335.0	65.32%	22112.1	63.12%	motif file (matrix)	svg
190	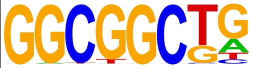	AT5G23930(mTERF)/col-AT5G23930-DAP-Seq(GSE60143)/Homer	1e-17	-4.010e+01	0.0000	18837.0	52.73%	17674.4	50.45%	motif file (matrix)	svg
191	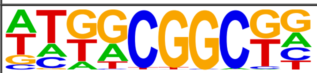	At2g33710(AP2EREBP)/colamp-At2g33710-DAP-Seq(GSE60143)/Homer	1e-17	-3.975e+01	0.0000	16433.0	46.00%	15325.8	43.75%	motif file (matrix)	svg
192	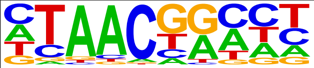	MYB56(MYB)/colamp-MYB56-DAP-Seq(GSE60143)/Homer	1e-17	-3.975e+01	0.0000	9974.0	27.92%	9079.8	25.92%	motif file (matrix)	svg
193	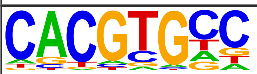	IBL1(bHLH)/Seedling-IBL1-ChIP-Seq(GSE51120)/Homer	1e-17	-3.973e+01	0.0000	22170.0	62.06%	20964.5	59.85%	motif file (matrix)	svg
194	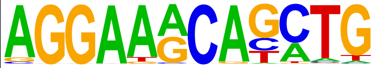	ETS:E-box(ETS,bHLH)/HPC7-Scl-ChIP-Seq(GSE22178)/Homer	1e-17	-3.972e+01	0.0000	1721.0	4.82%	1369.0	3.91%	motif file (matrix)	svg
195	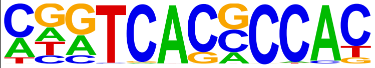	Srebp2(bHLH)/HepG2-Srebp2-ChIP-Seq(GSE31477)/Homer	1e-16	-3.797e+01	0.0000	2539.0	7.11%	2111.6	6.03%	motif file (matrix)	svg
196	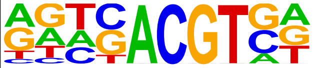	HY5(bZIP)/colamp-HY5-DAP-Seq(GSE60143)/Homer	1e-16	-3.766e+01	0.0000	10959.0	30.68%	10044.9	28.67%	motif file (matrix)	svg
197	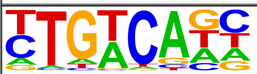	Tgif1(Homeobox)/mES-Tgif1-ChIP-Seq(GSE55404)/Homer	1e-16	-3.758e+01	0.0000	28856.0	80.78%	27675.9	79.01%	motif file (matrix)	svg
198	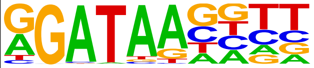	At1g49010(MYBrelated)/col-At1g49010-DAP-Seq(GSE60143)/Homer	1e-15	-3.681e+01	0.0000	20009.0	56.01%	18862.2	53.85%	motif file (matrix)	svg
199	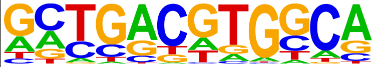	O2(bZIP)/Corn-O2-ChIP-Seq(GSE63991)/Homer	1e-15	-3.614e+01	0.0000	2286.0	6.40%	1891.2	5.40%	motif file (matrix)	svg
200	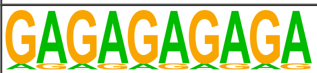	SeqBias: GA-repeat	1e-15	-3.595e+01	0.0000	35653.0	99.80%	34871.2	99.55%	motif file (matrix)	svg
201	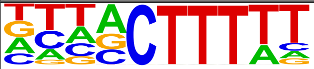	OBP3(C2C2dof)/col-OBP3-DAP-Seq(GSE60143)/Homer	1e-15	-3.573e+01	0.0000	29166.0	81.64%	28007.0	79.95%	motif file (matrix)	svg

202	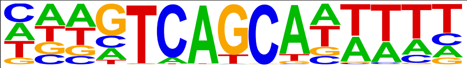	MafF(bZIP)/HepG2-MafF-ChIP-Seq(GSE31477)/Homer	1e-15	-3.552e+01	0.0000	3855.0	10.79%	3333.2	9.52%	motif file (matrix)	svg
203	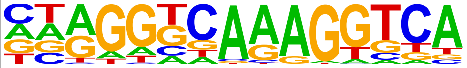	PPARa(NR),DR1/Liver-Ppara-ChIP-Seq(GSE47954)/Homer	1e-15	-3.508e+01	0.0000	16228.0	45.43%	15176.4	43.32%	motif file (matrix)	svg
204	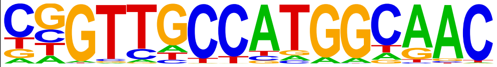	RFX(HTH)/K562-RFX3-ChIP-Seq(SRA012198)/Homer	1e-15	-3.500e+01	0.0000	1252.0	3.50%	974.6	2.78%	motif file (matrix)	svg
205	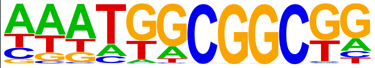	ABR1(AP2EREBP)/colamp-ABR1-DAP-Seq(GSE60143)/Homer	1e-15	-3.494e+01	0.0000	9267.0	25.94%	8448.5	24.12%	motif file (matrix)	svg
206	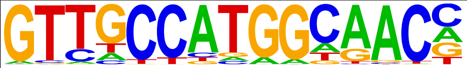	Rfx2(HTH)/LoVo-RFX2-ChIP-Seq(GSE49402)/Homer	1e-15	-3.468e+01	0.0000	1427.0	3.99%	1129.1	3.22%	motif file (matrix)	svg
207	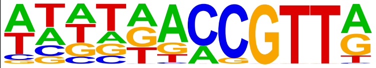	MYB3R1(MYB)/col-MYB3R1-DAP-Seq(GSE60143)/Homer	1e-14	-3.444e+01	0.0000	3235.0	9.06%	2767.7	7.90%	motif file (matrix)	svg
208	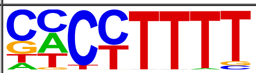	AT5G02460(C2C2dof)/col-AT5G02460-DAP-Seq(GSE60143)/Homer	1e-14	-3.361e+01	0.0000	24995.0	69.97%	23837.2	68.05%	motif file (matrix)	svg
209	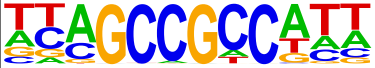	ERF13(AP2EREBP)/colamp-ERF13-DAP-Seq(GSE60143)/Homer	1e-14	-3.341e+01	0.0000	11764.0	32.93%	10863.4	31.01%	motif file (matrix)	svg
210	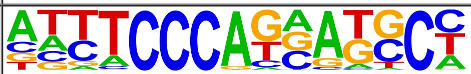	ZNF143|STAF(Zf)/CUTLL-ZNF143-ChIP-Seq(GSE29600)/Homer	1e-14	-3.323e+01	0.0000	6029.0	16.88%	5385.4	15.37%	motif file (matrix)	svg
211	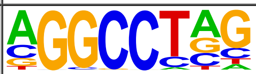	ZNF711(Zf)/SHSY5Y-ZNF711-ChIP-Seq(GSE20673)/Homer	1e-14	-3.318e+01	0.0000	24630.0	68.95%	23478.2	67.02%	motif file (matrix)	svg
212	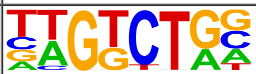	Smad3(MAD)/NPC-Smad3-ChIP-Seq(GSE36673)/Homer	1e-14	-3.317e+01	0.0000	30393.0	85.08%	29276.3	83.57%	motif file (matrix)	svg
213	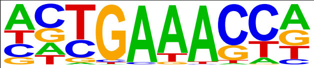	IRF4(IRF)/GM12878-IRF4-ChIP-Seq(GSE32465)/Homer	1e-14	-3.310e+01	0.0000	6693.0	18.74%	6015.0	17.17%	motif file (matrix)	svg
214	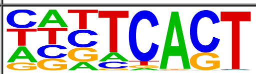	STZ(C2H2)/colamp-STZ-DAP-Seq(GSE60143)/Homer	1e-14	-3.306e+01	0.0000	35233.0	98.63%	34362.3	98.09%	motif file (matrix)	svg
215	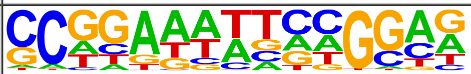	ASL18(LOBAS2)/colamp-ASL18-DAP-Seq(GSE60143)/Homer	1e-14	-3.278e+01	0.0000	22232.0	62.23%	21102.4	60.24%	motif file (matrix)	svg
216	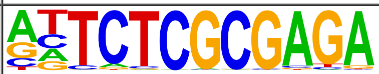	GFX(?)/Promoter/Homer	1e-14	-3.269e+01	0.0000	178.0	0.50%	92.8	0.26%	motif file (matrix)	svg
217	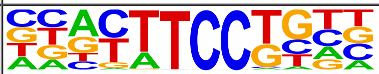	Etv2(ETS)/ES-ER71-ChIP-Seq(GSE59402)/Homer	1e-14	-3.268e+01	0.0000	16892.0	47.29%	15852.9	45.25%	motif file (matrix)	svg
218	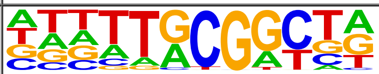	At5g08750(C3H)/col-At5g08750-DAP-Seq(GSE60143)/Homer	1e-14	-3.251e+01	0.0000	5540.0	15.51%	4930.1	14.07%	motif file (matrix)	svg
219	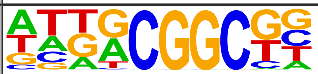	ERF115(AP2EREBP)/colamp-ERF115-DAP-Seq(GSE60143)/Homer	1e-14	-3.238e+01	0.0000	13515.0	37.83%	12569.0	35.88%	motif file (matrix)	svg
220	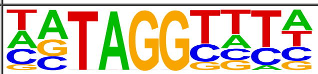	SRS7(SRS)/colamp-SRS7-DAP-Seq(GSE60143)/Homer	1e-14	-3.234e+01	0.0000	29710.0	83.17%	28589.3	81.61%	motif file (matrix)	svg
221	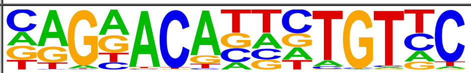	GRE(NR),IR3/RAW264.7-GRE-ChIP-Seq(Unpublished)/Homer	1e-13	-3.214e+01	0.0000	3370.0	9.43%	2906.9	8.30%	motif file (matrix)	svg
222		PR(NR)/T47D-PR-ChIP-Seq(GSE31130)/Homer	1e-13	-3.209e+01	0.0000	24953.0	69.85%	23812.4	67.98%	motif file	svg

	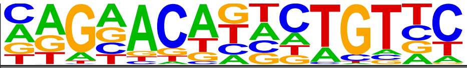									(matrix)	
223	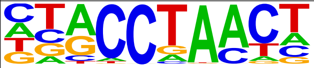	MYB62(MYB)/colamp-MYB62-DAP-Seq(GSE60143)/Homer	1e-13	-3.162e+01	0.0000	16023.0	44.85%	15016.4	42.87%	motif file (matrix)	svg
224	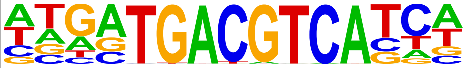	TGA3(bZIP)/colamp-TGA3-DAP-Seq(GSE60143)/Homer	1e-13	-3.158e+01	0.0000	768.0	2.15%	567.6	1.62%	motif file (matrix)	svg
225	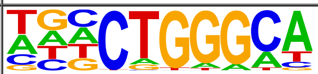	ZNF416(Zf)/HEK293-ZNF416.GFP-ChIP-Seq(GSE58341)/Homer	1e-13	-3.142e+01	0.0000	21288.0	59.59%	20185.5	57.62%	motif file (matrix)	svg
226	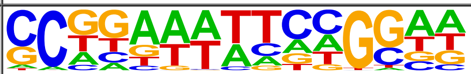	LBD19(LOBAS2)/colamp-LBD19-DAP-Seq(GSE60143)/Homer	1e-13	-3.107e+01	0.0000	23723.0	66.41%	22600.4	64.52%	motif file (matrix)	svg
227	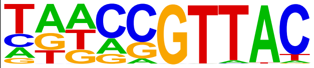	MYB118(MYB)/colamp-MYB118-DAP-Seq(GSE60143)/Homer	1e-13	-3.093e+01	0.0000	3643.0	10.20%	3167.3	9.04%	motif file (matrix)	svg
228	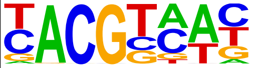	ATAF1(NAC)/col-ATAF1-DAP-Seq(GSE60143)/Homer	1e-13	-3.082e+01	0.0000	24570.0	68.78%	23445.4	66.93%	motif file (matrix)	svg
229	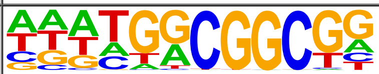	ERF4(AP2EREBP)/colamp-ERF4-DAP-Seq(GSE60143)/Homer	1e-13	-3.074e+01	0.0000	9487.0	26.56%	8701.2	24.84%	motif file (matrix)	svg
230	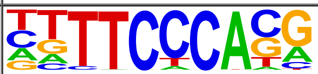	Rbpj1(?)/Panc1-Rbpj1-ChIP-Seq(GSE47459)/Homer	1e-13	-3.073e+01	0.0000	22253.0	62.29%	21147.1	60.37%	motif file (matrix)	svg
231	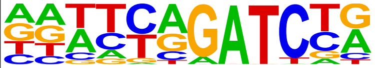	GATA4(C2C2gata)/col-GATA4-DAP-Seq(GSE60143)/Homer	1e-13	-3.059e+01	0.0000	9925.0	27.78%	9123.7	26.05%	motif file (matrix)	svg
232	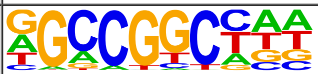	RAP211(AP2EREBP)/colamp-RAP211-DAP-Seq(GSE60143)/Homer	1e-13	-3.031e+01	0.0000	24357.0	68.18%	23239.8	66.34%	motif file (matrix)	svg
233	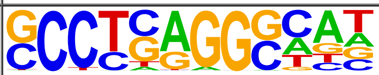	AP-2gamma(AP2)/MCF7-TFAP2C-ChIP-Seq(GSE21234)/Homer	1e-12	-2.987e+01	0.0000	18523.0	51.85%	17483.6	49.91%	motif file (matrix)	svg
234	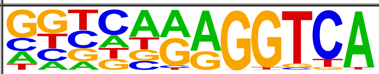	COUP-TFII(NR)/K562-NR2F1-ChIP-Seq(Encode)/Homer	1e-12	-2.976e+01	0.0000	20849.0	58.36%	19772.8	56.44%	motif file (matrix)	svg
235	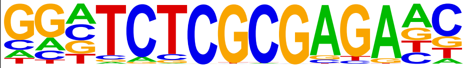	ZBTB33(Zf)/GM12878-ZBTB33-ChIP-Seq(GSE32465)/Homer	1e-12	-2.974e+01	0.0000	524.0	1.47%	367.0	1.05%	motif file (matrix)	svg
236	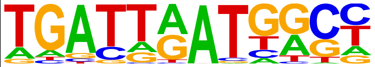	Hoxb4(Homeobox)/ES-Hoxb4-ChIP-Seq(GSE34014)/Homer	1e-12	-2.966e+01	0.0000	2613.0	7.31%	2224.2	6.35%	motif file (matrix)	svg
237	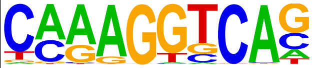	Erra(NR)/HepG2-Erra-ChIP-Seq(GSE31477)/Homer	1e-12	-2.966e+01	0.0000	26517.0	74.23%	25401.6	72.51%	motif file (matrix)	svg
238	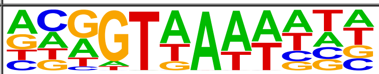	GT2(Trihelix)/colamp-GT2-DAP-Seq(GSE60143)/Homer	1e-12	-2.966e+01	0.0000	12883.0	36.06%	11987.2	34.22%	motif file (matrix)	svg
239	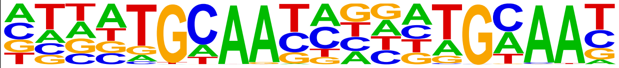	CEBP:CEBP(bZIP)/MEF-Chop-ChIP-Seq(GSE35681)/Homer	1e-12	-2.962e+01	0.0000	1863.0	5.22%	1541.9	4.40%	motif file (matrix)	svg
240	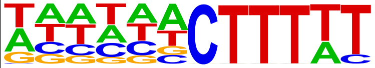	At5g62940(C2C2dof)/col-At5g62940-DAP-Seq(GSE60143)/Homer	1e-12	-2.960e+01	0.0000	30918.0	86.55%	29843.1	85.19%	motif file (matrix)	svg
241	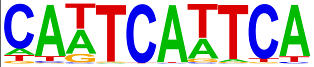	WUS1(Homeobox)/colamp-WUS1-DAP-Seq(GSE60143)/Homer	1e-12	-2.939e+01	0.0000	6145.0	17.20%	5528.2	15.78%	motif file (matrix)	svg
242	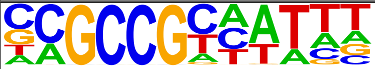	RRTF1(AP2EREBP)/colamp-RRTF1-DAP-Seq(GSE60143)/Homer	1e-12	-2.938e+01	0.0000	2302.0	6.44%	1941.7	5.54%	motif file (matrix)	svg

243	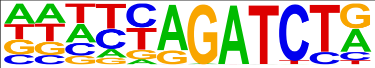	GATA11(C2C2gata)/col-GATA11-DAP-Seq(GSE60143)/Homer	1e-12	-2.936e+01	0.0000	5725.0	16.03%	5131.1	14.65%	motif file (matrix)	svg
244	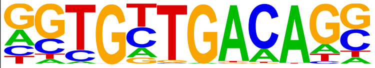	Tbx20(T-box)/Heart-Tbx20-ChIP-Seq(GSE29636)/Homer	1e-12	-2.903e+01	0.0000	3533.0	9.89%	3078.3	8.79%	motif file (matrix)	svg
245	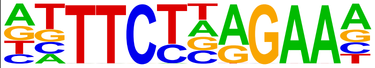	STAT5(Stat)/mCD4+-Stat5-ChIP-Seq(GSE12346)/Homer	1e-12	-2.891e+01	0.0000	5280.0	14.78%	4715.3	13.46%	motif file (matrix)	svg
246	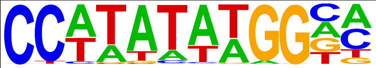	CArG(MADS)/PUER-Srf-ChIP-Seq(Sullivan_et_al.)/Homer	1e-12	-2.854e+01	0.0000	4640.0	12.99%	4116.2	11.75%	motif file (matrix)	svg
247	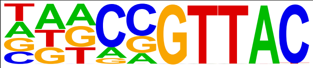	MYB98(MYB)/col-MYB98-DAP-Seq(GSE60143)/Homer	1e-12	-2.795e+01	0.0000	3542.0	9.92%	3094.6	8.83%	motif file (matrix)	svg
248	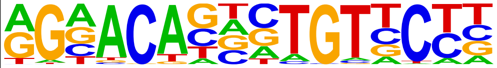	ARE(NR)/LNCAP-AR-ChIP-Seq(GSE27824)/Homer	1e-12	-2.792e+01	0.0000	3839.0	10.75%	3371.4	9.62%	motif file (matrix)	svg
249	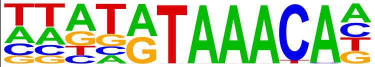	Foxf1(Forkhead)/Lung-Foxf1-ChIP-Seq(GSE77951)/Homer	1e-12	-2.773e+01	0.0000	11889.0	33.28%	11048.7	31.54%	motif file (matrix)	svg
250	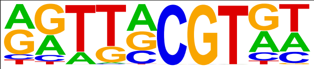	NAM(NAC)/col-NAM-DAP-Seq(GSE60143)/Homer	1e-12	-2.765e+01	0.0000	12691.0	35.53%	11825.5	33.76%	motif file (matrix)	svg
251	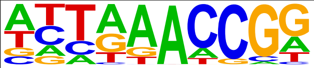	Unknown3/Arabidopsis-Promoters/Homer	1e-11	-2.731e+01	0.0000	2359.0	6.60%	2005.9	5.73%	motif file (matrix)	svg
252	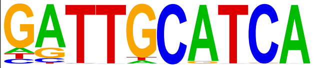	AARE(HLH)/mES-cMyc-ChIP-Seq/Homer	1e-11	-2.702e+01	0.0000	1244.0	3.48%	998.6	2.85%	motif file (matrix)	svg
253	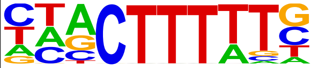	AT1G47655(C2C2dof)/colamp-AT1G47655-DAP-Seq(GSE60143)/Homer	1e-11	-2.700e+01	0.0000	30815.0	86.26%	29762.7	84.96%	motif file (matrix)	svg
254	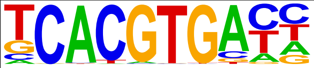	Cbf1(bHLH)/Yeast-Cbf1-ChIP-Seq(GSE29506)/Homer	1e-11	-2.650e+01	0.0000	4470.0	12.51%	3973.7	11.34%	motif file (matrix)	svg
255	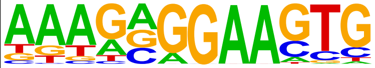	SpiB(ETS)/OCILY3-SPIB-ChIP-Seq(GSE56857)/Homer	1e-11	-2.619e+01	0.0000	3965.0	11.10%	3502.2	10.00%	motif file (matrix)	svg
256	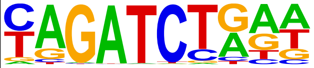	GATA12(C2C2gata)/col-GATA12-DAP-Seq(GSE60143)/Homer	1e-11	-2.608e+01	0.0000	7737.0	21.66%	7075.4	20.20%	motif file (matrix)	svg
257	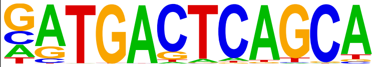	NF-E2(bZIP)/K562-NFE2-ChIP-Seq(GSE31477)/Homer	1e-11	-2.595e+01	0.0000	2252.0	6.30%	1915.6	5.47%	motif file (matrix)	svg
258	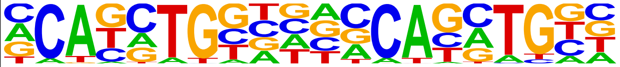	Twist(bHLH)/HMLE-TWIST1-ChIP-Seq(Chang_et_al)/Homer	1e-11	-2.581e+01	0.0000	2727.0	7.63%	2353.1	6.72%	motif file (matrix)	svg
259	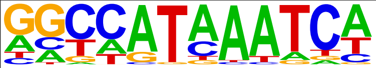	HOXA9(Homeobox)/HSC-Hoxa9-ChIP-Seq(GSE33509)/Homer	1e-11	-2.568e+01	0.0000	8874.0	24.84%	8168.5	23.32%	motif file (matrix)	svg
260	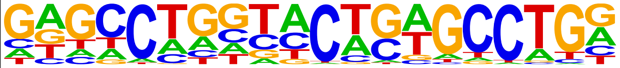	ZNF322(Zf)/HEK293-ZNF322.GFP-ChIP-Seq(GSE58341)/Homer	1e-11	-2.553e+01	0.0000	5952.0	16.66%	5381.2	15.36%	motif file (matrix)	svg
261	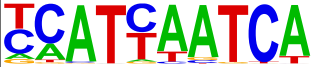	Pdx1(Homeobox)/Islet-Pdx1-ChIP-Seq(SRA008281)/Homer	1e-10	-2.498e+01	0.0000	12288.0	34.40%	11468.4	32.74%	motif file (matrix)	svg
262	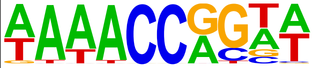	AT1G76870(Trihelix)/col-AT1G76870-DAP-Seq(GSE60143)/Homer	1e-10	-2.481e+01	0.0000	5641.0	15.79%	5093.5	14.54%	motif file (matrix)	svg
263		Lhx3(Homeobox)/Neuron-Lhx3-ChIP-Seq(GSE31456)/Homer	1e-10	-2.450e+01	0.0000	18012.0	50.42%	17052.9	48.68%	motif file	svg

	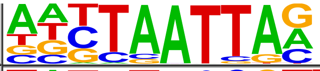									(matrix)	
264	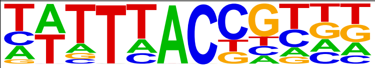	EMB1789(C3H)/col-EMB1789-DAP-Seq(GSE60143)/Homer	1e-10	-2.420e+01	0.0000	5224.0	14.62%	4704.9	13.43%	motif file (matrix)	svg
265	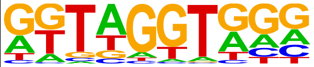	MYB3(MYB)/Arabidopsis-MYB3-ChIP-Seq(GSE80564)/Homer	1e-10	-2.385e+01	0.0000	21372.0	59.83%	20365.7	58.14%	motif file (matrix)	svg
266	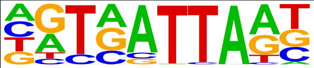	ATHB33(ZFHD)/col-ATHB33-DAP-Seq(GSE60143)/Homer	1e-10	-2.352e+01	0.0000	15243.0	42.67%	14359.9	40.99%	motif file (matrix)	svg
267	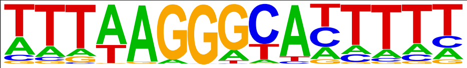	AT2G33550(Trihelix)/colamp-AT2G33550-DAP-Seq(GSE60143)/Homer	1e-10	-2.346e+01	0.0000	22274.0	62.35%	21262.0	60.70%	motif file (matrix)	svg
268	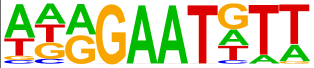	AT2G38300(G2like)/col-AT2G38300-DAP-Seq(GSE60143)/Homer	1e-10	-2.338e+01	0.0000	17549.0	49.13%	16614.6	47.43%	motif file (matrix)	svg
269	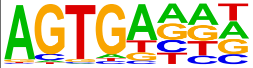	At5g04390(C2H2)/col200-At5g04390-DAP-Seq(GSE60143)/Homer	1e-10	-2.305e+01	0.0000	35120.0	98.31%	34273.7	97.84%	motif file (matrix)	svg
270	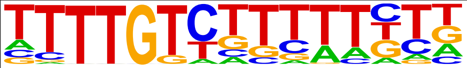	IDD5(C2H2)/colamp-IDD5-DAP-Seq(GSE60143)/Homer	1e-9	-2.291e+01	0.0000	8955.0	25.07%	8278.8	23.63%	motif file (matrix)	svg
271	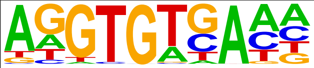	Tbet(T-box)/CD8-Tbet-ChIP-Seq(GSE33802)/Homer	1e-9	-2.283e+01	0.0000	14005.0	39.20%	13163.7	37.58%	motif file (matrix)	svg
272	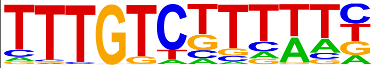	IDD4(C2H2)/col-IDD4-DAP-Seq(GSE60143)/Homer	1e-9	-2.268e+01	0.0000	10901.0	30.52%	10156.4	28.99%	motif file (matrix)	svg
273	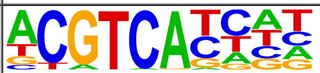	TGA2(bZIP)/colamp-TGA2-DAP-Seq(GSE60143)/Homer	1e-9	-2.267e+01	0.0000	7063.0	19.77%	6468.1	18.46%	motif file (matrix)	svg
274	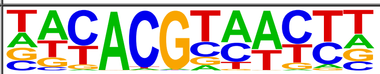	ANAC047(NAC)/colamp-ANAC047-DAP-Seq(GSE60143)/Homer	1e-9	-2.243e+01	0.0000	8176.0	22.89%	7536.6	21.51%	motif file (matrix)	svg
275	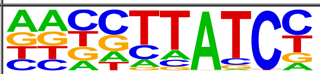	At5g08520(MYBrelated)/colamp-At5g08520-DAP-Seq(GSE60143)/Homer	1e-9	-2.242e+01	0.0000	13040.0	36.50%	12231.6	34.92%	motif file (matrix)	svg
276	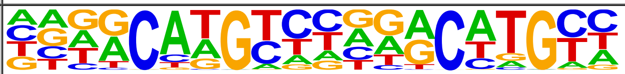	p63(p53)/Keratinocyte-p63-ChIP-Seq(GSE17611)/Homer	1e-9	-2.240e+01	0.0000	5096.0	14.27%	4600.4	13.13%	motif file (matrix)	svg
277	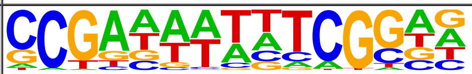	CDM1(C3H)/colamp-CDM1-DAP-Seq(GSE60143)/Homer	1e-9	-2.232e+01	0.0000	1091.0	3.05%	882.6	2.52%	motif file (matrix)	svg
278	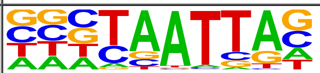	DLX2(Homeobox)/BasalGanglia-Dlx2-ChIP-seq(GSE124936)/Homer	1e-9	-2.205e+01	0.0000	16758.0	46.91%	15859.3	45.27%	motif file (matrix)	svg
279	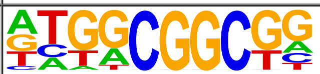	ERF11(AP2EREBP)/col-ERF11-DAP-Seq(GSE60143)/Homer	1e-9	-2.204e+01	0.0000	8020.0	22.45%	7391.1	21.10%	motif file (matrix)	svg
280	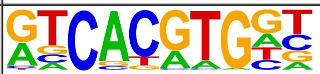	Usf2(bHLH)/C2C12-Usf2-ChIP-Seq(GSE36030)/Homer	1e-9	-2.196e+01	0.0000	5427.0	15.19%	4918.1	14.04%	motif file (matrix)	svg
281	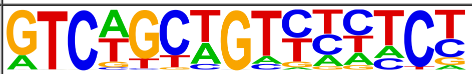	ZNF317(Zf)/HEK293-ZNF317.GFP-ChIP-Seq(GSE58341)/Homer	1e-9	-2.181e+01	0.0000	1670.0	4.67%	1407.9	4.02%	motif file (matrix)	svg
282	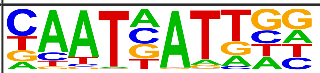	ATHB6(Homeobox)/Arabidopsis-HB6-ChIP-Seq(GSE80564)/Homer	1e-9	-2.179e+01	0.0000	8189.0	22.92%	7556.4	21.57%	motif file (matrix)	svg
283	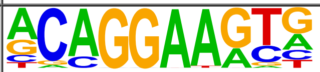	ERG(ETS)/VCaP-ERG-ChIP-Seq(GSE14097)/Homer	1e-9	-2.171e+01	0.0000	23997.0	67.18%	22991.8	65.63%	motif file (matrix)	svg

284	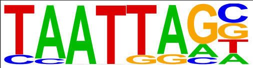	Lhx2(Homeobox)/HFSC-Lhx2-ChIP-Seq(GSE48068)/Homer	1e-9	-2.121e+01	0.0000	12260.0	34.32%	11491.3	32.80%	motif file (matrix)	svg
285	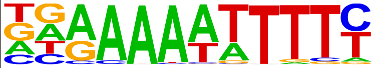	SFP1/SacCer-Promoters/Homer	1e-9	-2.114e+01	0.0000	1468.0	4.11%	1227.8	3.51%	motif file (matrix)	svg
286	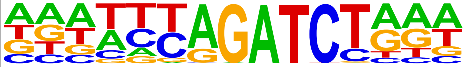	GATA1(C2C2gata)/colamp-GATA1-DAP-Seq(GSE60143)/Homer	1e-9	-2.112e+01	0.0000	4897.0	13.71%	4424.1	12.63%	motif file (matrix)	svg
287	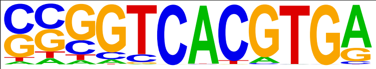	E-box(bHLH)/Promoter/Homer	1e-9	-2.102e+01	0.0000	1475.0	4.13%	1234.6	3.52%	motif file (matrix)	svg
288	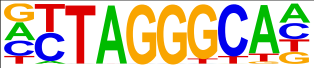	TBP3(MYBrelated)/col-TBP3-DAP-Seq(GSE60143)/Homer	1e-9	-2.099e+01	0.0000	10673.0	29.88%	9958.4	28.43%	motif file (matrix)	svg
289	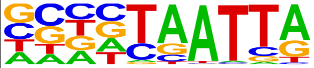	DLX1(Homeobox)/BasalGanglia-Dlx1-ChIP-seq(GSE124936)/Homer	1e-9	-2.097e+01	0.0000	15210.0	42.58%	14363.7	41.00%	motif file (matrix)	svg
290	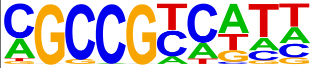	ERF8(AP2EREBP)/colamp-ERF8-DAP-Seq(GSE60143)/Homer	1e-9	-2.094e+01	0.0000	9610.0	26.90%	8933.4	25.50%	motif file (matrix)	svg
291	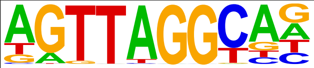	MYB116(MYB)/colamp-MYB116-DAP-Seq(GSE60143)/Homer	1e-9	-2.086e+01	0.0000	8599.0	24.07%	7961.6	22.73%	motif file (matrix)	svg
292	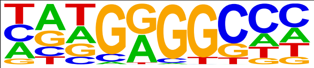	Zac1(Zf)/Neuro2A-Plagl1-ChIP-Seq(GSE75942)/Homer	1e-9	-2.076e+01	0.0000	30735.0	86.04%	29744.1	84.91%	motif file (matrix)	svg
293	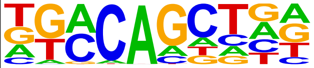	bZIP18(bZIP)/colamp-bZIP18-DAP-Seq(GSE60143)/Homer	1e-8	-2.063e+01	0.0000	35092.0	98.23%	34253.7	97.78%	motif file (matrix)	svg
294	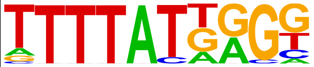	HOXB13(Homeobox)/ProstateTumor-HOXB13-ChIP-Seq(GSE56288)/Homer	1e-8	-2.061e+01	0.0000	13155.0	36.83%	12368.7	35.31%	motif file (matrix)	svg
295	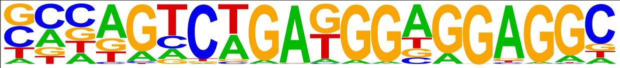	ZSCAN22(Zf)/HEK293-ZSCAN22.GFP-ChIP-Seq(GSE58341)/Homer	1e-8	-2.061e+01	0.0000	1566.0	4.38%	1319.5	3.77%	motif file (matrix)	svg
296	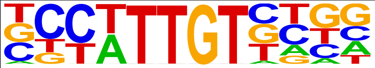	Sox21(HMG)/ESC-SOX21-ChIP-Seq(GSE110505)/Homer	1e-8	-2.061e+01	0.0000	20386.0	57.07%	19440.5	55.50%	motif file (matrix)	svg
297	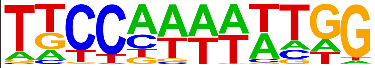	AGL63(MADS)/col-AGL63-DAP-Seq(GSE60143)/Homer	1e-8	-2.054e+01	0.0000	10585.0	29.63%	9879.6	28.20%	motif file (matrix)	svg
298	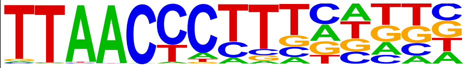	ZNF652/HepG2-ZNF652.Flag-ChIP-Seq(Encode)/Homer	1e-8	-2.042e+01	0.0000	3888.0	10.88%	3478.1	9.93%	motif file (matrix)	svg
299	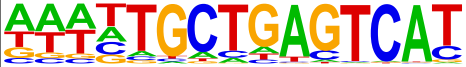	NFE2L2(bZIP)/HepG2-NFE2L2-ChIP-Seq(Encode)/Homer	1e-8	-2.015e+01	0.0000	1653.0	4.63%	1401.5	4.00%	motif file (matrix)	svg
300	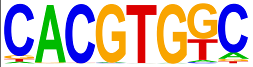	ABF1(bZIP)/Arabidopsis-ABF1-ChIP-Seq(GSE80564)/Homer	1e-8	-1.979e+01	0.0000	12734.0	35.65%	11971.1	34.17%	motif file (matrix)	svg
301	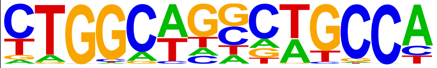	Tlx?(NR)/NPC-H3K4me1-ChIP-Seq(GSE16256)/Homer	1e-8	-1.977e+01	0.0000	8312.0	23.27%	7699.6	21.98%	motif file (matrix)	svg
302	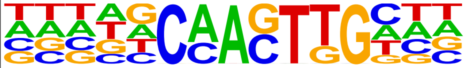	bHLH80(bHLH)/col-bHLH80-DAP-Seq(GSE60143)/Homer	1e-8	-1.968e+01	0.0000	13680.0	38.29%	12892.2	36.80%	motif file (matrix)	svg
303	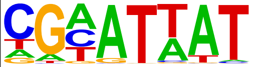	AtHB32(ZFHD)/col200-AtHB32-DAP-Seq(GSE60143)/Homer	1e-8	-1.966e+01	0.0000	13472.0	37.71%	12690.8	36.23%	motif file (matrix)	svg
304		MYB105(MYB)/colamp-MYB105-DAP-Seq(GSE60143)/Homer	1e-8	-1.963e+01	0.0000	7656.0	21.43%	7071.4	20.19%	motif file	svg

	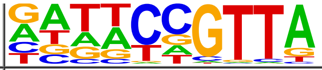									(matrix)	
305	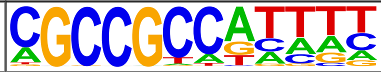	RAP212(AP2EREBP)/col-RAP212-DAP-Seq(GSE60143)/Homer	1e-8	-1.938e+01	0.0000	7016.0	19.64%	6461.2	18.44%	motif file (matrix)	svg
306	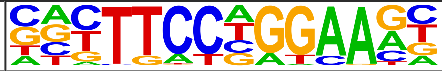	Stat3+il21(Stat)/CD4-Stat3-ChIP-Seq(GSE19198)/Homer	1e-8	-1.908e+01	0.0000	12276.0	34.36%	11537.0	32.93%	motif file (matrix)	svg
307	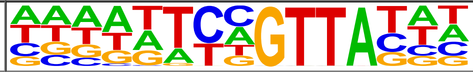	MYB113(MYB)/col-MYB113-DAP-Seq(GSE60143)/Homer	1e-8	-1.893e+01	0.0000	6816.0	19.08%	6275.3	17.91%	motif file (matrix)	svg
308	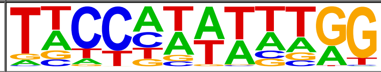	SOC1(MADS)/Seedling-SOC1-ChIP-Seq(GSE45846)/Homer	1e-8	-1.871e+01	0.0000	5469.0	15.31%	4992.7	14.25%	motif file (matrix)	svg
309	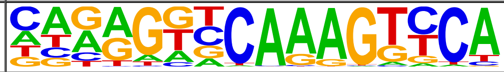	HNF4a(NR),DR1/HepG2-HNF4a-ChIP-Seq(GSE25021)/Homer	1e-8	-1.868e+01	0.0000	7679.0	21.50%	7105.6	20.28%	motif file (matrix)	svg
310	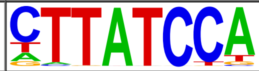	AT5G61620(MYBrelated)/colamp-AT5G61620-DAP-Seq(GSE60143)/Homer	1e-8	-1.860e+01	0.0000	16679.0	46.69%	15834.5	45.20%	motif file (matrix)	svg
311	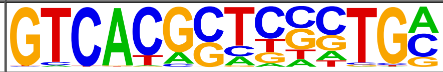	PAX5(Paired,Homeobox),condensed/GM12878-PAX5-ChIP-Seq(GSE32465)/Homer	1e-8	-1.851e+01	0.0000	1761.0	4.93%	1510.1	4.31%	motif file (matrix)	svg
312	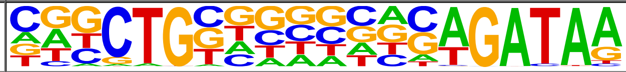	GATA:SCL(Zf,bHLH)/Ter119-SCL-ChIP-Seq(GSE18720)/Homer	1e-8	-1.846e+01	0.0000	1753.0	4.91%	1503.8	4.29%	motif file (matrix)	svg
313	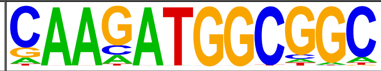	YY1(Zf)/Promoter/Homer	1e-7	-1.830e+01	0.0000	904.0	2.53%	733.2	2.09%	motif file (matrix)	svg
314	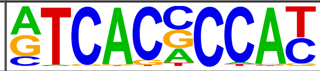	Srebp1a(bHLH)/HepG2-Srebp1a-ChIP-Seq(GSE31477)/Homer	1e-7	-1.829e+01	0.0000	3740.0	10.47%	3358.1	9.59%	motif file (matrix)	svg
315	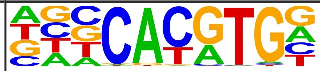	PIF4(bHLH)/Seedling-PIF4-ChIP-Seq(GSE35315)/Homer	1e-7	-1.820e+01	0.0000	17205.0	48.16%	16355.7	46.69%	motif file (matrix)	svg
316	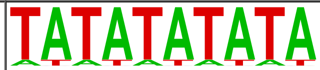	SeqBias: TA-repeat	1e-7	-1.803e+01	0.0000	24568.0	68.77%	23611.5	67.40%	motif file (matrix)	svg
317	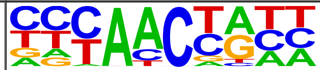	ATY13(MYB)/col-ATY13-DAP-Seq(GSE60143)/Homer	1e-7	-1.743e+01	0.0000	30525.0	85.45%	29570.8	84.41%	motif file (matrix)	svg
318	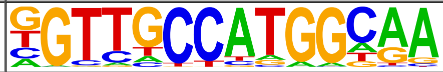	Rfx1(HTH)/NPC-H3K4me1-ChIP-Seq(GSE16256)/Homer	1e-7	-1.726e+01	0.0000	3220.0	9.01%	2878.6	8.22%	motif file (matrix)	svg
319	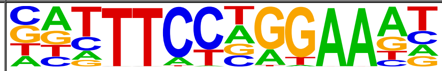	STAT1(Stat)/HelaS3-STAT1-ChIP-Seq(GSE12782)/Homer	1e-7	-1.716e+01	0.0000	4482.0	12.55%	4071.1	11.62%	motif file (matrix)	svg
320	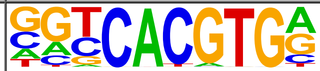	USF1(bHLH)/GM12878-Usf1-ChIP-Seq(GSE32465)/Homer	1e-7	-1.710e+01	0.0000	7873.0	22.04%	7312.4	20.87%	motif file (matrix)	svg
321	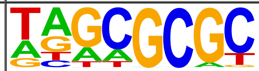	DPL-1(E2F)/cElegans-Adult-ChIP-Seq(modEncode)/Homer	1e-7	-1.706e+01	0.0000	7058.0	19.76%	6530.9	18.64%	motif file (matrix)	svg
322	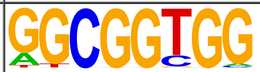	AT3G57600(AP2EREBP)/col-AT3G57600-DAP-Seq(GSE60143)/Homer	1e-7	-1.675e+01	0.0000	9483.0	26.55%	8868.2	25.32%	motif file (matrix)	svg
323	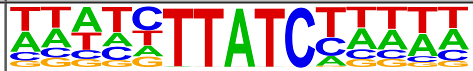	At5g58900(MYBrelated)/colamp-At5g58900-DAP-Seq(GSE60143)/Homer	1e-7	-1.671e+01	0.0000	16173.0	45.27%	15370.7	43.88%	motif file (matrix)	svg
324	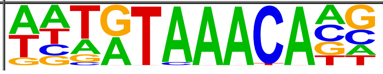	FoxL2(Forkhead)/Ovary-FoxL2-ChIP-Seq(GSE60858)/Homer	1e-7	-1.656e+01	0.0000	10873.0	30.44%	10215.2	29.16%	motif file	svg

										(matrix)	
325	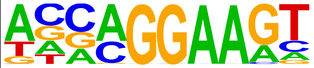	EHF(ETS)/LoVo-EHF-ChIP-Seq(GSE49402)/Homer	1e-7	-1.639e+01	0.0000	20595.0	57.65%	19713.3	56.28%	motif file (matrix)	svg
326	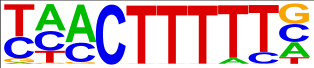	AT3G12130(C3H)/colamp-AT3G12130-DAP-Seq(GSE60143)/Homer	1e-7	-1.635e+01	0.0000	26675.0	74.67%	25730.5	73.45%	motif file (matrix)	svg
327	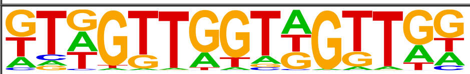	AT1G24250(Orphan)/col-AT1G24250-DAP-Seq(GSE60143)/Homer	1e-7	-1.621e+01	0.0000	4308.0	12.06%	3916.7	11.18%	motif file (matrix)	svg
328	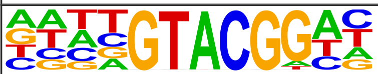	bHLH28(bHLH)/col-bHLH28-DAP-Seq(GSE60143)/Homer	1e-7	-1.614e+01	0.0000	2405.0	6.73%	2124.2	6.06%	motif file (matrix)	svg
329	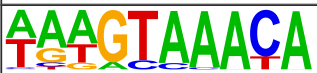	FOXA1(Forkhead)/LNCAP-FOXA1-ChIP-Seq(GSE27824)/Homer	1e-6	-1.602e+01	0.0000	14361.0	40.20%	13614.0	38.86%	motif file (matrix)	svg
330	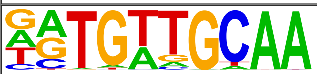	CEBP:AP1(bZIP)/ThioMac-CEBPb-ChIP-Seq(GSE21512)/Homer	1e-6	-1.599e+01	0.0000	9845.0	27.56%	9229.3	26.35%	motif file (matrix)	svg
331	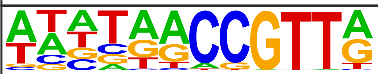	MYB3R4(MYB)/col-MYB3R4-DAP-Seq(GSE60143)/Homer	1e-6	-1.593e+01	0.0000	1776.0	4.97%	1541.8	4.40%	motif file (matrix)	svg
332	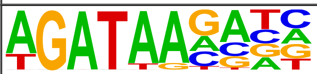	TRPS1(Zf)/MCF7-TRPS1-ChIP-Seq(GSE107013)/Homer	1e-6	-1.580e+01	0.0000	21188.0	59.31%	20307.8	57.97%	motif file (matrix)	svg
333	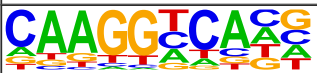	SF1(NR)/H295R-Nr5a1-ChIP-Seq(GSE44220)/Homer	1e-6	-1.579e+01	0.0000	7381.0	20.66%	6857.1	19.57%	motif file (matrix)	svg
334	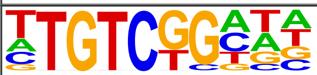	ARF2(ARF)/col-ARF2-DAP-Seq(GSE60143)/Homer	1e-6	-1.569e+01	0.0000	29573.0	82.78%	28635.3	81.74%	motif file (matrix)	svg
335	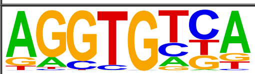	Tbx5(T-box)/HL1-Tbx5.biotin-ChIP-Seq(GSE21529)/Homer	1e-6	-1.568e+01	0.0000	30831.0	86.31%	29900.1	85.36%	motif file (matrix)	svg
336	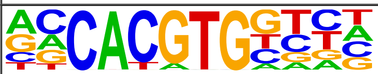	Max(bHLH)/K562-Max-ChIP-Seq(GSE31477)/Homer	1e-6	-1.565e+01	0.0000	10559.0	29.56%	9925.2	28.33%	motif file (matrix)	svg
337	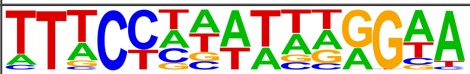	AGL15(MADS)/col-AGL15-DAP-Seq(GSE60143)/Homer	1e-6	-1.563e+01	0.0000	2198.0	6.15%	1935.5	5.53%	motif file (matrix)	svg
338	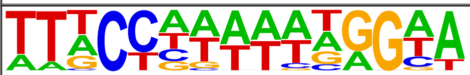	AGL6(MADS)/col-AGL6-DAP-Seq(GSE60143)/Homer	1e-6	-1.559e+01	0.0000	1688.0	4.73%	1462.1	4.17%	motif file (matrix)	svg
339	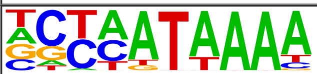	HOXD13(Homeobox)/Chicken-Hoxd13-ChIP-Seq(GSE38910)/Homer	1e-6	-1.547e+01	0.0000	12746.0	35.68%	12050.9	34.40%	motif file (matrix)	svg
340	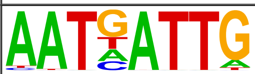	ATHB5(HB)/colamp-ATHB5-DAP-Seq(GSE60143)/Homer	1e-6	-1.539e+01	0.0000	10093.0	28.25%	9478.0	27.06%	motif file (matrix)	svg
341	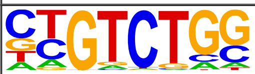	Smad2(MAD)/ES-SMAD2-ChIP-Seq(GSE29422)/Homer	1e-6	-1.534e+01	0.0000	22395.0	62.69%	21505.8	61.39%	motif file (matrix)	svg
342	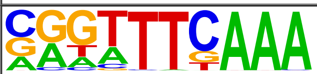	CHR(?)/Hela-CellCycle-Expression/Homer	1e-6	-1.496e+01	0.0000	8724.0	24.42%	8162.8	23.30%	motif file (matrix)	svg
343	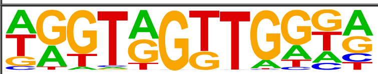	MYB94(MYB)/col-MYB94-DAP-Seq(GSE60143)/Homer	1e-6	-1.488e+01	0.0000	6275.0	17.57%	5808.3	16.58%	motif file (matrix)	svg
344	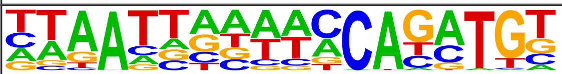	Pitx1:Ebox(Homeobox,bHLH)/Hindlimb-Pitx1-ChIP-Seq(GSE41591)/Homer	1e-6	-1.483e+01	0.0000	2822.0	7.90%	2526.9	7.21%	motif file (matrix)	svg
345		NPAS(bHLH)/Liver-NPAS-ChIP-	1e-6	-1.481e+01	0.0000	20430.0	57.19%	19578.5	55.89%	motif	svg

	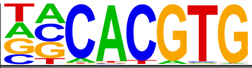	Seq(GSE39860)/Homer								file (matrix)	
346	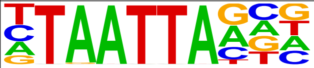	ATHB23(ZFHD)/col-ATHB23-DAP-Seq(GSE60143)/Homer	1e-6	-1.465e+01	0.0000	10815.0	30.27%	10189.8	29.09%	motif file (matrix)	svg
347	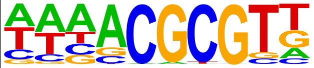	CAMTA1(CAMTA)/col-CAMTA1-DAP-Seq(GSE60143)/Homer	1e-6	-1.459e+01	0.0000	4973.0	13.92%	4568.0	13.04%	motif file (matrix)	svg
348	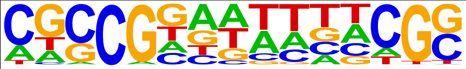	LOB(LOBAS2)/col-LOB-DAP-Seq(GSE60143)/Homer	1e-6	-1.446e+01	0.0000	2405.0	6.73%	2138.4	6.10%	motif file (matrix)	svg
349	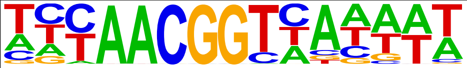	MYB3R5(MYB)/col-MYB3R5-DAP-Seq(GSE60143)/Homer	1e-6	-1.445e+01	0.0000	847.0	2.37%	700.9	2.00%	motif file (matrix)	svg
350	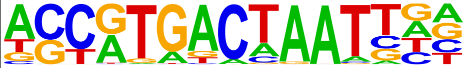	PAX3:FKHR-fusion(Paired,Homeobox)/Rh4-PAX3:FKHR-ChIP-Seq(GSE19063)/Homer	1e-6	-1.444e+01	0.0000	2519.0	7.05%	2245.1	6.41%	motif file (matrix)	svg
351	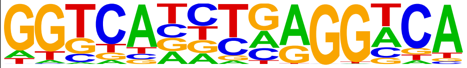	THRa(NR)/C17.2-THRa-ChIP-Seq(GSE38347)/Homer	1e-6	-1.444e+01	0.0000	7487.0	20.96%	6978.7	19.92%	motif file (matrix)	svg
352	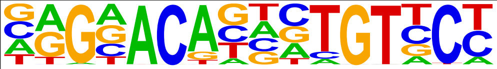	GRE(NR),IR3/A549-GR-ChIP-Seq(GSE32465)/Homer	1e-6	-1.443e+01	0.0000	2014.0	5.64%	1773.4	5.06%	motif file (matrix)	svg
353	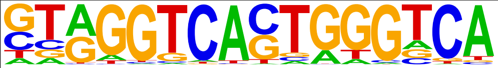	Reverb(NR),DR2/RAW-Reverba.biotin-ChIP-Seq(GSE45914)/Homer	1e-6	-1.419e+01	0.0000	2620.0	7.33%	2342.0	6.69%	motif file (matrix)	svg
354	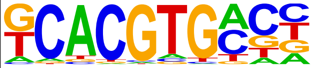	bHLHE41(bHLH)/proB-Bhlhe41-ChIP-Seq(GSE93764)/Homer	1e-6	-1.414e+01	0.0000	15597.0	43.66%	14852.5	42.40%	motif file (matrix)	svg
355	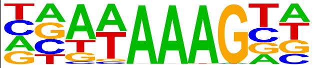	dof45(C2C2dof)/col-dof45-DAP-Seq(GSE60143)/Homer	1e-6	-1.412e+01	0.0000	26530.0	74.27%	25621.1	73.14%	motif file (matrix)	svg
356	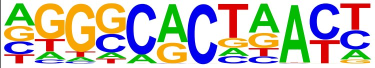	ZNF264(Zf)/HEK293-ZNF264.GFP-ChIP-Seq(GSE58341)/Homer	1e-6	-1.407e+01	0.0000	9697.0	27.14%	9116.5	26.02%	motif file (matrix)	svg
357	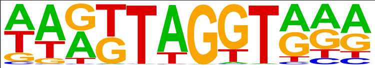	MS188(MYB)/colamp-MS188-DAP-Seq(GSE60143)/Homer	1e-6	-1.403e+01	0.0000	8849.0	24.77%	8297.6	23.69%	motif file (matrix)	svg
358	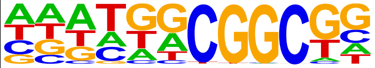	RAP26(AP2EREBP)/colamp-RAP26-DAP-Seq(GSE60143)/Homer	1e-6	-1.400e+01	0.0000	10516.0	29.44%	9910.2	28.29%	motif file (matrix)	svg
359	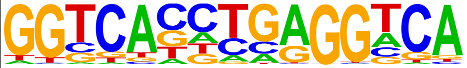	THRb(NR)/HepG2-THRb.Flag-ChIP-Seq(Encode)/Homer	1e-6	-1.400e+01	0.0000	10615.0	29.71%	10006.2	28.56%	motif file (matrix)	svg
360	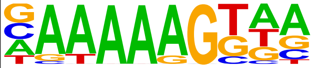	AT2G28810(C2C2dof)/colamp-AT2G28810-DAP-Seq(GSE60143)/Homer	1e-6	-1.399e+01	0.0000	25964.0	72.68%	25061.3	71.54%	motif file (matrix)	svg
361	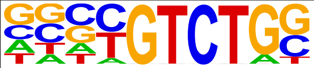	Smad4(MAD)/ESC-SMAD4-ChIP-Seq(GSE29422)/Homer	1e-6	-1.395e+01	0.0000	22447.0	62.84%	21582.0	61.61%	motif file (matrix)	svg
362	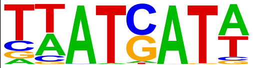	CRC(C2C2YABBY)/col-CRC-DAP-Seq(GSE60143)/Homer	1e-6	-1.387e+01	0.0000	12945.0	36.24%	12271.1	35.03%	motif file (matrix)	svg
363	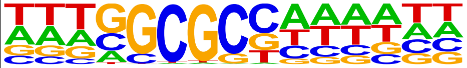	E2FA(E2FDP)/colamp-E2FA-DAP-Seq(GSE60143)/Homer	1e-5	-1.331e+01	0.0000	4993.0	13.98%	4602.9	13.14%	motif file (matrix)	svg
364	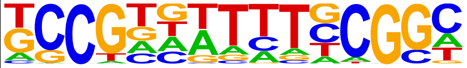	LBD13(LOBAS2)/colamp-LBD13-DAP-Seq(GSE60143)/Homer	1e-5	-1.331e+01	0.0000	9932.0	27.80%	9356.9	26.71%	motif file (matrix)	svg
365	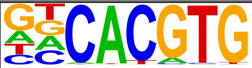	BMAL1(bHLH)/Liver-Bmal1-ChIP-Seq(GSE39860)/Homer	1e-5	-1.318e+01	0.0000	22090.0	61.84%	21243.5	60.64%	motif file (matrix)	svg

366	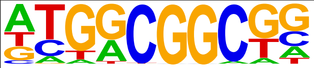	ERF3(AP2EREBP)/colamp-ERF3-DAP-Seq(GSE60143)/Homer	1e-5	-1.310e+01	0.0000	6669.0	18.67%	6211.6	17.73%	motif file (matrix)	svg
367	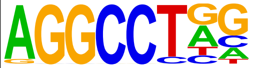	ZFX(Zf)/mES-Zfx-ChIP-Seq(GSE11431)/Homer	1e-5	-1.309e+01	0.0000	20489.0	57.36%	19668.3	56.15%	motif file (matrix)	svg
368	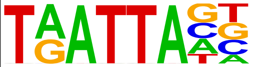	ATHB25(ZFHD)/colamp-ATHB25-DAP-Seq(GSE60143)/Homer	1e-5	-1.307e+01	0.0000	13079.0	36.61%	12417.0	35.45%	motif file (matrix)	svg
369	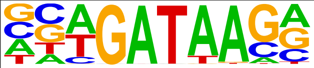	Gata4(Zf)/Heart-Gata4-ChIP-Seq(GSE35151)/Homer	1e-5	-1.297e+01	0.0000	12696.0	35.54%	12045.6	34.39%	motif file (matrix)	svg
370	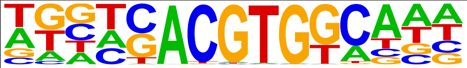	GBF5(bZIP)/colamp-GBF5-DAP-Seq(GSE60143)/Homer	1e-5	-1.291e+01	0.0000	4775.0	13.37%	4399.5	12.56%	motif file (matrix)	svg
371	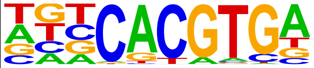	SPCH(bHLH)/Seedling-SPCH-ChIP-Seq(GSE57497)/Homer	1e-5	-1.282e+01	0.0000	14942.0	41.83%	14237.6	40.64%	motif file (matrix)	svg
372	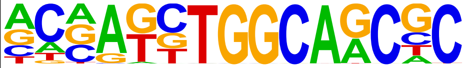	Unknown3/Drosophila-Promoters/Homer	1e-5	-1.273e+01	0.0000	1546.0	4.33%	1351.8	3.86%	motif file (matrix)	svg
373	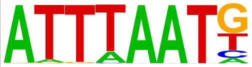	At1g76110(ARID)/colamp-At1g76110-DAP-Seq(GSE60143)/Homer	1e-5	-1.250e+01	0.0000	7551.0	21.14%	7069.6	20.18%	motif file (matrix)	svg
374	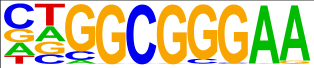	E2F1(E2F)/Hela-E2F1-ChIP-Seq(GSE22478)/Homer	1e-5	-1.248e+01	0.0000	3352.0	9.38%	3050.2	8.71%	motif file (matrix)	svg
375	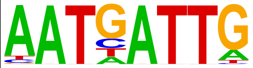	ATHB6(Homeobox)/col-ATHB6-DAP-Seq(GSE60143)/Homer	1e-5	-1.245e+01	0.0000	13026.0	36.46%	12376.5	35.33%	motif file (matrix)	svg
376	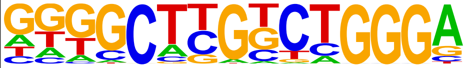	Zfp809(Zf)/ES-Zfp809-ChIP-Seq(GSE70799)/Homer	1e-5	-1.244e+01	0.0000	4889.0	13.69%	4514.7	12.89%	motif file (matrix)	svg
377	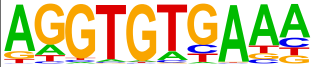	Tbx21(T-box)/GM12878-TBX21-ChIP-Seq(Encode)/Homer	1e-5	-1.243e+01	0.0000	13537.0	37.89%	12874.5	36.75%	motif file (matrix)	svg
378	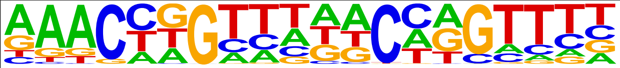	GRHL2(CP2)/HBE-GRHL2-ChIP-Seq(GSE46194)/Homer	1e-5	-1.237e+01	0.0000	5114.0	14.32%	4730.3	13.50%	motif file (matrix)	svg
379	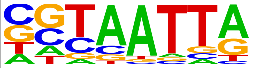	DLX5(Homeobox)/BasalGanglia-Dlx5-ChIP-seq(GSE124936)/Homer	1e-5	-1.235e+01	0.0000	9257.0	25.91%	8719.8	24.89%	motif file (matrix)	svg
380	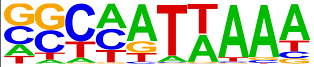	Unknown(Homeobox)/Limb-p300-ChIP-Seq/Homer	1e-5	-1.235e+01	0.0000	8446.0	23.64%	7935.9	22.65%	motif file (matrix)	svg
381	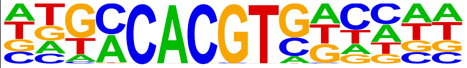	AREB3(bZIP)/col-AREB3-DAP-Seq(GSE60143)/Homer	1e-5	-1.234e+01	0.0000	4783.0	13.39%	4414.9	12.60%	motif file (matrix)	svg
382	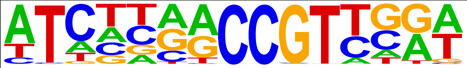	ZML1(C2C2gata)/colamp-ZML1-DAP-Seq(GSE60143)/Homer	1e-5	-1.223e+01	0.0000	155.0	0.43%	104.4	0.30%	motif file (matrix)	svg
383	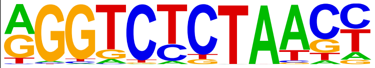	PRDM14(Zf)/H1-PRDM14-ChIP-Seq(GSE22767)/Homer	1e-5	-1.222e+01	0.0000	4573.0	12.80%	4215.0	12.03%	motif file (matrix)	svg
384	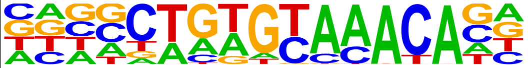	Fox:Ebox(Forkhead,bHLH)/Panc1-Foxa2-ChIP-Seq(GSE47459)/Homer	1e-5	-1.222e+01	0.0000	13740.0	38.46%	13076.6	37.33%	motif file (matrix)	svg
385	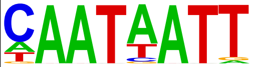	ATHB13(Homeobox)/col-ATHB13-DAP-Seq(GSE60143)/Homer	1e-5	-1.210e+01	0.0000	12048.0	33.73%	11432.0	32.63%	motif file (matrix)	svg
386		bZIP3(bZIP)/col-bZIP3-DAP-Seq(GSE60143)/Homer	1e-5	-1.181e+01	0.0000	7439.0	20.82%	6972.6	19.90%	motif file	svg

	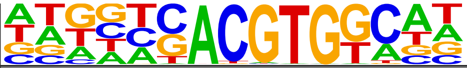									(matrix)	
387	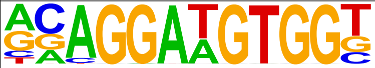	ETS:RUNX(ETS,Runt)/Jurkat-RUNX1-ChIP-Seq(GSE17954)/Homer	1e-5	-1.162e+01	0.0000	1804.0	5.05%	1600.3	4.57%	motif file (matrix)	svg
388	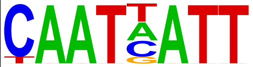	ATHB20(Homeobox)/colamp-ATHB20-DAP-Seq(GSE60143)/Homer	1e-5	-1.161e+01	0.0000	4708.0	13.18%	4352.8	12.43%	motif file (matrix)	svg
389	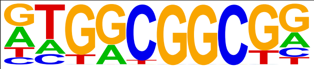	ERF10(AP2EREBP)/col-ERF10-DAP-Seq(GSE60143)/Homer	1e-5	-1.161e+01	0.0000	6817.0	19.08%	6376.4	18.20%	motif file (matrix)	svg
390	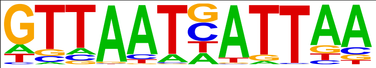	HNF1b(Homeobox)/PDAC-HNF1B-ChIP-Seq(GSE64557)/Homer	1e-5	-1.151e+01	0.0000	1818.0	5.09%	1614.5	4.61%	motif file (matrix)	svg
391	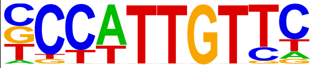	Sox2(HMG)/mES-Sox2-ChIP-Seq(GSE11431)/Homer	1e-4	-1.149e+01	0.0000	11107.0	31.09%	10528.7	30.06%	motif file (matrix)	svg
392	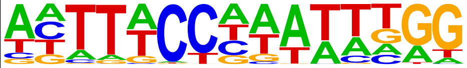	SVP(MADS)/col-SVP-DAP-Seq(GSE60143)/Homer	1e-4	-1.139e+01	0.0000	7102.0	19.88%	6654.3	19.00%	motif file (matrix)	svg
393	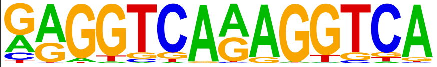	TR4(NR),DR1/Hela-TR4-ChIP-Seq(GSE24685)/Homer	1e-4	-1.137e+01	0.0000	2060.0	5.77%	1842.5	5.26%	motif file (matrix)	svg
394	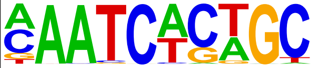	Gfi1b(Zf)/HPC7-Gfi1b-ChIP-Seq(GSE22178)/Homer	1e-4	-1.130e+01	0.0000	9333.0	26.13%	8811.1	25.15%	motif file (matrix)	svg
395	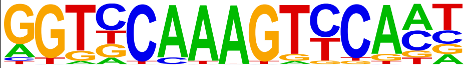	dHNF4(NR)/Fly-HNF4-ChIP-Seq(GSE73675)/Homer	1e-4	-1.121e+01	0.0000	626.0	1.75%	517.8	1.48%	motif file (matrix)	svg
396	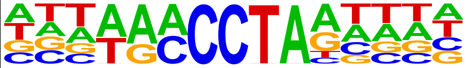	AT1G72740(MYBrelated)/colamp-AT1G72740-DAP-Seq(GSE60143)/Homer	1e-4	-1.120e+01	0.0000	13230.0	37.03%	12599.2	35.97%	motif file (matrix)	svg
397	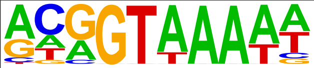	AT1G76880(Trihelix)/col-AT1G76880-DAP-Seq(GSE60143)/Homer	1e-4	-1.112e+01	0.0000	4540.0	12.71%	4198.9	11.99%	motif file (matrix)	svg
398	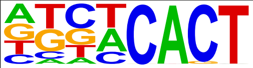	AZF1(C2H2)/colamp-AZF1-DAP-Seq(GSE60143)/Homer	1e-4	-1.106e+01	0.0000	35051.0	98.12%	34260.4	97.80%	motif file (matrix)	svg
399	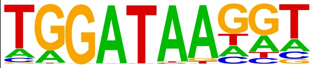	AT5G56840(MYBrelated)/colamp-AT5G56840-DAP-Seq(GSE60143)/Homer	1e-4	-1.105e+01	0.0000	14780.0	41.37%	14114.5	40.29%	motif file (matrix)	svg
400	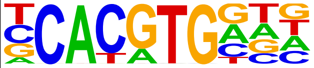	PIF5ox(bHLH)/Arabidopsis-PIF5ox-ChIP-Seq(GSE35062)/Homer	1e-4	-1.096e+01	0.0000	14995.0	41.98%	14326.4	40.90%	motif file (matrix)	svg
401	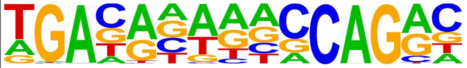	Hand2(bHLH)/Mesoderm-Hand2-ChIP-Seq(GSE61475)/Homer	1e-4	-1.094e+01	0.0000	8726.0	24.43%	8230.6	23.50%	motif file (matrix)	svg
402	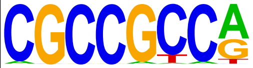	CRF4(AP2EREBP)/colamp-CRF4-DAP-Seq(GSE60143)/Homer	1e-4	-1.088e+01	0.0000	5378.0	15.05%	5004.7	14.29%	motif file (matrix)	svg
403	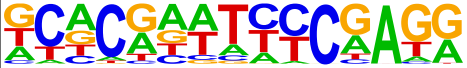	AIL7(AP2EREBP)/colamp-AIL7-DAP-Seq(GSE60143)/Homer	1e-4	-1.082e+01	0.0000	6268.0	17.55%	5861.0	16.73%	motif file (matrix)	svg
404	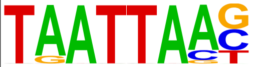	ATHB24(ZFHD)/colamp-ATHB24-DAP-Seq(GSE60143)/Homer	1e-4	-1.082e+01	0.0000	8916.0	24.96%	8416.3	24.03%	motif file (matrix)	svg
405	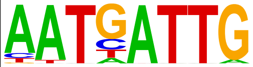	ATHB7(Homeobox)/col-ATHB7-DAP-Seq(GSE60143)/Homer	1e-4	-1.078e+01	0.0001	7526.0	21.07%	7073.3	20.19%	motif file (matrix)	svg
406	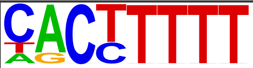	At1g64620(C2C2dof)/colamp-At1g64620-DAP-Seq(GSE60143)/Homer	1e-4	-1.072e+01	0.0001	19218.0	53.80%	18467.5	52.72%	motif file (matrix)	svg

407	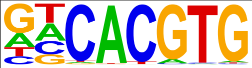	CLOCK(bHLH)/Liver-Clock-ChIP-Seq(GSE39860)/Homer	1e-4	-1.068e+01	0.0001	8994.0	25.18%	8494.0	24.25%	motif file (matrix)	svg
408	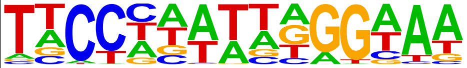	AGL16(MADS)/col-AGL16-DAP-Seq(GSE60143)/Homer	1e-4	-1.054e+01	0.0001	1459.0	4.08%	1287.0	3.67%	motif file (matrix)	svg
409	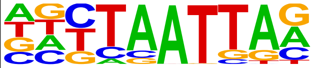	Lhx1(Homeobox)/EmbryoCarcinoma-Lhx1-ChIP-Seq(GSE70957)/Homer	1e-4	-1.053e+01	0.0001	12982.0	36.34%	12371.2	35.32%	motif file (matrix)	svg
410	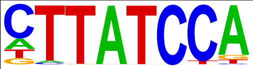	At5g47390(MYBrelated)/col-At5g47390-DAP-Seq(GSE60143)/Homer	1e-4	-1.034e+01	0.0001	14868.0	41.62%	14215.8	40.58%	motif file (matrix)	svg
411	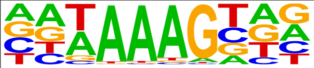	Adof1(C2C2dof)/col-Adof1-DAP-Seq(GSE60143)/Homer	1e-4	-1.034e+01	0.0001	27921.0	78.16%	27070.4	77.28%	motif file (matrix)	svg
412	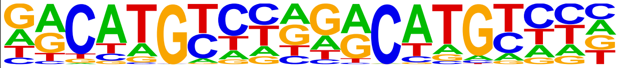	p53(p53)/Saos-p53-ChIP-Seq(GSE15780)/Homer	1e-4	-1.023e+01	0.0001	1306.0	3.66%	1147.7	3.28%	motif file (matrix)	svg
413	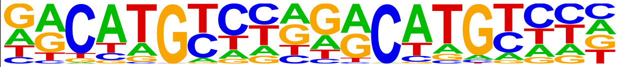	p53(p53)/Saos-p53-ChIP-Seq/Homer	1e-4	-1.023e+01	0.0001	1306.0	3.66%	1147.7	3.28%	motif file (matrix)	svg
414	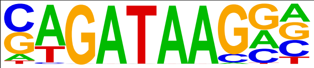	Gata1(Zf)/K562-GATA1-ChIP-Seq(GSE18829)/Homer	1e-4	-1.022e+01	0.0001	7874.0	22.04%	7419.9	21.18%	motif file (matrix)	svg
415	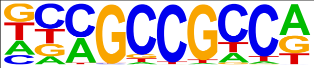	ERF5(AP2EREBP)/colamp-ERF5-DAP-Seq(GSE60143)/Homer	1e-4	-1.003e+01	0.0001	4785.0	13.39%	4448.8	12.70%	motif file (matrix)	svg
416	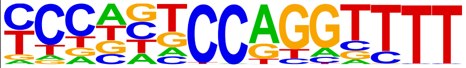	PRDM15(Zf)/ESC-Prdm15-ChIP-Seq(GSE73694)/Homer	1e-4	-1.001e+01	0.0001	16591.0	46.44%	15907.0	45.41%	motif file (matrix)	svg
417	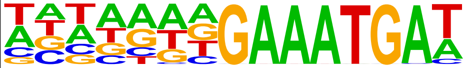	AT2G31460(REMB3)/col-AT2G31460-DAP-Seq(GSE60143)/Homer	1e-4	-9.921e+00	0.0001	5293.0	14.82%	4937.6	14.10%	motif file (matrix)	svg
418	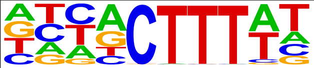	OBP1(C2C2dof)/col-OBP1-DAP-Seq(GSE60143)/Homer	1e-4	-9.866e+00	0.0001	27886.0	78.06%	27045.0	77.20%	motif file (matrix)	svg
419	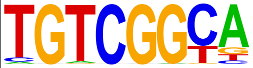	AT1G12630(AP2EREBP)/colamp-AT1G12630-DAP-Seq(GSE60143)/Homer	1e-4	-9.836e+00	0.0001	4637.0	12.98%	4309.9	12.30%	motif file (matrix)	svg
420	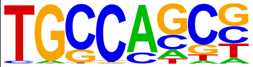	HIC1(Zf)/Treg-ZBTB29-ChIP-Seq(GSE99889)/Homer	1e-4	-9.823e+00	0.0001	27544.0	77.10%	26705.6	76.24%	motif file (matrix)	svg
421	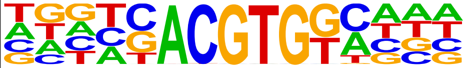	bZIP53(bZIP)/colamp-bZIP53-DAP-Seq(GSE60143)/Homer	1e-4	-9.806e+00	0.0001	4939.0	13.83%	4599.1	13.13%	motif file (matrix)	svg
422	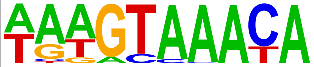	FOXA1(Forkhead)/MCF7-FOXA1-ChIP-Seq(GSE26831)/Homer	1e-4	-9.753e+00	0.0001	12039.0	33.70%	11469.9	32.74%	motif file (matrix)	svg
423	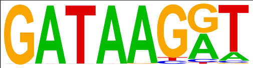	At3g11280(MYBrelated)/col-At3g11280-DAP-Seq(GSE60143)/Homer	1e-4	-9.686e+00	0.0001	10848.0	30.37%	10312.4	29.44%	motif file (matrix)	svg
424	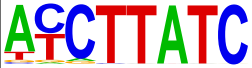	At5g05790(MYBrelated)/col-At5g05790-DAP-Seq(GSE60143)/Homer	1e-4	-9.668e+00	0.0002	11220.0	31.41%	10674.9	30.47%	motif file (matrix)	svg
425	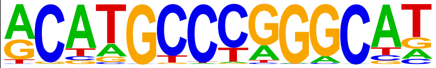	p53(p53)/mES-cMyc-ChIP-Seq(GSE11431)/Homer	1e-4	-9.560e+00	0.0002	285.0	0.80%	221.7	0.63%	motif file (matrix)	svg
426	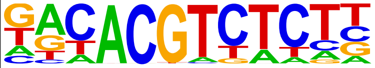	bHLH157(bHLH)/col-bHLH157-DAP-Seq(GSE60143)/Homer	1e-4	-9.440e+00	0.0002	3380.0	9.46%	3113.3	8.89%	motif file (matrix)	svg
427		ZNF7(Zf)/HepG2-ZNF7.Flag-ChIP-Seq(Encode)/Homer	1e-4	-9.417e+00	0.0002	7240.0	20.27%	6821.2	19.47%	motif file	svg

	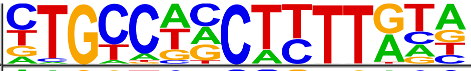									(matrix)	
428	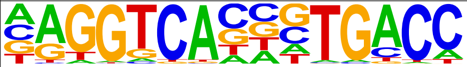	ERE(NR),IR3/MCF7-ERa-ChIP-Seq(Unpublished)/Homer	1e-4	-9.366e+00	0.0002	4397.0	12.31%	4087.0	11.67%	motif file (matrix)	svg
429	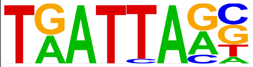	ATHB34(ZFHD)/colamp-ATHB34-DAP-Seq(GSE60143)/Homer	1e-4	-9.233e+00	0.0002	9649.0	27.01%	9157.8	26.14%	motif file (matrix)	svg
430	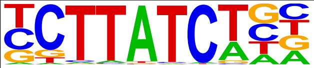	Gata6(Zf)/HUG1N-GATA6-ChIP-Seq(GSE51936)/Homer	1e-3	-8.986e+00	0.0003	11499.0	32.19%	10960.5	31.29%	motif file (matrix)	svg
431	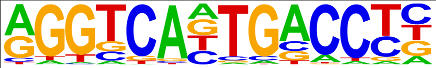	FXR(NR),IR1/Liver-FXR-ChIP-Seq(Chong_et_al.)/Homer	1e-3	-8.863e+00	0.0003	6749.0	18.89%	6357.1	18.15%	motif file (matrix)	svg
432	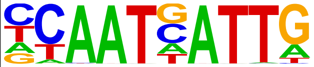	ATHB18(Homeobox)/colamp-ATHB18-DAP-Seq(GSE60143)/Homer	1e-3	-8.796e+00	0.0004	1871.0	5.24%	1689.2	4.82%	motif file (matrix)	svg
433	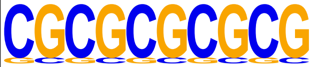	SeqBias: CG-repeat	1e-3	-8.583e+00	0.0004	13255.0	37.10%	12680.5	36.20%	motif file (matrix)	svg
434	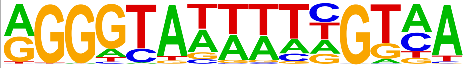	Unknown1/Arabidopsis-Promoters/Homer	1e-3	-8.541e+00	0.0005	1272.0	3.56%	1129.8	3.23%	motif file (matrix)	svg
435	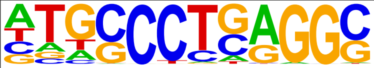	AP-2alpha(AP2)/Hela-AP2alpha-ChIP-Seq(GSE31477)/Homer	1e-3	-8.519e+00	0.0005	15328.0	42.91%	14706.5	41.98%	motif file (matrix)	svg
436	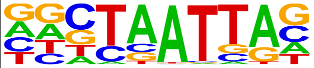	En1(Homeobox)/SUM149-EN1-ChIP-Seq(GSE120957)/Homer	1e-3	-8.438e+00	0.0005	19788.0	55.39%	19079.2	54.47%	motif file (matrix)	svg
437	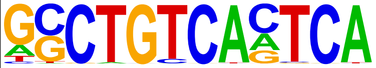	PBX1(Homeobox)/MCF7-PBX1-ChIP-Seq(GSE28007)/Homer	1e-3	-8.420e+00	0.0005	1242.0	3.48%	1103.0	3.15%	motif file (matrix)	svg
438	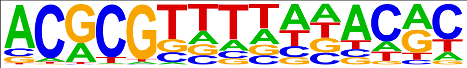	CAMTA5(CAMTA)/col-CAMTA5-DAP-Seq(GSE60143)/Homer	1e-3	-8.395e+00	0.0005	3315.0	9.28%	3065.3	8.75%	motif file (matrix)	svg
439	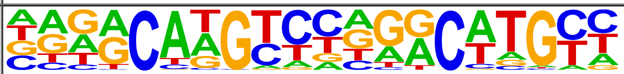	p73(p53)/Trachea-p73-ChIP-Seq(PRJNA310161)/Homer	1e-3	-8.368e+00	0.0005	700.0	1.96%	600.8	1.72%	motif file (matrix)	svg
440	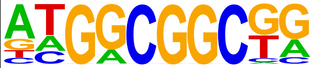	AT4G18450(AP2EREBP)/col-AT4G18450-DAP-Seq(GSE60143)/Homer	1e-3	-8.368e+00	0.0005	4602.0	12.88%	4298.6	12.27%	motif file (matrix)	svg
441	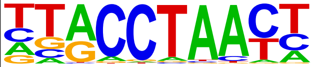	MYB57(MYB)/col-MYB57-DAP-Seq(GSE60143)/Homer	1e-3	-8.339e+00	0.0005	5556.0	15.55%	5216.1	14.89%	motif file (matrix)	svg
442	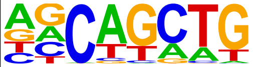	SCL(bHLH)/HPC7-Scl-ChIP-Seq(GSE13511)/Homer	1e-3	-8.318e+00	0.0006	34430.0	96.38%	33637.4	96.02%	motif file (matrix)	svg
443	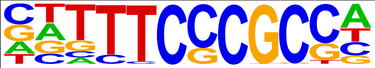	E2F7(E2F)/Hela-E2F7-ChIP-Seq(GSE32673)/Homer	1e-3	-8.187e+00	0.0006	1700.0	4.76%	1534.5	4.38%	motif file (matrix)	svg
444	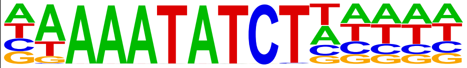	LCL1(MYBrelated)/colamp-LCL1-DAP-Seq(GSE60143)/Homer	1e-3	-8.182e+00	0.0006	1824.0	5.11%	1651.1	4.71%	motif file (matrix)	svg
445	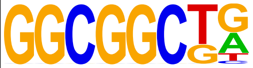	ERF1(AP2EREBP)/colamp-ERF1-DAP-Seq(GSE60143)/Homer	1e-3	-8.140e+00	0.0007	6357.0	17.80%	5992.2	17.11%	motif file (matrix)	svg
446	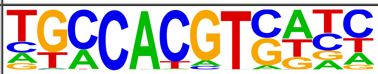	GBF3(bZIP)/Arabidopsis-GBF3-ChIP-Seq(GSE80564)/Homer	1e-3	-8.035e+00	0.0007	6270.0	17.55%	5910.0	16.87%	motif file (matrix)	svg
447	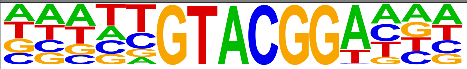	SPL14(SBP)/col-SPL14-DAP-Seq(GSE60143)/Homer	1e-3	-8.033e+00	0.0007	1488.0	4.17%	1336.3	3.81%	motif file (matrix)	svg

448	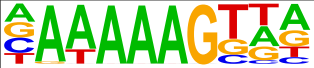	BBX31(Orphan)/col-BBX31-DAP-Seq(GSE60143)/Homer	1e-3	-7.983e+00	0.0008	19919.0	55.76%	19219.6	54.87%	motif file (matrix)	svg
449	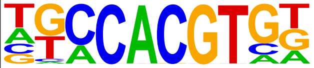	bZIP68(bZIP)/col-bZIP68-DAP-Seq(GSE60143)/Homer	1e-3	-7.839e+00	0.0009	5307.0	14.86%	4985.4	14.23%	motif file (matrix)	svg
450	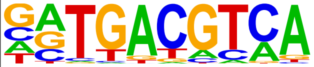	Atf1(bZIP)/K562-ATF1-ChIP-Seq(GSE31477)/Homer	1e-3	-7.822e+00	0.0009	9798.0	27.43%	9332.2	26.64%	motif file (matrix)	svg
451	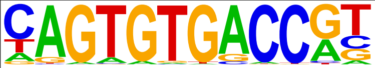	M1BP(Zf)/S2R+-M1BP-ChIP-Seq(GSE49842)/Homer	1e-3	-7.777e+00	0.0009	3799.0	10.63%	3537.0	10.10%	motif file (matrix)	svg
452	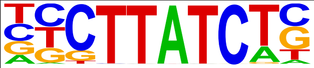	Gata2(Zf)/K562-GATA2-ChIP-Seq(GSE18829)/Homer	1e-3	-7.754e+00	0.0010	8651.0	24.22%	8220.2	23.47%	motif file (matrix)	svg
453	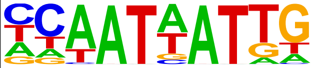	ATHB40(HB)/col-ATHB40-DAP-Seq(GSE60143)/Homer	1e-3	-7.741e+00	0.0010	8963.0	25.09%	8523.5	24.33%	motif file (matrix)	svg
454	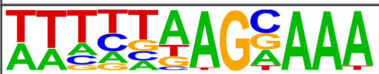	AT5G60130(ABI3VP1)/col-AT5G60130-DAP-Seq(GSE60143)/Homer	1e-3	-7.694e+00	0.0010	20577.0	57.60%	19873.9	56.73%	motif file (matrix)	svg
455	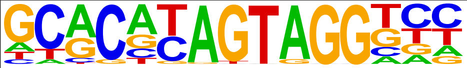	ZKSCAN1(Zf)/HepG2-ZKSCAN1-ChIP-Seq(Encode)/Homer	1e-3	-7.654e+00	0.0010	633.0	1.77%	543.2	1.55%	motif file (matrix)	svg
456	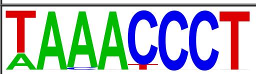	TRP2(MYBrelated)/colamp-TRP2-DAP-Seq(GSE60143)/Homer	1e-3	-7.640e+00	0.0011	2712.0	7.59%	2500.8	7.14%	motif file (matrix)	svg
457	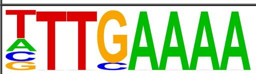	At2g41835(C2H2)/col-At2g41835-DAP-Seq(GSE60143)/Homer	1e-3	-7.535e+00	0.0012	7224.0	20.22%	6842.5	19.53%	motif file (matrix)	svg
458	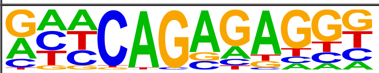	ZNF768(Zf)/Rajj-ZNF768-ChIP-Seq(GSE111879)/Homer	1e-3	-7.448e+00	0.0013	1109.0	3.10%	986.4	2.82%	motif file (matrix)	svg
459	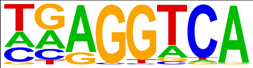	THRb(NR)/Liver-NR1A2-ChIP-Seq(GSE52613)/Homer	1e-3	-7.419e+00	0.0013	33251.0	93.08%	32450.5	92.64%	motif file (matrix)	svg
460	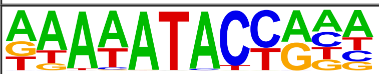	Unknown4/Drosophila-Promoters/Homer	1e-3	-7.395e+00	0.0013	1759.0	4.92%	1598.3	4.56%	motif file (matrix)	svg
461	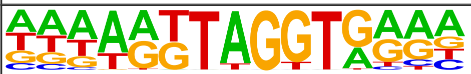	MYB39(MYB)/col-MYB39-DAP-Seq(GSE60143)/Homer	1e-3	-7.363e+00	0.0014	1896.0	5.31%	1728.7	4.93%	motif file (matrix)	svg
462	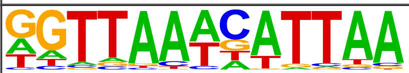	Hnf1(Homeobox)/Liver-Foxa2-Chip-Seq(GSE25694)/Homer	1e-3	-7.300e+00	0.0015	1723.0	4.82%	1565.2	4.47%	motif file (matrix)	svg
463	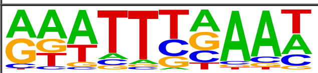	TSO1(CPP)/col-TSO1-DAP-Seq(GSE60143)/Homer	1e-3	-7.273e+00	0.0015	1973.0	5.52%	1802.6	5.15%	motif file (matrix)	svg
464	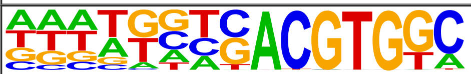	bZIP48(bZIP)/colamp-bZIP48-DAP-Seq(GSE60143)/Homer	1e-3	-7.256e+00	0.0015	5103.0	14.28%	4799.8	13.70%	motif file (matrix)	svg
465	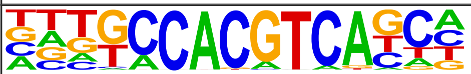	bZIP44(bZIP)/colamp-bZIP44-DAP-Seq(GSE60143)/Homer	1e-3	-7.204e+00	0.0016	322.0	0.90%	263.3	0.75%	motif file (matrix)	svg
466	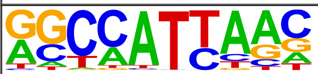	Nanog(Homeobox)/mES-Nanog-ChIP-Seq(GSE11724)/Homer	1e-3	-6.990e+00	0.0020	32947.0	92.23%	32150.1	91.78%	motif file (matrix)	svg
467	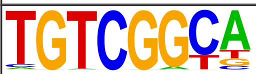	Rap210(AP2EREBP)/col-Rap210-DAP-Seq(GSE60143)/Homer	1e-2	-6.838e+00	0.0023	5322.0	14.90%	5018.8	14.33%	motif file (matrix)	svg
468		ANAC094(NAC)/col-ANAC094-DAP-Seq(GSE60143)/Homer	1e-2	-6.830e+00	0.0023	3337.0	9.34%	3109.6	8.88%	motif file	svg

	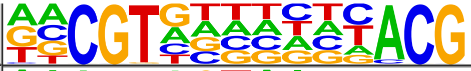									(matrix)	
469	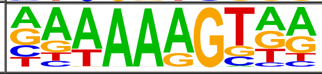	AT5G63260(C3H)/col-AT5G63260-DAP-Seq(GSE60143)/Homer	1e-2	-6.819e+00	0.0023	25735.0	72.04%	24979.3	71.31%	motif file (matrix)	svg
470	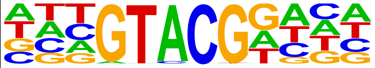	SPL5(SBP)/colamp-SPL5-DAP-Seq(GSE60143)/Homer	1e-2	-6.788e+00	0.0024	4204.0	11.77%	3942.5	11.25%	motif file (matrix)	svg
471	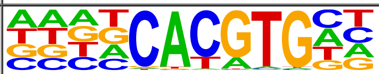	BIM2(bHLH)/col-BIM2-DAP-Seq(GSE60143)/Homer	1e-2	-6.761e+00	0.0025	12529.0	35.07%	12017.7	34.31%	motif file (matrix)	svg
472	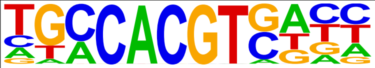	bZIP28(bZIP)/col-bZIP28-DAP-Seq(GSE60143)/Homer	1e-2	-6.740e+00	0.0025	4200.0	11.76%	3939.2	11.25%	motif file (matrix)	svg
473	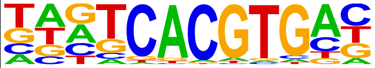	BIM3(bHLH)/col-BIM3-DAP-Seq(GSE60143)/Homer	1e-2	-6.610e+00	0.0029	2053.0	5.75%	1886.0	5.38%	motif file (matrix)	svg
474	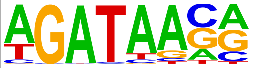	GATA3(Zf)/iTreg-Gata3-ChIP-Seq(GSE20898)/Homer	1e-2	-6.603e+00	0.0029	17066.0	47.77%	16457.3	46.98%	motif file (matrix)	svg
475	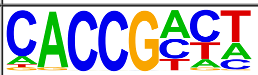	At1g75490(AP2EREBP)/colamp-At1g75490-DAP-Seq(GSE60143)/Homer	1e-2	-6.569e+00	0.0030	17714.0	49.59%	17093.6	48.80%	motif file (matrix)	svg
476	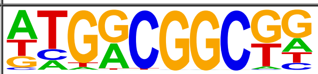	ERF7(AP2EREBP)/col-ERF7-DAP-Seq(GSE60143)/Homer	1e-2	-6.547e+00	0.0030	12681.0	35.50%	12171.3	34.75%	motif file (matrix)	svg
477	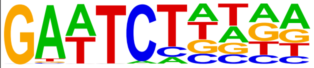	AT1G49560(G2like)/colamp-AT1G49560-DAP-Seq(GSE60143)/Homer	1e-2	-6.527e+00	0.0031	21011.0	58.82%	20331.5	58.04%	motif file (matrix)	svg
478	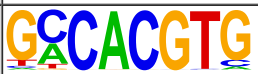	E-box/Arabidopsis-Promoters/Homer	1e-2	-6.522e+00	0.0031	7609.0	21.30%	7237.1	20.66%	motif file (matrix)	svg
479	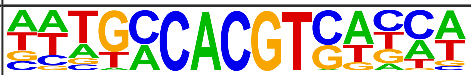	GBF6(bZIP)/colamp-GBF6-DAP-Seq(GSE60143)/Homer	1e-2	-6.510e+00	0.0031	3461.0	9.69%	3233.4	9.23%	motif file (matrix)	svg
480	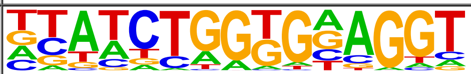	STOP1(C2H2)/colamp-STOP1-DAP-Seq(GSE60143)/Homer	1e-2	-6.502e+00	0.0031	7265.0	20.34%	6904.7	19.71%	motif file (matrix)	svg
481	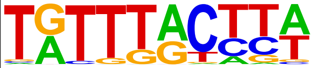	FOXM1(Forkhead)/MCF7-FOXM1-ChIP-Seq(GSE72977)/Homer	1e-2	-6.461e+00	0.0033	12530.0	35.08%	12026.3	34.33%	motif file (matrix)	svg
482	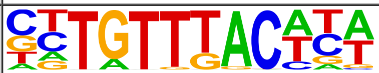	Foxa2(Forkhead)/Liver-Foxa2-ChIP-Seq(GSE25694)/Homer	1e-2	-6.457e+00	0.0033	10413.0	29.15%	9963.1	28.44%	motif file (matrix)	svg
483	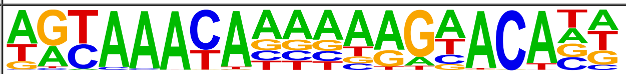	FOXA1:AR(Forkhead,NR)/LNCAP-AR-ChIP-Seq(GSE27824)/Homer	1e-2	-6.206e+00	0.0042	813.0	2.28%	720.1	2.06%	motif file (matrix)	svg
484	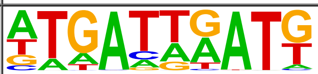	LIN-39(Homeobox)/cElegans.L3-LIN39-ChIP-Seq(modEncode)/Homer	1e-2	-6.107e+00	0.0046	13489.0	37.76%	12972.9	37.03%	motif file (matrix)	svg
485	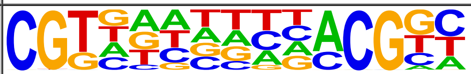	ANAC042(NAC)/col-ANAC042-DAP-Seq(GSE60143)/Homer	1e-2	-6.104e+00	0.0046	5848.0	16.37%	5542.0	15.82%	motif file (matrix)	svg
486	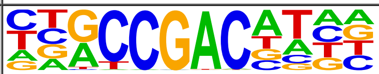	CBF3(AP2EREBP)/colamp-CBF3-DAP-Seq(GSE60143)/Homer	1e-2	-6.054e+00	0.0049	4292.0	12.01%	4040.3	11.53%	motif file (matrix)	svg
487	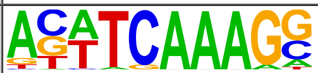	Tcf3(HMG)/mES-Tcf3-ChIP-Seq(GSE11724)/Homer	1e-2	-5.990e+00	0.0052	3353.0	9.39%	3138.1	8.96%	motif file (matrix)	svg
488	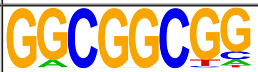	ERF104(AP2EREBP)/col-ERF104-DAP-Seq(GSE60143)/Homer	1e-2	-5.944e+00	0.0054	9607.0	26.89%	9192.9	26.24%	motif file (matrix)	svg

489	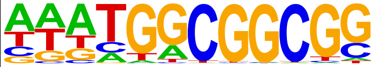	ERF9(AP2EREBP)/colamp-ERF9-DAP-Seq(GSE60143)/Homer	1e-2	-5.911e+00	0.0056	2870.0	8.03%	2676.2	7.64%	motif file (matrix)	svg
490	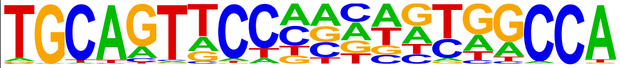	CTCF-SatelliteElement(Zf?)/CD4+-CTCF-ChIP-Seq(Barski_et_al.)/Homer	1e-2	-5.890e+00	0.0057	208.0	0.58%	167.1	0.48%	motif file (matrix)	svg
491	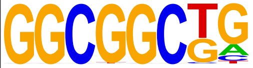	ERF2(AP2EREBP)/colamp-ERF2-DAP-Seq(GSE60143)/Homer	1e-2	-5.835e+00	0.0060	6790.0	19.01%	6460.0	18.44%	motif file (matrix)	svg
492	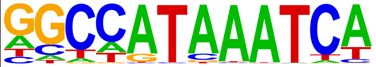	Hoxc9(Homeobox)/Ainv15-Hoxc9-ChIP-Seq(GSE21812)/Homer	1e-2	-5.766e+00	0.0064	6713.0	18.79%	6386.8	18.23%	motif file (matrix)	svg
493	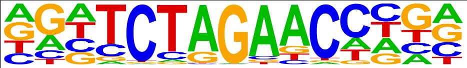	ZBTB12(Zf)/HEK293-ZBTB12.GFP-ChIP-Seq(GSE58341)/Homer	1e-2	-5.629e+00	0.0073	6852.0	19.18%	6524.9	18.63%	motif file (matrix)	svg
494	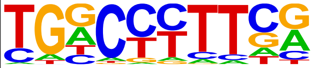	NLP7(RWPRK)/col-NLP7-DAP-Seq(GSE60143)/Homer	1e-2	-5.594e+00	0.0076	24940.0	69.81%	24227.8	69.16%	motif file (matrix)	svg
495	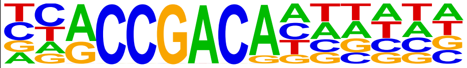	At2g44940(AP2EREBP)/colamp-At2g44940-DAP-Seq(GSE60143)/Homer	1e-2	-5.582e+00	0.0077	1937.0	5.42%	1789.4	5.11%	motif file (matrix)	svg
496	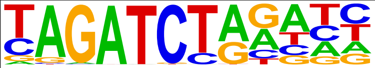	GATA6(C2C2gata)/col200-GATA6-DAP-Seq(GSE60143)/Homer	1e-2	-5.542e+00	0.0080	1759.0	4.92%	1620.8	4.63%	motif file (matrix)	svg
497	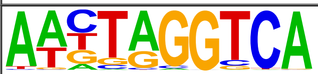	RORgt(NR)/EL4-RORgt.Flag-ChIP-Seq(GSE56019)/Homer	1e-2	-5.473e+00	0.0085	1926.0	5.39%	1780.1	5.08%	motif file (matrix)	svg
498	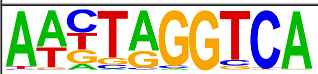	RORgt(NR)/EL4-RORgt.Flag-ChIP-Seq(GSE56019)/Homer	1e-2	-5.473e+00	0.0085	1926.0	5.39%	1780.1	5.08%	motif file (matrix)	svg
499	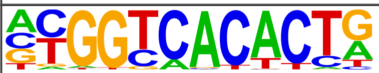	Unknown1(NR/Ini-like)/Drosophila-Promoters/Homer	1e-2	-5.409e+00	0.0090	3176.0	8.89%	2978.9	8.50%	motif file (matrix)	svg
500	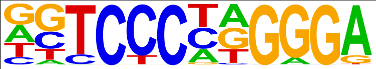	EBF(EBF)/proBcell-EBF-ChIP-Seq(GSE21978)/Homer	1e-2	-5.361e+00	0.0094	5096.0	14.27%	4830.5	13.79%	motif file (matrix)	svg
501	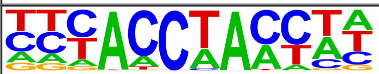	MYB74(MYB)/colamp-MYB74-DAP-Seq(GSE60143)/Homer	1e-2	-5.359e+00	0.0095	7548.0	21.13%	7206.9	20.57%	motif file (matrix)	svg
502	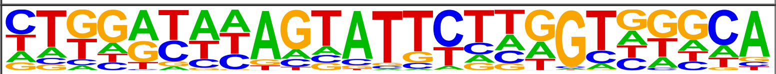	ZNF136(Zf)/HEK293-ZNF136.GFP-ChIP-Seq(GSE58341)/Homer	1e-2	-5.320e+00	0.0098	1148.0	3.21%	1043.6	2.98%	motif file (matrix)	svg
503	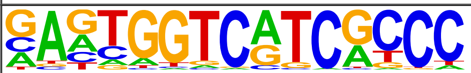	ZNF669(Zf)/HEK293-ZNF669.GFP-ChIP-Seq(GSE58341)/Homer	1e-2	-5.259e+00	0.0104	1329.0	3.72%	1215.0	3.47%	motif file (matrix)	svg
504	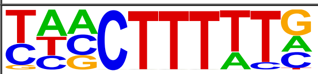	dof24(C2C2dof)/col-dof24-DAP-Seq(GSE60143)/Homer	1e-2	-5.256e+00	0.0104	24085.0	67.42%	23394.9	66.78%	motif file (matrix)	svg
505	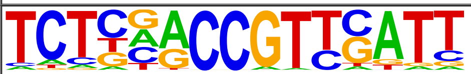	AT4G27900(C2C2COlike)/col-AT4G27900-DAP-Seq(GSE60143)/Homer	1e-2	-5.244e+00	0.0105	67.0	0.19%	47.5	0.14%	motif file (matrix)	svg
506	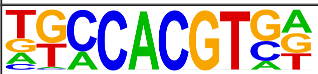	bZIP16(bZIP)/colamp-bZIP16-DAP-Seq(GSE60143)/Homer	1e-2	-5.205e+00	0.0109	4313.0	12.07%	4077.6	11.64%	motif file (matrix)	svg
507	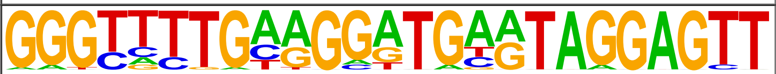	ZFP3(Zf)/HEK293-ZFP3.GFP-ChIP-Seq(GSE58341)/Homer	1e-2	-5.188e+00	0.0111	93.0	0.26%	69.1	0.20%	motif file (matrix)	svg
508	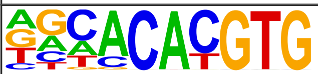	MNT(bHLH)/HepG2-MNT-ChIP-Seq(Encode)/Homer	1e-2	-5.126e+00	0.0118	14486.0	40.55%	13976.7	39.90%	motif file (matrix)	svg
509		GT3a(Trihelix)/col-GT3a-DAP-Seq(GSE60143)/Homer	1e-2	-5.120e+00	0.0118	2478.0	6.94%	2313.2	6.60%	motif file	svg

	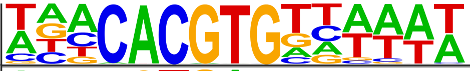									(matrix)	
510	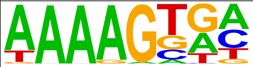	CDF3(C2C2dof)/colamp-CDF3-DAP-Seq(GSE60143)/Homer	1e-2	-5.058e+00	0.0125	21378.0	59.84%	20736.1	59.19%	motif file (matrix)	svg
511	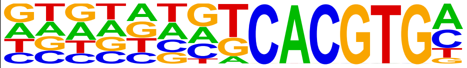	BIM1(bHLH)/colamp-BIM1-DAP-Seq(GSE60143)/Homer	1e-2	-5.014e+00	0.0131	1782.0	4.99%	1649.9	4.71%	motif file (matrix)	svg
512	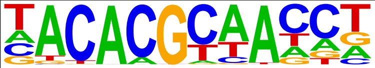	ANAC079(NAC)/colamp-ANAC079-DAP-Seq(GSE60143)/Homer	1e-2	-4.983e+00	0.0135	4896.0	13.71%	4645.2	13.26%	motif file (matrix)	svg
513	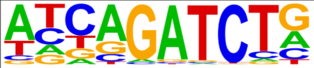	GATA14(C2C2gata)/col-GATA14-DAP-Seq(GSE60143)/Homer	1e-2	-4.978e+00	0.0135	8734.0	24.45%	8369.4	23.89%	motif file (matrix)	svg
514	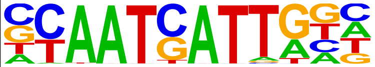	HAT1(Homeobox)/col-HAT1-DAP-Seq(GSE60143)/Homer	1e-2	-4.959e+00	0.0137	1755.0	4.91%	1624.7	4.64%	motif file (matrix)	svg
515	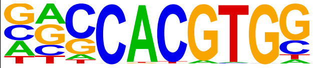	n-Myc(bHLH)/mES-nMyc-ChIP-Seq(GSE11431)/Homer	1e-2	-4.852e+00	0.0153	11700.0	32.75%	11263.2	32.15%	motif file (matrix)	svg
516	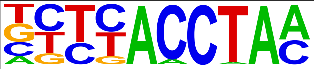	MYB41(MYB)/col-MYB41-DAP-Seq(GSE60143)/Homer	1e-2	-4.839e+00	0.0154	4631.0	12.96%	4392.2	12.54%	motif file (matrix)	svg
517	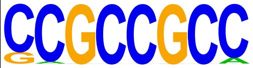	ERF73(AP2EREBP)/col-ERF73-DAP-Seq(GSE60143)/Homer	1e-2	-4.834e+00	0.0155	6782.0	18.98%	6476.5	18.49%	motif file (matrix)	svg
518	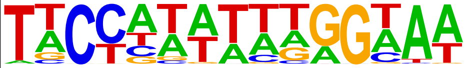	AGL13(MADS)/col-AGL13-DAP-Seq(GSE60143)/Homer	1e-2	-4.812e+00	0.0158	640.0	1.79%	570.8	1.63%	motif file (matrix)	svg
519	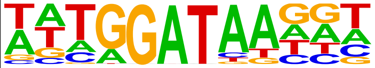	At1g19000(MYBrelated)/colamp-At1g19000-DAP-Seq(GSE60143)/Homer	1e-2	-4.810e+00	0.0158	9982.0	27.94%	9589.6	27.38%	motif file (matrix)	svg
520	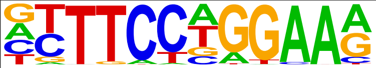	STAT4(Stat)/CD4-Stat4-ChIP-Seq(GSE22104)/Homer	1e-2	-4.747e+00	0.0168	14825.0	41.50%	14320.2	40.88%	motif file (matrix)	svg
521	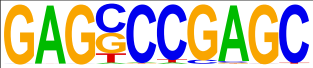	ZNF519(Zf)/HEK293-ZNF519.GFP-ChIP-Seq(GSE58341)/Homer	1e-2	-4.722e+00	0.0172	4028.0	11.28%	3812.5	10.88%	motif file (matrix)	svg
522	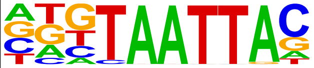	Dlx3(Homeobox)/Kerainocytes-Dlx3-ChIP-Seq(GSE89884)/Homer	1e-2	-4.645e+00	0.0185	7982.0	22.34%	7647.4	21.83%	motif file (matrix)	svg
523	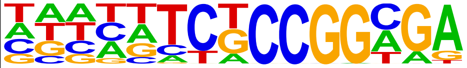	AT5G05550(Trihelix)/col-AT5G05550-DAP-Seq(GSE60143)/Homer	1e-2	-4.640e+00	0.0186	17416.0	48.75%	16861.9	48.14%	motif file (matrix)	svg
