# Supplementary material for: Genome-wide identification of enhancers and transcription factors regulating the myogenic differentiation of bovine satellite cells
Source: BMC Genomics. 2021 Dec 16;22:901. doi: 10.1186/s12864-021-08224-7 (PMC8675486; doi:10.1186/s12864-021-08224-7)
Supplement: Supplementary file 12 — Additional file 12. Motifs enriched in enhancers marked with H3K27ac in before-differentiation bovine satellite cells [file 12864_2021_8224_MOESM12_ESM.pdf]

Motifs enriched in enhancers marked with H3K27ac in before-differentiation bovine satellite cells

Rank	Motif	Name	P-value	log P-pvalue	q-value (Benjamini)	# Target Sequences with Motif	% of Targets Sequences with Motif	# Background Sequences with Motif	% of Background Sequences with Motif	Motif File	SVG
1	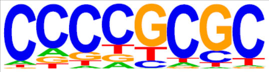	SUT1?/SacCer-Promoters/Homer	1e-20	-4.631e+01	0.0000	5871.0	99.85%	43463.1	98.78%	motif file (matrix)	svg
2	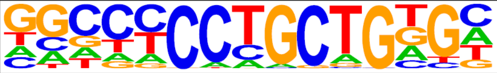	Zic3(Zf)/mES-Zic3-ChIP-Seq(GSE37889)/Homer	1e-14	-3.453e+01	0.0000	2531.0	43.04%	16708.2	37.97%	motif file (matrix)	svg
3	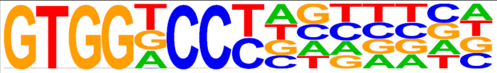	TCP16(TCP)/colamp-TCP16-DAP-Seq(GSE60143)/Homer	1e-14	-3.301e+01	0.0000	2517.0	42.81%	16658.2	37.86%	motif file (matrix)	svg
4	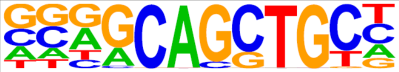	Ascl2(bHLH)/ESC-Ascl2-ChIP-Seq(GSE97712)/Homer	1e-11	-2.579e+01	0.0000	3358.0	57.11%	23188.5	52.70%	motif file (matrix)	svg
5	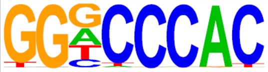	At5g08330(TCP)/col-At5g08330-DAP-Seq(GSE60143)/Homer	1e-10	-2.387e+01	0.0000	2378.0	40.44%	15987.4	36.33%	motif file (matrix)	svg
6	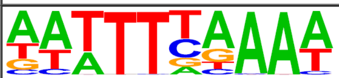	Unknown6/Drosophila-Promoters/Homer	1e-10	-2.337e+01	0.0000	1614.0	27.45%	10481.7	23.82%	motif file (matrix)	svg
7	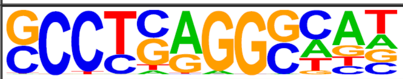	AP-2gamma(AP2)/MCF7-TFAP2C-ChIP-Seq(GSE21234)/Homer	1e-9	-2.203e+01	0.0000	3687.0	62.70%	25842.2	58.73%	motif file (matrix)	svg
8	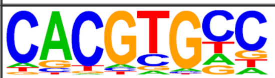	IBL1(bHLH)/Seedling-IBL1-ChIP-Seq(GSE51120)/Homer	1e-9	-2.180e+01	0.0000	4151.0	70.60%	29408.3	66.84%	motif file (matrix)	svg
9	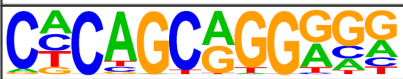	Zic2(Zf)/ESC-Zic2-ChIP-Seq(SRP197560)/Homer	1e-9	-2.176e+01	0.0000	1984.0	33.74%	13204.3	30.01%	motif file (matrix)	svg
10	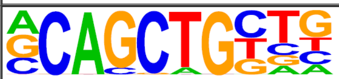	Tcf12(bHLH)/GM12878-Tcf12-ChIP-Seq(GSE32465)/Homer	1e-9	-2.165e+01	0.0000	2967.0	50.46%	20437.5	46.45%	motif file (matrix)	svg
11	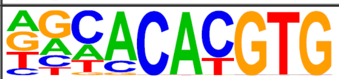	MNT(bHLH)/HepG2-MNT-ChIP-Seq(Encode)/Homer	1e-9	-2.156e+01	0.0000	2805.0	47.70%	19236.5	43.72%	motif file (matrix)	svg
12	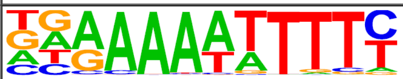	SFP1/SacCer-Promoters/Homer	1e-9	-2.112e+01	0.0000	223.0	3.79%	1085.7	2.47%	motif file (matrix)	svg
13	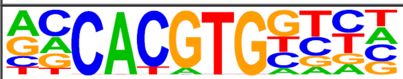	Max(bHLH)/K562-Max-ChIP-Seq(GSE31477)/Homer	1e-9	-2.106e+01	0.0000	2133.0	36.28%	14316.9	32.54%	motif file (matrix)	svg
14	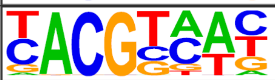	ATAF1(NAC)/col-ATAF1-DAP-Seq(GSE60143)/Homer	1e-9	-2.087e+01	0.0000	4374.0	74.39%	31176.1	70.85%	motif file (matrix)	svg
15		ERF15(AP2EREBP)/colamp-ERF15-	1e-8	-1.967e+01	0.0000	4226.0	71.87%	30081.2	68.37%	motif	svg

	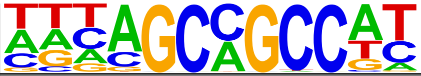	DAP-Seq(GSE60143)/Homer								file (matrix)	
16	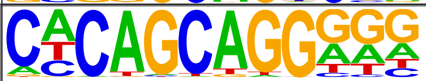	Unknown-ESC-element(?)/mES-Nanog-ChIP-Seq(GSE11724)/Homer	1e-8	-1.966e+01	0.0000	2367.0	40.26%	16089.0	36.57%	motif file (matrix)	svg
17	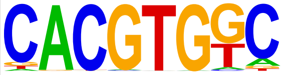	ABF1(bZIP)/Arabidopsis-ABF1-ChIP-Seq(GSE80564)/Homer	1e-8	-1.939e+01	0.0000	2587.0	44.00%	17722.7	40.28%	motif file (matrix)	svg
18	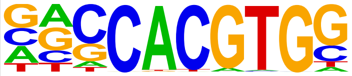	n-Myc(bHLH)/mES-nMyc-ChIP-Seq(GSE11431)/Homer	1e-8	-1.912e+01	0.0000	2398.0	40.78%	16342.0	37.14%	motif file (matrix)	svg
19	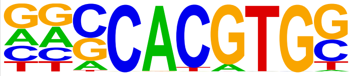	c-Myc(bHLH)/mES-cMyc-ChIP-Seq(GSE11431)/Homer	1e-8	-1.892e+01	0.0000	1849.0	31.45%	12347.6	28.06%	motif file (matrix)	svg
20	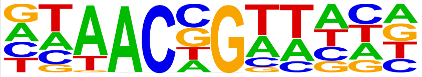	MYB81(MYB)/col-MYB81-DAP-Seq(GSE60143)/Homer	1e-8	-1.882e+01	0.0000	2641.0	44.91%	18148.3	41.25%	motif file (matrix)	svg
21	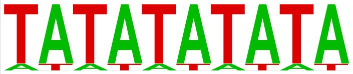	SeqBias: TA-repeat	1e-8	-1.869e+01	0.0000	3649.0	62.06%	25709.8	58.43%	motif file (matrix)	svg
22	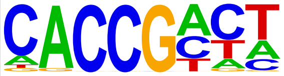	At1g75490(AP2EREBP)/colamp-At1g75490-DAP-Seq(GSE60143)/Homer	1e-7	-1.819e+01	0.0000	3286.0	55.88%	22991.6	52.25%	motif file (matrix)	svg
23	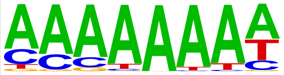	REM19(REM)/colamp-REM19-DAP-Seq(GSE60143)/Homer	1e-7	-1.812e+01	0.0000	1536.0	26.12%	10128.6	23.02%	motif file (matrix)	svg
24	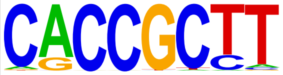	At5g18450(AP2EREBP)/col-At5g18450-DAP-Seq(GSE60143)/Homer	1e-7	-1.801e+01	0.0000	3112.0	52.93%	21694.1	49.30%	motif file (matrix)	svg
25	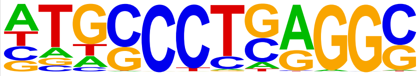	AP-2alpha(AP2)/Hela-AP2alpha-ChIP-Seq(GSE31477)/Homer	1e-7	-1.798e+01	0.0000	3158.0	53.71%	22040.7	50.09%	motif file (matrix)	svg
26	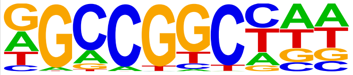	RAP211(AP2EREBP)/colamp-RAP211-DAP-Seq(GSE60143)/Homer	1e-7	-1.766e+01	0.0000	4459.0	75.83%	31979.1	72.68%	motif file (matrix)	svg
27	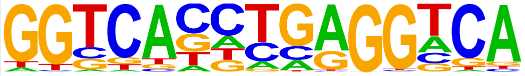	THRb(NR)/HepG2-THRb.Flag-ChIP-Seq(Encode)/Homer	1e-7	-1.762e+01	0.0000	2229.0	37.91%	15173.2	34.48%	motif file (matrix)	svg
28	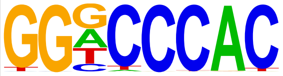	At1g72010(TCP)/colamp-At1g72010-DAP-Seq(GSE60143)/Homer	1e-7	-1.748e+01	0.0000	2027.0	34.47%	13704.7	31.15%	motif file (matrix)	svg
29	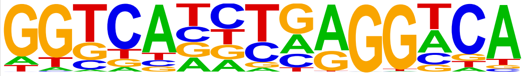	THRa(NR)/C17.2-THRa-ChIP-Seq(GSE38347)/Homer	1e-7	-1.722e+01	0.0000	1576.0	26.80%	10453.8	23.76%	motif file (matrix)	svg
30	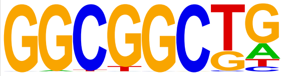	AT5G23930(mTERF)/col-AT5G23930-DAP-Seq(GSE60143)/Homer	1e-7	-1.691e+01	0.0000	3563.0	60.60%	25147.0	57.15%	motif file (matrix)	svg
31	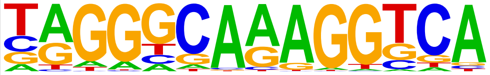	RXR(NR),DR1/3T3L1-RXR-ChIP-Seq(GSE13511)/Homer	1e-7	-1.682e+01	0.0000	3290.0	55.95%	23091.7	52.48%	motif file (matrix)	svg
32	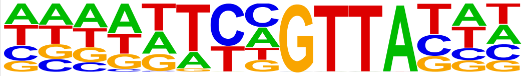	MYB113(MYB)/col-MYB113-DAP-Seq(GSE60143)/Homer	1e-7	-1.646e+01	0.0000	982.0	16.70%	6266.5	14.24%	motif file (matrix)	svg
33		ZFX(Zf)/mES-Zfx-ChIP-	1e-6	-1.612e+01	0.0000	3898.0	66.29%	27734.2	63.03%	motif	svg

	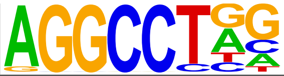	Seq(GSE11431)/Homer								file (matrix)	
34	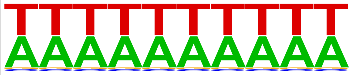	SeqBias: A/T bias	1e-6	-1.610e+01	0.0000	5788.0	98.44%	42868.1	97.43%	motif file (matrix)	svg
35	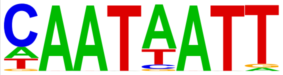	ATHB13(Homeobox)/col-ATHB13-DAP-Seq(GSE60143)/Homer	1e-6	-1.607e+01	0.0000	1703.0	28.96%	11419.9	25.95%	motif file (matrix)	svg
36	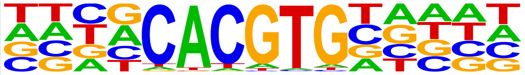	At1g78700(BZR)/col-At1g78700-DAP-Seq(GSE60143)/Homer	1e-6	-1.550e+01	0.0000	1235.0	21.00%	8089.9	18.39%	motif file (matrix)	svg
37	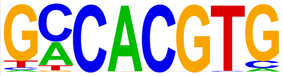	E-box/Arabidopsis-Promoters/Homer	1e-6	-1.539e+01	0.0000	1646.0	27.99%	11039.9	25.09%	motif file (matrix)	svg
38	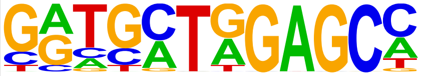	ZNF415(Zf)/HEK293-ZNF415.GFP-ChIP-Seq(GSE58341)/Homer	1e-6	-1.508e+01	0.0000	1927.0	32.77%	13092.9	29.76%	motif file (matrix)	svg
39	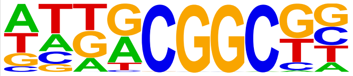	ERF115(AP2EREBP)/colamp-ERF115-DAP-Seq(GSE60143)/Homer	1e-6	-1.493e+01	0.0000	2513.0	42.74%	17400.8	39.55%	motif file (matrix)	svg
40	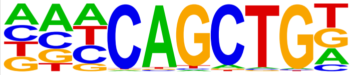	Ap4(bHLH)/AML-Tfap4-ChIP-Seq(GSE45738)/Homer	1e-6	-1.470e+01	0.0000	3362.0	57.18%	23747.4	53.97%	motif file (matrix)	svg
41	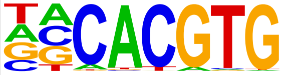	NPAS(bHLH)/Liver-NPAS-ChIP-Seq(GSE39860)/Homer	1e-6	-1.464e+01	0.0000	3673.0	62.47%	26101.1	59.32%	motif file (matrix)	svg
42	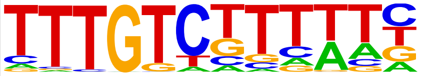	IDD4(C2H2)/col-IDD4-DAP-Seq(GSE60143)/Homer	1e-6	-1.461e+01	0.0000	1618.0	27.52%	10874.4	24.71%	motif file (matrix)	svg
43	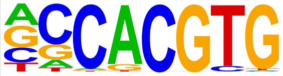	c-Myc(bHLH)/LNCAP-cMyc-ChIP-Seq(Unpublished)/Homer	1e-6	-1.453e+01	0.0000	1679.0	28.55%	11319.7	25.73%	motif file (matrix)	svg
44	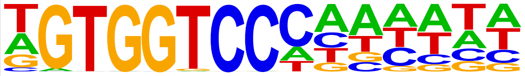	TCP3(TCP)/colamp-TCP3-DAP-Seq(GSE60143)/Homer	1e-6	-1.436e+01	0.0000	1180.0	20.07%	7746.5	17.61%	motif file (matrix)	svg
45	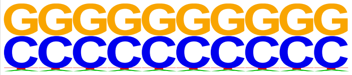	SeqBias: CG bias	1e-6	-1.405e+01	0.0000	5880.0	100.00%	43895.8	99.76%	motif file (matrix)	svg
46	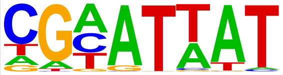	AtHB32(ZFHD)/col200-AtHB32-DAP-Seq(GSE60143)/Homer	1e-6	-1.404e+01	0.0000	1937.0	32.94%	13218.9	30.04%	motif file (matrix)	svg
47	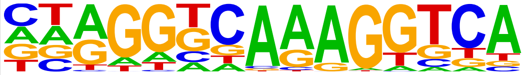	PPARa(NR),DR1/Liver-Ppara-ChIP-Seq(GSE47954)/Homer	1e-6	-1.401e+01	0.0000	2925.0	49.74%	20511.5	46.62%	motif file (matrix)	svg
48	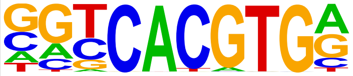	USF1(bHLH)/GM12878-Usf1-ChIP-Seq(GSE32465)/Homer	1e-6	-1.384e+01	0.0000	1599.0	27.19%	10774.4	24.49%	motif file (matrix)	svg
49	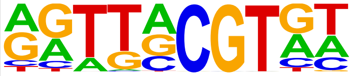	NAM(NAC)/col-NAM-DAP-Seq(GSE60143)/Homer	1e-5	-1.374e+01	0.0000	2305.0	39.20%	15931.3	36.21%	motif file (matrix)	svg
50	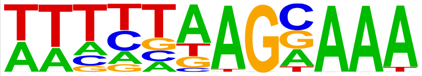	AT5G60130(ABI3VP1)/col-AT5G60130-DAP-Seq(GSE60143)/Homer	1e-5	-1.372e+01	0.0000	3033.0	51.58%	21334.2	48.49%	motif file (matrix)	svg
51		NAP(NAC)/col-NAP-DAP-	1e-5	-1.332e+01	0.0000	1818.0	30.92%	12389.5	28.16%	motif	svg

	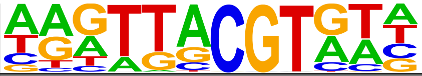	Seq(GSE60143)/Homer								file (matrix)	
52	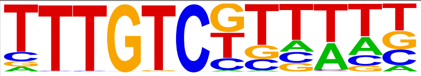	At5g66730(C2H2)/colamp-At5g66730-DAP-Seq(GSE60143)/Homer	1e-5	-1.323e+01	0.0000	595.0	10.12%	3691.8	8.39%	motif file (matrix)	svg
53	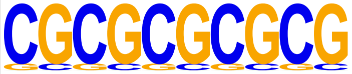	SeqBias: CG-repeat	1e-5	-1.303e+01	0.0000	2695.0	45.83%	18856.5	42.86%	motif file (matrix)	svg
54	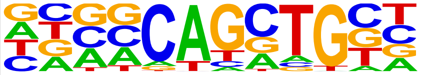	Ascl1(bHLH)/NeuralTubes-Ascl1-ChIP-Seq(GSE55840)/Homer	1e-5	-1.300e+01	0.0000	4200.0	71.43%	30214.3	68.67%	motif file (matrix)	svg
55	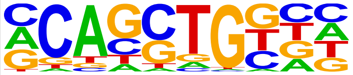	HEB(bHLH)/mES-Heb-ChIP-Seq(GSE53233)/Homer	1e-5	-1.299e+01	0.0000	4672.0	79.46%	33863.3	76.96%	motif file (matrix)	svg
56	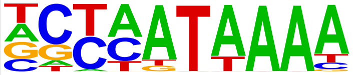	HOXD13(Homeobox)/Chicken-Hoxd13-ChIP-Seq(GSE38910)/Homer	1e-5	-1.288e+01	0.0000	1821.0	30.97%	12434.6	28.26%	motif file (matrix)	svg
57	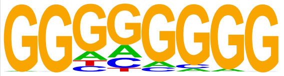	Maz(Zf)/HepG2-Maz-ChIP-Seq(GSE31477)/Homer	1e-5	-1.286e+01	0.0000	3908.0	66.46%	27989.2	63.61%	motif file (matrix)	svg
58	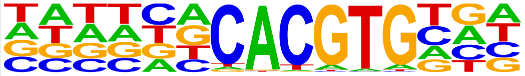	At4g36780(BZR)/col-At4g36780-DAP-Seq(GSE60143)/Homer	1e-5	-1.282e+01	0.0000	1185.0	20.15%	7849.9	17.84%	motif file (matrix)	svg
59	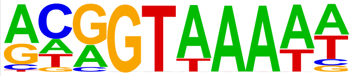	AT1G76880(Trihelix)/col-AT1G76880-DAP-Seq(GSE60143)/Homer	1e-5	-1.278e+01	0.0000	660.0	11.22%	4154.5	9.44%	motif file (matrix)	svg
60	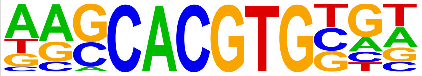	Pho4(bHLH)/Yeast-Pho4-ChIP-Seq(GSE29506)/Homer	1e-5	-1.260e+01	0.0001	947.0	16.11%	6169.9	14.02%	motif file (matrix)	svg
61	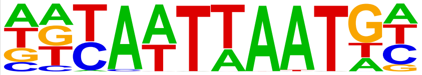	HDG1(Homeobox)/col100-HDG1-DAP-Seq(GSE60143)/Homer	1e-5	-1.250e+01	0.0001	948.0	16.12%	6180.2	14.05%	motif file (matrix)	svg
62	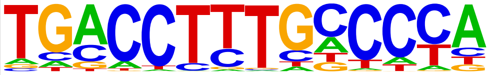	PPARE(NR),DR1/3T3L1-Pparg-ChIP-Seq(GSE13511)/Homer	1e-5	-1.236e+01	0.0001	2809.0	47.77%	19744.2	44.87%	motif file (matrix)	svg
63	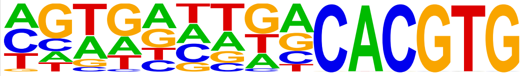	bHLH34(bHLH)/colamp-bHLH34-DAP-Seq(GSE60143)/Homer	1e-5	-1.227e+01	0.0001	1080.0	18.37%	7126.2	16.20%	motif file (matrix)	svg
64	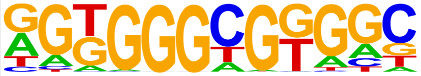	KLF14(Zf)/HEK293-KLF14.GFP-ChIP-Seq(GSE58341)/Homer	1e-5	-1.202e+01	0.0001	4397.0	74.78%	31786.0	72.24%	motif file (matrix)	svg
65	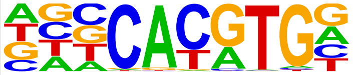	PIF4(bHLH)/Seedling-PIF4-ChIP-Seq(GSE35315)/Homer	1e-5	-1.194e+01	0.0001	3153.0	53.62%	22340.3	50.77%	motif file (matrix)	svg
66	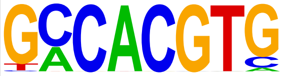	ABI5(bZIP)/col-ABI5-DAP-Seq(GSE60143)/Homer	1e-5	-1.193e+01	0.0001	1410.0	23.98%	9506.7	21.61%	motif file (matrix)	svg
67	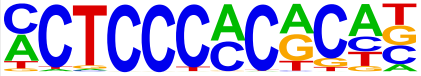	WT1(Zf)/Kidney-WT1-ChIP-Seq(GSE90016)/Homer	1e-5	-1.192e+01	0.0001	2117.0	36.00%	14653.9	33.30%	motif file (matrix)	svg
68	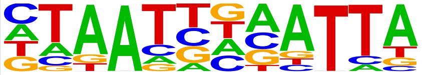	Prop1(Homeobox)/GHFT1-PROP1.biotin-ChIP-Seq(GSE77302)/Homer	1e-5	-1.187e+01	0.0001	1005.0	17.09%	6611.6	15.03%	motif file (matrix)	svg
69		bHLH74(bHLH)/col-bHLH74-DAP-	1e-5	-1.185e+01	0.0001	964.0	16.39%	6321.1	14.37%	motif	svg

	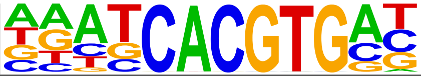	Seq(GSE60143)/Homer								file (matrix)	
70	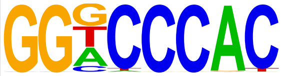	TCP20(TCP)/col-TCP20-DAP-Seq(GSE60143)/Homer	1e-5	-1.184e+01	0.0001	1555.0	26.45%	10558.2	24.00%	motif file (matrix)	svg
71	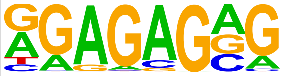	Trl(Zf)/S2-GAGAfactor-ChIP-Seq(GSE40646)/Homer	1e-5	-1.161e+01	0.0001	5009.0	85.19%	36572.9	83.12%	motif file (matrix)	svg
72	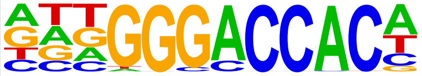	PTF1(TCP)/colamp-PTF1-DAP-Seq(GSE60143)/Homer	1e-5	-1.159e+01	0.0001	504.0	8.57%	3119.5	7.09%	motif file (matrix)	svg
73	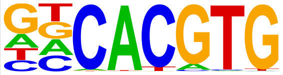	BMAL1(bHLH)/Liver-Bmal1-ChIP-Seq(GSE39860)/Homer	1e-5	-1.155e+01	0.0001	3933.0	66.89%	28258.2	64.22%	motif file (matrix)	svg
74	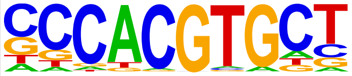	Pho2(bHLH)/Yeast-Pho2-ChIP-Seq(GSE29506)/Homer	1e-4	-1.139e+01	0.0002	1512.0	25.71%	10271.0	23.34%	motif file (matrix)	svg
75	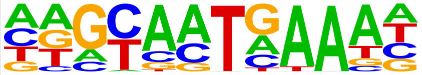	Hoxd12(Homeobox)/ChickenMSG-Hoxd12.Flag-ChIP-Seq(GSE86088)/Homer	1e-4	-1.122e+01	0.0002	3079.0	52.36%	21832.5	49.62%	motif file (matrix)	svg
76	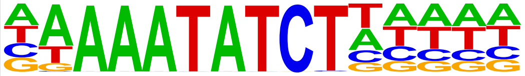	LCL1(MYBrelated)/colamp-LCL1-DAP-Seq(GSE60143)/Homer	1e-4	-1.117e+01	0.0002	269.0	4.57%	1548.6	3.52%	motif file (matrix)	svg
77	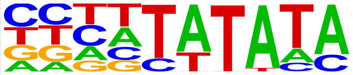	TATA-box/SacCer-Promoters/Homer	1e-4	-1.116e+01	0.0002	852.0	14.49%	5559.4	12.64%	motif file (matrix)	svg
78	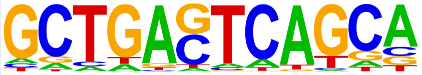	MafK(bZIP)/C2C12-MafK-ChIP-Seq(GSE36030)/Homer	1e-4	-1.097e+01	0.0002	753.0	12.81%	4870.0	11.07%	motif file (matrix)	svg
79	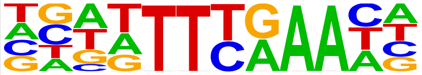	TCX2(CPP)/colamp-TCX2-DAP-Seq(GSE60143)/Homer	1e-4	-1.090e+01	0.0002	2571.0	43.72%	18067.8	41.06%	motif file (matrix)	svg
80	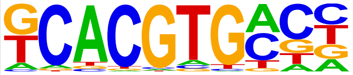	bHLHE41(bHLH)/proB-Bhlhe41-ChIP-Seq(GSE93764)/Homer	1e-4	-1.089e+01	0.0002	2999.0	51.00%	21255.1	48.31%	motif file (matrix)	svg
81	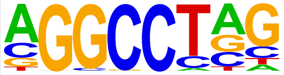	ZNF711(Zf)/SHSY5Y-ZNF711-ChIP-Seq(GSE20673)/Homer	1e-4	-1.085e+01	0.0002	4471.0	76.04%	32423.1	73.69%	motif file (matrix)	svg
82	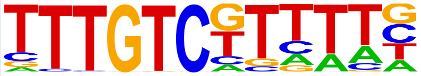	IDD7(C2H2)/col-IDD7-DAP-Seq(GSE60143)/Homer	1e-4	-1.078e+01	0.0003	792.0	13.47%	5152.3	11.71%	motif file (matrix)	svg
83	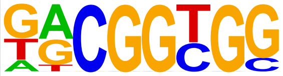	ESE3(AP2EREBP)/col-ESE3-DAP-Seq(GSE60143)/Homer	1e-4	-1.076e+01	0.0003	2170.0	36.90%	15113.6	34.35%	motif file (matrix)	svg
84	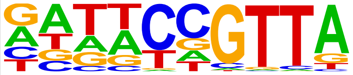	MYB105(MYB)/colamp-MYB105-DAP-Seq(GSE60143)/Homer	1e-4	-1.073e+01	0.0003	1095.0	18.62%	7305.3	16.60%	motif file (matrix)	svg
85	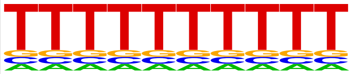	VRN1(ABI3VP1)/col-VRN1-DAP-Seq(GSE60143)/Homer	1e-4	-1.068e+01	0.0003	675.0	11.48%	4335.5	9.85%	motif file (matrix)	svg
86	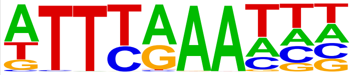	SOL1(CPP)/colamp-SOL1-DAP-Seq(GSE60143)/Homer	1e-4	-1.068e+01	0.0003	2487.0	42.30%	17459.2	39.68%	motif file (matrix)	svg
87		Zac1(Zf)/Neuro2A-Plagl1-ChIP-	1e-4	-1.068e+01	0.0003	5379.0	91.48%	39559.6	89.91%	motif	svg

	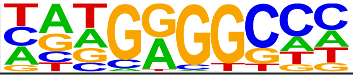	Seq(GSE75942)/Homer								file (matrix)	
88	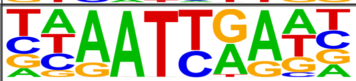	AT1G20910(ARID)/col-AT1G20910-DAP-Seq(GSE60143)/Homer	1e-4	-1.060e+01	0.0003	2344.0	39.86%	16406.1	37.29%	motif file (matrix)	svg
89	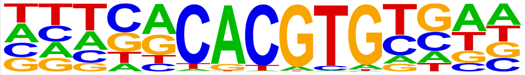	BAM8(BES1)/col-BAM8-DAP-Seq(GSE60143)/Homer	1e-4	-1.054e+01	0.0003	576.0	9.80%	3651.7	8.30%	motif file (matrix)	svg
90	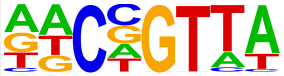	MYB65(MYB)/colamp-MYB65-DAP-Seq(GSE60143)/Homer	1e-4	-1.046e+01	0.0003	2123.0	36.11%	14786.8	33.61%	motif file (matrix)	svg
91	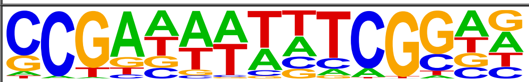	CDM1(C3H)/colamp-CDM1-DAP-Seq(GSE60143)/Homer	1e-4	-1.043e+01	0.0003	192.0	3.27%	1062.7	2.42%	motif file (matrix)	svg
92	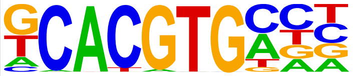	bHLHE40(bHLH)/HepG2-BHLHE40-ChIP-Seq(GSE31477)/Homer	1e-4	-1.031e+01	0.0004	1136.0	19.32%	7619.8	17.32%	motif file (matrix)	svg
93	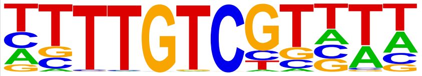	MGP(C2H2)/colamp-MGP-DAP-Seq(GSE60143)/Homer	1e-4	-1.027e+01	0.0004	510.0	8.67%	3204.3	7.28%	motif file (matrix)	svg
94	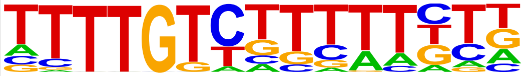	IDD5(C2H2)/colamp-IDD5-DAP-Seq(GSE60143)/Homer	1e-4	-1.024e+01	0.0004	1297.0	22.06%	8780.5	19.96%	motif file (matrix)	svg
95	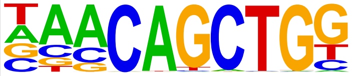	Tcf21(bHLH)/ArterySmoothMuscle-Tcf21-ChIP-Seq(GSE61369)/Homer	1e-4	-1.024e+01	0.0004	2845.0	48.38%	20149.1	45.79%	motif file (matrix)	svg
96	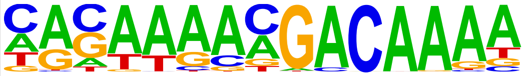	At1g14580(C2H2)/colamp-At1g14580-DAP-Seq(GSE60143)/Homer	1e-4	-1.019e+01	0.0004	412.0	7.01%	2534.4	5.76%	motif file (matrix)	svg
97	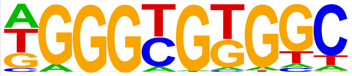	KLF5(Zf)/LoVo-KLF5-ChIP-Seq(GSE49402)/Homer	1e-4	-1.019e+01	0.0004	3561.0	60.56%	25525.7	58.01%	motif file (matrix)	svg
98	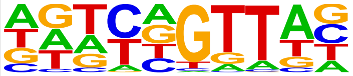	MYB33(MYB)/col-MYB33-DAP-Seq(GSE60143)/Homer	1e-4	-1.013e+01	0.0004	2899.0	49.30%	20559.8	46.73%	motif file (matrix)	svg
99	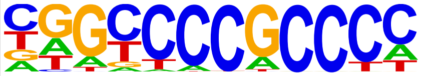	Sp2(Zf)/HEK293-Sp2.eGFP-ChIP-Seq(Encode)/Homer	1e-4	-1.012e+01	0.0004	4130.0	70.24%	29851.7	67.84%	motif file (matrix)	svg
100	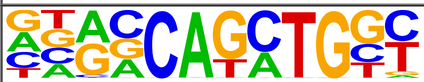	Atoh1(bHLH)/Cerebellum-Atoh1-ChIP-Seq(GSE22111)/Homer	1e-4	-1.011e+01	0.0004	3104.0	52.79%	22093.4	50.21%	motif file (matrix)	svg
101	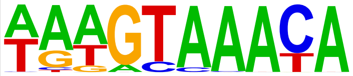	FOXA1(Forkhead)/MCF7-FOXA1-ChIP-Seq(GSE26831)/Homer	1e-4	-9.912e+00	0.0005	1790.0	30.44%	12379.8	28.14%	motif file (matrix)	svg
102	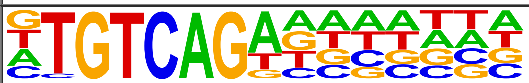	AtGRF6(GRF)/col-AtGRF6-DAP-Seq(GSE60143)/Homer	1e-4	-9.785e+00	0.0006	2959.0	50.32%	21031.9	47.80%	motif file (matrix)	svg
103	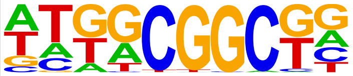	At2g33710(AP2EREBP)/colamp-At2g33710-DAP-Seq(GSE60143)/Homer	1e-4	-9.736e+00	0.0006	2941.0	50.02%	20900.1	47.50%	motif file (matrix)	svg
104	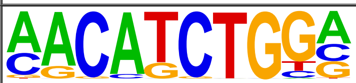	ZBTB18(Zf)/HEK293-ZBTB18.GFP-ChIP-Seq(GSE58341)/Homer	1e-4	-9.716e+00	0.0006	1559.0	26.51%	10707.3	24.33%	motif file (matrix)	svg
105		Tgif1(Homeobox)/mES-Tgif1-ChIP-	1e-4	-9.699e+00	0.0006	4780.0	81.29%	34882.5	79.28%	motif	svg

	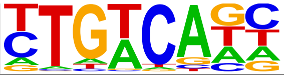	Seq(GSE55404)/Homer								file (matrix)	
106	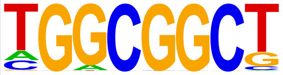	ERF105(AP2EREBP)/colamp-ERF105-DAP-Seq(GSE60143)/Homer	1e-4	-9.692e+00	0.0006	2526.0	42.96%	17815.0	40.49%	motif file (matrix)	svg
107	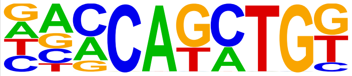	BHLHA15(bHLH)/NIH3T3-BHLHB8.HA-ChIP-Seq(GSE119782)/Homer	1e-4	-9.632e+00	0.0006	3625.0	61.65%	26047.4	59.20%	motif file (matrix)	svg
108	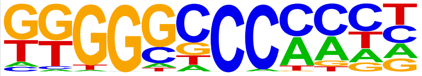	TCP1(TCP)/col-TCP1-DAP-Seq(GSE60143)/Homer	1e-4	-9.599e+00	0.0006	1570.0	26.70%	10794.2	24.53%	motif file (matrix)	svg
109	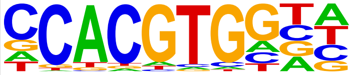	PIF7(bHLH)/col-PIF7-DAP-Seq(GSE60143)/Homer	1e-4	-9.584e+00	0.0006	864.0	14.69%	5714.5	12.99%	motif file (matrix)	svg
110	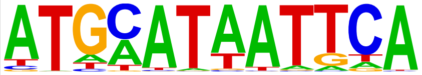	Pit1+1bp(Homeobox)/GCrat-Pit1-ChIP-Seq(GSE58009)/Homer	1e-4	-9.569e+00	0.0006	583.0	9.91%	3736.6	8.49%	motif file (matrix)	svg
111	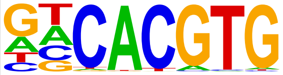	CLOCK(bHLH)/Liver-Clock-ChIP-Seq(GSE39860)/Homer	1e-4	-9.544e+00	0.0006	1793.0	30.49%	12424.1	28.24%	motif file (matrix)	svg
112	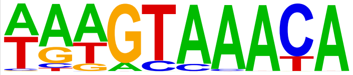	FOXA1(Forkhead)/LNCAP-FOXA1-ChIP-Seq(GSE27824)/Homer	1e-4	-9.420e+00	0.0007	2120.0	36.05%	14832.0	33.71%	motif file (matrix)	svg
113	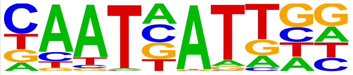	ATHB6(Homeobox)/Arabidopsis-HB6-ChIP-Seq(GSE80564)/Homer	1e-4	-9.379e+00	0.0008	1132.0	19.25%	7639.2	17.36%	motif file (matrix)	svg
114	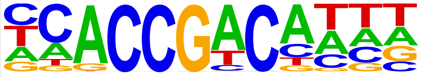	DEAR2(AP2EREBP)/colamp-DEAR2-DAP-Seq(GSE60143)/Homer	1e-4	-9.313e+00	0.0008	1987.0	33.79%	13861.9	31.50%	motif file (matrix)	svg
115	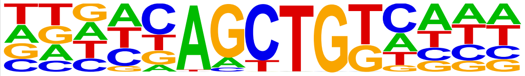	bZIP52(bZIP)/colamp-bZIP52-DAP-Seq(GSE60143)/Homer	1e-4	-9.293e+00	0.0008	3157.0	53.69%	22548.9	51.25%	motif file (matrix)	svg
116	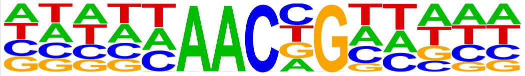	MYB73(MYB)/col-MYB73-DAP-Seq(GSE60143)/Homer	1e-4	-9.225e+00	0.0009	3139.0	53.38%	22418.2	50.95%	motif file (matrix)	svg
117	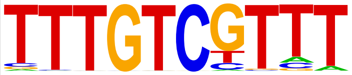	AtIDD11(C2H2)/colamp-AtIDD11-DAP-Seq(GSE60143)/Homer	1e-3	-9.177e+00	0.0009	776.0	13.20%	5109.0	11.61%	motif file (matrix)	svg
118	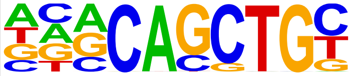	E2A(bHLH)/proBcell-E2A-ChIP-Seq(GSE21978)/Homer	1e-3	-9.167e+00	0.0009	3994.0	67.93%	28878.2	65.63%	motif file (matrix)	svg
119	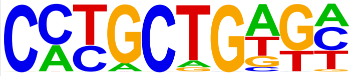	Zic(Zf)/Cerebellum-ZIC1.2-ChIP-Seq(GSE60731)/Homer	1e-3	-9.157e+00	0.0009	2591.0	44.06%	18335.0	41.67%	motif file (matrix)	svg
120	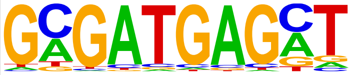	TOD6?/SacCer-Promoters/Homer	1e-3	-9.139e+00	0.0009	572.0	9.73%	3676.4	8.36%	motif file (matrix)	svg
121	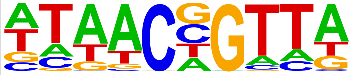	MYB70(MYB)/col-MYB70-DAP-Seq(GSE60143)/Homer	1e-3	-9.125e+00	0.0009	2548.0	43.33%	18017.8	40.95%	motif file (matrix)	svg
122	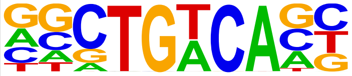	Meis1(Homeobox)/MastCells-Meis1-ChIP-Seq(GSE48085)/Homer	1e-3	-9.111e+00	0.0009	3714.0	63.16%	26757.2	60.81%	motif file (matrix)	svg
123		At1g76110(ARID)/colamp-At1g76110-	1e-3	-8.990e+00	0.0010	1043.0	17.74%	7022.0	15.96%	motif	svg

	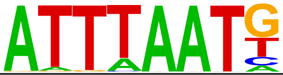	DAP-Seq(GSE60143)/Homer								file (matrix)	
124	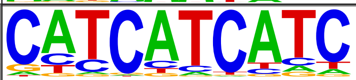	ZML2(C2C2gata)/col-ZML2-DAP-Seq(GSE60143)/Homer	1e-3	-8.987e+00	0.0010	688.0	11.70%	4497.0	10.22%	motif file (matrix)	svg
125	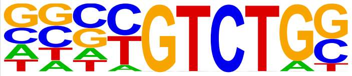	Smad4(MAD)/ESC-SMAD4-ChIP-Seq(GSE29422)/Homer	1e-3	-8.903e+00	0.0011	4044.0	68.78%	29277.9	66.54%	motif file (matrix)	svg
126	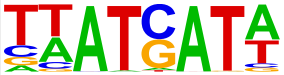	CRC(C2C2YABBY)/col-CRC-DAP-Seq(GSE60143)/Homer	1e-3	-8.880e+00	0.0011	1830.0	31.12%	12738.8	28.95%	motif file (matrix)	svg
127	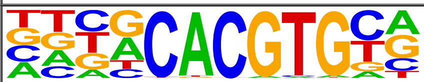	At4g18890(BZR)/col-At4g18890-DAP-Seq(GSE60143)/Homer	1e-3	-8.641e+00	0.0014	742.0	12.62%	4893.9	11.12%	motif file (matrix)	svg
128	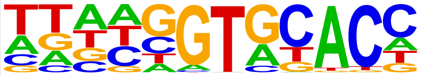	AT3G51470(DBP)/col-AT3G51470-DAP-Seq(GSE60143)/Homer	1e-3	-8.636e+00	0.0014	3222.0	54.80%	23084.7	52.46%	motif file (matrix)	svg
129	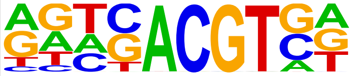	HY5(bZIP)/colamp-HY5-DAP-Seq(GSE60143)/Homer	1e-3	-8.447e+00	0.0017	2009.0	34.17%	14082.0	32.00%	motif file (matrix)	svg
130	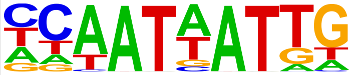	ATHB40(HB)/col-ATHB40-DAP-Seq(GSE60143)/Homer	1e-3	-8.437e+00	0.0017	1242.0	21.12%	8486.0	19.29%	motif file (matrix)	svg
131	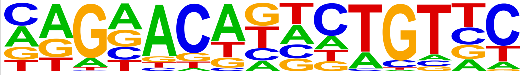	PR(NR)/T47D-PR-ChIP-Seq(GSE31130)/Homer	1e-3	-8.393e+00	0.0017	4182.0	71.12%	30365.9	69.01%	motif file (matrix)	svg
132	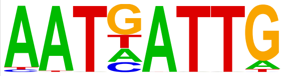	ATHB5(HB)/colamp-ATHB5-DAP-Seq(GSE60143)/Homer	1e-3	-8.370e+00	0.0018	1377.0	23.42%	9467.8	21.52%	motif file (matrix)	svg
133	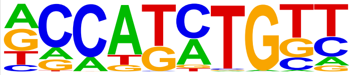	Olig2(bHLH)/Neuron-Olig2-ChIP-Seq(GSE30882)/Homer	1e-3	-8.366e+00	0.0018	4094.0	69.63%	29696.8	67.49%	motif file (matrix)	svg
134	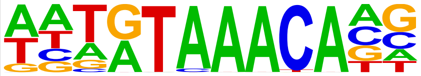	FoxL2(Forkhead)/Ovary-FoxL2-ChIP-Seq(GSE60858)/Homer	1e-3	-8.179e+00	0.0021	1581.0	26.89%	10964.3	24.92%	motif file (matrix)	svg
135	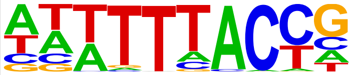	AT5G47660(Trihelix)/colamp-AT5G47660-DAP-Seq(GSE60143)/Homer	1e-3	-8.148e+00	0.0022	2353.0	40.02%	16643.0	37.82%	motif file (matrix)	svg
136	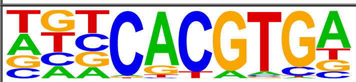	SPCH(bHLH)/Seedling-SPCH-ChIP-Seq(GSE57497)/Homer	1e-3	-8.128e+00	0.0022	2736.0	46.53%	19489.8	44.29%	motif file (matrix)	svg
137	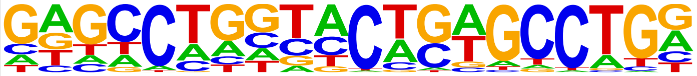	ZNF322(Zf)/HEK293-ZNF322.GFP-ChIP-Seq(GSE58341)/Homer	1e-3	-8.102e+00	0.0022	1250.0	21.26%	8563.4	19.46%	motif file (matrix)	svg
138	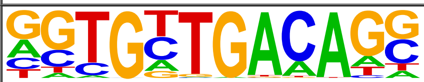	Tbx20(T-box)/Heart-Tbx20-ChIP-Seq(GSE29636)/Homer	1e-3	-8.014e+00	0.0024	661.0	11.24%	4349.5	9.89%	motif file (matrix)	svg
139	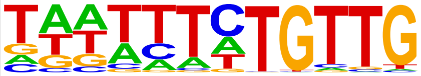	RAV1(RAV)/colamp-RAV1-DAP-Seq(GSE60143)/Homer	1e-3	-7.955e+00	0.0025	1342.0	22.82%	9239.7	21.00%	motif file (matrix)	svg
140	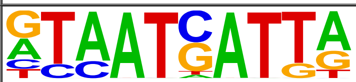	PHV(HB)/col-PHV-DAP-Seq(GSE60143)/Homer	1e-3	-7.921e+00	0.0026	641.0	10.90%	4212.8	9.57%	motif file (matrix)	svg
141		MyoG(bHLH)/C2C12-MyoG-ChIP-	1e-3	-7.843e+00	0.0028	3054.0	51.94%	21886.3	49.74%	motif	svg

	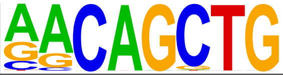	Seq(GSE36024)/Homer								file (matrix)	
142	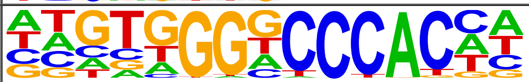	At1g69690(TCP)/colamp-At1g69690-DAP-Seq(GSE60143)/Homer	1e-3	-7.834e+00	0.0028	1049.0	17.84%	7128.1	16.20%	motif file (matrix)	svg
143	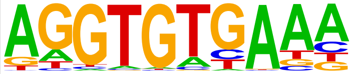	Tbx21(T-box)/GM12878-TBX21-ChIP-Seq(Encode)/Homer	1e-3	-7.812e+00	0.0028	2252.0	38.30%	15921.4	36.18%	motif file (matrix)	svg
144	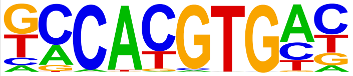	NPAS2(bHLH)/Liver-NPAS2-ChIP-Seq(GSE39860)/Homer	1e-3	-7.812e+00	0.0028	3092.0	52.59%	22173.0	50.39%	motif file (matrix)	svg
145	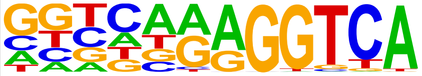	COUP-TFII(NR)/K562-NR2F1-ChIP-Seq(Encode)/Homer	1e-3	-7.803e+00	0.0028	3738.0	63.57%	27036.2	61.45%	motif file (matrix)	svg
146	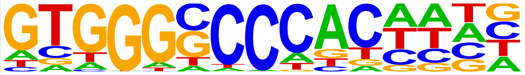	TCP7(TCP)/col-TCP7-DAP-Seq(GSE60143)/Homer	1e-3	-7.758e+00	0.0029	1954.0	33.23%	13727.7	31.20%	motif file (matrix)	svg
147	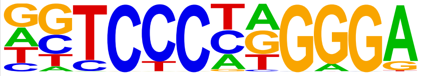	EBF(EBF)/proBcell-EBF-ChIP-Seq(GSE21978)/Homer	1e-3	-7.726e+00	0.0030	1077.0	18.32%	7336.0	16.67%	motif file (matrix)	svg
148	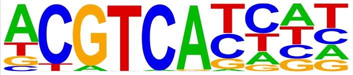	TGA2(bZIP)/colamp-TGA2-DAP-Seq(GSE60143)/Homer	1e-3	-7.718e+00	0.0030	1255.0	21.34%	8623.1	19.60%	motif file (matrix)	svg
149	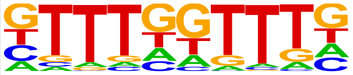	HuR(?)/HEK293-HuR-CLIP-Seq(GSE87887)/Homer	1e-3	-7.654e+00	0.0032	4608.0	78.37%	33681.0	76.55%	motif file (matrix)	svg
150	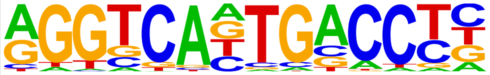	FXR(NR),IR1/Liver-FXR-ChIP-Seq(Chong_et_al.)/Homer	1e-3	-7.609e+00	0.0033	1393.0	23.69%	9632.1	21.89%	motif file (matrix)	svg
151	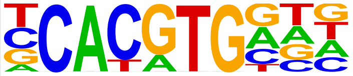	PIF5ox(bHLH)/Arabidopsis-PIF5ox-ChIP-Seq(GSE35062)/Homer	1e-3	-7.577e+00	0.0034	2764.0	47.01%	19741.2	44.87%	motif file (matrix)	svg
152	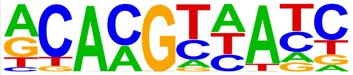	ANAC038(NAC)/col-ANAC038-DAP-Seq(GSE60143)/Homer	1e-3	-7.571e+00	0.0034	3555.0	60.46%	25672.5	58.35%	motif file (matrix)	svg
153	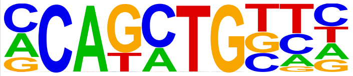	Twist2(bHLH)/Myoblast-Twist2.Ty1-ChIP-Seq(GSE127998)/Homer	1e-3	-7.532e+00	0.0035	4109.0	69.88%	29872.6	67.89%	motif file (matrix)	svg
154	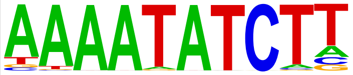	At3g09600(MYBrelated)/colamp-At3g09600-DAP-Seq(GSE60143)/Homer	1e-3	-7.486e+00	0.0037	776.0	13.20%	5191.9	11.80%	motif file (matrix)	svg
155	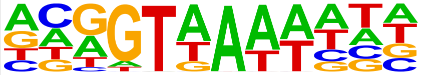	GT2(Trihelix)/colamp-GT2-DAP-Seq(GSE60143)/Homer	1e-3	-7.409e+00	0.0039	1747.0	29.71%	12232.5	27.80%	motif file (matrix)	svg
156	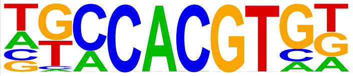	bZIP68(bZIP)/col-bZIP68-DAP-Seq(GSE60143)/Homer	1e-3	-7.232e+00	0.0047	1083.0	18.42%	7409.5	16.84%	motif file (matrix)	svg
157	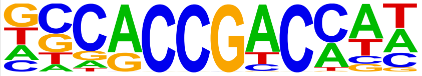	At4g28140(AP2EREBP)/colamp-At4g28140-DAP-Seq(GSE60143)/Homer	1e-3	-7.186e+00	0.0049	647.0	11.00%	4289.4	9.75%	motif file (matrix)	svg
158	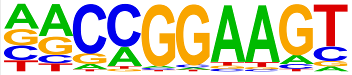	GABPA(ETS)/Jurkat-GABPa-ChIP-Seq(GSE17954)/Homer	1e-3	-7.173e+00	0.0049	2307.0	39.23%	16378.4	37.22%	motif file (matrix)	svg
159		ZNF692(Zf)/HEK293-ZNF692.GFP-	1e-3	-7.150e+00	0.0050	749.0	12.74%	5016.1	11.40%	motif	svg

	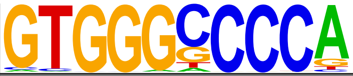	ChIP-Seq(GSE58341)/Homer								file (matrix)	
160	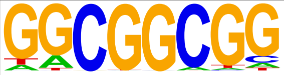	AT1G28160(AP2EREBP)/colamp-AT1G28160-DAP-Seq(GSE60143)/Homer	1e-3	-7.124e+00	0.0051	3055.0	51.96%	21952.9	49.89%	motif file (matrix)	svg
161	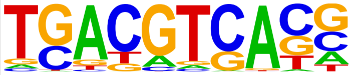	FEA4(bZIP)/Corn-FEA4-ChIP-Seq(GSE61954)/Homer	1e-3	-7.067e+00	0.0053	2727.0	46.38%	19507.4	44.33%	motif file (matrix)	svg
162	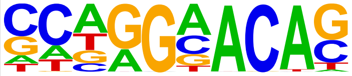	AR-halfsite(NR)/LNCaP-AR-ChIP-Seq(GSE27824)/Homer	1e-3	-7.060e+00	0.0053	5541.0	94.23%	41018.0	93.22%	motif file (matrix)	svg
163	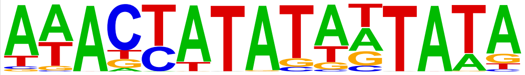	AT1G04880(ARID)/colamp-AT1G04880-DAP-Seq(GSE60143)/Homer	1e-3	-6.959e+00	0.0059	205.0	3.49%	1227.8	2.79%	motif file (matrix)	svg
164	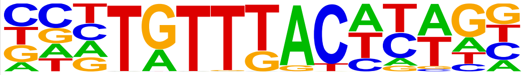	Foxa3(Forkhead)/Liver-Foxa3-ChIP-Seq(GSE77670)/Homer	1e-2	-6.889e+00	0.0063	641.0	10.90%	4261.0	9.68%	motif file (matrix)	svg
165	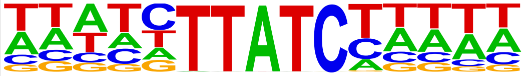	At5g58900(MYBrelated)/colamp-At5g58900-DAP-Seq(GSE60143)/Homer	1e-2	-6.887e+00	0.0063	2321.0	39.47%	16505.6	37.51%	motif file (matrix)	svg
166	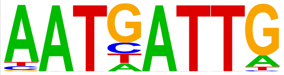	ATHB6(Homeobox)/col-ATHB6-DAP-Seq(GSE60143)/Homer	1e-2	-6.830e+00	0.0065	1810.0	30.78%	12738.9	28.95%	motif file (matrix)	svg
167	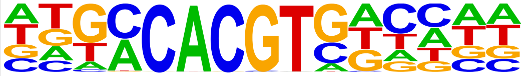	AREB3(bZIP)/col-AREB3-DAP-Seq(GSE60143)/Homer	1e-2	-6.827e+00	0.0065	962.0	16.36%	6561.3	14.91%	motif file (matrix)	svg
168	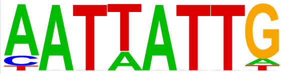	LMI1(HB)/colamp-LMI1-DAP-Seq(GSE60143)/Homer	1e-2	-6.769e+00	0.0069	1096.0	18.64%	7532.1	17.12%	motif file (matrix)	svg
169	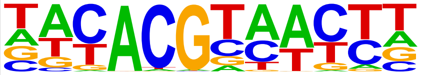	ANAC047(NAC)/colamp-ANAC047-DAP-Seq(GSE60143)/Homer	1e-2	-6.768e+00	0.0069	1432.0	24.35%	9974.0	22.67%	motif file (matrix)	svg
170	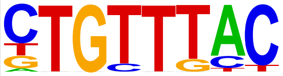	Foxo1(Forkhead)/RAW-Foxo1-ChIP-Seq(Fan_et_al.)/Homer	1e-2	-6.680e+00	0.0074	3693.0	62.81%	26787.1	60.88%	motif file (matrix)	svg
171	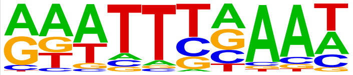	TSO1(CPP)/col-TSO1-DAP-Seq(GSE60143)/Homer	1e-2	-6.596e+00	0.0080	266.0	4.52%	1651.5	3.75%	motif file (matrix)	svg
172	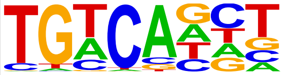	Tgif2(Homeobox)/mES-Tgif2-ChIP-Seq(GSE55404)/Homer	1e-2	-6.550e+00	0.0084	4989.0	84.85%	36699.2	83.41%	motif file (matrix)	svg
173	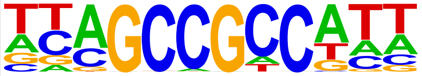	ERF13(AP2EREBP)/colamp-ERF13-DAP-Seq(GSE60143)/Homer	1e-2	-6.522e+00	0.0086	2069.0	35.19%	14671.8	33.34%	motif file (matrix)	svg
174	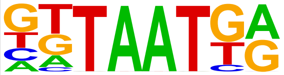	Nkx6.1(Homeobox)/Islet-Nkx6.1-ChIP-Seq(GSE40975)/Homer	1e-2	-6.454e+00	0.0091	3621.0	61.58%	26263.9	59.69%	motif file (matrix)	svg
175	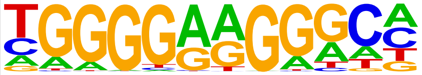	ZNF467(Zf)/HEK293-ZNF467.GFP-ChIP-Seq(GSE58341)/Homer	1e-2	-6.440e+00	0.0092	2970.0	50.51%	21375.0	48.58%	motif file (matrix)	svg
176	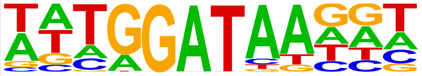	At1g19000(MYBrelated)/colamp-At1g19000-DAP-Seq(GSE60143)/Homer	1e-2	-6.395e+00	0.0095	1425.0	24.23%	9950.8	22.62%	motif file (matrix)	svg
177		FOXM1(Forkhead)/MCF7-FOXM1-	1e-2	-6.388e+00	0.0096	1877.0	31.92%	13266.8	30.15%	motif	svg

	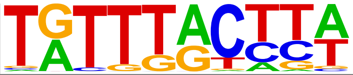	ChIP-Seq(GSE72977)/Homer								file (matrix)	
178	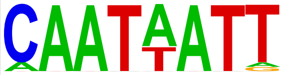	ATHB53(HB)/col-ATHB53-DAP-Seq(GSE60143)/Homer	1e-2	-6.382e+00	0.0096	795.0	13.52%	5387.6	12.24%	motif file (matrix)	svg
179	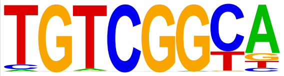	AT1G12630(AP2EREBP)/colamp-AT1G12630-DAP-Seq(GSE60143)/Homer	1e-2	-6.342e+00	0.0099	880.0	14.97%	6000.4	13.64%	motif file (matrix)	svg
180	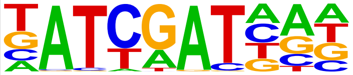	CUX1(Homeobox)/K562-CUX1-ChIP-Seq(GSE92882)/Homer	1e-2	-6.335e+00	0.0099	1078.0	18.33%	7430.1	16.89%	motif file (matrix)	svg
181	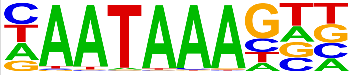	PABPC1(?)/MEL-PABC1-CLIP-Seq(GSE69755)/Homer	1e-2	-6.303e+00	0.0102	2980.0	50.68%	21462.2	48.78%	motif file (matrix)	svg
182	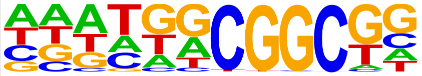	RAP26(AP2EREBP)/colamp-RAP26-DAP-Seq(GSE60143)/Homer	1e-2	-6.281e+00	0.0103	1864.0	31.70%	13179.7	29.95%	motif file (matrix)	svg
183	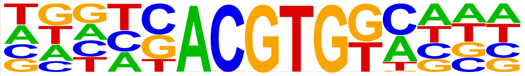	bZIP53(bZIP)/colamp-bZIP53-DAP-Seq(GSE60143)/Homer	1e-2	-6.279e+00	0.0103	994.0	16.90%	6826.3	15.51%	motif file (matrix)	svg
184	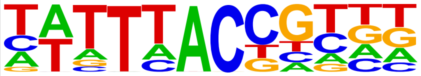	EMB1789(C3H)/col-EMB1789-DAP-Seq(GSE60143)/Homer	1e-2	-6.273e+00	0.0103	719.0	12.23%	4849.2	11.02%	motif file (matrix)	svg
185	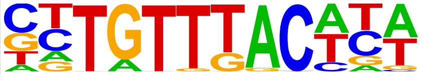	Foxa2(Forkhead)/Liver-Foxa2-ChIP-Seq(GSE25694)/Homer	1e-2	-6.266e+00	0.0103	1596.0	27.14%	11211.6	25.48%	motif file (matrix)	svg
186	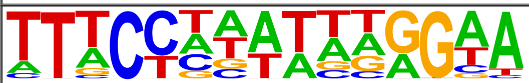	AGL15(MADS)/col-AGL15-DAP-Seq(GSE60143)/Homer	1e-2	-6.213e+00	0.0108	296.0	5.03%	1870.3	4.25%	motif file (matrix)	svg
187	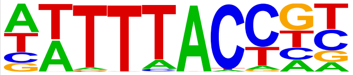	GTL1(Trihelix)/colamp-GTL1-DAP-Seq(GSE60143)/Homer	1e-2	-6.208e+00	0.0108	1842.0	31.33%	13023.3	29.60%	motif file (matrix)	svg
188	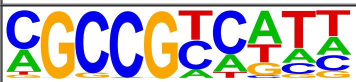	ERF8(AP2EREBP)/colamp-ERF8-DAP-Seq(GSE60143)/Homer	1e-2	-6.172e+00	0.0112	1675.0	28.49%	11798.4	26.81%	motif file (matrix)	svg
189	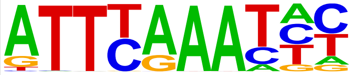	AT2G20110(CPP)/colamp-AT2G20110-DAP-Seq(GSE60143)/Homer	1e-2	-6.154e+00	0.0113	2399.0	40.80%	17146.4	38.97%	motif file (matrix)	svg
190	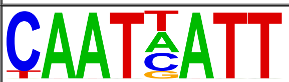	ATHB20(Homeobox)/colamp-ATHB20-DAP-Seq(GSE60143)/Homer	1e-2	-6.142e+00	0.0114	622.0	10.58%	4164.2	9.46%	motif file (matrix)	svg
191	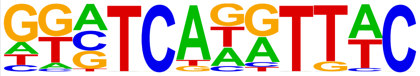	Six1(Homeobox)/Myoblast-Six1-ChIP-Chip(GSE20150)/Homer	1e-2	-6.120e+00	0.0116	575.0	9.78%	3831.0	8.71%	motif file (matrix)	svg
192	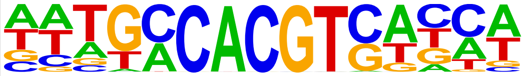	GBF6(bZIP)/colamp-GBF6-DAP-Seq(GSE60143)/Homer	1e-2	-6.104e+00	0.0117	703.0	11.96%	4744.2	10.78%	motif file (matrix)	svg
193	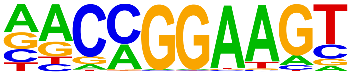	ETV1(ETS)/GIST48-ETV1-ChIP-Seq(GSE22441)/Homer	1e-2	-6.103e+00	0.0117	3260.0	55.44%	23578.2	53.59%	motif file (matrix)	svg
194	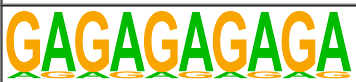	SeqBias: GA-repeat	1e-2	-6.081e+00	0.0119	5864.0	99.73%	43768.6	99.47%	motif file (matrix)	svg
195		caudal(Homeobox)/Drosophila-	1e-2	-6.081e+00	0.0119	1452.0	24.69%	10171.0	23.12%	motif	svg

	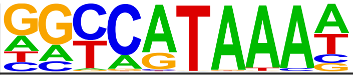	Embryos-ChIP-Chip(modEncode)/Homer								file (matrix)	
196	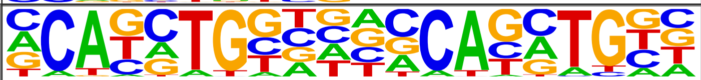	Twist(bHLH)/HMLE-TWIST1-ChIP-Seq(Chang_et_al)/Homer	1e-2	-6.071e+00	0.0119	525.0	8.93%	3479.8	7.91%	motif file (matrix)	svg
197	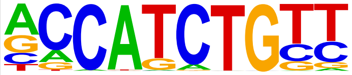	NeuroG2(bHLH)/Fibroblast-NeuroG2-ChIP-Seq(GSE75910)/Homer	1e-2	-6.012e+00	0.0125	3741.0	63.62%	27208.2	61.84%	motif file (matrix)	svg
198	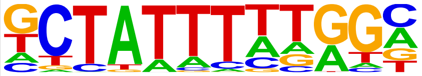	Mef2b(MADS)/HEK293-Mef2b.V5-ChIP-Seq(GSE67450)/Homer	1e-2	-5.995e+00	0.0127	1466.0	24.93%	10280.4	23.36%	motif file (matrix)	svg
199	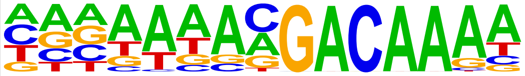	IDD2(C2H2)/colamp-IDD2-DAP-Seq(GSE60143)/Homer	1e-2	-5.987e+00	0.0127	277.0	4.71%	1747.6	3.97%	motif file (matrix)	svg
200	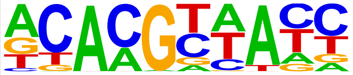	ANAC046(NAC)/colamp-ANAC046-DAP-Seq(GSE60143)/Homer	1e-2	-5.966e+00	0.0129	3217.0	54.71%	23268.4	52.88%	motif file (matrix)	svg
201	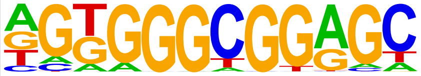	Sp5(Zf)/mES-Sp5.Flag-ChIP-Seq(GSE72989)/Homer	1e-2	-5.878e+00	0.0140	2930.0	49.83%	21127.5	48.02%	motif file (matrix)	svg
202	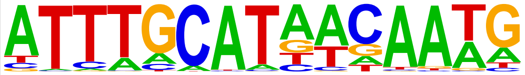	OCT4-SOX2-TCF-NANOG(POU,Homeobox,HMG)/mES-Oct4-ChIP-Seq(GSE11431)/Homer	1e-2	-5.877e+00	0.0140	298.0	5.07%	1896.9	4.31%	motif file (matrix)	svg
203	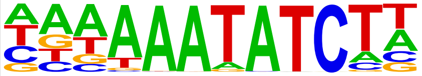	At5g52660(MYBrelated)/colamp-At5g52660-DAP-Seq(GSE60143)/Homer	1e-2	-5.831e+00	0.0146	802.0	13.64%	5470.2	12.43%	motif file (matrix)	svg
204	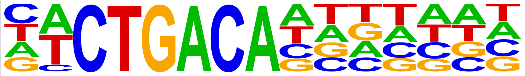	GRF9(GRF)/colamp-GRF9-DAP-Seq(GSE60143)/Homer	1e-2	-5.825e+00	0.0146	2407.0	40.94%	17235.4	39.17%	motif file (matrix)	svg
205	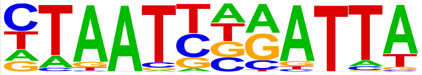	Phox2a(Homeobox)/Neuron-Phox2a-ChIP-Seq(GSE31456)/Homer	1e-2	-5.817e+00	0.0146	634.0	10.78%	4267.1	9.70%	motif file (matrix)	svg
206	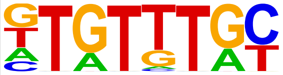	PHA-4(Forkhead)/cElegans-Embryos-PHA4-ChIP-Seq(modEncode)/Homer	1e-2	-5.815e+00	0.0146	4602.0	78.27%	33771.7	76.75%	motif file (matrix)	svg
207	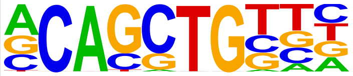	Ptf1a(bHLH)/Panc1-Ptf1a-ChIP-Seq(GSE47459)/Homer	1e-2	-5.798e+00	0.0147	5224.0	88.84%	38576.0	87.67%	motif file (matrix)	svg
208	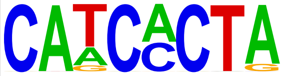	Unknown2/Drosophila-Promoters/Homer	1e-2	-5.790e+00	0.0148	3389.0	57.64%	24576.8	55.86%	motif file (matrix)	svg
209	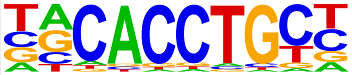	Snail1(Zf)/LS174T-SNAIL1.HA-ChIP-Seq(GSE127183)/Homer	1e-2	-5.786e+00	0.0148	2751.0	46.79%	19799.8	45.00%	motif file (matrix)	svg
210	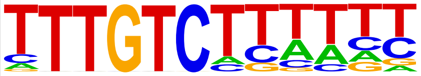	SGR5(C2H2)/colamp-SGR5-DAP-Seq(GSE60143)/Homer	1e-2	-5.754e+00	0.0152	1400.0	23.81%	9816.8	22.31%	motif file (matrix)	svg
211	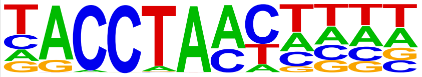	AT3G10580(MYBrelated)/colamp-AT3G10580-DAP-Seq(GSE60143)/Homer	1e-2	-5.667e+00	0.0165	781.0	13.28%	5329.3	12.11%	motif file (matrix)	svg
212	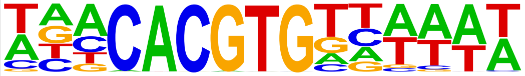	GT3a(Trihelix)/col-GT3a-DAP-Seq(GSE60143)/Homer	1e-2	-5.660e+00	0.0165	486.0	8.27%	3223.5	7.33%	motif file (matrix)	svg
213		CES-1(Homeobox)/cElegans-L1-CES1-	1e-2	-5.657e+00	0.0165	857.0	14.57%	5877.2	13.36%	motif	svg

	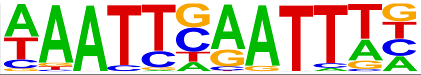	ChIP-Seq(modEncode)/Homer								file (matrix)	
214	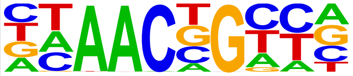	BMYB(HTH)/Hela-BMYB-ChIP-Seq(GSE27030)/Homer	1e-2	-5.615e+00	0.0171	2970.0	50.51%	21452.0	48.75%	motif file (matrix)	svg
215	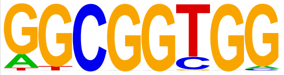	AT3G57600(AP2EREBP)/col-AT3G57600-DAP-Seq(GSE60143)/Homer	1e-2	-5.613e+00	0.0171	1748.0	29.73%	12381.4	28.14%	motif file (matrix)	svg
216	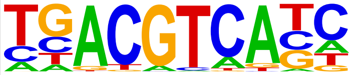	TGA6(bZIP)/colamp-TGA6-DAP-Seq(GSE60143)/Homer	1e-2	-5.598e+00	0.0172	1224.0	20.82%	8543.3	19.42%	motif file (matrix)	svg
217	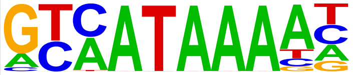	Cdx2(Homeobox)/mES-Cdx2-ChIP-Seq(GSE14586)/Homer	1e-2	-5.594e+00	0.0172	1195.0	20.32%	8332.8	18.94%	motif file (matrix)	svg
218	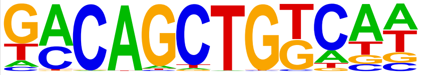	bZIP69(bZIP)/col-bZIP69-DAP-Seq(GSE60143)/Homer	1e-2	-5.525e+00	0.0184	379.0	6.45%	2475.4	5.63%	motif file (matrix)	svg
219	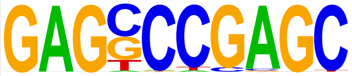	ZNF519(Zf)/HEK293-ZNF519.GFP-ChIP-Seq(GSE58341)/Homer	1e-2	-5.523e+00	0.0184	816.0	13.88%	5590.9	12.71%	motif file (matrix)	svg
220	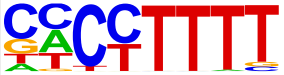	AT5G02460(C2C2dof)/col-AT5G02460-DAP-Seq(GSE60143)/Homer	1e-2	-5.484e+00	0.0190	3683.0	62.64%	26819.9	60.95%	motif file (matrix)	svg
221	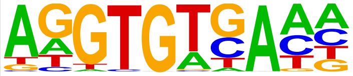	Tbet(T-box)/CD8-Tbet-ChIP-Seq(GSE33802)/Homer	1e-2	-5.467e+00	0.0192	2312.0	39.32%	16563.3	37.64%	motif file (matrix)	svg
222	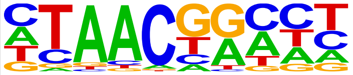	MYB56(MYB)/colamp-MYB56-DAP-Seq(GSE60143)/Homer	1e-2	-5.464e+00	0.0192	1471.0	25.02%	10359.6	23.54%	motif file (matrix)	svg
223	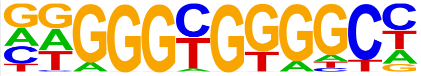	KLF1(Zf)/HUDEP2-KLF1-CutnRun(GSE136251)/Homer	1e-2	-5.455e+00	0.0193	2717.0	46.21%	19577.3	44.49%	motif file (matrix)	svg
224	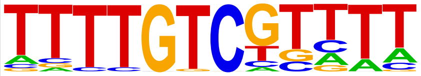	JKD(C2H2)/col-JKD-DAP-Seq(GSE60143)/Homer	1e-2	-5.431e+00	0.0197	373.0	6.34%	2437.7	5.54%	motif file (matrix)	svg
225	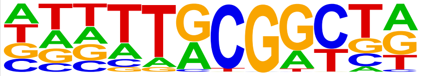	At5g08750(C3H)/col-At5g08750-DAP-Seq(GSE60143)/Homer	1e-2	-5.417e+00	0.0198	916.0	15.58%	6319.2	14.36%	motif file (matrix)	svg
226	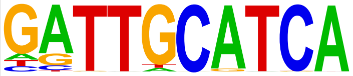	AARE(HLH)/mES-cMyc-ChIP-Seq/Homer	1e-2	-5.417e+00	0.0198	184.0	3.13%	1129.4	2.57%	motif file (matrix)	svg
227	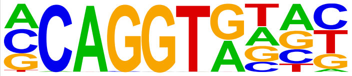	ZEB1(Zf)/PDAC-ZEB1-ChIP-Seq(GSE64557)/Homer	1e-2	-5.393e+00	0.0202	4301.0	73.15%	31509.6	71.61%	motif file (matrix)	svg
228	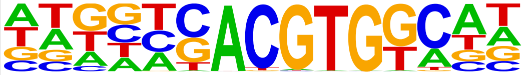	bZIP3(bZIP)/col-bZIP3-DAP-Seq(GSE60143)/Homer	1e-2	-5.287e+00	0.0223	1383.0	23.52%	9729.5	22.11%	motif file (matrix)	svg
229	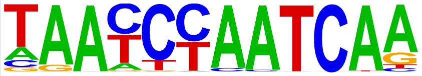	Duxbl(Homeobox)/NIH3T3-Duxbl.HA-ChIP-Seq(GSE119782)/Homer	1e-2	-5.269e+00	0.0226	148.0	2.52%	890.1	2.02%	motif file (matrix)	svg
230	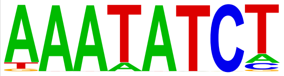	At4g01280(MYBrelated)/colamp-At4g01280-DAP-Seq(GSE60143)/Homer	1e-2	-5.240e+00	0.0232	948.0	16.12%	6563.4	14.92%	motif file (matrix)	svg
231		bZIP48(bZIP)/colamp-bZIP48-DAP-	1e-2	-5.212e+00	0.0237	984.0	16.73%	6826.8	15.52%	motif	svg

	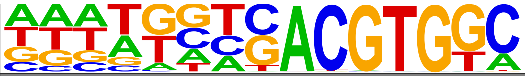	Seq(GSE60143)/Homer								file (matrix)	
232	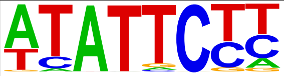	KAN2(G2like)/colamp-KAN2-DAP-Seq(GSE60143)/Homer	1e-2	-5.207e+00	0.0238	2343.0	39.85%	16818.3	38.22%	motif file (matrix)	svg
233	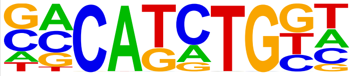	TCF4(bHLH)/SHSY5Y-TCF4-ChIP-Seq(GSE96915)/Homer	1e-2	-5.176e+00	0.0244	3755.0	63.86%	27392.6	62.26%	motif file (matrix)	svg
234	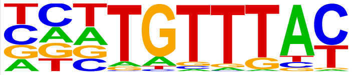	FOXK1(Forkhead)/HEK293-FOXK1-ChIP-Seq(GSE51673)/Homer	1e-2	-5.084e+00	0.0266	1887.0	32.09%	13454.0	30.58%	motif file (matrix)	svg
235	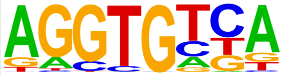	Tbx5(T-box)/HL1-Tbx5.biotin-ChIP-Seq(GSE21529)/Homer	1e-2	-5.045e+00	0.0276	5238.0	89.08%	38734.0	88.03%	motif file (matrix)	svg
236	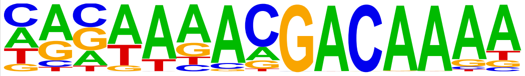	NUC(C2H2)/col-NUC-DAP-Seq(GSE60143)/Homer	1e-2	-4.985e+00	0.0291	206.0	3.50%	1293.9	2.94%	motif file (matrix)	svg
237	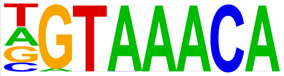	Foxo3(Forkhead)/U2OS-Foxo3-ChIP-Seq(E-MTAB-2701)/Homer	1e-2	-4.982e+00	0.0291	1391.0	23.66%	9813.6	22.30%	motif file (matrix)	svg
238	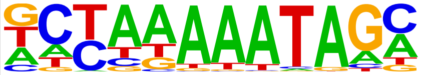	Mef2c(MADS)/GM12878-Mef2c-ChIP-Seq(GSE32465)/Homer	1e-2	-4.965e+00	0.0295	795.0	13.52%	5475.4	12.44%	motif file (matrix)	svg
239	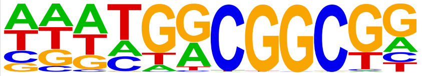	ERF4(AP2EREBP)/colamp-ERF4-DAP-Seq(GSE60143)/Homer	1e-2	-4.889e+00	0.0317	1648.0	28.03%	11709.3	26.61%	motif file (matrix)	svg
240	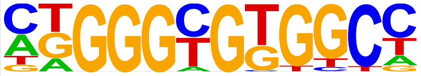	KLF6(Zf)/PDAC-KLF6-ChIP-Seq(GSE64557)/Homer	1e-2	-4.764e+00	0.0358	3182.0	54.12%	23124.1	52.55%	motif file (matrix)	svg
241	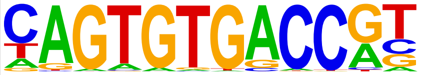	M1BP(Zf)/S2R+-M1BP-ChIP-Seq(GSE49842)/Homer	1e-2	-4.761e+00	0.0358	737.0	12.53%	5070.3	11.52%	motif file (matrix)	svg
242	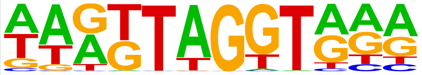	MS188(MYB)/colamp-MS188-DAP-Seq(GSE60143)/Homer	1e-2	-4.756e+00	0.0358	1276.0	21.70%	8990.3	20.43%	motif file (matrix)	svg
243	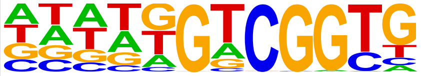	At1g36060(AP2EREBP)/colamp-At1g36060-DAP-Seq(GSE60143)/Homer	1e-2	-4.709e+00	0.0373	1301.0	22.13%	9177.3	20.86%	motif file (matrix)	svg
244	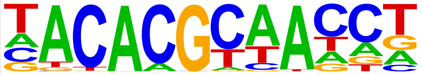	ANAC079(NAC)/colamp-ANAC079-DAP-Seq(GSE60143)/Homer	1e-2	-4.673e+00	0.0385	829.0	14.10%	5741.8	13.05%	motif file (matrix)	svg
245	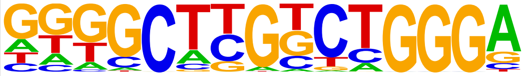	Zfp809(Zf)/ES-Zfp809-ChIP-Seq(GSE70799)/Homer	1e-2	-4.634e+00	0.0399	995.0	16.92%	6949.8	15.79%	motif file (matrix)	svg
246	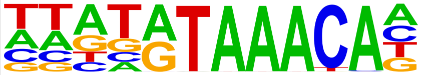	Foxf1(Forkhead)/Lung-Foxf1-ChIP-Seq(GSE77951)/Homer	1e-2	-4.625e+00	0.0401	1671.0	28.42%	11903.3	27.05%	motif file (matrix)	svg
247	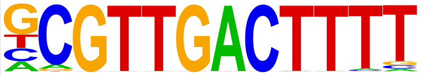	WRKY17(WRKY)/colamp-WRKY17-DAP-Seq(GSE60143)/Homer	1e-2	-4.623e+00	0.0401	22.0	0.37%	95.0	0.22%	motif file (matrix)	svg
