# Supplementary material for: Genome-wide identification of enhancers and transcription factors regulating the myogenic differentiation of bovine satellite cells
Source: BMC Genomics. 2021 Dec 16;22:901. doi: 10.1186/s12864-021-08224-7 (PMC8675486; doi:10.1186/s12864-021-08224-7)
Supplement: Supplementary file 13 — Additional file 13. Motifs enriched in enhancers marked with H3K27ac in both before- and during-differentiation bovine satellite cells [file 12864_2021_8224_MOESM13_ESM.pdf]

Motifs enriched in enhancers marked with H3K27ac in both before- and during-differentiation bovine satellite cells

Rank	Motif	Name	P-value	log P-pvalue	q-value (Benjamini)	# Target Sequences with Motif	% of Targets Sequences with Motif	# Background Sequences with Motif	% of Background Sequences with Motif	Motif File	SVG
1	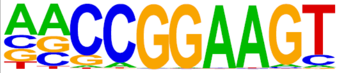	ETS(ETS)/Promoter/Homer	1e-172	-3.962e+02	0.0000	3559.0	26.96%	5206.6	17.18%	motif file (matrix)	svg
2	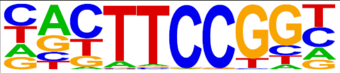	Elk1(ETS)/Hela-Elk1-ChIP-Seq(GSE31477)/Homer	1e-162	-3.744e+02	0.0000	5912.0	44.79%	10109.3	33.36%	motif file (matrix)	svg
3	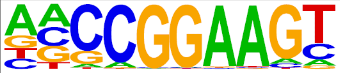	ELF1(ETS)/Jurkat-ELF1-ChIP-Seq(SRA014231)/Homer	1e-161	-3.719e+02	0.0000	5451.0	41.30%	9135.4	30.15%	motif file (matrix)	svg
4	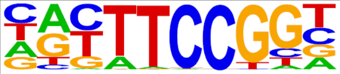	Elk4(ETS)/Hela-Elk4-ChIP-Seq(GSE31477)/Homer	1e-158	-3.653e+02	0.0000	6069.0	45.98%	10490.1	34.62%	motif file (matrix)	svg
5	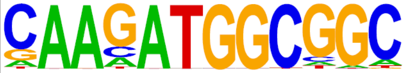	YY1(Zf)/Promoter/Homer	1e-142	-3.274e+02	0.0000	1077.0	8.16%	1041.1	3.44%	motif file (matrix)	svg
6	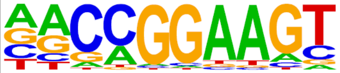	GABPA(ETS)/Jurkat-GABPa-ChIP-Seq(GSE17954)/Homer	1e-114	-2.645e+02	0.0000	6450.0	48.87%	11835.2	39.06%	motif file (matrix)	svg
7	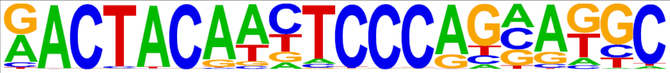	Ronin(THAP)/ES-Thap11-ChIP-Seq(GSE51522)/Homer	1e-111	-2.566e+02	0.0000	563.0	4.27%	426.1	1.41%	motif file (matrix)	svg
8	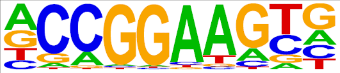	ETV4(ETS)/HepG2-ETV4-ChIP-Seq(ENCODE)/Homer	1e-110	-2.551e+02	0.0000	8345.0	63.22%	16231.5	53.57%	motif file (matrix)	svg
9	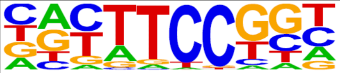	Fli1(ETS)/CD8-FLI-ChIP-Seq(GSE20898)/Homer	1e-96	-2.219e+02	0.0000	7989.0	60.53%	15599.2	51.48%	motif file (matrix)	svg
10	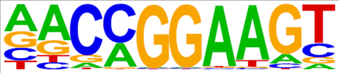	ETV1(ETS)/GIST48-ETV1-ChIP-Seq(GSE22441)/Homer	1e-91	-2.104e+02	0.0000	8316.0	63.00%	16439.5	54.26%	motif file (matrix)	svg
11	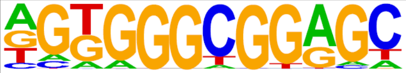	Sp5(Zf)/mES-Sp5.Flag-ChIP-Seq(GSE72989)/Homer	1e-88	-2.046e+02	0.0000	10244.0	77.61%	21157.8	69.83%	motif file (matrix)	svg
12	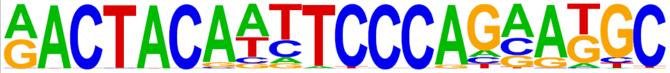	GFY-Staf(?,Zf)/Promoter/Homer	1e-83	-1.919e+02	0.0000	647.0	4.90%	629.3	2.08%	motif file (matrix)	svg
13	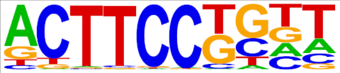	Elf4(ETS)/BMDM-Elf4-ChIP-Seq(GSE88699)/Homer	1e-83	-1.915e+02	0.0000	6389.0	48.41%	12139.9	40.07%	motif file (matrix)	svg
14	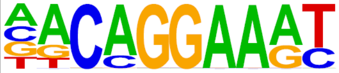	EWS:FLI1-fusion(ETS)/SK_N_MC-EWS:FLI1-ChIP-Seq(SRA014231)/Homer	1e-74	-1.727e+02	0.0000	4105.0	31.10%	7289.9	24.06%	motif file (matrix)	svg
15	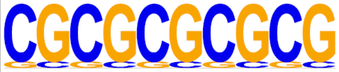	SeqBias: CG-repeat	1e-67	-1.549e+02	0.0000	11042.0	83.66%	23502.4	77.57%	motif file (matrix)	svg

16	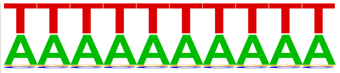	SeqBias: A/T bias	1e-65	-1.502e+02	0.0000	12407.0	94.00%	27209.1	89.80%	motif file (matrix)	svg
17	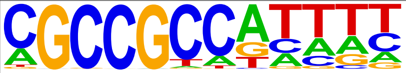	RAP212(AP2EREBP)/col-RAP212-DAP-Seq(GSE60143)/Homer	1e-62	-1.428e+02	0.0000	7420.0	56.22%	14835.5	48.96%	motif file (matrix)	svg
18	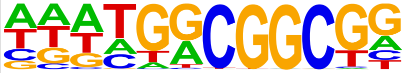	ERF4(AP2EREBP)/colamp-ERF4-DAP-Seq(GSE60143)/Homer	1e-61	-1.412e+02	0.0000	8603.0	65.18%	17611.5	58.12%	motif file (matrix)	svg
19	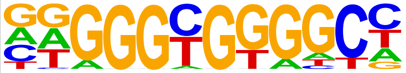	KLF1(Zf)/HUDEP2-KLF1-CutnRun(GSE136251)/Homer	1e-60	-1.391e+02	0.0000	9319.0	70.60%	19338.7	63.83%	motif file (matrix)	svg
20	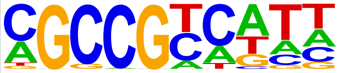	ERF8(AP2EREBP)/colamp-ERF8-DAP-Seq(GSE60143)/Homer	1e-59	-1.369e+02	0.0000	8572.0	64.94%	17572.5	58.00%	motif file (matrix)	svg
21	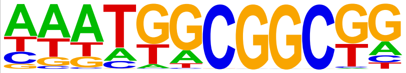	ABR1(AP2EREBP)/colamp-ABR1-DAP-Seq(GSE60143)/Homer	1e-59	-1.366e+02	0.0000	8185.0	62.01%	16663.3	55.00%	motif file (matrix)	svg
22	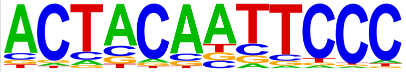	GFY(?)/Promoter/Homer	1e-59	-1.362e+02	0.0000	687.0	5.20%	799.0	2.64%	motif file (matrix)	svg
23	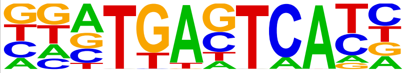	Fos(bZIP)/TSC-Fos-ChIP-Seq(GSE110950)/Homer	1e-58	-1.358e+02	0.0000	2715.0	20.57%	4624.1	15.26%	motif file (matrix)	svg
24	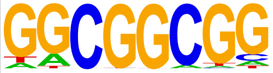	AT1G28160(AP2EREBP)/colamp-AT1G28160-DAP-Seq(GSE60143)/Homer	1e-58	-1.342e+02	0.0000	11087.0	84.00%	23757.7	78.41%	motif file (matrix)	svg
25	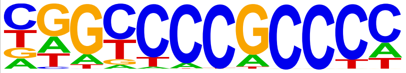	Sp2(Zf)/HEK293-Sp2.eGFP-ChIP-Seq(Encode)/Homer	1e-57	-1.313e+02	0.0000	11629.0	88.11%	25184.8	83.12%	motif file (matrix)	svg
26	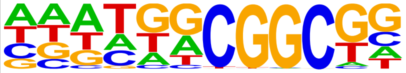	RAP26(AP2EREBP)/colamp-RAP26-DAP-Seq(GSE60143)/Homer	1e-56	-1.311e+02	0.0000	9087.0	68.85%	18844.2	62.19%	motif file (matrix)	svg
27	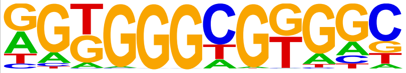	KLF14(Zf)/HEK293-KLF14.GFP-ChIP-Seq(GSE58341)/Homer	1e-56	-1.291e+02	0.0000	12026.0	91.11%	26260.6	86.67%	motif file (matrix)	svg
28	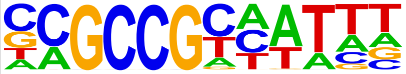	RRTF1(AP2EREBP)/colamp-RRTF1-DAP-Seq(GSE60143)/Homer	1e-55	-1.284e+02	0.0000	2478.0	18.77%	4185.6	13.81%	motif file (matrix)	svg
29	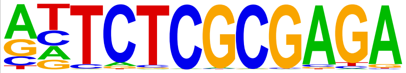	GFX(?)/Promoter/Homer	1e-52	-1.218e+02	0.0000	307.0	2.33%	254.0	0.84%	motif file (matrix)	svg
30	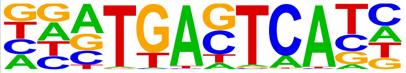	Fra1(bZIP)/BT549-Fra1-ChIP-Seq(GSE46166)/Homer	1e-52	-1.214e+02	0.0000	2574.0	19.50%	4419.2	14.59%	motif file (matrix)	svg
31	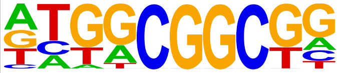	ERF11(AP2EREBP)/col-ERF11-DAP-Seq(GSE60143)/Homer	1e-52	-1.208e+02	0.0000	8087.0	61.27%	16565.9	54.67%	motif file (matrix)	svg
32	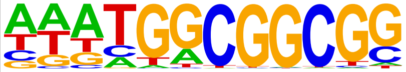	ERF9(AP2EREBP)/colamp-ERF9-DAP-Seq(GSE60143)/Homer	1e-52	-1.202e+02	0.0000	4743.0	35.93%	9013.7	29.75%	motif file (matrix)	svg
33	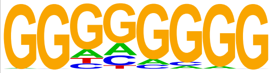	Maz(Zf)/HepG2-Maz-ChIP-Seq(GSE31477)/Homer	1e-52	-1.201e+02	0.0000	11442.0	86.69%	24770.7	81.75%	motif file (matrix)	svg
34	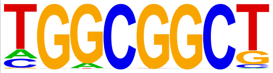	ERF105(AP2EREBP)/colamp-ERF105-DAP-Seq(GSE60143)/Homer	1e-51	-1.195e+02	0.0000	10073.0	76.32%	21328.6	70.39%	motif file (matrix)	svg

35	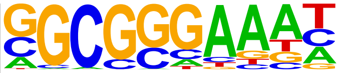	E2F4(E2F)/K562-E2F4-ChIP-Seq(GSE31477)/Homer	1e-51	-1.187e+02	0.0000	5795.0	43.90%	11346.3	37.45%	motif file (matrix)	svg
36	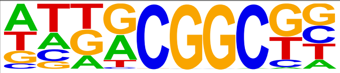	ERF115(AP2EREBP)/colamp-ERF115-DAP-Seq(GSE60143)/Homer	1e-49	-1.146e+02	0.0000	10086.0	76.41%	21400.2	70.63%	motif file (matrix)	svg
37	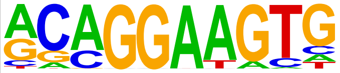	ETS1(ETS)/Jurkat-ETS1-ChIP-Seq(GSE17954)/Homer	1e-49	-1.141e+02	0.0000	6784.0	51.40%	13616.4	44.94%	motif file (matrix)	svg
38	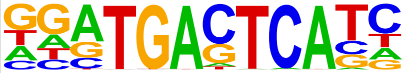	Fra2(bZIP)/Striatum-Fra2-ChIP-Seq(GSE43429)/Homer	1e-49	-1.141e+02	0.0000	2429.0	18.40%	4167.6	13.75%	motif file (matrix)	svg
39	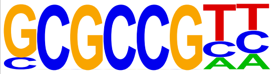	PUCHI(AP2EREBP)/colamp-PUCHI-DAP-Seq(GSE60143)/Homer	1e-49	-1.137e+02	0.0000	7778.0	58.93%	15906.4	52.50%	motif file (matrix)	svg
40	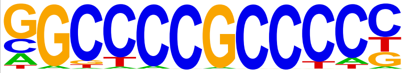	Sp1(Zf)/Promoter/Homer	1e-47	-1.083e+02	0.0000	5344.0	40.49%	10431.1	34.43%	motif file (matrix)	svg
41	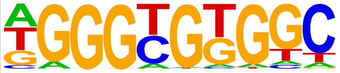	KLF5(Zf)/LoVo-KLF5-ChIP-Seq(GSE49402)/Homer	1e-46	-1.077e+02	0.0000	10671.0	80.85%	22903.8	75.59%	motif file (matrix)	svg
42	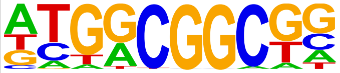	ERF3(AP2EREBP)/colamp-ERF3-DAP-Seq(GSE60143)/Homer	1e-46	-1.069e+02	0.0000	7297.0	55.28%	14855.2	49.03%	motif file (matrix)	svg
43	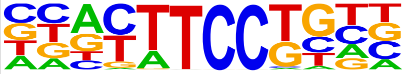	Etv2(ETS)/ES-ER71-ChIP-Seq(GSE59402)/Homer	1e-45	-1.048e+02	0.0000	5965.0	45.19%	11846.4	39.10%	motif file (matrix)	svg
44	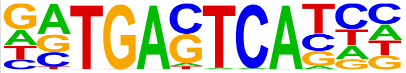	Jun-AP1(bZIP)/K562-cJun-ChIP-Seq(GSE31477)/Homer	1e-44	-1.025e+02	0.0000	1434.0	10.86%	2256.2	7.45%	motif file (matrix)	svg
45	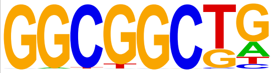	AT5G23930(mTERF)/col-AT5G23930-DAP-Seq(GSE60143)/Homer	1e-44	-1.015e+02	0.0000	11090.0	84.02%	24005.2	79.23%	motif file (matrix)	svg
46	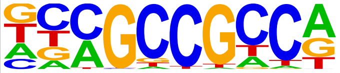	ERF5(AP2EREBP)/colamp-ERF5-DAP-Seq(GSE60143)/Homer	1e-43	-1.010e+02	0.0000	6391.0	48.42%	12841.1	42.38%	motif file (matrix)	svg
47	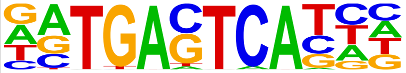	Atf3(bZIP)/GBM-ATF3-ChIP-Seq(GSE33912)/Homer	1e-43	-1.010e+02	0.0000	2950.0	22.35%	5324.1	17.57%	motif file (matrix)	svg
48	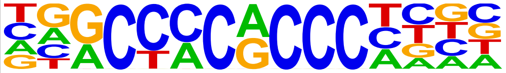	KLF3(Zf)/MEF-Klf3-ChIP-Seq(GSE44748)/Homer	1e-43	-1.010e+02	0.0000	6281.0	47.59%	12593.9	41.56%	motif file (matrix)	svg
49	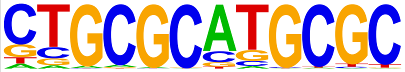	NRF1(NRF)/MCF7-NRF1-ChIP-Seq(Unpublished)/Homer	1e-43	-9.937e+01	0.0000	2257.0	17.10%	3905.9	12.89%	motif file (matrix)	svg
50	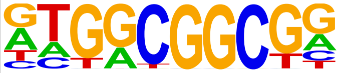	ERF10(AP2EREBP)/col-ERF10-DAP-Seq(GSE60143)/Homer	1e-43	-9.902e+01	0.0000	7416.0	56.19%	15203.3	50.18%	motif file (matrix)	svg
51	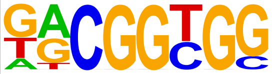	ESE3(AP2EREBP)/col-ESE3-DAP-Seq(GSE60143)/Homer	1e-42	-9.848e+01	0.0000	9544.0	72.31%	20222.2	66.74%	motif file (matrix)	svg
52	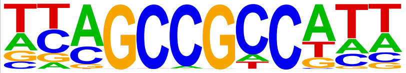	ERF13(AP2EREBP)/colamp-ERF13-DAP-Seq(GSE60143)/Homer	1e-42	-9.824e+01	0.0000	8742.0	66.23%	18309.9	60.43%	motif file (matrix)	svg
53	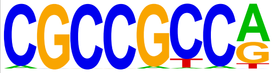	CRF4(AP2EREBP)/colamp-CRF4-DAP-Seq(GSE60143)/Homer	1e-42	-9.766e+01	0.0000	6984.0	52.91%	14223.9	46.94%	motif file (matrix)	svg

54	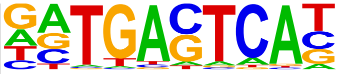	JunB(bZIP)/DendriticCells-Junb-ChIP-Seq(GSE36099)/Homer	1e-42	-9.711e+01	0.0000	2571.0	19.48%	4563.9	15.06%	motif file (matrix)	svg
55	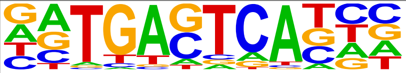	Fosl2(bZIP)/3T3L1-Fosl2-ChIP-Seq(GSE56872)/Homer	1e-42	-9.680e+01	0.0000	1914.0	14.50%	3228.0	10.65%	motif file (matrix)	svg
56	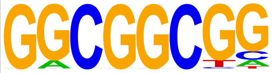	ERF104(AP2EREBP)/col-ERF104-DAP-Seq(GSE60143)/Homer	1e-41	-9.442e+01	0.0000	9304.0	70.49%	19683.0	64.96%	motif file (matrix)	svg
57	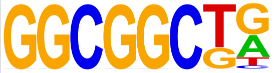	ERF1(AP2EREBP)/colamp-ERF1-DAP-Seq(GSE60143)/Homer	1e-40	-9.315e+01	0.0000	7644.0	57.91%	15787.6	52.11%	motif file (matrix)	svg
58	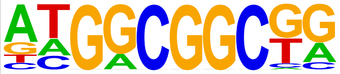	AT4G18450(AP2EREBP)/col-AT4G18450-DAP-Seq(GSE60143)/Homer	1e-40	-9.314e+01	0.0000	6101.0	46.22%	12260.6	40.46%	motif file (matrix)	svg
59	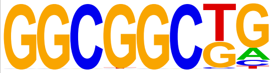	ERF2(AP2EREBP)/colamp-ERF2-DAP-Seq(GSE60143)/Homer	1e-39	-9.105e+01	0.0000	8056.0	61.03%	16766.4	55.34%	motif file (matrix)	svg
60	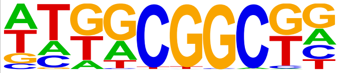	At2g33710(AP2EREBP)/colamp-At2g33710-DAP-Seq(GSE60143)/Homer	1e-39	-9.097e+01	0.0000	10410.0	78.87%	22398.8	73.92%	motif file (matrix)	svg
61	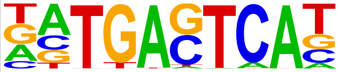	BATF(bZIP)/Th17-BATF-ChIP-Seq(GSE39756)/Homer	1e-39	-9.057e+01	0.0000	2811.0	21.30%	5107.5	16.86%	motif file (matrix)	svg
62	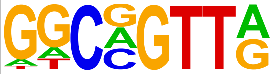	MYB(HTH)/ERMYB-Myb-ChIPSeq(GSE22095)/Homer	1e-38	-8.872e+01	0.0000	7834.0	59.35%	16272.1	53.70%	motif file (matrix)	svg
63	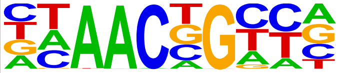	BMYB(HTH)/Hela-BMYB-ChIP-Seq(GSE27030)/Homer	1e-37	-8.734e+01	0.0000	6581.0	49.86%	13404.9	44.24%	motif file (matrix)	svg
64	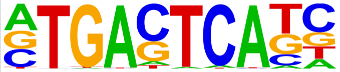	AP-1(bZIP)/ThioMac-PU.1-ChIP-Seq(GSE21512)/Homer	1e-37	-8.698e+01	0.0000	3210.0	24.32%	5975.6	19.72%	motif file (matrix)	svg
65	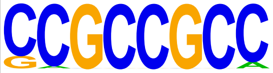	ERF73(AP2EREBP)/col-ERF73-DAP-Seq(GSE60143)/Homer	1e-37	-8.688e+01	0.0000	7927.0	60.06%	16507.7	54.48%	motif file (matrix)	svg
66	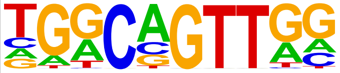	AMYB(HTH)/Testes-AMYB-ChIP-Seq(GSE44588)/Homer	1e-36	-8.496e+01	0.0000	6781.0	51.38%	13884.4	45.82%	motif file (matrix)	svg
67	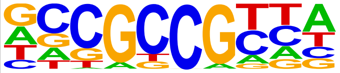	CRF10(AP2EREBP)/col100-CRF10-DAP-Seq(GSE60143)/Homer	1e-36	-8.431e+01	0.0000	9371.0	71.00%	19941.6	65.82%	motif file (matrix)	svg
68	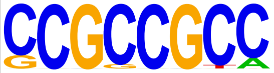	SHN3(AP2EREBP)/col-SHN3-DAP-Seq(GSE60143)/Homer	1e-36	-8.406e+01	0.0000	6007.0	45.51%	12137.3	40.06%	motif file (matrix)	svg
69	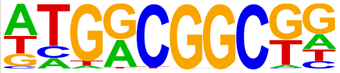	ERF7(AP2EREBP)/col-ERF7-DAP-Seq(GSE60143)/Homer	1e-36	-8.348e+01	0.0000	9237.0	69.98%	19629.8	64.79%	motif file (matrix)	svg
70	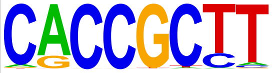	At5g18450(AP2EREBP)/col-At5g18450-DAP-Seq(GSE60143)/Homer	1e-35	-8.222e+01	0.0000	10428.0	79.01%	22522.5	74.33%	motif file (matrix)	svg
71	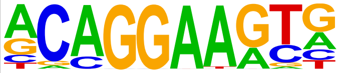	ERG(ETS)/VCaP-ERG-ChIP-Seq(GSE14097)/Homer	1e-35	-8.151e+01	0.0000	8595.0	65.12%	18127.5	59.83%	motif file (matrix)	svg
72	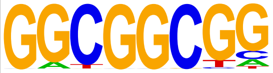	ESE1(AP2EREBP)/col-ESE1-DAP-Seq(GSE60143)/Homer	1e-34	-8.049e+01	0.0000	8403.0	63.66%	17686.3	58.37%	motif file (matrix)	svg

73	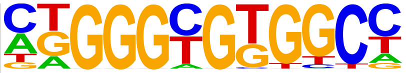	KLF6(Zf)/PDAC-KLF6-ChIP-Seq(GSE64557)/Homer	1e-34	-8.041e+01	0.0000	9829.0	74.47%	21081.9	69.58%	motif file (matrix)	svg
74	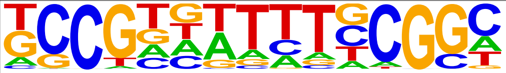	LBD13(LOBAS2)/colamp-LBD13-DAP-Seq(GSE60143)/Homer	1e-34	-7.961e+01	0.0000	7494.0	56.78%	15578.3	51.41%	motif file (matrix)	svg
75	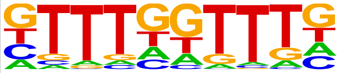	HuR(?)/HEK293-HuR-CLIP-Seq(GSE87887)/Homer	1e-34	-7.908e+01	0.0000	8775.0	66.48%	18577.0	61.31%	motif file (matrix)	svg
76	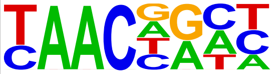	MYB101(MYB)/colamp-MYB101-DAP-Seq(GSE60143)/Homer	1e-34	-7.886e+01	0.0000	6891.0	52.21%	14199.7	46.86%	motif file (matrix)	svg
77	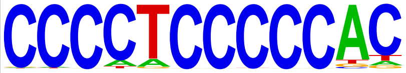	Zfp281(Zf)/ES-Zfp281-ChIP-Seq(GSE81042)/Homer	1e-33	-7.802e+01	0.0000	3741.0	28.34%	7188.9	23.73%	motif file (matrix)	svg
78	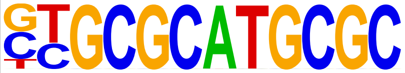	NRF(NRF)/Promoter/Homer	1e-33	-7.632e+01	0.0000	2121.0	16.07%	3776.6	12.46%	motif file (matrix)	svg
79	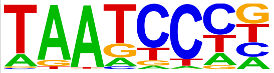	Pitx1(Homeobox)/Chicken-Pitx1-ChIP-Seq(GSE38910)/Homer	1e-32	-7.459e+01	0.0000	10702.0	81.08%	23268.9	76.80%	motif file (matrix)	svg
80	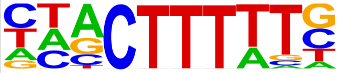	AT1G47655(C2C2dof)/colamp-AT1G47655-DAP-Seq(GSE60143)/Homer	1e-32	-7.400e+01	0.0000	9543.0	72.30%	20458.8	67.52%	motif file (matrix)	svg
81	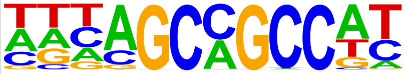	ERF15(AP2EREBP)/colamp-ERF15-DAP-Seq(GSE60143)/Homer	1e-31	-7.236e+01	0.0000	11522.0	87.29%	25339.5	83.63%	motif file (matrix)	svg
82	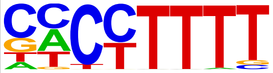	AT5G02460(C2C2dof)/col-AT5G02460-DAP-Seq(GSE60143)/Homer	1e-30	-7.095e+01	0.0000	7401.0	56.07%	15458.6	51.02%	motif file (matrix)	svg
83	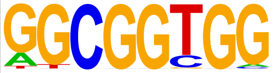	AT3G57600(AP2EREBP)/col-AT3G57600-DAP-Seq(GSE60143)/Homer	1e-30	-7.016e+01	0.0000	8755.0	66.33%	18626.6	61.48%	motif file (matrix)	svg
84	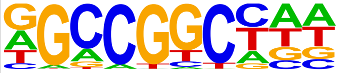	RAP211(AP2EREBP)/colamp-RAP211-DAP-Seq(GSE60143)/Homer	1e-29	-6.822e+01	0.0000	11909.0	90.23%	26365.4	87.02%	motif file (matrix)	svg
85	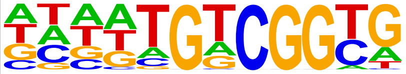	AT1G71450(AP2EREBP)/col-AT1G71450-DAP-Seq(GSE60143)/Homer	1e-29	-6.751e+01	0.0000	9653.0	73.13%	20791.0	68.62%	motif file (matrix)	svg
86	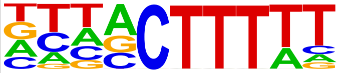	OBP3(C2C2dof)/col-OBP3-DAP-Seq(GSE60143)/Homer	1e-28	-6.627e+01	0.0000	8606.0	65.20%	18319.3	60.46%	motif file (matrix)	svg
87	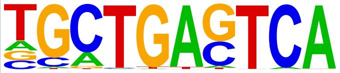	Bach2(bZIP)/OCILy7-Bach2-ChIP-Seq(GSE44420)/Homer	1e-28	-6.513e+01	0.0000	1211.0	9.17%	2010.5	6.64%	motif file (matrix)	svg
88	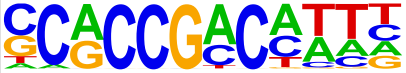	At5g65130(AP2EREBP)/colamp-At5g65130-DAP-Seq(GSE60143)/Homer	1e-28	-6.507e+01	0.0000	2893.0	21.92%	5483.9	18.10%	motif file (matrix)	svg
89	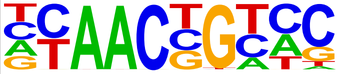	MYB77(MYB)/col-MYB77-DAP-Seq(GSE60143)/Homer	1e-27	-6.274e+01	0.0000	5700.0	43.19%	11675.7	38.53%	motif file (matrix)	svg
90	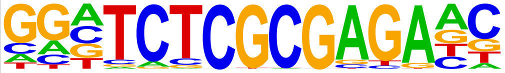	ZBTB33(Zf)/GM12878-ZBTB33-ChIP-Seq(GSE32465)/Homer	1e-27	-6.253e+01	0.0000	779.0	5.90%	1190.8	3.93%	motif file (matrix)	svg
91	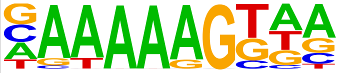	AT2G28810(C2C2dof)/colamp-AT2G28810-DAP-Seq(GSE60143)/Homer	1e-27	-6.245e+01	0.0000	7676.0	58.16%	16193.1	53.44%	motif file (matrix)	svg

92	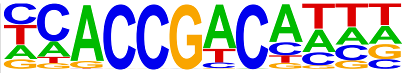	DEAR2(AP2EREBP)/colamp-DEAR2-DAP-Seq(GSE60143)/Homer	1e-26	-5.991e+01	0.0000	7088.0	53.70%	14867.3	49.07%	motif file (matrix)	svg
93	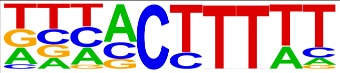	AT3G52440(C2C2dof)/colamp-AT3G52440-DAP-Seq(GSE60143)/Homer	1e-25	-5.932e+01	0.0000	7094.0	53.75%	14888.7	49.14%	motif file (matrix)	svg
94	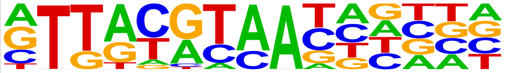	NFIL3(bZIP)/HepG2-NFIL3-ChIP-Seq(Encode)/Homer	1e-25	-5.868e+01	0.0000	1862.0	14.11%	3369.4	11.12%	motif file (matrix)	svg
95	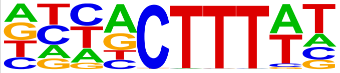	OBP1(C2C2dof)/col-OBP1-DAP-Seq(GSE60143)/Homer	1e-25	-5.853e+01	0.0000	7888.0	59.76%	16733.1	55.23%	motif file (matrix)	svg
96	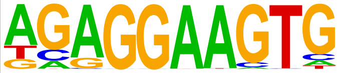	PU.1(ETS)/ThioMac-PU.1-ChIP-Seq(GSE21512)/Homer	1e-24	-5.741e+01	0.0000	2799.0	21.21%	5352.2	17.66%	motif file (matrix)	svg
97	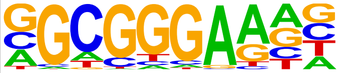	E2F6(E2F)/Hela-E2F6-ChIP-Seq(GSE31477)/Homer	1e-24	-5.733e+01	0.0000	6471.0	49.03%	13487.3	44.51%	motif file (matrix)	svg
98	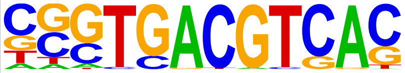	CRE(bZIP)/Promoter/Homer	1e-24	-5.712e+01	0.0000	1864.0	14.12%	3385.6	11.17%	motif file (matrix)	svg
99	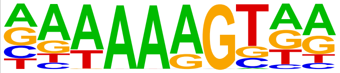	AT5G63260(C3H)/col-AT5G63260-DAP-Seq(GSE60143)/Homer	1e-24	-5.672e+01	0.0000	7402.0	56.08%	15630.5	51.59%	motif file (matrix)	svg
100	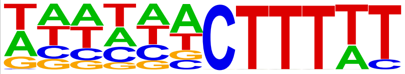	At5g62940(C2C2dof)/col-At5g62940-DAP-Seq(GSE60143)/Homer	1e-24	-5.605e+01	0.0000	9334.0	70.72%	20161.2	66.54%	motif file (matrix)	svg
101	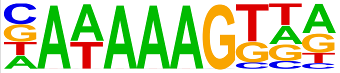	dof43(C2C2dof)/colamp-dof43-DAP-Seq(GSE60143)/Homer	1e-24	-5.601e+01	0.0000	4910.0	37.20%	9984.5	32.95%	motif file (matrix)	svg
102	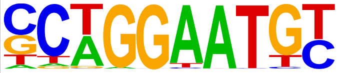	TEAD2(TEA)/Py2T-Tead2-ChIP-Seq(GSE55709)/Homer	1e-24	-5.583e+01	0.0000	2329.0	17.65%	4368.3	14.42%	motif file (matrix)	svg
103	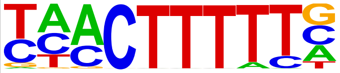	AT3G12130(C3H)/colamp-AT3G12130-DAP-Seq(GSE60143)/Homer	1e-24	-5.560e+01	0.0000	8005.0	60.65%	17042.0	56.25%	motif file (matrix)	svg
104	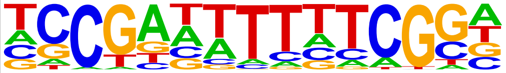	LBD2(LOBAS2)/colamp-LBD2-DAP-Seq(GSE60143)/Homer	1e-24	-5.556e+01	0.0000	2326.0	17.62%	4364.3	14.40%	motif file (matrix)	svg
105	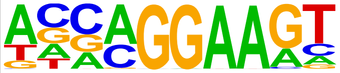	EHF(ETS)/LoVo-EHF-ChIP-Seq(GSE49402)/Homer	1e-23	-5.521e+01	0.0000	6813.0	51.62%	14295.7	47.18%	motif file (matrix)	svg
106	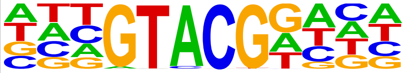	SPL5(SBP)/colamp-SPL5-DAP-Seq(GSE60143)/Homer	1e-23	-5.518e+01	0.0000	2152.0	16.30%	4001.3	13.21%	motif file (matrix)	svg
107	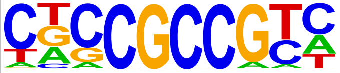	LEP(AP2EREBP)/col-LEP-DAP-Seq(GSE60143)/Homer	1e-23	-5.491e+01	0.0000	5216.0	39.52%	10681.0	35.25%	motif file (matrix)	svg
108	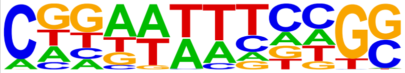	LBD18(LOBAS2)/colamp-LBD18-DAP-Seq(GSE60143)/Homer	1e-23	-5.482e+01	0.0000	9113.0	69.04%	19652.2	64.86%	motif file (matrix)	svg
109	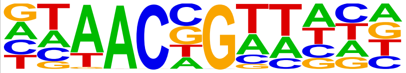	MYB81(MYB)/col-MYB81-DAP-Seq(GSE60143)/Homer	1e-23	-5.454e+01	0.0000	4838.0	36.65%	9841.8	32.48%	motif file (matrix)	svg
110	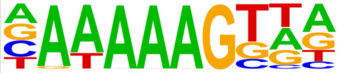	BBX31(Orphan)/col-BBX31-DAP-Seq(GSE60143)/Homer	1e-23	-5.404e+01	0.0000	5296.0	40.12%	10872.0	35.88%	motif file (matrix)	svg

111	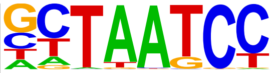	CRX(Homeobox)/Retina-Crx-ChIP-Seq(GSE20012)/Homer	1e-23	-5.373e+01	0.0000	6953.0	52.68%	14635.7	48.30%	motif file (matrix)	svg
112	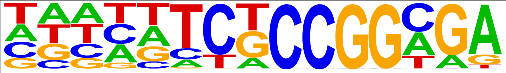	AT5G05550(Trihelix)/col-AT5G05550-DAP-Seq(GSE60143)/Homer	1e-22	-5.245e+01	0.0000	9282.0	70.32%	20082.8	66.28%	motif file (matrix)	svg
113	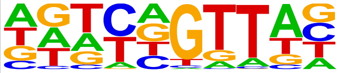	MYB33(MYB)/col-MYB33-DAP-Seq(GSE60143)/Homer	1e-22	-5.237e+01	0.0000	4988.0	37.79%	10202.2	33.67%	motif file (matrix)	svg
114	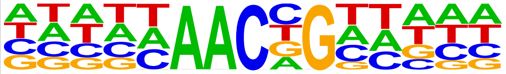	MYB73(MYB)/col-MYB73-DAP-Seq(GSE60143)/Homer	1e-22	-5.192e+01	0.0000	5492.0	41.61%	11338.2	37.42%	motif file (matrix)	svg
115	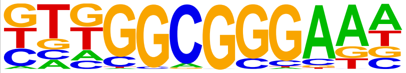	E2F3(E2F)/MEF-E2F3-ChIP-Seq(GSE71376)/Homer	1e-22	-5.190e+01	0.0000	7221.0	54.71%	15275.3	50.41%	motif file (matrix)	svg
116	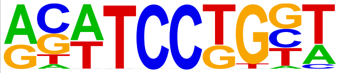	SPDEF(ETS)/VCaP-SPDEF-ChIP-Seq(SRA014231)/Homer	1e-22	-5.184e+01	0.0000	5607.0	42.48%	11598.5	38.28%	motif file (matrix)	svg
117	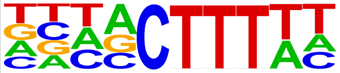	AT5G66940(C2C2dof)/col-AT5G66940-DAP-Seq(GSE60143)/Homer	1e-22	-5.162e+01	0.0000	6126.0	46.41%	12775.1	42.16%	motif file (matrix)	svg
118	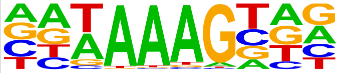	Adof1(C2C2dof)/col-Adof1-DAP-Seq(GSE60143)/Homer	1e-22	-5.131e+01	0.0000	8212.0	62.22%	17580.5	58.02%	motif file (matrix)	svg
119	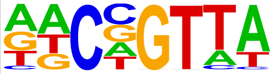	MYB65(MYB)/colamp-MYB65-DAP-Seq(GSE60143)/Homer	1e-22	-5.088e+01	0.0000	4008.0	30.37%	8046.2	26.56%	motif file (matrix)	svg
120	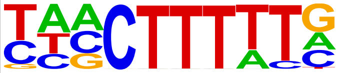	dof24(C2C2dof)/col-dof24-DAP-Seq(GSE60143)/Homer	1e-21	-4.991e+01	0.0000	7059.0	53.48%	14930.0	49.27%	motif file (matrix)	svg
121	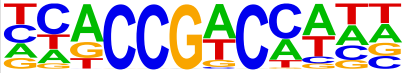	At1g19210(AP2EREBP)/colamp-At1g19210-DAP-Seq(GSE60143)/Homer	1e-21	-4.987e+01	0.0000	8638.0	65.44%	18596.4	61.38%	motif file (matrix)	svg
122	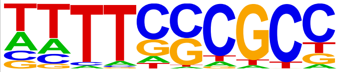	DEL2(E2FDP)/col-DEL2-DAP-Seq(GSE60143)/Homer	1e-21	-4.934e+01	0.0000	3412.0	25.85%	6758.1	22.30%	motif file (matrix)	svg
123	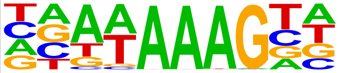	dof45(C2C2dof)/col-dof45-DAP-Seq(GSE60143)/Homer	1e-21	-4.924e+01	0.0000	7314.0	55.41%	15525.4	51.24%	motif file (matrix)	svg
124	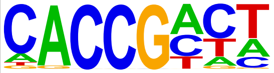	At1g75490(AP2EREBP)/colamp-At1g75490-DAP-Seq(GSE60143)/Homer	1e-20	-4.773e+01	0.0000	10103.0	76.54%	22101.3	72.94%	motif file (matrix)	svg
125	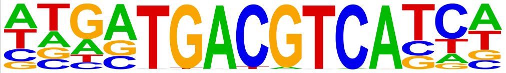	TGA3(bZIP)/colamp-TGA3-DAP-Seq(GSE60143)/Homer	1e-19	-4.580e+01	0.0000	389.0	2.95%	540.5	1.78%	motif file (matrix)	svg
126	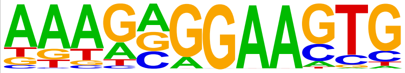	SpiB(ETS)/OCILY3-SPIB-ChIP-Seq(GSE56857)/Homer	1e-19	-4.565e+01	0.0000	1341.0	10.16%	2395.4	7.91%	motif file (matrix)	svg
127	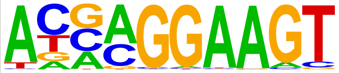	ELF5(ETS)/T47D-ELF5-ChIP-Seq(GSE30407)/Homer	1e-19	-4.515e+01	0.0000	4341.0	32.89%	8853.0	29.22%	motif file (matrix)	svg
128	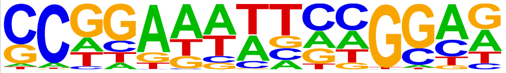	ASL18(LOBAS2)/colamp-ASL18-DAP-Seq(GSE60143)/Homer	1e-19	-4.479e+01	0.0000	9235.0	69.97%	20071.2	66.24%	motif file (matrix)	svg
129	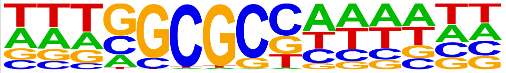	E2FA(E2FDP)/colamp-E2FA-DAP-Seq(GSE60143)/Homer	1e-19	-4.467e+01	0.0000	3698.0	28.02%	7438.3	24.55%	motif file (matrix)	svg

130	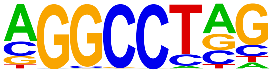	ZNF711(Zf)/SHSY5Y-ZNF711-ChIP-Seq(GSE20673)/Homer	1e-19	-4.457e+01	0.0000	11390.0	86.29%	25274.0	83.41%	motif file (matrix)	svg
131	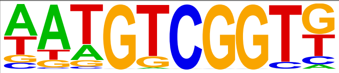	DREB19(AP2EREBP)/colamp-DREB19-DAP-Seq(GSE60143)/Homer	1e-19	-4.395e+01	0.0000	3858.0	29.23%	7799.6	25.74%	motif file (matrix)	svg
132	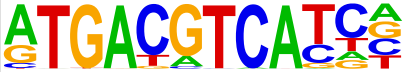	JunD(bZIP)/K562-JunD-ChIP-Seq/Homer	1e-18	-4.315e+01	0.0000	537.0	4.07%	821.5	2.71%	motif file (matrix)	svg
133	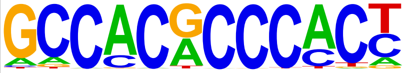	Klf9(Zf)/GBM-Klf9-ChIP-Seq(GSE62211)/Homer	1e-18	-4.295e+01	0.0000	5255.0	39.81%	10924.1	36.05%	motif file (matrix)	svg
134	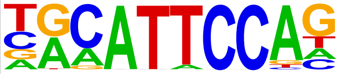	TEAD3(TEA)/HepG2-TEAD3-ChIP-Seq(Encode)/Homer	1e-18	-4.287e+01	0.0000	4361.0	33.04%	8926.1	29.46%	motif file (matrix)	svg
135	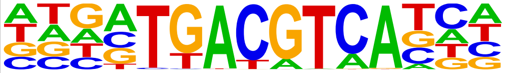	TGA5(bZIP)/col-TGA5-DAP-Seq(GSE60143)/Homer	1e-18	-4.247e+01	0.0000	529.0	4.01%	809.1	2.67%	motif file (matrix)	svg
136	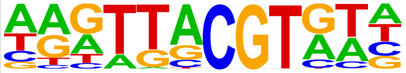	NAP(NAC)/col-NAP-DAP-Seq(GSE60143)/Homer	1e-18	-4.246e+01	0.0000	4032.0	30.55%	8202.5	27.07%	motif file (matrix)	svg
137	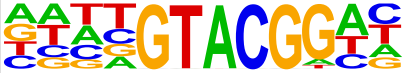	bHLH28(bHLH)/col-bHLH28-DAP-Seq(GSE60143)/Homer	1e-18	-4.226e+01	0.0000	1313.0	9.95%	2363.2	7.80%	motif file (matrix)	svg
138	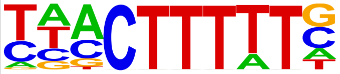	At3g45610(C2C2dof)/col-At3g45610-DAP-Seq(GSE60143)/Homer	1e-18	-4.215e+01	0.0000	5105.0	38.68%	10598.8	34.98%	motif file (matrix)	svg
139	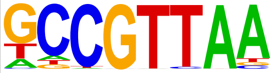	AT3G10030(Trihelix)/colamp-AT3G10030-DAP-Seq(GSE60143)/Homer	1e-18	-4.213e+01	0.0000	2546.0	19.29%	4962.1	16.38%	motif file (matrix)	svg
140	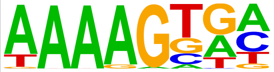	CDF3(C2C2dof)/colamp-CDF3-DAP-Seq(GSE60143)/Homer	1e-18	-4.178e+01	0.0000	5958.0	45.14%	12529.5	41.35%	motif file (matrix)	svg
141	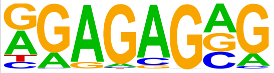	Trl(Zf)/S2-GAGAfactor-ChIP-Seq(GSE40646)/Homer	1e-18	-4.167e+01	0.0000	11880.0	90.01%	26528.1	87.55%	motif file (matrix)	svg
142	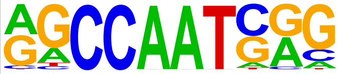	NFY(CCAAT)/Promoter/Homer	1e-18	-4.152e+01	0.0000	3469.0	26.28%	6974.1	23.02%	motif file (matrix)	svg
143	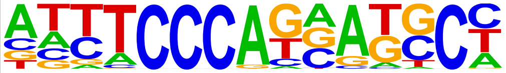	ZNF143|STAF(Zf)/CUTLL-ZNF143-ChIP-Seq(GSE29600)/Homer	1e-17	-4.134e+01	0.0000	2476.0	18.76%	4820.7	15.91%	motif file (matrix)	svg
144	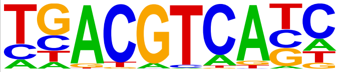	TGA6(bZIP)/colamp-TGA6-DAP-Seq(GSE60143)/Homer	1e-17	-4.122e+01	0.0000	2938.0	22.26%	5819.9	19.21%	motif file (matrix)	svg
145	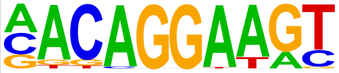	Ets1-distal(ETS)/CD4+-PolII-ChIP-Seq(Barski_et_al.)/Homer	1e-17	-4.085e+01	0.0000	1597.0	12.10%	2962.1	9.78%	motif file (matrix)	svg
146	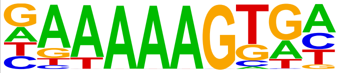	COG1(C2C2dof)/col-COG1-DAP-Seq(GSE60143)/Homer	1e-17	-4.082e+01	0.0000	5215.0	39.51%	10864.2	35.86%	motif file (matrix)	svg
147	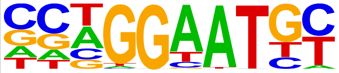	TEAD4(TEA)/Tropoblast-Tead4-ChIP-Seq(GSE37350)/Homer	1e-17	-4.056e+01	0.0000	3934.0	29.81%	8010.4	26.44%	motif file (matrix)	svg
148	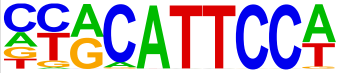	TEAD1(TEAD)/HepG2-TEAD1-ChIP-Seq(Encode)/Homer	1e-16	-3.891e+01	0.0000	4056.0	30.73%	8302.4	27.40%	motif file (matrix)	svg

149	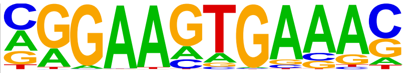	PU.1-IRF(ETS:IRF)/Bcell-PU.1-ChIP-Seq(GSE21512)/Homer	1e-16	-3.869e+01	0.0000	6185.0	46.86%	13091.2	43.21%	motif file (matrix)	svg
150	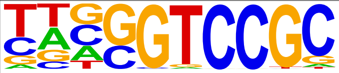	HINFP(Zf)/K562-HINFP.eGFP-ChIP-Seq(Encode)/Homer	1e-16	-3.856e+01	0.0000	4813.0	36.46%	9994.3	32.99%	motif file (matrix)	svg
151	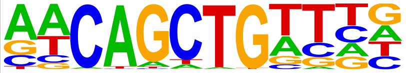	E-box/Drosophila-Promoters/Homer	1e-16	-3.827e+01	0.0000	1326.0	10.05%	2421.9	7.99%	motif file (matrix)	svg
152	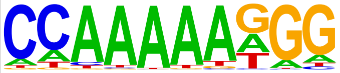	SEP3(MADS)/Arabidoposis-Flower-Sep3-ChIP-Seq/Homer	1e-16	-3.793e+01	0.0000	4377.0	33.16%	9029.6	29.80%	motif file (matrix)	svg
153	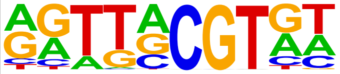	NAM(NAC)/col-NAM-DAP-Seq(GSE60143)/Homer	1e-16	-3.724e+01	0.0000	5487.0	41.57%	11530.3	38.05%	motif file (matrix)	svg
154	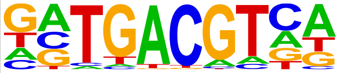	bZIP50(bZIP)/colamp-bZIP50-DAP-Seq(GSE60143)/Homer	1e-16	-3.716e+01	0.0000	4369.0	33.10%	9022.2	29.78%	motif file (matrix)	svg
155	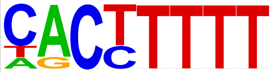	At1g64620(C2C2dof)/colamp-At1g64620-DAP-Seq(GSE60143)/Homer	1e-16	-3.712e+01	0.0000	5309.0	40.22%	11130.4	36.73%	motif file (matrix)	svg
156	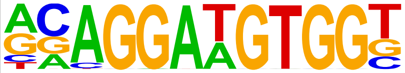	ETS:RUNX(ETS,Runt)/Jurkat-RUNX1-ChIP-Seq(GSE17954)/Homer	1e-15	-3.622e+01	0.0000	841.0	6.37%	1449.8	4.78%	motif file (matrix)	svg
157	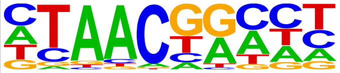	MYB56(MYB)/colamp-MYB56-DAP-Seq(GSE60143)/Homer	1e-15	-3.610e+01	0.0000	3047.0	23.09%	6118.7	20.19%	motif file (matrix)	svg
158	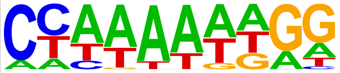	RIN(MADS)/Tomato-RIN-ChIP-Seq(GSE116581)/Homer	1e-15	-3.607e+01	0.0000	3833.0	29.04%	7847.0	25.90%	motif file (matrix)	svg
159	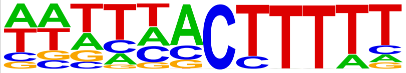	DAG2(C2C2dof)/col-DAG2-DAP-Seq(GSE60143)/Homer	1e-15	-3.594e+01	0.0000	4922.0	37.29%	10277.4	33.92%	motif file (matrix)	svg
160	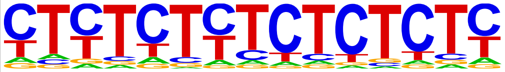	GAGA-repeat/SacCer-Promoters/Homer	1e-15	-3.578e+01	0.0000	11592.0	87.82%	25873.5	85.39%	motif file (matrix)	svg
161	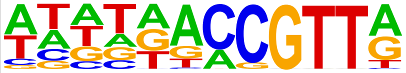	MYB3R1(MYB)/col-MYB3R1-DAP-Seq(GSE60143)/Homer	1e-15	-3.499e+01	0.0000	1206.0	9.14%	2200.9	7.26%	motif file (matrix)	svg
162	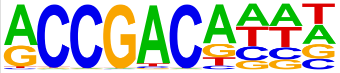	AT1G77200(AP2EREBP)/colamp-AT1G77200-DAP-Seq(GSE60143)/Homer	1e-14	-3.437e+01	0.0000	5011.0	37.96%	10501.7	34.66%	motif file (matrix)	svg
163	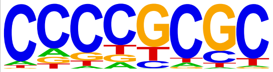	SUT1?/SacCer-Promoters/Homer	1e-14	-3.402e+01	0.0000	13196.0	99.98%	30199.6	99.67%	motif file (matrix)	svg
164	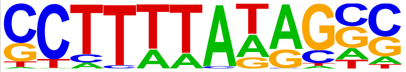	TATA-Box(TBP)/Promoter/Homer	1e-14	-3.396e+01	0.0000	4409.0	33.40%	9159.0	30.23%	motif file (matrix)	svg
165	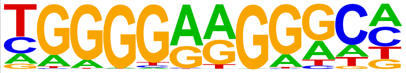	ZNF467(Zf)/HEK293-ZNF467.GFP-ChIP-Seq(GSE58341)/Homer	1e-14	-3.391e+01	0.0000	8212.0	62.22%	17834.7	58.86%	motif file (matrix)	svg
166	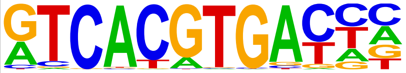	TFE3(bHLH)/MEF-TFE3-ChIP-Seq(GSE75757)/Homer	1e-14	-3.386e+01	0.0000	627.0	4.75%	1040.8	3.44%	motif file (matrix)	svg
167	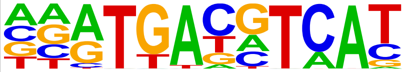	CREB5(bZIP)/LNCaP-CREB5.V5-ChIP-Seq(GSE137775)/Homer	1e-14	-3.369e+01	0.0000	1467.0	11.11%	2754.6	9.09%	motif file (matrix)	svg

168	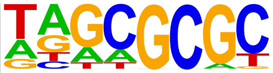	DPL-1(E2F)/cElegans-Adult-ChIP-Seq(modEncode)/Homer	1e-14	-3.264e+01	0.0000	7049.0	53.41%	15165.1	50.05%	motif file (matrix)	svg
169	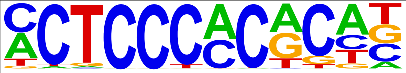	WT1(Zf)/Kidney-WT1-ChIP-Seq(GSE90016)/Homer	1e-14	-3.248e+01	0.0000	6584.0	49.88%	14101.3	46.54%	motif file (matrix)	svg
170	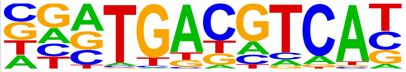	Atf2(bZIP)/3T3L1-Atf2-ChIP-Seq(GSE56872)/Homer	1e-13	-3.168e+01	0.0000	1675.0	12.69%	3212.5	10.60%	motif file (matrix)	svg
171	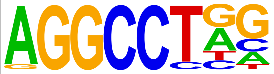	ZFX(Zf)/mES-Zfx-ChIP-Seq(GSE11431)/Homer	1e-13	-3.097e+01	0.0000	9156.0	69.37%	20091.8	66.31%	motif file (matrix)	svg
172	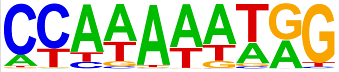	TAGL1(MADS)/Tomato-TAGL1-ChIP-Seq(GSE116581)/Homer	1e-13	-3.091e+01	0.0000	3477.0	26.34%	7134.0	23.54%	motif file (matrix)	svg
173	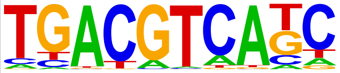	TGA1(bZIP)/colamp-TGA1-DAP-Seq(GSE60143)/Homer	1e-13	-3.087e+01	0.0000	1895.0	14.36%	3689.3	12.18%	motif file (matrix)	svg
174	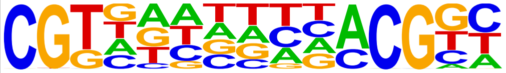	ANAC042(NAC)/col-ANAC042-DAP-Seq(GSE60143)/Homer	1e-13	-2.998e+01	0.0000	6438.0	48.78%	13810.8	45.58%	motif file (matrix)	svg
175	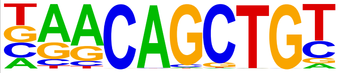	Myf5(bHLH)/GM-Myf5-ChIP-Seq(GSE24852)/Homer	1e-12	-2.984e+01	0.0000	4243.0	32.15%	8852.0	29.22%	motif file (matrix)	svg
176	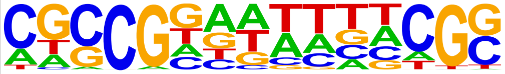	LOB(LOBAS2)/col-LOB-DAP-Seq(GSE60143)/Homer	1e-12	-2.972e+01	0.0000	3253.0	24.65%	6656.1	21.97%	motif file (matrix)	svg
177	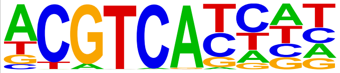	TGA2(bZIP)/colamp-TGA2-DAP-Seq(GSE60143)/Homer	1e-12	-2.949e+01	0.0000	3112.0	23.58%	6349.6	20.96%	motif file (matrix)	svg
178	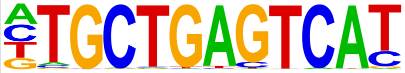	Nrf2(bZIP)/Lymphoblast-Nrf2-ChIP-Seq(GSE37589)/Homer	1e-12	-2.904e+01	0.0000	300.0	2.27%	441.2	1.46%	motif file (matrix)	svg
179	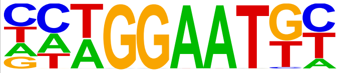	TEAD(TEA)/Fibroblast-PU.1-ChIP-Seq(Unpublished)/Homer	1e-12	-2.903e+01	0.0000	2520.0	19.09%	5060.0	16.70%	motif file (matrix)	svg
180	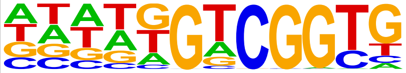	At1g36060(AP2EREBP)/colamp-At1g36060-DAP-Seq(GSE60143)/Homer	1e-12	-2.881e+01	0.0000	4803.0	36.39%	10123.1	33.41%	motif file (matrix)	svg
181	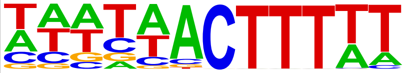	At4g38000(C2C2dof)/col-At4g38000-DAP-Seq(GSE60143)/Homer	1e-12	-2.873e+01	0.0000	3265.0	24.74%	6697.5	22.10%	motif file (matrix)	svg
182	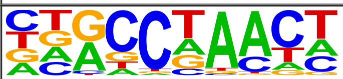	BOS1(MYB)/col-BOS1-DAP-Seq(GSE60143)/Homer	1e-12	-2.856e+01	0.0000	3608.0	27.34%	7458.1	24.61%	motif file (matrix)	svg
183	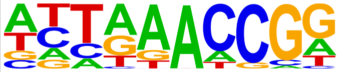	Unknown3/Arabidopsis-Promoters/Homer	1e-12	-2.842e+01	0.0000	1186.0	8.99%	2215.1	7.31%	motif file (matrix)	svg
184	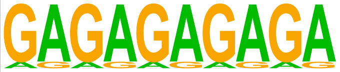	SeqBias: GA-repeat	1e-12	-2.802e+01	0.0000	13148.0	99.61%	30023.6	99.09%	motif file (matrix)	svg
185	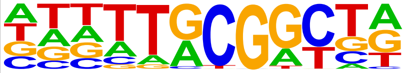	At5g08750(C3H)/col-At5g08750-DAP-Seq(GSE60143)/Homer	1e-12	-2.799e+01	0.0000	3039.0	23.02%	6211.0	20.50%	motif file (matrix)	svg
186	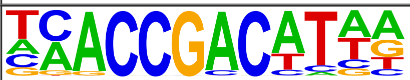	DREB2(AP2EREBP)/col-DREB2-DAP-Seq(GSE60143)/Homer	1e-12	-2.773e+01	0.0000	3060.0	23.18%	6260.9	20.66%	motif file (matrix)	svg

187	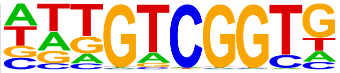	CEJ1(AP2EREBP)/col-CEJ1-DAP-Seq(GSE60143)/Homer	1e-11	-2.748e+01	0.0000	6213.0	47.07%	13341.3	44.03%	motif file (matrix)	svg
188	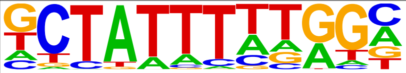	Mef2b(MADS)/HEK293-Mef2b.V5-ChIP-Seq(GSE67450)/Homer	1e-11	-2.733e+01	0.0000	2386.0	18.08%	4791.3	15.81%	motif file (matrix)	svg
189	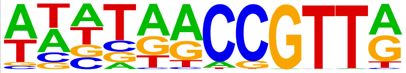	MYB3R4(MYB)/col-MYB3R4-DAP-Seq(GSE60143)/Homer	1e-11	-2.730e+01	0.0000	745.0	5.64%	1317.4	4.35%	motif file (matrix)	svg
190	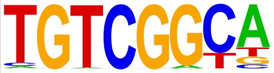	Rap210(AP2EREBP)/col-Rap210-DAP-Seq(GSE60143)/Homer	1e-11	-2.723e+01	0.0000	3988.0	30.21%	8324.3	27.47%	motif file (matrix)	svg
191	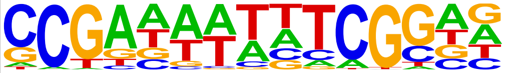	CDM1(C3H)/colamp-CDM1-DAP-Seq(GSE60143)/Homer	1e-11	-2.723e+01	0.0000	797.0	6.04%	1423.5	4.70%	motif file (matrix)	svg
192	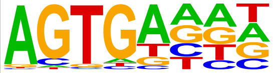	At5g04390(C2H2)/col200-At5g04390-DAP-Seq(GSE60143)/Homer	1e-11	-2.713e+01	0.0000	12266.0	92.93%	27653.1	91.27%	motif file (matrix)	svg
193	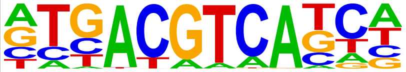	TGA4(bZIP)/colamp-TGA4-DAP-Seq(GSE60143)/Homer	1e-11	-2.695e+01	0.0000	1340.0	10.15%	2552.6	8.42%	motif file (matrix)	svg
194	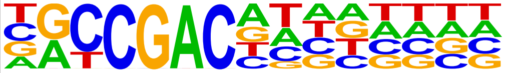	CBF4(AP2EREBP)/colamp-CBF4-DAP-Seq(GSE60143)/Homer	1e-11	-2.688e+01	0.0000	4601.0	34.86%	9702.3	32.02%	motif file (matrix)	svg
195	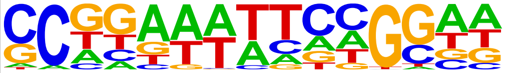	LBD19(LOBAS2)/colamp-LBD19-DAP-Seq(GSE60143)/Homer	1e-11	-2.656e+01	0.0000	8046.0	60.96%	17576.1	58.01%	motif file (matrix)	svg
196	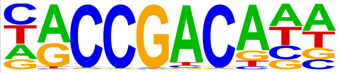	At4g16750(AP2EREBP)/col-At4g16750-DAP-Seq(GSE60143)/Homer	1e-11	-2.650e+01	0.0000	4712.0	35.70%	9958.2	32.87%	motif file (matrix)	svg
197	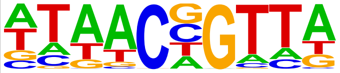	MYB70(MYB)/col-MYB70-DAP-Seq(GSE60143)/Homer	1e-11	-2.648e+01	0.0000	4334.0	32.84%	9110.8	30.07%	motif file (matrix)	svg
198	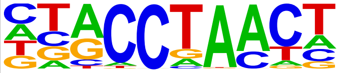	MYB62(MYB)/colamp-MYB62-DAP-Seq(GSE60143)/Homer	1e-11	-2.608e+01	0.0000	5073.0	38.43%	10779.7	35.58%	motif file (matrix)	svg
199	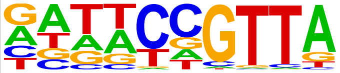	MYB105(MYB)/colamp-MYB105-DAP-Seq(GSE60143)/Homer	1e-11	-2.598e+01	0.0000	1967.0	14.90%	3901.2	12.88%	motif file (matrix)	svg
200	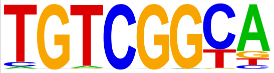	AT1G12630(AP2EREBP)/colamp-AT1G12630-DAP-Seq(GSE60143)/Homer	1e-11	-2.576e+01	0.0000	3269.0	24.77%	6751.1	22.28%	motif file (matrix)	svg
201	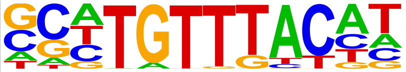	FOXK2(Forkhead)/U2OS-FOXK2-ChIP-Seq(E-MTAB-2204)/Homer	1e-11	-2.573e+01	0.0000	2193.0	16.61%	4393.0	14.50%	motif file (matrix)	svg
202	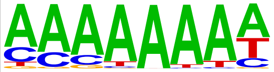	REM19(REM)/colamp-REM19-DAP-Seq(GSE60143)/Homer	1e-11	-2.565e+01	0.0000	2606.0	19.74%	5294.2	17.47%	motif file (matrix)	svg
203	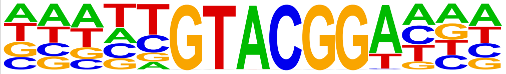	SPL14(SBP)/col-SPL14-DAP-Seq(GSE60143)/Homer	1e-11	-2.537e+01	0.0000	813.0	6.16%	1470.2	4.85%	motif file (matrix)	svg
204	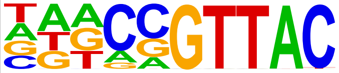	MYB98(MYB)/col-MYB98-DAP-Seq(GSE60143)/Homer	1e-10	-2.529e+01	0.0000	1169.0	8.86%	2209.8	7.29%	motif file (matrix)	svg
205	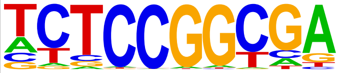	AT3G58630(Trihelix)/col-AT3G58630-DAP-Seq(GSE60143)/Homer	1e-10	-2.509e+01	0.0000	2271.0	17.21%	4571.3	15.09%	motif file (matrix)	svg

206	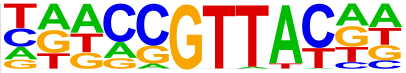	MYB119(MYB)/colamp-MYB119-DAP-Seq(GSE60143)/Homer	1e-10	-2.504e+01	0.0000	1144.0	8.67%	2159.7	7.13%	motif file (matrix)	svg
207	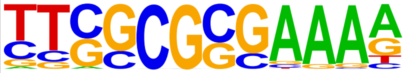	E2F(E2F)/Hela-CellCycle-Expression/Homer	1e-10	-2.504e+01	0.0000	820.0	6.21%	1487.2	4.91%	motif file (matrix)	svg
208	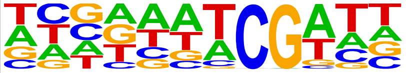	AT2G15740(C2H2)/col-AT2G15740-DAP-Seq(GSE60143)/Homer	1e-10	-2.475e+01	0.0000	3184.0	24.12%	6579.5	21.72%	motif file (matrix)	svg
209	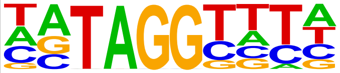	SRS7(SRS)/colamp-SRS7-DAP-Seq(GSE60143)/Homer	1e-10	-2.422e+01	0.0000	10240.0	77.58%	22768.6	75.15%	motif file (matrix)	svg
210	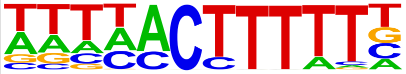	OBP4(C2C2dof)/col-OBP4-DAP-Seq(GSE60143)/Homer	1e-10	-2.413e+01	0.0000	4626.0	35.05%	9807.8	32.37%	motif file (matrix)	svg
211	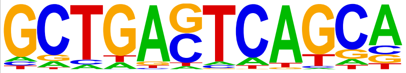	MafK(bZIP)/C2C12-MafK-ChIP-Seq(GSE36030)/Homer	1e-10	-2.384e+01	0.0000	1311.0	9.93%	2523.6	8.33%	motif file (matrix)	svg
212	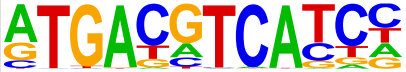	c-Jun-CRE(bZIP)/K562-cJun-ChIP-Seq(GSE31477)/Homer	1e-10	-2.377e+01	0.0000	1411.0	10.69%	2737.0	9.03%	motif file (matrix)	svg
213	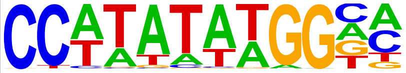	CArG(MADS)/PUER-Srf-ChIP-Seq(Sullivan_et_al.)/Homer	1e-10	-2.357e+01	0.0000	1206.0	9.14%	2304.9	7.61%	motif file (matrix)	svg
214	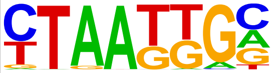	Isl1(Homeobox)/Neuron-Isl1-ChIP-Seq(GSE31456)/Homer	1e-10	-2.345e+01	0.0000	5678.0	43.02%	12199.9	40.26%	motif file (matrix)	svg
215	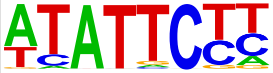	KAN2(G2like)/colamp-KAN2-DAP-Seq(GSE60143)/Homer	1e-10	-2.305e+01	0.0000	3657.0	27.71%	7657.4	25.27%	motif file (matrix)	svg
216	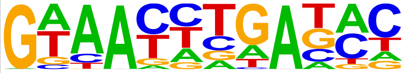	Six2(Homeobox)/NephronProgenitor-Six2-ChIP-Seq(GSE39837)/Homer	1e-9	-2.297e+01	0.0000	3778.0	28.62%	7928.8	26.17%	motif file (matrix)	svg
217	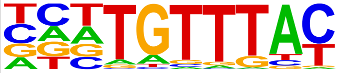	FOXK1(Forkhead)/HEK293-FOXK1-ChIP-Seq(GSE51673)/Homer	1e-9	-2.266e+01	0.0000	3086.0	23.38%	6396.7	21.11%	motif file (matrix)	svg
218	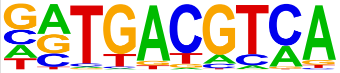	Atf1(bZIP)/K562-ATF1-ChIP-Seq(GSE31477)/Homer	1e-9	-2.247e+01	0.0000	3043.0	23.05%	6305.0	20.81%	motif file (matrix)	svg
219	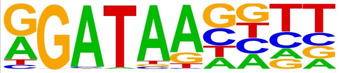	At1g49010(MYBrelated)/col-At1g49010-DAP-Seq(GSE60143)/Homer	1e-9	-2.245e+01	0.0000	4953.0	37.53%	10576.5	34.91%	motif file (matrix)	svg
220	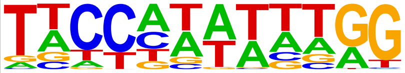	SOC1(MADS)/Seedling-SOC1-ChIP-Seq(GSE45846)/Homer	1e-9	-2.229e+01	0.0000	1158.0	8.77%	2216.6	7.32%	motif file (matrix)	svg
221	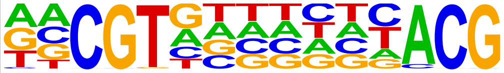	ANAC094(NAC)/col-ANAC094-DAP-Seq(GSE60143)/Homer	1e-9	-2.161e+01	0.0000	3479.0	26.36%	7286.9	24.05%	motif file (matrix)	svg
222	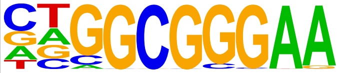	E2F1(E2F)/Hela-E2F1-ChIP-Seq(GSE22478)/Homer	1e-9	-2.153e+01	0.0000	3561.0	26.98%	7470.2	24.65%	motif file (matrix)	svg
223	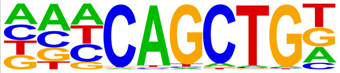	Ap4(bHLH)/AML-Tfap4-ChIP-Seq(GSE45738)/Homer	1e-9	-2.129e+01	0.0000	6675.0	50.57%	14519.1	47.92%	motif file (matrix)	svg
224	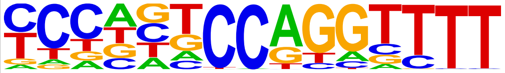	PRDM15(Zf)/ESC-Prdm15-ChIP-Seq(GSE73694)/Homer	1e-9	-2.124e+01	0.0000	5904.0	44.73%	12758.9	42.11%	motif file (matrix)	svg

225	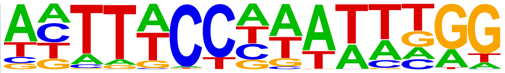	SVP(MADS)/col-SVP-DAP-Seq(GSE60143)/Homer	1e-9	-2.120e+01	0.0000	1436.0	10.88%	2819.5	9.31%	motif file (matrix)	svg
226	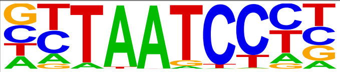	Otx2(Homeobox)/EpiLC-Otx2-ChIP-Seq(GSE56098)/Homer	1e-9	-2.073e+01	0.0000	2528.0	19.15%	5198.0	17.16%	motif file (matrix)	svg
227	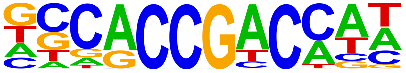	At4g28140(AP2EREBP)/colamp-At4g28140-DAP-Seq(GSE60143)/Homer	1e-8	-2.071e+01	0.0000	2759.0	20.90%	5706.2	18.83%	motif file (matrix)	svg
228	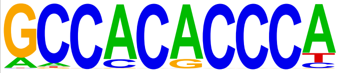	Klf4(Zf)/mES-Klf4-ChIP-Seq(GSE11431)/Homer	1e-8	-2.050e+01	0.0000	3867.0	29.30%	8172.1	26.97%	motif file (matrix)	svg
229	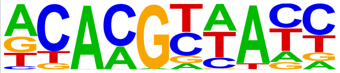	ANAC046(NAC)/colamp-ANAC046-DAP-Seq(GSE60143)/Homer	1e-8	-2.039e+01	0.0000	6336.0	48.00%	13763.0	45.42%	motif file (matrix)	svg
230	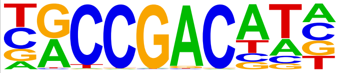	CBF1(AP2EREBP)/colamp-CBF1-DAP-Seq(GSE60143)/Homer	1e-8	-2.010e+01	0.0000	4658.0	35.29%	9957.6	32.86%	motif file (matrix)	svg
231	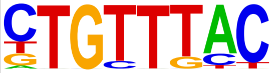	Foxo1(Forkhead)/RAW-Foxo1-ChIP-Seq(Fan_et_al.)/Homer	1e-8	-2.005e+01	0.0000	6755.0	51.18%	14729.4	48.61%	motif file (matrix)	svg
232	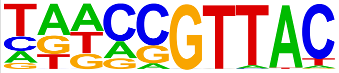	MYB118(MYB)/colamp-MYB118-DAP-Seq(GSE60143)/Homer	1e-8	-1.994e+01	0.0000	1155.0	8.75%	2235.1	7.38%	motif file (matrix)	svg
233	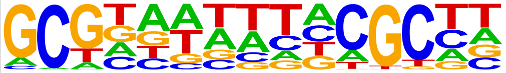	LBD23(LOBAS2)/colamp-LBD23-DAP-Seq(GSE60143)/Homer	1e-8	-1.941e+01	0.0000	6577.0	49.83%	14335.4	47.31%	motif file (matrix)	svg
234	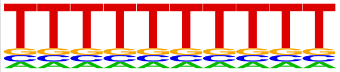	VRN1(ABI3VP1)/col-VRN1-DAP-Seq(GSE60143)/Homer	1e-8	-1.940e+01	0.0000	1316.0	9.97%	2584.9	8.53%	motif file (matrix)	svg
235	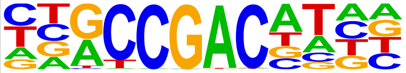	CBF3(AP2EREBP)/colamp-CBF3-DAP-Seq(GSE60143)/Homer	1e-8	-1.902e+01	0.0000	3244.0	24.58%	6810.3	22.48%	motif file (matrix)	svg
236	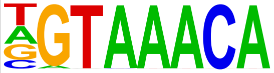	Foxo3(Forkhead)/U2OS-Foxo3-ChIP-Seq(E-MTAB-2701)/Homer	1e-8	-1.900e+01	0.0000	2180.0	16.52%	4461.1	14.72%	motif file (matrix)	svg
237	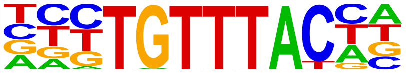	FOXP1(Forkhead)/H9-FOXP1-ChIP-Seq(GSE31006)/Homer	1e-8	-1.900e+01	0.0000	1421.0	10.77%	2814.4	9.29%	motif file (matrix)	svg
238	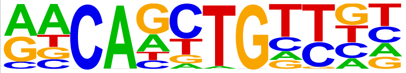	HLH-1(bHLH)/cElegans-Embryo-HLH1-ChIP-Seq(modEncode)/Homer	1e-8	-1.891e+01	0.0000	3520.0	26.67%	7427.5	24.51%	motif file (matrix)	svg
239	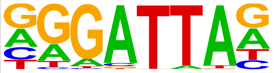	GSC(Homeobox)/FrogEmbryos-GSC-ChIP-Seq(DRA000576)/Homer	1e-8	-1.889e+01	0.0000	3326.0	25.20%	6995.5	23.09%	motif file (matrix)	svg
240	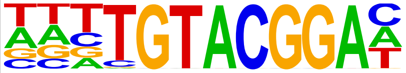	SPL3(SBP)/colamp-SPL3-DAP-Seq(GSE60143)/Homer	1e-8	-1.859e+01	0.0000	263.0	1.99%	418.5	1.38%	motif file (matrix)	svg
241	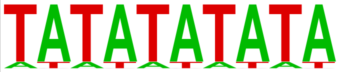	SeqBias: TA-repeat	1e-8	-1.851e+01	0.0000	5616.0	42.55%	12161.6	40.14%	motif file (matrix)	svg
242	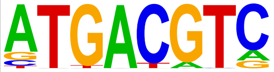	TGA10(bZIP)/colamp-TGA10-DAP-Seq(GSE60143)/Homer	1e-7	-1.814e+01	0.0000	2514.0	19.05%	5209.8	17.19%	motif file (matrix)	svg
243	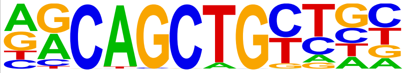	MyoD(bHLH)/Myotube-MyoD-ChIP-Seq(GSE21614)/Homer	1e-7	-1.793e+01	0.0000	5345.0	40.50%	11558.8	38.15%	motif file (matrix)	svg

244	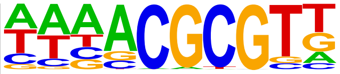	CAMTA1(CAMTA)/col-CAMTA1-DAP-Seq(GSE60143)/Homer	1e-7	-1.764e+01	0.0000	3959.0	29.99%	8435.7	27.84%	motif file (matrix)	svg
245	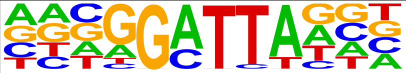	bcd(Homeobox)/Embryo-Bcd-ChIP-Seq(GSE86966)/Homer	1e-7	-1.743e+01	0.0000	3958.0	29.99%	8437.9	27.85%	motif file (matrix)	svg
246	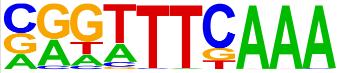	CHR(?)/Hela-CellCycle-Expression/Homer	1e-7	-1.740e+01	0.0000	1965.0	14.89%	4016.1	13.25%	motif file (matrix)	svg
247	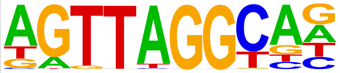	MYB116(MYB)/colamp-MYB116-DAP-Seq(GSE60143)/Homer	1e-7	-1.735e+01	0.0000	2399.0	18.18%	4969.9	16.40%	motif file (matrix)	svg
248	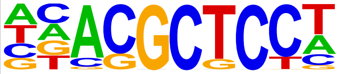	MYB88(MYB)/col-MYB88-DAP-Seq(GSE60143)/Homer	1e-7	-1.732e+01	0.0000	11691.0	88.57%	26364.3	87.01%	motif file (matrix)	svg
249	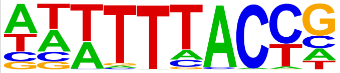	AT5G47660(Trihelix)/colamp-AT5G47660-DAP-Seq(GSE60143)/Homer	1e-7	-1.724e+01	0.0000	3810.0	28.87%	8109.7	26.77%	motif file (matrix)	svg
250	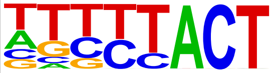	At3g60580(C2H2)/col-At3g60580-DAP-Seq(GSE60143)/Homer	1e-7	-1.721e+01	0.0000	11885.0	90.04%	26837.8	88.58%	motif file (matrix)	svg
251	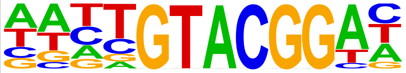	SPL13(SBP)/col-SPL13-DAP-Seq(GSE60143)/Homer	1e-7	-1.710e+01	0.0000	485.0	3.67%	866.6	2.86%	motif file (matrix)	svg
252	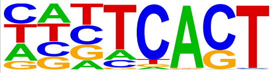	STZ(C2H2)/colamp-STZ-DAP-Seq(GSE60143)/Homer	1e-7	-1.703e+01	0.0000	12323.0	93.36%	27915.7	92.13%	motif file (matrix)	svg
253	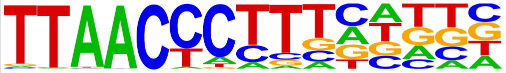	ZNF652/HepG2-ZNF652.Flag-ChIP-Seq(Encode)/Homer	1e-7	-1.690e+01	0.0000	1013.0	7.67%	1968.6	6.50%	motif file (matrix)	svg
254	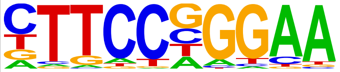	Stat3(Stat)/mES-Stat3-ChIP-Seq(GSE11431)/Homer	1e-7	-1.672e+01	0.0000	3278.0	24.84%	6930.2	22.87%	motif file (matrix)	svg
255	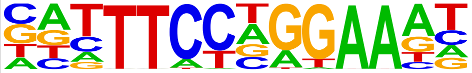	STAT1(Stat)/HelaS3-STAT1-ChIP-Seq(GSE12782)/Homer	1e-7	-1.647e+01	0.0000	1206.0	9.14%	2385.2	7.87%	motif file (matrix)	svg
256	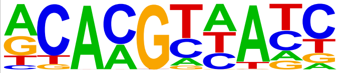	ANAC038(NAC)/col-ANAC038-DAP-Seq(GSE60143)/Homer	1e-7	-1.641e+01	0.0000	6965.0	52.77%	15295.5	50.48%	motif file (matrix)	svg
257	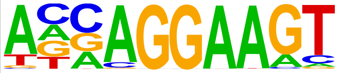	ELF3(ETS)/PDAC-ELF3-ChIP-Seq(GSE64557)/Homer	1e-7	-1.612e+01	0.0000	3946.0	29.90%	8438.6	27.85%	motif file (matrix)	svg
258	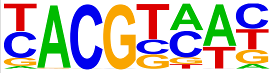	ATAF1(NAC)/col-ATAF1-DAP-Seq(GSE60143)/Homer	1e-6	-1.604e+01	0.0000	10124.0	76.70%	22650.9	74.76%	motif file (matrix)	svg
259	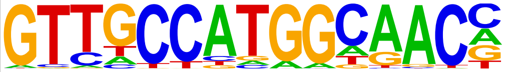	Rfx2(HTH)/LoVo-RFX2-ChIP-Seq(GSE49402)/Homer	1e-6	-1.590e+01	0.0000	660.0	5.00%	1236.2	4.08%	motif file (matrix)	svg
260	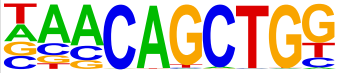	Tcf21(bHLH)/ArterySmoothMuscle-Tcf21-ChIP-Seq(GSE61369)/Homer	1e-6	-1.582e+01	0.0000	5701.0	43.19%	12417.6	40.98%	motif file (matrix)	svg
261	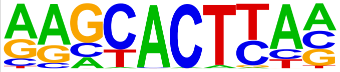	Nkx3.1(Homeobox)/LNCaP-Nkx3.1-ChIP-Seq(GSE28264)/Homer	1e-6	-1.563e+01	0.0000	7981.0	60.47%	17658.0	58.28%	motif file (matrix)	svg
262	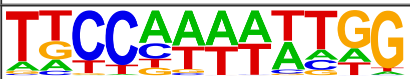	AGL63(MADS)/col-AGL63-DAP-Seq(GSE60143)/Homer	1e-6	-1.549e+01	0.0000	2302.0	17.44%	4788.9	15.81%	motif file (matrix)	svg

263	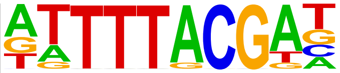	ARF16(ARF)/col-ARF16-DAP-Seq(GSE60143)/Homer	1e-6	-1.518e+01	0.0000	625.0	4.74%	1170.0	3.86%	motif file (matrix)	svg
264	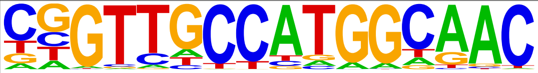	RFX(HTH)/K562-RFX3-ChIP-Seq(SRA012198)/Homer	1e-6	-1.481e+01	0.0000	618.0	4.68%	1159.1	3.83%	motif file (matrix)	svg
265	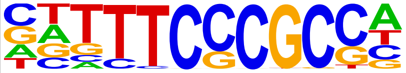	E2F7(E2F)/Hela-E2F7-ChIP-Seq(GSE32673)/Homer	1e-6	-1.478e+01	0.0000	1814.0	13.74%	3728.5	12.31%	motif file (matrix)	svg
266	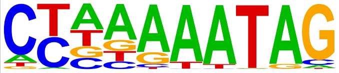	Mef2a(MADS)/HL1-Mef2a.biotin-ChIP-Seq(GSE21529)/Homer	1e-6	-1.474e+01	0.0000	1214.0	9.20%	2425.2	8.00%	motif file (matrix)	svg
267	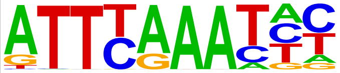	AT2G20110(CPP)/colamp-AT2G20110-DAP-Seq(GSE60143)/Homer	1e-6	-1.455e+01	0.0000	3852.0	29.18%	8262.2	27.27%	motif file (matrix)	svg
268	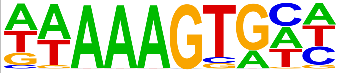	AT1G69570(C2C2dof)/col-AT1G69570-DAP-Seq(GSE60143)/Homer	1e-6	-1.440e+01	0.0000	4247.0	32.18%	9155.7	30.22%	motif file (matrix)	svg
269	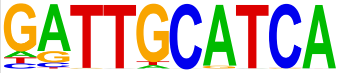	AARE(HLH)/mES-cMyc-ChIP-Seq/Homer	1e-6	-1.429e+01	0.0000	326.0	2.47%	566.6	1.87%	motif file (matrix)	svg
270	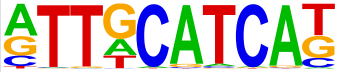	Chop(bZIP)/MEF-Chop-ChIP-Seq(GSE35681)/Homer	1e-6	-1.397e+01	0.0000	710.0	5.38%	1360.8	4.49%	motif file (matrix)	svg
271	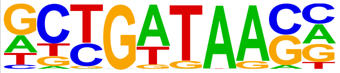	Unknown5/Drosophila-Promoters/Homer	1e-5	-1.381e+01	0.0000	2371.0	17.96%	4972.7	16.41%	motif file (matrix)	svg
272	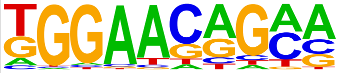	ZNF189(Zf)/HEK293-ZNF189.GFP-ChIP-Seq(GSE58341)/Homer	1e-5	-1.363e+01	0.0000	4415.0	33.45%	9553.3	31.53%	motif file (matrix)	svg
273	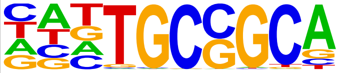	Zfp57(Zf)/H1-ZFP57.HA-ChIP-Seq(GSE115387)/Homer	1e-5	-1.351e+01	0.0000	4469.0	33.86%	9678.7	31.94%	motif file (matrix)	svg
274	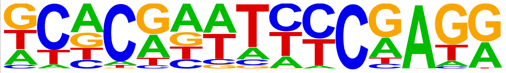	AIL7(AP2EREBP)/colamp-AIL7-DAP-Seq(GSE60143)/Homer	1e-5	-1.348e+01	0.0000	2507.0	18.99%	5280.4	17.43%	motif file (matrix)	svg
275	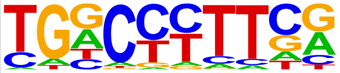	NLP7(RWPRK)/col-NLP7-DAP-Seq(GSE60143)/Homer	1e-5	-1.345e+01	0.0000	8792.0	66.61%	19594.5	64.67%	motif file (matrix)	svg
276	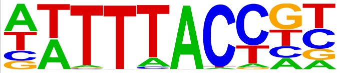	GTL1(Trihelix)/colamp-GTL1-DAP-Seq(GSE60143)/Homer	1e-5	-1.324e+01	0.0000	2903.0	21.99%	6166.4	20.35%	motif file (matrix)	svg
277	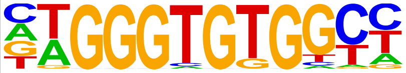	EKLF(Zf)/Erythrocyte-Klf1-ChIP-Seq(GSE20478)/Homer	1e-5	-1.324e+01	0.0000	1581.0	11.98%	3244.0	10.71%	motif file (matrix)	svg
278	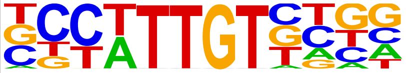	Sox21(HMG)/ESC-SOX21-ChIP-Seq(GSE110505)/Homer	1e-5	-1.311e+01	0.0000	5842.0	44.26%	12808.7	42.27%	motif file (matrix)	svg
279	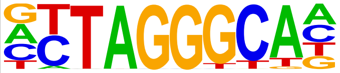	TBP3(MYBrelated)/col-TBP3-DAP-Seq(GSE60143)/Homer	1e-5	-1.303e+01	0.0000	3278.0	24.84%	7009.9	23.14%	motif file (matrix)	svg
280	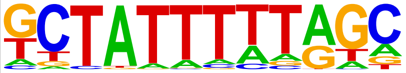	Mef2d(MADS)/Retina-Mef2d-ChIP-Seq(GSE61391)/Homer	1e-5	-1.298e+01	0.0000	606.0	4.59%	1152.4	3.80%	motif file (matrix)	svg
281	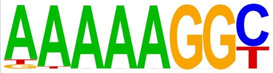	dof42(C2C2dof)/col-dof42-DAP-Seq(GSE60143)/Homer	1e-5	-1.289e+01	0.0000	2583.0	19.57%	5461.6	18.03%	motif file (matrix)	svg

282	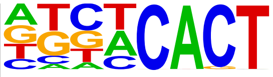	AZF1(C2H2)/colamp-AZF1-DAP-Seq(GSE60143)/Homer	1e-5	-1.287e+01	0.0000	12414.0	94.05%	28197.1	93.06%	motif file (matrix)	svg
283	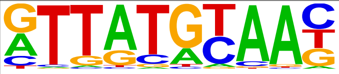	HLF(bZIP)/HSC-HLF.Flag-ChIP-Seq(GSE69817)/Homer	1e-5	-1.287e+01	0.0000	1949.0	14.77%	4058.5	13.39%	motif file (matrix)	svg
284	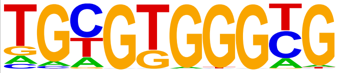	Egr1(Zf)/K562-Egr1-ChIP-Seq(GSE32465)/Homer	1e-5	-1.281e+01	0.0000	7563.0	57.30%	16765.7	55.33%	motif file (matrix)	svg
285	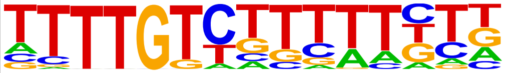	IDD5(C2H2)/colamp-IDD5-DAP-Seq(GSE60143)/Homer	1e-5	-1.278e+01	0.0000	1936.0	14.67%	4031.2	13.30%	motif file (matrix)	svg
286	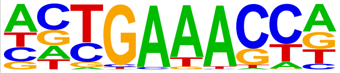	IRF4(IRF)/GM12878-IRF4-ChIP-Seq(GSE32465)/Homer	1e-5	-1.256e+01	0.0000	1735.0	13.14%	3593.7	11.86%	motif file (matrix)	svg
287	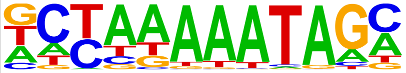	Mef2c(MADS)/GM12878-Mef2c-ChIP-Seq(GSE32465)/Homer	1e-5	-1.249e+01	0.0000	1188.0	9.00%	2401.6	7.93%	motif file (matrix)	svg
288	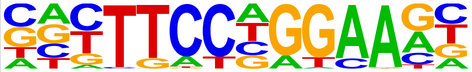	Stat3+il21(Stat)/CD4-Stat3-ChIP-Seq(GSE19198)/Homer	1e-5	-1.240e+01	0.0000	3775.0	28.60%	8140.2	26.87%	motif file (matrix)	svg
289	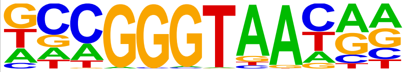	REB1/SacCer-Promoters/Homer	1e-5	-1.238e+01	0.0000	1028.0	7.79%	2057.8	6.79%	motif file (matrix)	svg
290	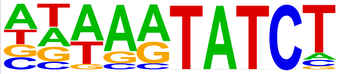	LHY(Myb)/Seedling-LHY-ChIP-Seq(GSE52175)/Homer	1e-5	-1.197e+01	0.0000	2087.0	15.81%	4380.1	14.46%	motif file (matrix)	svg
291	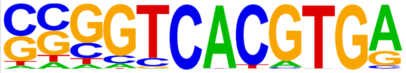	E-box(bHLH)/Promoter/Homer	1e-5	-1.190e+01	0.0000	861.0	6.52%	1705.3	5.63%	motif file (matrix)	svg
292	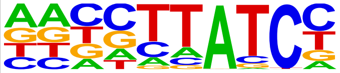	At5g08520(MYBrelated)/colamp-At5g08520-DAP-Seq(GSE60143)/Homer	1e-5	-1.174e+01	0.0000	3173.0	24.04%	6804.9	22.46%	motif file (matrix)	svg
293	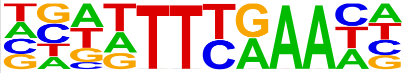	TCX2(CPP)/colamp-TCX2-DAP-Seq(GSE60143)/Homer	1e-5	-1.153e+01	0.0000	4099.0	31.06%	8893.0	29.35%	motif file (matrix)	svg
294	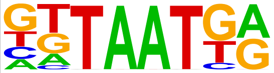	Nkx6.1(Homeobox)/Islet-Nkx6.1-ChIP-Seq(GSE40975)/Homer	1e-4	-1.148e+01	0.0000	5819.0	44.09%	12801.5	42.25%	motif file (matrix)	svg
295	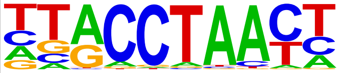	MYB57(MYB)/col-MYB57-DAP-Seq(GSE60143)/Homer	1e-4	-1.148e+01	0.0000	1339.0	10.14%	2745.8	9.06%	motif file (matrix)	svg
296	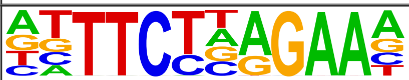	STAT5(Stat)/mCD4+-Stat5-ChIP-Seq(GSE12346)/Homer	1e-4	-1.143e+01	0.0000	1389.0	10.52%	2855.7	9.42%	motif file (matrix)	svg
297	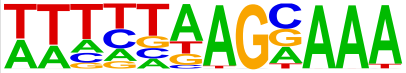	AT5G60130(ABI3VP1)/col-AT5G60130-DAP-Seq(GSE60143)/Homer	1e-4	-1.140e+01	0.0000	4663.0	35.33%	10173.6	33.58%	motif file (matrix)	svg
298	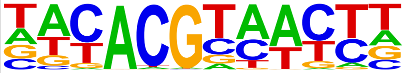	ANAC047(NAC)/colamp-ANAC047-DAP-Seq(GSE60143)/Homer	1e-4	-1.138e+01	0.0000	3095.0	23.45%	6638.6	21.91%	motif file (matrix)	svg
299	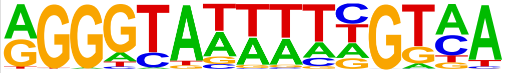	Unknown1/Arabidopsis-Promoters/Homer	1e-4	-1.136e+01	0.0000	316.0	2.39%	567.4	1.87%	motif file (matrix)	svg
300	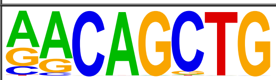	MyoG(bHLH)/C2C12-MyoG-ChIP-Seq(GSE36024)/Homer	1e-4	-1.108e+01	0.0001	6304.0	47.76%	13922.0	45.95%	motif file (matrix)	svg

301	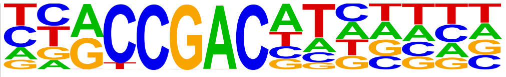	CBF2(AP2EREBP)/colamp-CBF2-DAP-Seq(GSE60143)/Homer	1e-4	-1.092e+01	0.0001	3172.0	24.03%	6822.2	22.52%	motif file (matrix)	svg
302	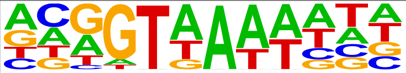	GT2(Trihelix)/colamp-GT2-DAP-Seq(GSE60143)/Homer	1e-4	-1.088e+01	0.0001	2569.0	19.46%	5475.0	18.07%	motif file (matrix)	svg
303	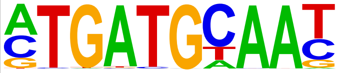	Atf4(bZIP)/MEF-Atf4-ChIP-Seq(GSE35681)/Homer	1e-4	-1.061e+01	0.0001	905.0	6.86%	1817.2	6.00%	motif file (matrix)	svg
304	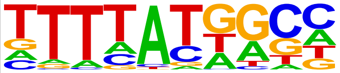	Hoxa11(Homeobox)/ChickenMSG-Hoxa11.Flag-ChIP-Seq(GSE86088)/Homer	1e-4	-1.057e+01	0.0001	6085.0	46.10%	13436.6	44.35%	motif file (matrix)	svg
305	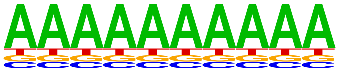	SeqBias: polyA-repeat	1e-4	-1.046e+01	0.0001	13199.0	100.00%	30275.7	99.92%	motif file (matrix)	svg
306	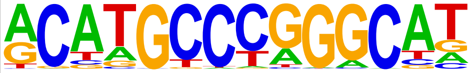	p53(p53)/mES-cMyc-ChIP-Seq(GSE11431)/Homer	1e-4	-1.038e+01	0.0001	143.0	1.08%	230.6	0.76%	motif file (matrix)	svg
307	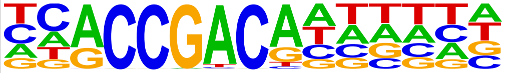	AT3G16280(AP2EREBP)/colamp-AT3G16280-DAP-Seq(GSE60143)/Homer	1e-4	-1.034e+01	0.0001	2263.0	17.15%	4805.8	15.86%	motif file (matrix)	svg
308	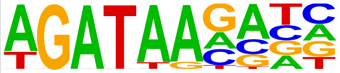	TRPS1(Zf)/MCF7-TRPS1-ChIP-Seq(GSE107013)/Homer	1e-4	-1.024e+01	0.0001	4939.0	37.42%	10833.7	35.76%	motif file (matrix)	svg
309	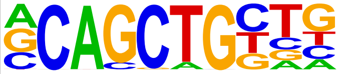	Tcf12(bHLH)/GM12878-Tcf12-ChIP-Seq(GSE32465)/Homer	1e-4	-1.022e+01	0.0001	6047.0	45.81%	13360.7	44.10%	motif file (matrix)	svg
310	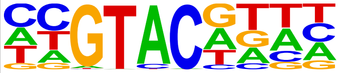	SPL1(SBP)/colamp-SPL1-DAP-Seq(GSE60143)/Homer	1e-4	-1.014e+01	0.0001	4968.0	37.64%	10902.7	35.98%	motif file (matrix)	svg
311	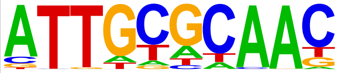	CEBP(bZIP)/ThioMac-CEBPb-ChIP-Seq(GSE21512)/Homer	1e-4	-1.011e+01	0.0001	1780.0	13.49%	3739.5	12.34%	motif file (matrix)	svg
312	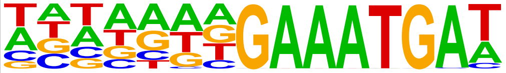	AT2G31460(REMB3)/col-AT2G31460-DAP-Seq(GSE60143)/Homer	1e-4	-9.881e+00	0.0002	1050.0	7.96%	2142.3	7.07%	motif file (matrix)	svg
313	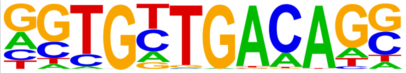	Tbx20(T-box)/Heart-Tbx20-ChIP-Seq(GSE29636)/Homer	1e-4	-9.744e+00	0.0002	1150.0	8.71%	2362.6	7.80%	motif file (matrix)	svg
314	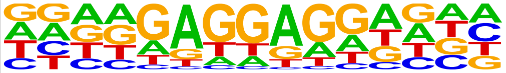	TF3A(C2H2)/col-TF3A-DAP-Seq(GSE60143)/Homer	1e-4	-9.589e+00	0.0002	8477.0	64.22%	18973.8	62.62%	motif file (matrix)	svg
315	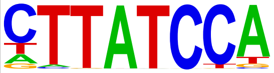	AT5G61620(MYBrelated)/colamp-AT5G61620-DAP-Seq(GSE60143)/Homer	1e-4	-9.577e+00	0.0002	4012.0	30.40%	8751.6	28.88%	motif file (matrix)	svg
316	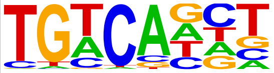	Tgif2(Homeobox)/mES-Tgif2-ChIP-Seq(GSE55404)/Homer	1e-4	-9.438e+00	0.0003	9359.0	70.91%	21026.2	69.39%	motif file (matrix)	svg
317	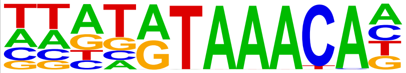	Foxf1(Forkhead)/Lung-Foxf1-ChIP-Seq(GSE77951)/Homer	1e-3	-9.208e+00	0.0003	2477.0	18.77%	5309.9	17.52%	motif file (matrix)	svg
318	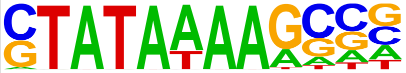	TATA-box/Drosophila-Promoters/Homer	1e-3	-8.752e+00	0.0005	211.0	1.60%	375.0	1.24%	motif file (matrix)	svg
319	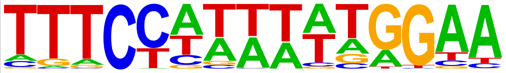	AGL25(MADS)/colamp-AGL25-DAP-Seq(GSE60143)/Homer	1e-3	-8.745e+00	0.0005	170.0	1.29%	292.8	0.97%	motif file (matrix)	svg

320	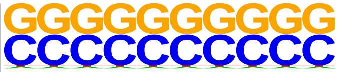	SeqBias: CG bias	1e-3	-8.715e+00	0.0005	13199.0	100.00%	30279.4	99.93%	motif file (matrix)	svg
321	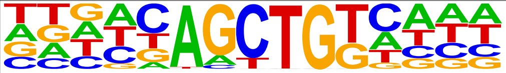	bZIP52(bZIP)/colamp-bZIP52-DAP-Seq(GSE60143)/Homer	1e-3	-8.689e+00	0.0005	5336.0	40.43%	11786.6	38.90%	motif file (matrix)	svg
322	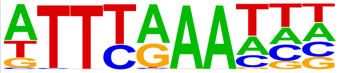	SOL1(CPP)/colamp-SOL1-DAP-Seq(GSE60143)/Homer	1e-3	-8.648e+00	0.0005	3863.0	29.27%	8442.5	27.86%	motif file (matrix)	svg
323	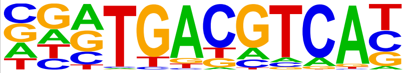	Atf7(bZIP)/3T3L1-Atf7-ChIP-Seq(GSE56872)/Homer	1e-3	-8.554e+00	0.0006	2338.0	17.71%	5016.0	16.55%	motif file (matrix)	svg
324	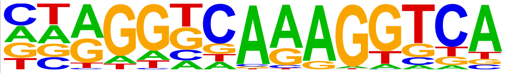	PPARa(NR),DR1/Liver-Ppara-ChIP-Seq(GSE47954)/Homer	1e-3	-8.551e+00	0.0006	5600.0	42.43%	12393.4	40.90%	motif file (matrix)	svg
325	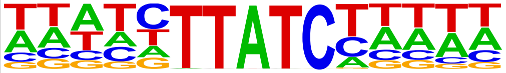	At5g58900(MYBrelated)/colamp-At5g58900-DAP-Seq(GSE60143)/Homer	1e-3	-8.516e+00	0.0006	3161.0	23.95%	6862.2	22.65%	motif file (matrix)	svg
326	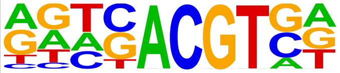	HY5(bZIP)/colamp-HY5-DAP-Seq(GSE60143)/Homer	1e-3	-8.406e+00	0.0007	4687.0	35.51%	10318.0	34.05%	motif file (matrix)	svg
327	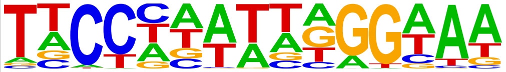	AGL16(MADS)/col-AGL16-DAP-Seq(GSE60143)/Homer	1e-3	-8.384e+00	0.0007	299.0	2.27%	557.2	1.84%	motif file (matrix)	svg
328	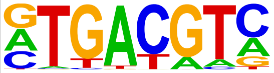	TGA9(bZIP)/colamp-TGA9-DAP-Seq(GSE60143)/Homer	1e-3	-8.327e+00	0.0007	4278.0	32.41%	9392.6	31.00%	motif file (matrix)	svg
329	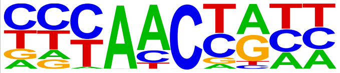	ATY13(MYB)/col-ATY13-DAP-Seq(GSE60143)/Homer	1e-3	-8.114e+00	0.0009	10238.0	77.57%	23118.5	76.30%	motif file (matrix)	svg
330	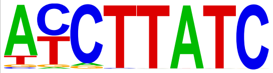	At5g05790(MYBrelated)/col-At5g05790-DAP-Seq(GSE60143)/Homer	1e-3	-8.113e+00	0.0009	2525.0	19.13%	5445.0	17.97%	motif file (matrix)	svg
331	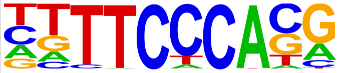	Rbpj1(?)/Panc1-Rbpj1-ChIP-Seq(GSE47459)/Homer	1e-3	-8.086e+00	0.0009	6911.0	52.36%	15412.7	50.87%	motif file (matrix)	svg
332	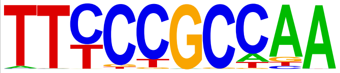	DEL1(E2FDP)/colamp-DEL1-DAP-Seq(GSE60143)/Homer	1e-3	-8.024e+00	0.0010	35.0	0.27%	42.5	0.14%	motif file (matrix)	svg
333	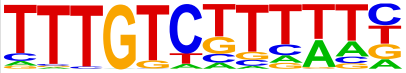	IDD4(C2H2)/col-IDD4-DAP-Seq(GSE60143)/Homer	1e-3	-7.807e+00	0.0012	2348.0	17.79%	5057.3	16.69%	motif file (matrix)	svg
334	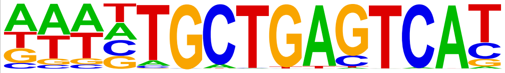	Bach1(bZIP)/K562-Bach1-ChIP-Seq(GSE31477)/Homer	1e-3	-7.792e+00	0.0012	305.0	2.31%	575.4	1.90%	motif file (matrix)	svg
335	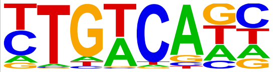	Tgif1(Homeobox)/mES-Tgif1-ChIP-Seq(GSE55404)/Homer	1e-3	-7.792e+00	0.0012	8588.0	65.07%	19290.9	63.67%	motif file (matrix)	svg
336	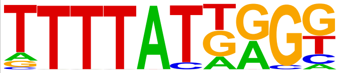	HOXB13(Homeobox)/ProstateTumor-HOXB13-ChIP-Seq(GSE56288)/Homer	1e-3	-7.738e+00	0.0013	2998.0	22.71%	6518.4	21.51%	motif file (matrix)	svg
337	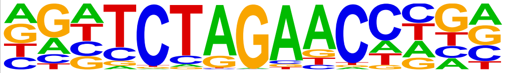	ZBTB12(Zf)/HEK293-ZBTB12.GFP-ChIP-Seq(GSE58341)/Homer	1e-3	-7.494e+00	0.0017	2329.0	17.65%	5023.7	16.58%	motif file (matrix)	svg
338	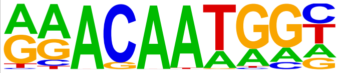	Sox15(HMG)/CPA-Sox15-ChIP-Seq(GSE62909)/Homer	1e-3	-7.492e+00	0.0017	3431.0	25.99%	7502.6	24.76%	motif file (matrix)	svg

339	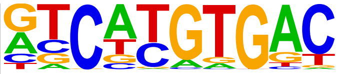	MITF(bHLH)/MastCells-MITF-ChIP-Seq(GSE48085)/Homer	1e-3	-7.469e+00	0.0017	4364.0	33.06%	9616.8	31.74%	motif file (matrix)	svg
340	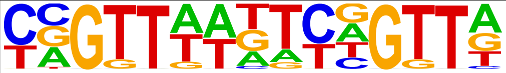	MYB44(MYB)/colamp-MYB44-DAP-Seq(GSE60143)/Homer	1e-3	-7.437e+00	0.0017	230.0	1.74%	423.6	1.40%	motif file (matrix)	svg
341	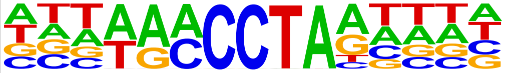	AT1G72740(MYBrelated)/colamp-AT1G72740-DAP-Seq(GSE60143)/Homer	1e-3	-7.391e+00	0.0018	3271.0	24.78%	7144.7	23.58%	motif file (matrix)	svg
342	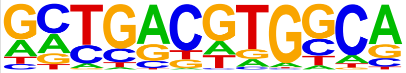	O2(bZIP)/Corn-O2-ChIP-Seq(GSE63991)/Homer	1e-3	-7.275e+00	0.0020	983.0	7.45%	2041.5	6.74%	motif file (matrix)	svg
343	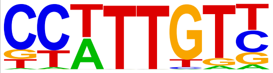	Sox3(HMG)/NPC-Sox3-ChIP-Seq(GSE33059)/Homer	1e-3	-7.260e+00	0.0021	5420.0	41.06%	12028.6	39.70%	motif file (matrix)	svg
344	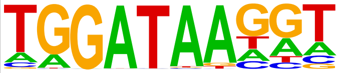	AT5G56840(MYBrelated)/colamp-AT5G56840-DAP-Seq(GSE60143)/Homer	1e-3	-7.133e+00	0.0023	3262.0	24.71%	7132.3	23.54%	motif file (matrix)	svg
345	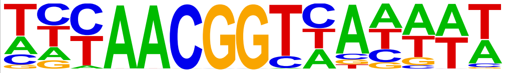	MYB3R5(MYB)/col-MYB3R5-DAP-Seq(GSE60143)/Homer	1e-3	-7.115e+00	0.0024	286.0	2.17%	542.1	1.79%	motif file (matrix)	svg
346	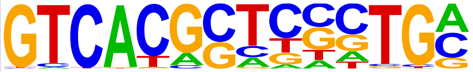	PAX5(Paired,Homeobox),condensed/GM12878-PAX5-ChIP-Seq(GSE32465)/Homer	1e-3	-7.011e+00	0.0026	801.0	6.07%	1648.0	5.44%	motif file (matrix)	svg
347	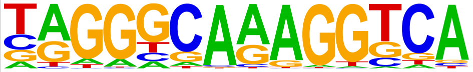	RXR(NR),DR1/3T3L1-RXR-ChIP-Seq(GSE13511)/Homer	1e-3	-6.972e+00	0.0027	6781.0	51.38%	15156.0	50.02%	motif file (matrix)	svg
348	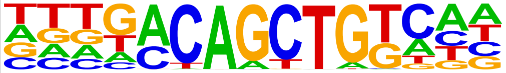	VIP1(bZIP)/col-VIP1-DAP-Seq(GSE60143)/Homer	1e-2	-6.854e+00	0.0031	957.0	7.25%	1992.1	6.57%	motif file (matrix)	svg
349	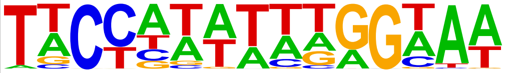	AGL13(MADS)/col-AGL13-DAP-Seq(GSE60143)/Homer	1e-2	-6.834e+00	0.0031	117.0	0.89%	199.8	0.66%	motif file (matrix)	svg
350	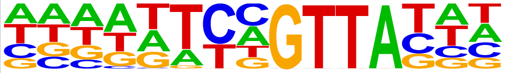	MYB113(MYB)/col-MYB113-DAP-Seq(GSE60143)/Homer	1e-2	-6.824e+00	0.0031	1208.0	9.15%	2545.7	8.40%	motif file (matrix)	svg
351	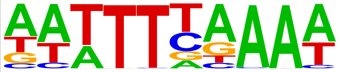	Unknown6/Drosophila-Promoters/Homer	1e-2	-6.808e+00	0.0032	2140.0	16.21%	4619.6	15.25%	motif file (matrix)	svg
352	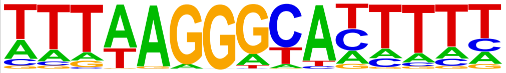	AT2G33550(Trihelix)/colamp-AT2G33550-DAP-Seq(GSE60143)/Homer	1e-2	-6.758e+00	0.0033	5218.0	39.53%	11586.9	38.24%	motif file (matrix)	svg
353	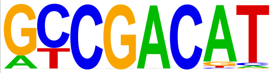	DDF1(AP2EREBP)/col-DDF1-DAP-Seq(GSE60143)/Homer	1e-2	-6.638e+00	0.0037	2539.0	19.24%	5519.4	18.22%	motif file (matrix)	svg
354	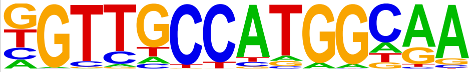	Rfx1(HTH)/NPC-H3K4me1-ChIP-Seq(GSE16256)/Homer	1e-2	-6.504e+00	0.0043	1165.0	8.83%	2457.0	8.11%	motif file (matrix)	svg
355	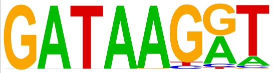	At3g11280(MYBrelated)/col-At3g11280-DAP-Seq(GSE60143)/Homer	1e-2	-6.454e+00	0.0045	2427.0	18.39%	5273.4	17.40%	motif file (matrix)	svg
356	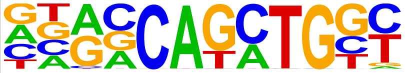	Atoh1(bHLH)/Cerebellum-Atoh1-ChIP-Seq(GSE22111)/Homer	1e-2	-6.448e+00	0.0045	5926.0	44.90%	13216.3	43.62%	motif file (matrix)	svg
357	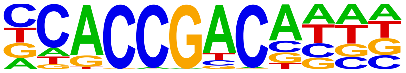	At1g22810(AP2EREBP)/colamp-At1g22810-DAP-Seq(GSE60143)/Homer	1e-2	-6.392e+00	0.0047	3089.0	23.40%	6766.7	22.33%	motif file (matrix)	svg

358	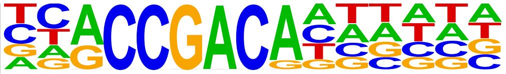	At2g44940(AP2EREBP)/colamp-At2g44940-DAP-Seq(GSE60143)/Homer	1e-2	-6.358e+00	0.0049	1277.0	9.67%	2708.7	8.94%	motif file (matrix)	svg
359	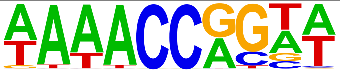	AT1G76870(Trihelix)/col-AT1G76870-DAP-Seq(GSE60143)/Homer	1e-2	-6.324e+00	0.0050	1315.0	9.96%	2793.4	9.22%	motif file (matrix)	svg
360	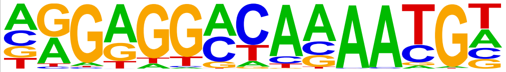	ZNF675(Zf)/HEK293-ZNF675.GFP-ChIP-Seq(GSE58341)/Homer	1e-2	-6.269e+00	0.0053	604.0	4.58%	1232.8	4.07%	motif file (matrix)	svg
361	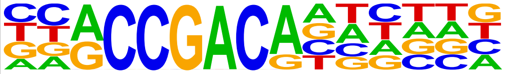	AT3G60490(AP2EREBP)/colamp-AT3G60490-DAP-Seq(GSE60143)/Homer	1e-2	-6.261e+00	0.0053	1881.0	14.25%	4055.1	13.38%	motif file (matrix)	svg
362	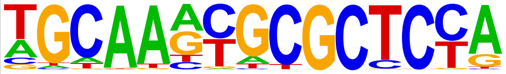	EFL-1(E2F)/cElegans-L1-EFL1-ChIP-Seq(modEncode)/Homer	1e-2	-6.257e+00	0.0053	151.0	1.14%	271.5	0.90%	motif file (matrix)	svg
363	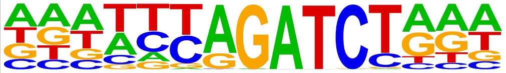	GATA1(C2C2gata)/colamp-GATA1-DAP-Seq(GSE60143)/Homer	1e-2	-6.253e+00	0.0053	1129.0	8.55%	2383.1	7.87%	motif file (matrix)	svg
364	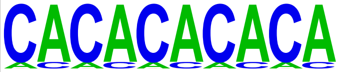	SeqBias: CA-repeat	1e-2	-6.169e+00	0.0058	12658.0	95.90%	28900.3	95.38%	motif file (matrix)	svg
365	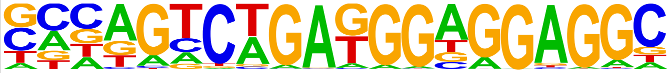	ZSCAN22(Zf)/HEK293-ZSCAN22.GFP-ChIP-Seq(GSE58341)/Homer	1e-2	-6.110e+00	0.0061	831.0	6.30%	1730.9	5.71%	motif file (matrix)	svg
366	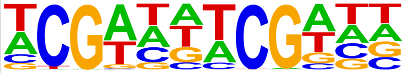	AT5G22990(C2H2)/col-AT5G22990-DAP-Seq(GSE60143)/Homer	1e-2	-6.039e+00	0.0066	640.0	4.85%	1314.1	4.34%	motif file (matrix)	svg
367	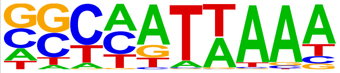	Unknown(Homeobox)/Limb-p300-ChIP-Seq/Homer	1e-2	-6.028e+00	0.0066	1815.0	13.75%	3914.5	12.92%	motif file (matrix)	svg
368	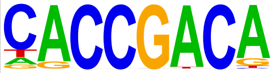	ERF38(AP2EREBP)/col-ERF38-DAP-Seq(GSE60143)/Homer	1e-2	-6.003e+00	0.0068	3283.0	24.87%	7218.0	23.82%	motif file (matrix)	svg
369	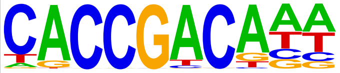	At4g31060(AP2EREBP)/colamp-At4g31060-DAP-Seq(GSE60143)/Homer	1e-2	-5.936e+00	0.0072	2510.0	19.02%	5476.1	18.07%	motif file (matrix)	svg
370	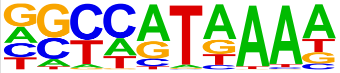	Hoxd11(Homeobox)/ChickenMSG-Hoxd11.Flag-ChIP-Seq(GSE86088)/Homer	1e-2	-5.847e+00	0.0079	6615.0	50.12%	14820.4	48.91%	motif file (matrix)	svg
371	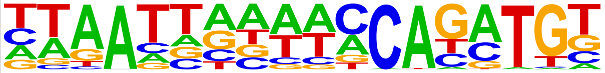	Pitx1:Ebox(Homeobox,bHLH)/Hindlimb-Pitx1-ChIP-Seq(GSE41591)/Homer	1e-2	-5.817e+00	0.0081	482.0	3.65%	975.0	3.22%	motif file (matrix)	svg
372	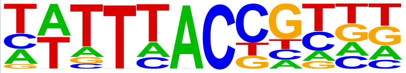	EMB1789(C3H)/col-EMB1789-DAP-Seq(GSE60143)/Homer	1e-2	-5.696e+00	0.0091	1191.0	9.02%	2533.1	8.36%	motif file (matrix)	svg
373	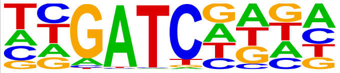	GATA20(C2C2gata)/colamp-GATA20-DAP-Seq(GSE60143)/Homer	1e-2	-5.688e+00	0.0091	6889.0	52.19%	15456.9	51.01%	motif file (matrix)	svg
374	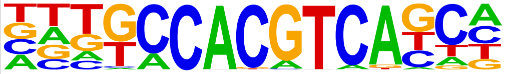	bZIP44(bZIP)/colamp-bZIP44-DAP-Seq(GSE60143)/Homer	1e-2	-5.676e+00	0.0092	139.0	1.05%	251.4	0.83%	motif file (matrix)	svg
375	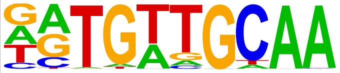	CEBP:AP1(bZIP)/ThioMac-CEBPb-ChIP-Seq(GSE21512)/Homer	1e-2	-5.627e+00	0.0097	2172.0	16.46%	4726.7	15.60%	motif file (matrix)	svg
376	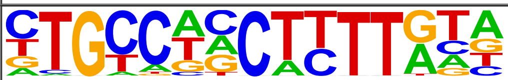	ZNF7(Zf)/HepG2-ZNF7.Flag-ChIP-Seq(Encode)/Homer	1e-2	-5.574e+00	0.0102	1844.0	13.97%	3992.2	13.18%	motif file (matrix)	svg

377	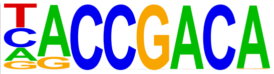	bHLH10(bHLH)/colamp-bHLH10-DAP-Seq(GSE60143)/Homer	1e-2	-5.538e+00	0.0105	1827.0	13.84%	3955.2	13.05%	motif file (matrix)	svg
378	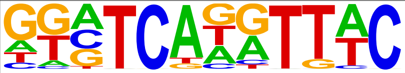	Six1(Homeobox)/Myoblast-Six1-ChIP-Chip(GSE20150)/Homer	1e-2	-5.498e+00	0.0109	958.0	7.26%	2022.1	6.67%	motif file (matrix)	svg
379	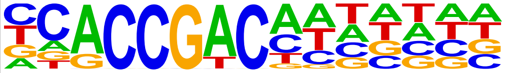	DEAR3(AP2EREBP)/colamp-DEAR3-DAP-Seq(GSE60143)/Homer	1e-2	-5.455e+00	0.0113	2178.0	16.50%	4745.9	15.66%	motif file (matrix)	svg
380	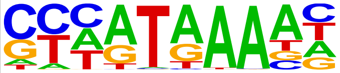	Hoxa13(Homeobox)/ChickenMSG-Hoxa13.Flag-ChIP-Seq(GSE86088)/Homer	1e-2	-5.422e+00	0.0117	6330.0	47.96%	14186.0	46.82%	motif file (matrix)	svg
381	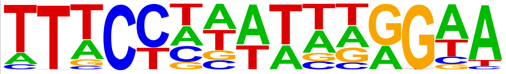	AGL15(MADS)/col-AGL15-DAP-Seq(GSE60143)/Homer	1e-2	-5.395e+00	0.0120	417.0	3.16%	841.9	2.78%	motif file (matrix)	svg
382	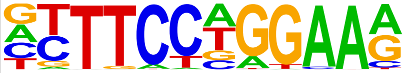	STAT4(Stat)/CD4-Stat4-ChIP-Seq(GSE22104)/Homer	1e-2	-5.378e+00	0.0122	3931.0	29.78%	8711.8	28.75%	motif file (matrix)	svg
383	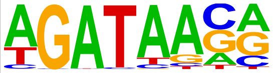	GATA3(Zf)/iTreg-Gata3-ChIP-Seq(GSE20898)/Homer	1e-2	-5.321e+00	0.0128	3893.0	29.49%	8627.0	28.47%	motif file (matrix)	svg
384	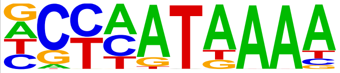	Hoxd13(Homeobox)/ChickenMSG-Hoxd13.Flag-ChIP-Seq(GSE86088)/Homer	1e-2	-5.309e+00	0.0130	4082.0	30.93%	9057.1	29.89%	motif file (matrix)	svg
385	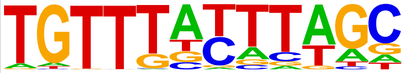	FoxD3(forkhead)/ZebrafishEmbryo-Foxd3.biotin-ChIP-seq(GSE106676)/Homer	1e-2	-5.272e+00	0.0134	2354.0	17.83%	5147.3	16.99%	motif file (matrix)	svg
386	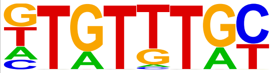	PHA-4(Forkhead)/cElegans-Embryos-PHA4-ChIP-Seq(modEncode)/Homer	1e-2	-5.262e+00	0.0135	8131.0	61.60%	18334.4	60.51%	motif file (matrix)	svg
387	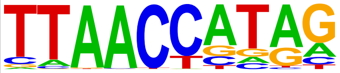	AT3G25990(Trihelix)/colamp-AT3G25990-DAP-Seq(GSE60143)/Homer	1e-2	-5.252e+00	0.0136	2046.0	15.50%	4456.0	14.71%	motif file (matrix)	svg
388	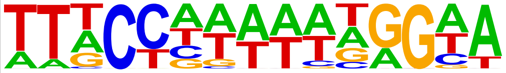	AGL6(MADS)/col-AGL6-DAP-Seq(GSE60143)/Homer	1e-2	-5.202e+00	0.0143	287.0	2.17%	565.9	1.87%	motif file (matrix)	svg
389	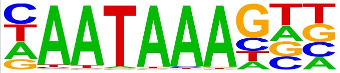	PABPC1(?)/MEL-PABC1-CLIP-Seq(GSE69755)/Homer	1e-2	-5.193e+00	0.0144	4557.0	34.53%	10143.8	33.48%	motif file (matrix)	svg
390	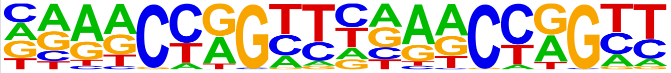	Tcfcp2l1(CP2)/mES-Tcfcp2l1-ChIP-Seq(GSE11431)/Homer	1e-2	-5.185e+00	0.0144	939.0	7.11%	1987.0	6.56%	motif file (matrix)	svg
391	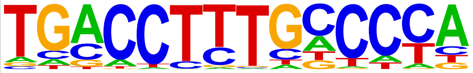	PPARE(NR),DR1/3T3L1-Pparg-ChIP-Seq(GSE13511)/Homer	1e-2	-5.079e+00	0.0160	5565.0	42.16%	12449.8	41.09%	motif file (matrix)	svg
392	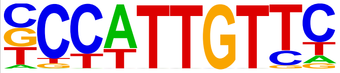	Sox2(HMG)/mES-Sox2-ChIP-Seq(GSE11431)/Homer	1e-2	-4.998e+00	0.0173	3090.0	23.41%	6819.9	22.51%	motif file (matrix)	svg
393	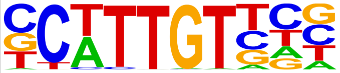	Sox10(HMG)/SciaticNerve-Sox3-ChIP-Seq(GSE35132)/Homer	1e-2	-4.944e+00	0.0182	5470.0	41.44%	12238.8	40.39%	motif file (matrix)	svg
394	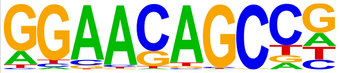	ZNF341(Zf)/EBV-ZNF341-ChIP-Seq(GSE113194)/Homer	1e-2	-4.933e+00	0.0184	4652.0	35.25%	10371.5	34.23%	motif file (matrix)	svg
395	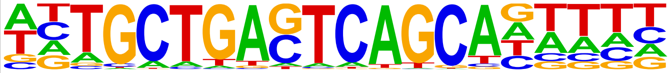	MafB(bZIP)/BMM-Mafb-ChIP-Seq(GSE75722)/Homer	1e-2	-4.924e+00	0.0185	1574.0	11.93%	3407.0	11.24%	motif file (matrix)	svg

396	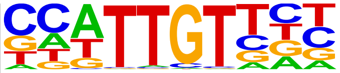	Sox6(HMG)/Myotubes-Sox6-ChIP-Seq(GSE32627)/Homer	1e-2	-4.904e+00	0.0188	4733.0	35.86%	10557.7	34.84%	motif file (matrix)	svg
397	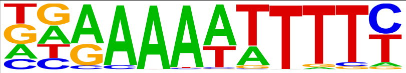	SFP1/SacCer-Promoters/Homer	1e-2	-4.888e+00	0.0191	241.0	1.83%	471.9	1.56%	motif file (matrix)	svg
398	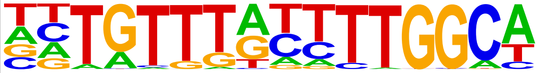	NF1:FOXA1(CTF,Forkhead)/LNCAP-FOXA1-ChIP-Seq(GSE27824)/Homer	1e-2	-4.884e+00	0.0191	174.0	1.32%	330.8	1.09%	motif file (matrix)	svg
399	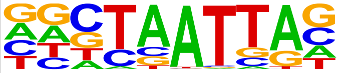	En1(Homeobox)/SUM149-EN1-ChIP-Seq(GSE120957)/Homer	1e-2	-4.764e+00	0.0215	4785.0	36.25%	10682.5	35.26%	motif file (matrix)	svg
400	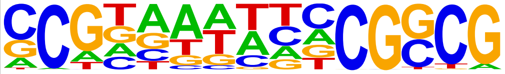	AS2(LOBAS2)/col-AS2-DAP-Seq(GSE60143)/Homer	1e-2	-4.733e+00	0.0221	1290.0	9.77%	2778.3	9.17%	motif file (matrix)	svg
401	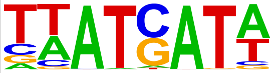	CRC(C2C2YABBY)/col-CRC-DAP-Seq(GSE60143)/Homer	1e-2	-4.728e+00	0.0222	2255.0	17.08%	4943.5	16.32%	motif file (matrix)	svg
402	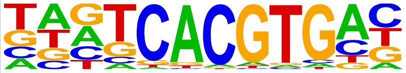	BIM3(bHLH)/col-BIM3-DAP-Seq(GSE60143)/Homer	1e-2	-4.711e+00	0.0225	925.0	7.01%	1967.7	6.49%	motif file (matrix)	svg
403	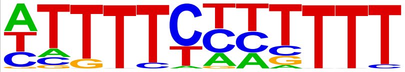	RLR1?/SacCer-Promoters/Homer	1e-2	-4.696e+00	0.0228	2427.0	18.39%	5332.5	17.60%	motif file (matrix)	svg
404	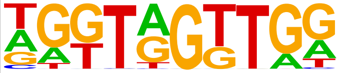	MYB96(MYB)/colamp-MYB96-DAP-Seq(GSE60143)/Homer	1e-2	-4.683e+00	0.0230	3900.0	29.55%	8670.6	28.62%	motif file (matrix)	svg
405	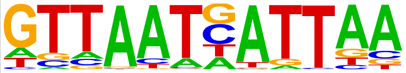	HNF1b(Homeobox)/PDAC-HNF1B-ChIP-Seq(GSE64557)/Homer	1e-2	-4.634e+00	0.0241	320.0	2.42%	643.7	2.12%	motif file (matrix)	svg
